# Enantioselective reductive multicomponent coupling reactions between isatins and aldehydes†
†Electronic supplementary information (ESI) available: Experimental procedures and characterizations of compounds. CCDC 1055582. For ESI and crystallographic data in CIF or other electronic format see DOI: 10.1039/c5sc02170g


**DOI:** 10.1039/c5sc02170g

**Published:** 2015-07-30

**Authors:** Matthew A. Horwitz, Naoya Tanaka, Takuya Yokosaka, Daisuke Uraguchi, Jeffrey S. Johnson, Takashi Ooi

**Affiliations:** a Department of Chemistry , The University of North Carolina at Chapel Hill , Chapel Hill , NC 27599 , USA . Email: jsj@unc.edu; b Institute of Transformative Bio-Molecules (WPI-ITbM) and Department of Applied Chemistry , Graduate School of Engineering , Nagoya University , Furo-cho D2-1, Chikusa , Nagoya 464-8602 , Japan . Email: tooi@apchem.nagoya-u.ac.jp; c CREST , Japan Science and Technology Agency (JST) , Nagoya University , Nagoya 464-8603 , Japan

## Abstract

A metal-free stereoselective reductive coupling reaction between isatins and aldehydes is reported. The reaction relies on commercial diethyl phosphite (∼€70 kg^–1^) as the stoichiometric reductant. Base-catalyzed Pudovik addition and phosphonate/phosphate rearrangement achieved polarity inversion on the isatin, and the derived carbanions were trapped by aldehydes with subsequent dialkoxyphosphinyl migration. Chiral iminophosphoranes were used as basic catalysts to achieve high diastereo- and enantioselectivities with excellent yields.

## Abstract 2

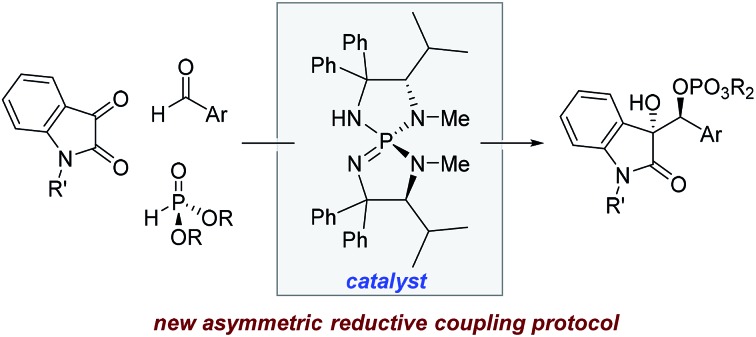
A reductive coupling of two different carbonyls *via* a polar two-electron reaction mechanism was developed and the stereochemical outcome of this multicomponent process is precisely controlled by a chiral triaminoiminophosphorane.

## 

The reductive coupling of π-unsaturation is a powerful method for the construction of carbon–carbon bonds. When the two coupling partners are prochiral, there exists the opportunity to establish multiple stereogenic centers concurrent with C–C bond formation. In the specific case of two carbonyl reactants, reductive coupling offers an attractive and straightforward method for the synthesis of vicinal diols, valuable building blocks in organic chemistry. A generic carbonyl reductive coupling manifold encompasses many mechanistic subtypes,^1^ but the pinacol reaction is preeminent among them. The traditional pinacol coupling entails single-electron reduction of the carbonyl functionality to generate the corresponding ketyl radical and subsequent dimerization between two radical species. The reaction has been studied extensively using low-valent metals in this single-electron transfer manifold.^2^–^6^ Despite numerous advances, however, myriad challenges remain: a stoichiometric or superstoichiometric amount of metal agents is often required and there are sparse examples that use catalytic conditions.4n–r Moreover, the nature of the mechanism can render it difficult to control both chemoselectivity (homo- *versus* cross-coupling) and stereoselectivity, and the lack of differentiation of the nascent alcohols can be nettlesome. These precedents collectively informed our interest in developing an alternative, potentially generalizable reductive coupling strategy that utilizes a polar two-electron reaction mechanism for addressing the aforementioned issues. The purpose of this communication is to detail a new base-catalyzed cross coupling of carbonyls mediated by an economical organic reductant, diethyl phosphite; the stereochemical outcome of this multicomponent process is precisely controlled by a chiral triaminoiminophosphorane (Figure 1a).^7^,^8^

**Fig. 1:**
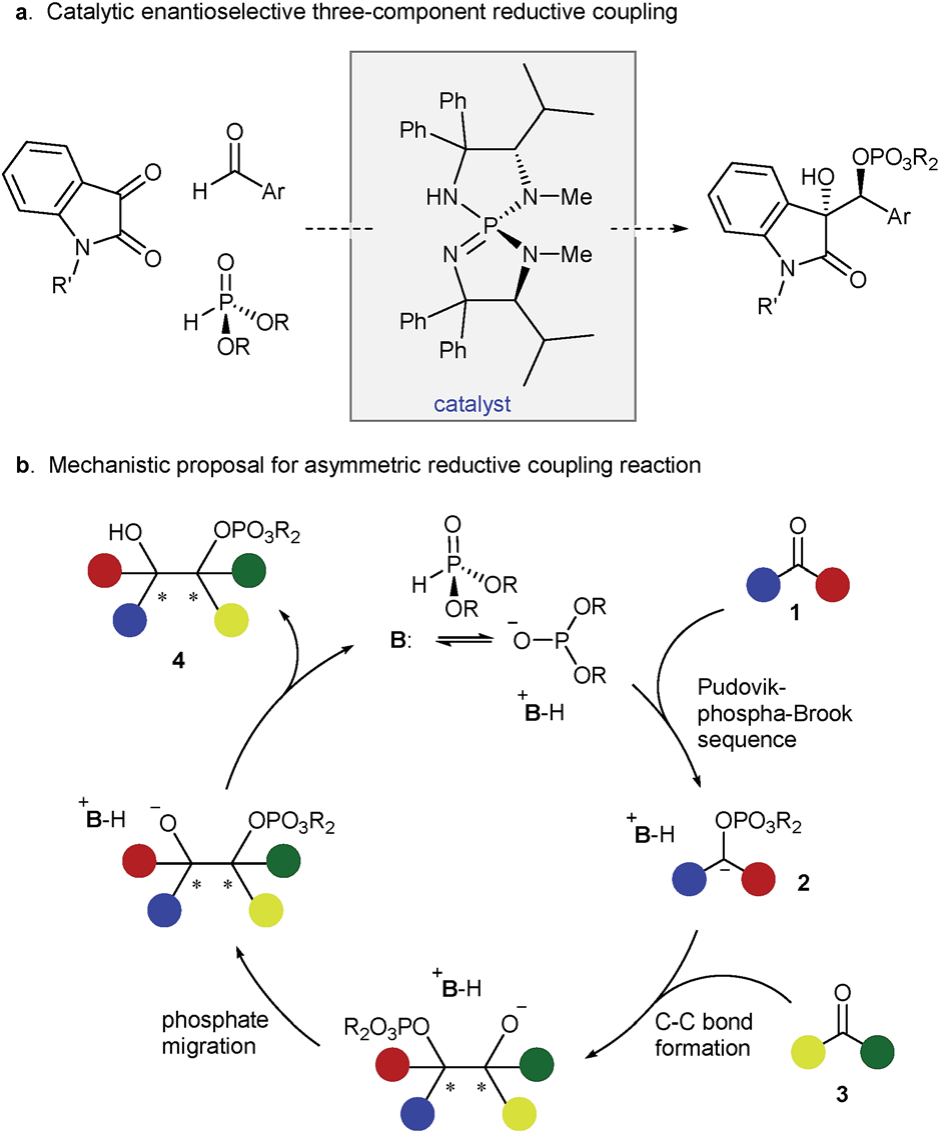
Stereoselective reductive coupling reactions.

At the outset, we envisaged the possibility of catalytic generation of an α-oxycarbanion from a carbonyl substrate and its rapid and selective trapping with another carbonyl compound to form 1,2-diols. For substantiating this hypothesis, polarity reversal of a particular carbonyl group is of critical importance and we sought to take advantage of the phosphonate–phosphate (phospha-Brook) rearrangement to achieve this requisite process. Thus, a base-catalyzed sequence of Pudovik addition and phosphonate–phosphate rearrangement between ketone **1** and dialkyl phosphite was projected to lead to carbanion **2**. The interception of this key intermediate by aldehyde **3** would afford mono-protected diol **4** through dialkoxyphosphinyl migration (Figure 1b).^9^ A crucial departure from prior art is the fully intermolecular nature of the coupling and the need for the phosphite to exhibit complete selectivity between the two carbonyl reactants. We reasoned that the crucial chemoselectivity issue underlying this mechanistic framework, *viz.* the selective generation of α-oxycarbanion **2** from ketone **1**, would be ensured by the inherent reversibility of Pudovik reaction and the reluctance of the aldehyde Pudovik product to undergo phospha-Brook rearrangement. In addition, absolute stereochemical guidance in the C–C bond-forming event could be provided by the conjugate acid of a suitable chiral base. In providing the conceptual blueprint for this scenario, we focused our attention on the exceptional electrophilicity and utility of α-dicarbonyls.9d–g,^10^

Steps were initially taken to assess the feasibility of the proposed reaction in a racemic sense using achiral bases such as potassium *tert*-butoxide (KO^*t*^Bu). Initial trials with diethyl phosphite as the stoichiometric reductant indicated that the reaction proceeds most cleanly and efficiently when a protecting group is used on the isatin. Benzyl, allyl, and methyl protecting groups were examined using 20 mol% KO^*t*^Bu in THF at 0 °C (Table 1, (±)-**4a**–(±)-**4c**). Under these conditions, the reactions were complete in minutes with no observable intermediates (if the aldehyde is omitted from the reaction, the Pudovik-phospha-Brook product can be observed, however).9*f* These experiments revealed that the benzyl protecting group provided the highest isolated yield and diastereoselectivity. We subsequently verified that *para*-tolualdehyde is not capable of phospha-Brook rearrangement when treated with diethyl phosphite and 20 mol% KO^*t*^Bu: only the Pudovik adduct was observed, implying that it is the isatin that is undergoing polarity reversal as we expected.

**Table 1:** Three component reductive coupling: racemic^*a*^

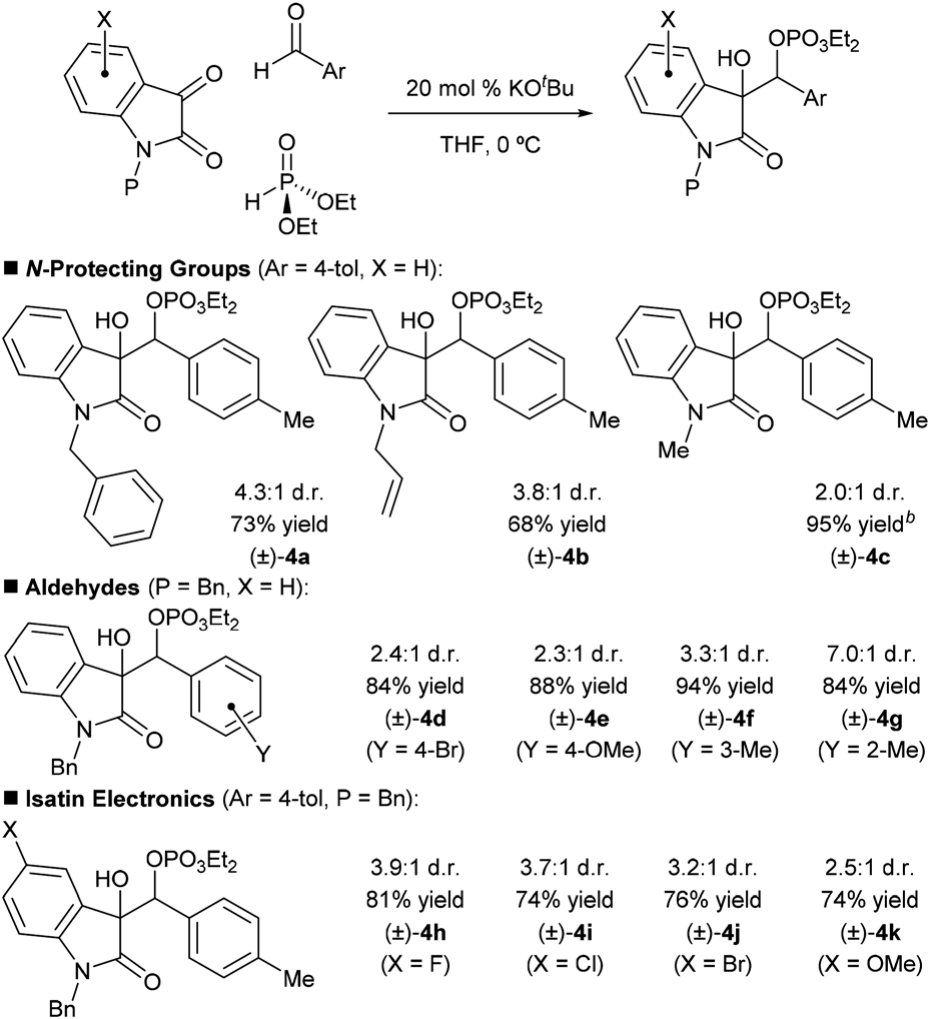

^*a*^All reactions were run on 0.2 mmol scale, using 1.1 equiv. of dialkylphosphite and 5.0 equiv. of aldehyde. % Yields refer to isolated yields. All d.r. and % yield values are the averages of two trials. Reactions were run until complete as adjudged by TLC.

^*b*^% Yield determined by crude ^1^H NMR using mesitylene as an internal standard. Products derived from apparent retro-reaction significantly diminished the isolated yield; therefore, this substrate was not selected for further study.

We then briefly studied the scope of the racemic reaction. The reaction gives consistently good yields for various aryl aldehydes incorporating substituents of different electronic properties (Table 1, (±)-**4d**–(±)-**4g**). At the current level of optimization, alkyl aldehydes and Boc-protected imine electrophiles were not well tolerated and only provided messy reactions.^11^ The substitution pattern of the isatin was also examined; we found that the racemic reaction is reasonably flexible in terms of isatin electronics ((±)-**4h**–(±)-**4k**).

Efforts were next directed to the development of the enantioselective variant.^12^ We were encouraged to find that when we used the chiral iminophosphorane (**C1**), we obtained the secondary phosphate **4a** with appreciable enantioenrichment (er 89.5 : 10.5), although the diastereoselectivity was poor (Table 2, entry 1). Gratifyingly, we found that upon lowering the temperature to –78 °C, phosphate **4a** was obtained in 82% yield, 15 : 1 diastereoselectivity and an er of 96.5 : 3.5 (entry 2). Using the same temperature, we proceeded to evaluate the effect of the catalyst structure (entries 3 to 6), but ultimately concluded that α-branching in ligand substituent R is essential for promoting the desired transformations and the valine-derived iminophosphorane **C1** was optimal in terms of stereoselectivity and chemical yield.

**Table 2:** Optimization of the asymmetric catalytic reductive coupling^*a*^

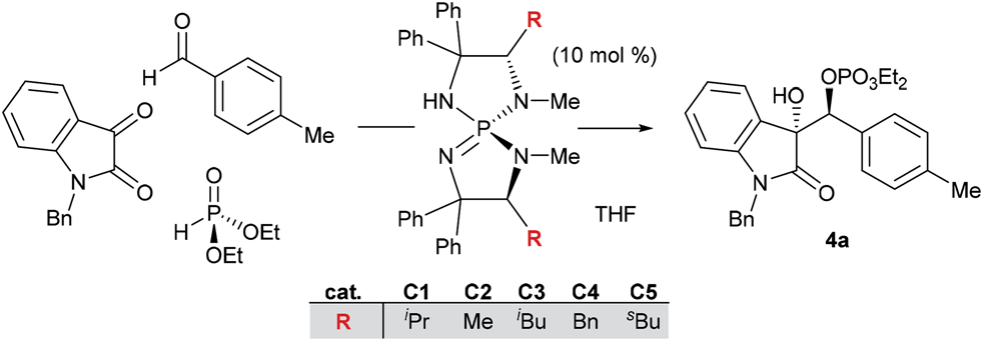					
Entry	T (°C)	Catalyst	d.r.	e.r.	% Conv.
1	0	**C1**	3.4 : 1	89.5 : 10.5	96
2	–78	**C1**	15 : 1	96.5 : 3.5	82
3	–78	**C2**	n.a.	n.a.	18
4	–78	**C3**	n.a.	n.a.	15
5	–78	**C4**	n.a.	n.a.	12
6	–78	**C5**	7.9 : 1	86 : 14	80

^*a*^All reactions were conducted on a 0.1 mmol scale, using 1.1 equiv. of dialkylphosphite and 5.0 equiv. of 4-tolualdehyde. Argon was used to purge the reaction flasks. All d.r., e.r., and % conversion values are the average of two trials. n.a. = not analyzed.

The disparity between the stereoselectivities at 0 °C and –78 °C prompted us to investigate the reversibility of the carbon–carbon bond formation *via* crossover experiments in that temperature range (Table 3). When racemic phosphate (±)-**4a** was subjected to standard conditions in the presence of 4-fluorobenzaldehyde, significant incorporation of that component in the form of phosphate **4a–F** was observed at 0 °C and –40 °C, but no crossover was observed at –78 °C. These data support the hypothesis that the increase in enantioselectivity at –78 °C is not only a consequence of more rigorous facial discrimination of both substrates but also shutting down a stereoablative retro-aldol process that is operative at higher temperatures.

**Table 3:** Crossover experiments establish reversibility^*a*^

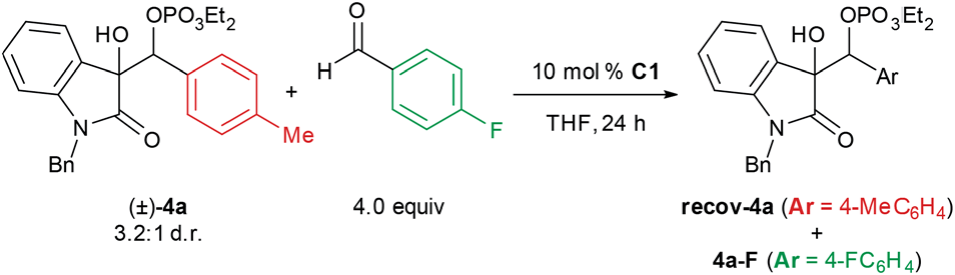		
Entry	T (°C)	**4a** : **4a–F**
1	0	1.0 : 1.5
2	–40	1.0 : 1.1
3	–78	Only **4a**

^*a*^Product distributions were determined by ^1^H NMR analysis (800 MHz) of the crude mixture.

Using the optimized conditions, we evaluated the scope of the asymmetric reaction by initially looking at various isatins. While electron-deficient 5-halogenated isatins were well accommodated under the optimized conditions, use of dimethyl phosphite was indispensable for completion of the reactions with 5-methyl and methoxy isatins probably because of the slow phospha-Brook rearrangement (Table 4, **4h–4m**).^13^ 6-Chloro and 7-fluoro isatins were also smoothly converted into the reductive coupling products of high stereochemical purity using appropriate phosphite (**4n** and **4o**). The absolute stereochemistry was determined at this stage by an X-ray diffraction study of phosphate **4j** (Fig. 2).^14^

**Table 4:** Scope of asymmetric reaction^*a*^

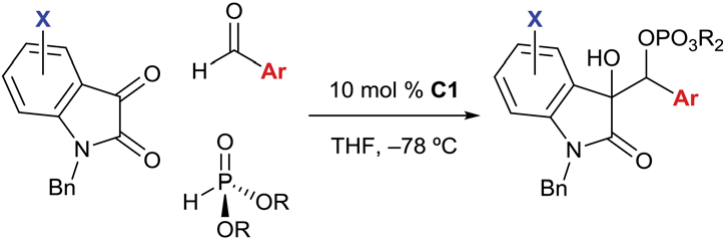
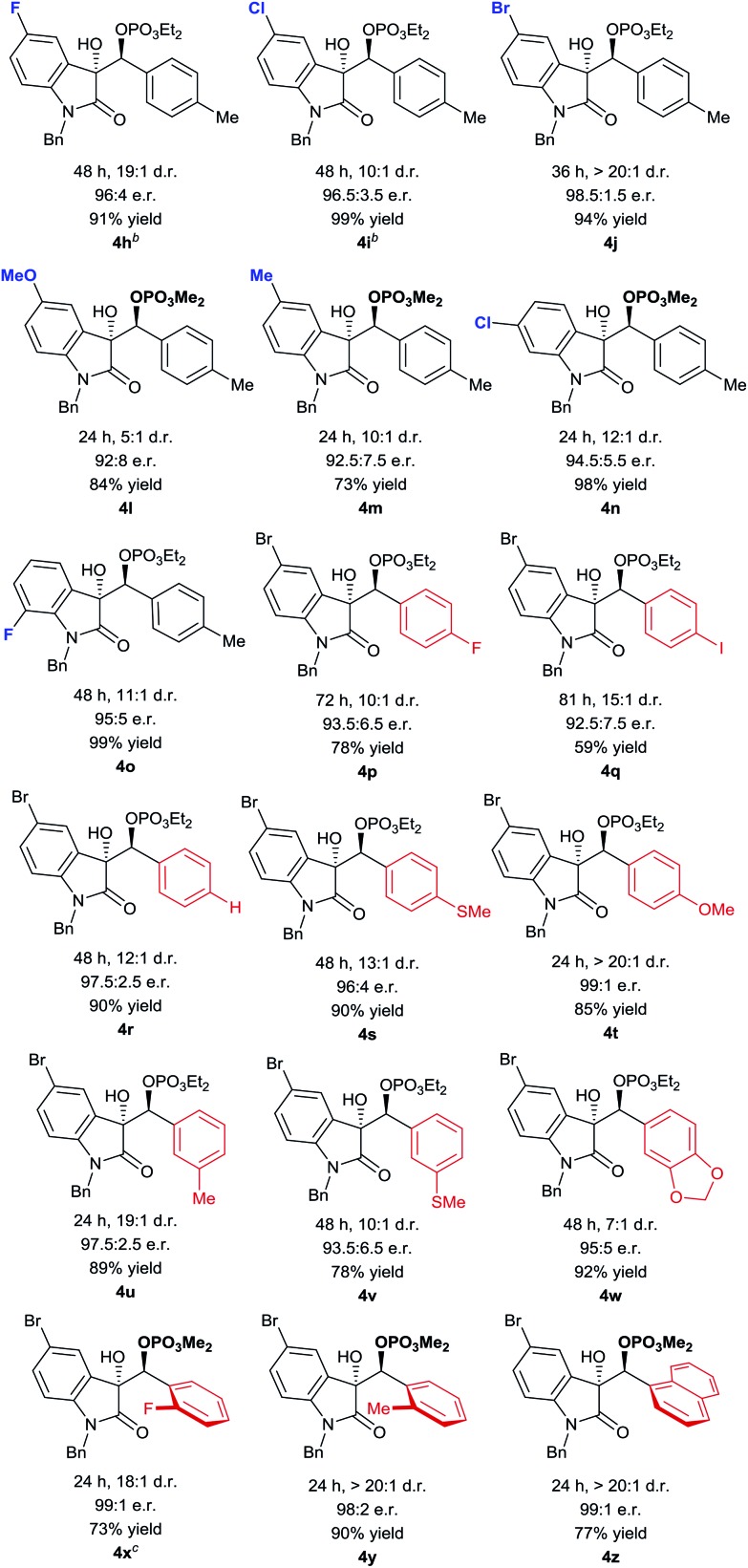

^*a*^All reactions were conducted on a 0.1 mmol scale, using 1.1 equiv. of dialkylphosphite and 5.0 equiv. of ArCHO. Argon was used to purge the reaction flasks. % Yields refer to isolated yields. All d.r., e.r., and % yield values are the average of two trials.

^*b*^15 mol% of catalyst was used.

^*c*^2.2 equiv. of dialkylphosphite was used.

**Fig. 2:**
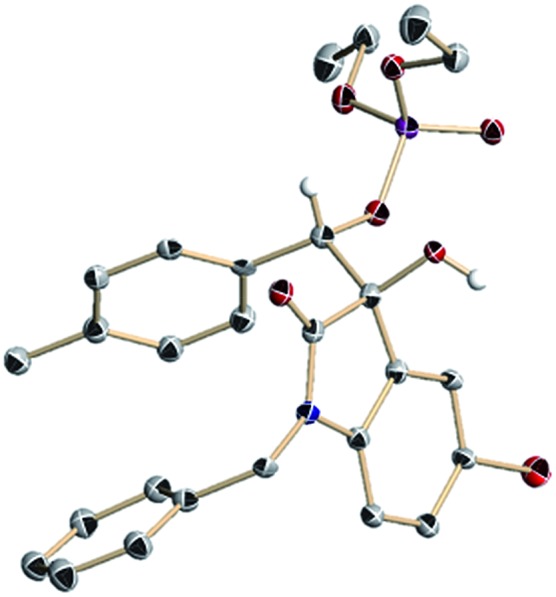
ORTEP diagram of **4j** (ellipsoids displayed at 50% probability. Calculated hydrogen atoms except for that attached to the stereogenic carbon atom are omitted for clarity. Black: carbon, red: oxygen, purple: phosphorous, blue: nitrogen, vermilion: bromine, white: hydrogen).

For exploration of aldehyde generality, we selected 5-bromo isatin as a coupling partner in consideration of its high reactivity and advantage of having an additional functional handle at the aromatic nuclei. As included in Table 4, various *para*-substituted aromatic aldehydes were tolerated and relatively electron rich aldehydes exhibited higher reactivity and selectivity (**4p–4t**). Hetero-substituents at the *meta*-position slightly affected the stereochemical outcome (**4u–4w**). For sterically demanding *ortho*-substituted aldehydes, dimethyl phosphite was needed to accelerate the reaction and virtually complete stereocontrol could be achieved (**4x–4z**).

In summary, we have developed a highly stereoselective, fully organic multicomponent coupling reaction between isatins and aldehydes with dialkyl phosphite as an economical reductant. The advantages of extending the reductive coupling into a two-electron manifold are manifest, and the mechanistic framework established herein may be applicable to other stereoselective reductive carbon–carbon bond constructions. Efforts to exploit this reaction paradigm in other systems are ongoing in our laboratories.

## Supplementary Material

Supplementary information

Crystal structure data
