# Supplementary material for: Enantioselective reductive multicomponent coupling reactions between isatins and aldehydes
Source: Chem Sci. 2015 Jul 30;6(11):6086–90. doi: 10.1039/c5sc02170g (PMC4618493; doi:10.1039/c5sc02170g)
Supplement: Supplementary file 1 [file SC-006-C5SC02170G-s001.pdf]

Electronic Supplementary Material (ESI) for Chemical Science.
This journal is © The Royal Society of Chemistry 2015

Electronic Supplementary Information

Enantioselective Reductive Multicomponent Coupling Reactions between Isatins and Aldehydes

Matthew A. Horwitz,[§a] Naoya Tanaka,[§b] Takuya Yokosaka,[a] Daisuke Uraguchi,[b] Jeffrey S. Johnson,[*a] Takashi Ooi[*bc]

[a] Department of Chemistry, University of North Carolina at Chapel Hill, Chapel Hill, North Carolina 27599-3290, United States

[b] Institute of Transformative Bio-Molecules (WPI-ITbM), and Department of Applied Chemistry, Graduate School of Engineering, Nagoya University, Chikusa, Nagoya 464-8602, Japan

[c] CREST, Japan Science and Technology Agency (JST), Chikusa, Nagoya 464-8603, Japan

[§] These authors contributed equally.

jsj@unc.edu

tooi@apchem.nagoya-u.ac.jp

Table of Contents

Table of Contents	Page
General Information (Materials and Methods)	S2
Racemic three component reactions using KO*t*Bu	S3-S5
Enantioselective three component reactions using chiral iminophosphorane	S6-S14
Details of crossover experiment	S15
Crystallographic structure determination	S16-17
References	S17
Crude ^{1}H NMR spectra for the racemic reaction	S18-S27
Crude ^{1}H NMR spectra for the asymmetric reaction	S28-S45
HPLC traces	S46-S64
^{1}H and ^{13}C NMR spectra of new compounds	S65-S118

General Information:

Methods: Infrared (IR) spectra were obtained using an ASI ReactIR 1000 Fourier transform infrared spectrometer or a Shimadzu IRAffinity-1 spectrometer. Magnetic resonance spectra (^{1}H, ^{13}C, ^{19}F, and ^{31}P NMR) were recorded on a Bruker model DRX 400 (^{1}H NMR at 400 MHz, ^{13}C NMR at 101 MHz, ^{19}F NMR at 376 MHz, and ^{31}P NMR at 162 MHz), a Bruker model DRX 600 (^{1}H NMR at 600 MHz, ^{13}C NMR at 151 MHz, and ^{31}P NMR at 243 MHz), a JEOL JNM-ECS400 (^{1}H NMR at 400 MHz, ^{19}F NMR at 376 MHz, and ^{31}P NMR at 162 MHz), ECA-800 (^{1}H NMR at 800 MHz), a Bruker AVANCE III-OneBay500 (^{13}C NMR at 126 MHz) spectrometer with solvent resonance as the internal standard (^{1}H NMR: $CDCl_3$ at 7.26 ppm and ^{13}C NMR: $CDCl_3$ at 77.16 ppm), or benzotrifluoride (^{19}F NMR: -64.0 ppm) and H_3PO_4 (^{31}P NMR: 0.0 ppm) resonances as the external standard. ^{1}H NMR data are reported as follows: chemical shift, multiplicity (s = singlet, br = broad, d = doublet, dd = doublet of doublet, t = triplet, dt = doublet of triplet, m = multiplet), coupling constants (Hz), and integration. High resolution mass spectra were obtained with a Thermo Fisher Scientific Exactive, Finnigan™ LTQ-ICR FT™, or Micromass Quattro II (triple quad) instrument with nanoelectrospray ionization (all samples prepared in methanol). Melting points were obtained using a Stanford Research Systems OptiMelt MPA100 or Thomas Hoover UniMelt Capillary Melting Point Apparatus. Analytical thin layer chromatography was carried out using Whatman 0.25 mm silica gel 60 plates, Sorbent Technologies 0.20 mm Silica Gel TLC plates, or Merck precoated TLC plates (silica gel 60 GF254, 0.25 mm). Visualization was allowed by UV light, phosphomolybdic acid in ethanol, or aqueous ceric ammonium nitrate solution. HPLC analysis was performed on a Shimadzu SPD-M20A PDA detector with a Shimadzu SPD20AD eluent system using DAICEL CHIRALPAK IA or AD3 columns (ϕ 4.6 mm x 250 mm, constant flow at 1.00 mL/min), using hexane, 2-propanol, and ethanol as eluents. To perform HPLC trials at 4 ºC, a Shimadzu LC-2010C HT unit was used. Asymmetric reactions were carried out under an atmosphere of argon, in oven-dried glass with magnetic stirring, using a UC Reactor (Techno Sigma) or a PSL-1810 (EYELA) reactor. Purification of the reaction products was carried out by using Siliaflash-P60 silica gel (40-63 μm) purchased from Silicycle, or silica gel 60 (spherical, 40-50 μm) from Kanto Chemical Co., Inc. Yields refer to isolated yields after flash column chromatography; some samples contain residual minor diastereomers. Since all results are the averages of two trials, the stereochemical outcomes listed in the paper may not exactly match those represented in the NMR and HPLC data below.

Materials: Tetrahydrofuran (THF) was supplied from Kanto Chemical Co., Inc. as "Dehydrated solvent system" and further purified by passing through neutral alumina under nitrogen atmosphere. Isatins were purchased from Acros Organics or Wako Chemical Co. and alkylated according to literature procedures.[1] Triaminoiminophosphorane catalysts **C1-C5** were prepared according to literature procedures.[2] Commercially available dimethyl phosphite, diethyl phosphite, and diisopropyl phosphite were distilled using a Kügelrohr apparatus prior to use. Commerically available aldehydes were freshly distilled directly before the reactions. Potassium *tert*-butoxide was purchased from Sigma Aldrich and used as is.

General procedure for the three component reaction using KO^{t}Bu:

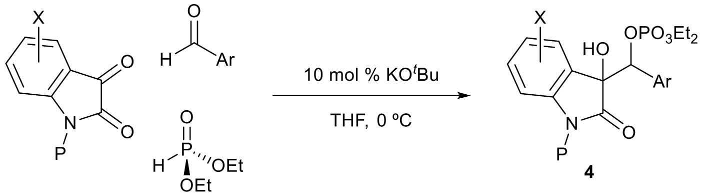

To a stirred solution of isatin derivative (0.20 mmol), diethyl phosphite (30.4 mg, 0.22 mmol, 1.1 equiv) and aldehyde (1.0 mmol, 5.0 equiv) in THF (2.0 mL) at 0 °C was added KO^{t}Bu (2.2 mg, 0.02 mmol, 10 mol%). The reaction was allowed to proceed at the same temperature and was followed by TLC. Once the isatin was fully consumed (typically 5-10 minutes), the reaction mixture was quenched with saturated aqueous NH_4Cl, and extracted twice with EtOAc. The combined organic layers were washed with brine, dried over Na_2SO_4, filtered, and concentrated *in vacuo*. The residue was purified by silica gel column chromatography to give the desired products **4**.

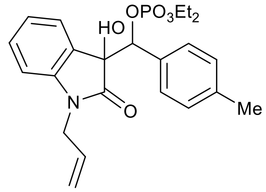

1-Allyl-3-((((diethyl-λ^3-oxidanyl)(λ^1-oxidanyl)phosphoryl)oxy)(*p*-tolyl)methyl)-3-hydroxyindolin-2-one ((±)-4b): The title compound was prepared according to general procedure. The diastereomeric ratio was determined by ^{1}H NMR spectroscopic analysis of the crude reaction mixture by comparison of the resonances at δ 6.58 (minor diastereomer) and δ 6.53 (major diastereomer). Clear oil; **^{1}H NMR** (600 MHz, $CDCl_3$) δ 7.71 (d, *J* = 7.3 Hz, 1H), 7.30-7.27 (m, 1H), 7.16-7.13 (m, 1H), 6.91 (d, *J* = 8.0 Hz, 2H), 6.85 (d, *J* = 8.0 Hz, 2H), 6.56 (d, *J* = 7.7 Hz, 1H), 5.72 (d, *J* = 8.6 Hz, 1H), 5.28-5.22 (m, 1H), 4.87 (d, *J* = 10.4 Hz, 1H), 4.48-4.44 (m, 2H), 4.29-4.20 (m, 2H), 4.14-4.01 (m, 2H), 3.77 (dd, *J* = 5.4, 16.8 Hz, 1H), 2.24 (s, 3H), 1.32 (t, *J* = 7.2 Hz, 3H), 1.24 (t, *J* = 7.2 Hz, 3H), OH-proton was not found probably due to broadening; **^{13}C NMR** (151 MHz, $CDCl_3$) δ 174.5, 143.2, 138.7, 130.9, 130.4, 130.0, 128.4, 127.6, 126.2, 122.8, 118.1, 116.8, 109.0, 83.5 (d, *J* = 5.6 Hz), 79.3 (d, *J* = 4.5 Hz), 64.5 (d, *J* = 5.7 Hz), 64.3 (d, *J* = 5.9 Hz), 42.0, 21.0, 16.1 (d, *J* = 6.8 Hz), 16.0 (d, *J* = 7.1 Hz); **^{31}P NMR** (162 MHz, $CDCl_3$) δ 0.23; **IR** (thin film) ν 3288.0, 2984.3, 1725.0, 1614.13, 1468.5, 1368.3, 1257.4, 1028.8, 754.0, 663.4 cm^{-1}; **HRMS** (ESI$^+$) Calcd. for $C_{23}H_{28}NNaO_6P$ 468.155197 (M+Na$^+$) found 468.1565; **TLC** (2:1 EtOAc/Hexanes): R_f = 0.17.

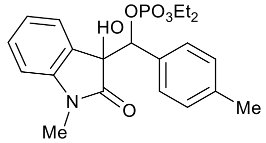

3-((((Diethyl-λ^3-oxidanyl)(λ^1-oxidanyl)phosphoryl)oxy)(*p*-tolyl)methyl)-3-hydroxy-1-methylindolin-2-one ((±)-4c): The title compound was prepared according to general procedure. The diastereomeric ratio was determined by ^{1}H NMR spectroscopic analysis of the crude reaction mixture by comparison of the resonances at δ 6.59 (minor diastereomer) and δ 6.51 (major diastereomer). The product was found to be unstable to silica gel chromatography. The percent yield was calculated by ^{1}H NMR spectroscopy using mesitylene as an internal standard.

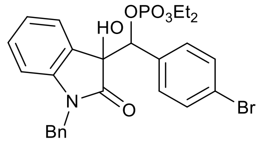

1-Benzyl-3-((4-bromophenyl)(((diethyl-λ^3-oxidanyl)(λ^1-oxidanyl)phosphoryl)oxy)methyl)-3-hydroxyindolin-2-one ((±)-4d): The title compound was prepared according to general procedure. The diastereomeric ratio was determined by ^{1}H NMR spectroscopic analysis of the crude reaction mixture by comparison of the resonances at δ 4.92 (minor diastereomer) and δ 4.83 (minor diastereomer). White solid (mp 168-169 °C); **^{1}H NMR** (400 MHz, $CDCl_3$) δ 7.73 (d, *J* = 7.2 Hz, 1H), 7.26-7.16 (m, 6H), 7.11 (t, *J* = 7.6 Hz, 1H), 6.89 (d, *J* = 8.0 Hz, 2H), 6.57-6.55 (m, 2H), 6.42 (d, *J* = 8.0 Hz, 1H), 5.78 (d, *J* = 8.8 Hz, 1H), 5.00 (d, *J* = 16.0 Hz, 1H), 4.71 (br, 1H), 4.28 (d, *J* = 16.0 Hz, 1H), 4.22-4.07 (m, 2H), 4.00 (quintet, *J* = 7.2 Hz, 2H), 1.29 (t, *J* = 6.8 Hz, 3H), 1.20 (t, *J* = 6.8 Hz, 3H); **^{13}C NMR** (151 MHz, $CDCl_3$) δ 174.8, 143.2, 134.6, 133.3 (d, *J* = 3.5 Hz), 131.2, 130.3, 129.7, 128.7, 127.5, 126.5, 126.3, 126.0, 123.2, 123.1, 109.5, 82.9 (d, *J* = 5.7 Hz), 78.9 (d, *J* = 4.7 Hz), 64.6 (d, *J* = 5.6 Hz), 64.4 (d, *J* = 5.6 Hz), 43.8, 16.1 (d, *J* = 7.2 Hz), 16.0 (d, *J* = 6.6 Hz); **^{31}P NMR** (243 MHz, $CDCl_3$) δ -0.6; **IR** (thin film) ν 3402.8, 2984.3, 1720.2, 1614.1, 1488.8, 1369.2, 1251.6, 1028.8, 1007.6, 971.9 cm^{-1}; **HRMS** (ESI$^+$) Calcd. for $C_{26}H_{27}BrNO_6P$ 560.0838 (M+H$^+$) found 560.0820; **TLC** (2:1 EtOAc/Hexanes): R_f = 0.33.

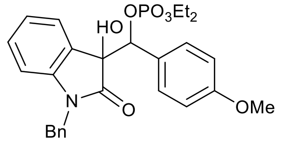

1-Benzyl-3-((((diethyl-λ^3-oxidanyl)(λ^1-oxidanyl)phosphoryl)oxy)(4-methoxyphenyl)methyl)-3-hydroxyindolin-2-one ((±)-4e): The title compound was prepared according to general procedure. The diastereomeric ratio was determined by ^{1}H NMR spectroscopic analysis of the crude reaction mixture by comparison of the resonances at δ 5.76 (major diastereomer) and δ 5.71 (minor diastereomer). White solid (mp 157-158 °C); **^{1}H NMR** (400 MHz, $CDCl_3$) δ 7.75 (d, *J* = 7.2 Hz, 1H), 7.21-7.05 (m, 5H), 6.91 (d, *J* = 8.4 Hz, 2H), 6.62 (d, *J* = 8.4 Hz, 2H), 6.48 (d, *J* = 7.6 Hz, 2H), 6.37 (d, *J* = 7.6 Hz, 1H), 5.78 (d, *J* = 8.8 Hz, 1H), 4.99 (d, *J* = 16.0 Hz, 1H), 4.76 (s, 1H), 4.24 (d, *J* = 16.0 Hz, 1H), 4.21-3.91 (m, 4H), 3.73 (s, 3H), 1.27 (t, *J* = 7.2 Hz, 3H), 1.20 (t, *J* = 7.2 Hz, 3H); **^{13}C NMR** (151 MHz, $CDCl_3$) δ 174.9, 160.1, 143.3, 134.7, 130.1, 129.4, 128.4, 127.3, 126.5, 126.4, 126.3, 126.2 (d, *J* = 4.1 Hz), 123.0, 113.4, 109.4, 83.4 (d, *J* = 6.2 Hz), 79.2 (d, *J* = 5.0 Hz), 64.4 (d, *J* = 5.6 Hz), 64.3 (d, *J* = 5.6 Hz), 55.1, 43.6, 16.1 (d, *J* = 7.2 Hz), 16.0 (d, *J* = 6.9 Hz); **^{31}P NMR** (243 MHz, $CDCl_3$) δ -0.5; **IR** (thin film) ν 3416.3, 2983.3, 2931.3, 1721.2, 1614.1, 1514.8, 1441.4, 1368.3, 1250.5, 1027.9 cm^{-1}; **HRMS** (ESI$^+$) Calcd. for $C_{27}H_{30}NO_7P$ 512.1838 (M+H$^+$) found 512.1803; **TLC** (2:1 EtOAc/Hexanes): R_f = 0.19.

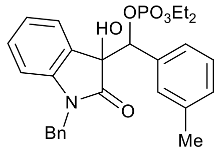

1-Benzyl-3-((((diethyl-λ^3-oxidanyl)(λ^1-oxidanyl)phosphoryl)oxy)(*m*-tolyl)methyl)-3-hydroxyindolin-2-one ((±)-4f): The title compound was prepared according to general procedure. The diastereomeric ratio was determined by ^{1}H NMR spectroscopic analysis of the crude reaction mixture by comparison of the resonances at δ 6.24 (major diastereomer) and δ 4.46 (minor diastereomer). White solid (mp 109-110 °C); **^{1}H NMR** (400 MHz, $CDCl_3$) δ 7.75 (d, *J* = 6.8 Hz, 1H), 7.19-6.89 (m, 7H), 6.82 (s, 1H), 6.78 (d, *J* = 7.6 Hz, 1H), 6.45 (d, *J* = 7.6 Hz, 2H), 6.33 (d, *J* = 7.6 Hz, 1H), 5.77 (d, *J* = 8.4 Hz, 1H), 4.96 (d, *J* = 16.4 Hz, 1H), 4.89 (br-s, 1H), 4.27 (d, *J* = 16.0 Hz, 1H), 4.22-3.95 (m, 4H), 2.10 (s, 3H), 1.26 (t, *J* = 6.8 Hz, 3H), 1.19 (t, *J* = 6.8 Hz, 3H); **^{13}C NMR** (151 MHz, $CDCl_3$) δ 174.9, 143.3, 137.7, 134.7,

133.9 (d, J = 3.0 Hz), 130.0, 129.7, 128.7, 128.6, 127.9, 127.2, 126.4, 126.3, 126.3, 125.0, 123.0, 109.4, 83.7 (d, J = 6.0 Hz), 79.2 (d, J = 4.5 Hz), 64.5 (d, J = 6.0 Hz), 64.4 (d, J = 6.0 Hz), 43.6, 21.2, 16.1 (d, J = 6.0 Hz), 16.0 (d, J = 7.6 Hz); **^{31}P NMR** (162 MHz, $CDCl_3$) δ -0.6; **IR** (thin film) ν 3290.0, 1724.1, 1614.1, 1468.4, 1366.3, 1250.6, 1027.9, 753.1, 699.1 cm^{-1}; **HRMS** (ESI^+) Calcd. for $C_{27}H_{30}NO_6P$ 496.1889 ($M+H^+$) found 496.1815; **TLC** (2:1 EtOAc/Hexanes): R_f = 0.25.

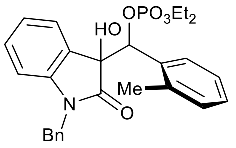

1-Benzyl-3-((((diethyl-λ^3-oxidanyl)(λ^1-oxidanyl)phosphoryl)oxy)(*o*-tolyl)methyl)-3-hydroxyindolin-2-one ((±)-4g): The title compound was prepared according to general procedure. The diastereomeric ratio was determined by ^{1}H NMR spectroscopic analysis of the crude reaction mixture by comparison of the resonances at δ 4.86 (major diastereomer) and δ 4.54 (minor diastereomer). White solid (mp 170-171 °C); **^{1}H NMR** (400 MHz, $CDCl_3$) δ 7.69 (d, J = 7.2 Hz, 1H), 7.26-7.04 (m, 7H), 6.82 (t, J = 7.6 Hz, 1H), 6.63 (d, J = 8.0 Hz, 1H), 6.46-6.39 (m, 3H), 6.13 (d, J = 8.8 Hz, 1H), 5.06 (d, J = 16.0 Hz, 1H), 4.61 (s, 1H), 4.21 (d, J = 16.0 Hz, 1H), 4.16-3.88 (m, 4H), 2.45 (s, 3H), 1.23 (t, J = 7.2 Hz, 3H), 1.16 (t, J = 6.8 Hz, 3H); **^{13}C NMR** (151 MHz, $CDCl_3$) δ 175.0, 143.4, 137.4, 134.8, 132.7 (d, J = 4.5 Hz), 130.5, 130.2, 128.9, 128.7, 127.8, 127.2, 126.6, 126.4, 126.3, 125.5, 123.1, 109.4, 79.1 (d, J = 4.5 Hz), 78.9 (d, J = 6.0 Hz), 64.3 (d, J = 4.5 Hz), 64.1 (d, J = 6.0 Hz), 43.7, 19.8, 16.0 (d, J = 7.6 Hz), 15.9 (d, J = 7.6 Hz); **^{31}P NMR** (162 MHz, $CDCl_3$) δ -0.5; **IR** (thin film) ν 3263.9, 2982.4, 2341.2, 1716.3, 1613.2, 1468.4, 1361.5, 1241.0, 1016.3, 753.1 cm^{-1}; **HRMS** (ESI^+) Calcd. for $C_{27}H_{30}NO_6P$ 496.1889 ($M+H^+$) found 496.1812; **TLC** (2:1 EtOAc/Hexanes): R_f = 0.29.

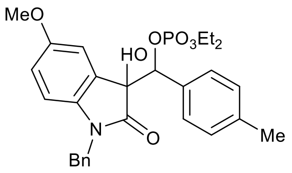

1-Benzyl-3-((((diethyl-λ^3-oxidanyl)(λ^1-oxidanyl)phosphoryl)oxy)(*p*-tolyl)methyl)-5-methoxy-3-hydroxyindolin-2-one ((±)-4k): The title compound was prepared according to general procedure. The diastereomeric ratio was determined by ^{1}H NMR spectroscopic analysis of the crude reaction mixture by comparison of the resonances at δ 6.24 (minor diastereomer) and δ 6.15 (major diastereomer). White solid (mp 128-129 °C); **^{1}H NMR** (400 MHz, $CDCl_3$) δ 7.35 (d, J = 2.4 Hz, 1H), 7.26-7.05 (m, 3H), 6.95-6.90 (m, 4H), 6.67 (dd, J = 2.4, 8.0 Hz, 1H), 6.52 (d, J = 7.6 Hz, 2H), 6.25 (d, J = 8.8 Hz, 1H), 5.77 (d, J = 8.8 Hz, 1H), 4.95 (d, J = 16.0 Hz, 1H), 4.76 (s, 1H), 4.25-4.07 (m, 2H), 3.99 (quintet, J = 7.2 Hz, 2H), 3.79 (s, 3H), 2.30 (s, 3H), 1.29 (t, J = 7.2 Hz, 3H), 1.19 (t, J = 6.8 Hz, 3H); **^{13}C NMR** (151 MHz, $CDCl_3$) δ 174.7, 156.1, 138.7, 136.5, 134.9, 131.1 (d, J = 3.8 Hz), 128.7, 128.4, 128.0, 127.7, 127.2, 126.6, 114.6, 113.4, 109.8, 83.6 (d, J = 5.6 Hz), 79.5 (d, J = 4.4 Hz), 64.4 (d, J = 5.6 Hz), 64.3 (d, J = 6.0 Hz), 55.8, 43.8, 21.3, 16.1 (d, J =6.8 Hz), 16.0 (d, J = 6.8 Hz); **^{31}P NMR** (243 MHz, $CDCl_3$) δ -0.3; **IR** (thin film) ν 3401.8, 2985.9, 1717.3, 1604.5, 1493.6, 1370.2, 1350.9, 1252.5, 1183.1, 1027.9 cm^{-1}; **HRMS** (ESI^+) Calcd. for $C_{28}H_{32}NO_7P$ 526.1995 ($M+H^+$) found 526.1944; **TLC** (2:1 EtOAc/Hexanes): R_f = 0.19.

General procedure for three component reaction using chiral iminophosphorane:

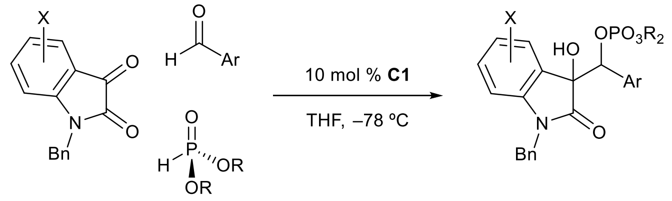

An oven-dried test tube was evacuated and filled with argon, then charged sequentially with the isatin substrate (0.1 mmol) and THF (0.5 mL), followed by the dialkylphosphite (0.11 mmol, 1.1 equiv) and the aldehyde (0.5 mmol, 5.0 equiv). THF (0.5 mL) was then added and used to wash the residual solids on the sides of the test tube to the bottom. The reaction was stirred at –78 ºC in a cryogenic cooling apparatus, then the iminophosphorane catalyst **C1** (5.81 mg, 0.01 mmol, 0.1 equiv) was added. The reaction was then stirred at –78 ºC and monitored by TLC until the reaction was complete. Trifluoroacetic acid in toluene (40 µL of an 0.5 M solution) was added to quench the reaction and the reaction was concentrated on a rotatory evaporator. The crude materials thusly obtained were purified using flash column chromatography, with a gradient from 1:1 hexane/EtOAc to 1:2 hexane/EtOAc.

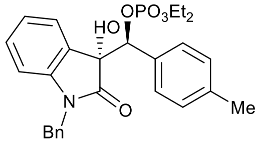

1-Benzyl-3-((((diethyl-λ^3-oxidanyl)(λ^1-oxidanyl)phosphoryl)oxy)(*p*-tolyl)methyl)-3-hydroxyindolin-2-one ((±)-4a): The title compound was prepared according to general procedure. The diastereomeric ratio was determined by ^{1}H NMR spectroscopic analysis of the crude reaction mixture by comparison of the resonances at δ 5.73 (major diastereomer) and δ 4.44 (minor diastereomer). White solid (mp 166-167 °C); **^{1}H NMR** (400 MHz, $CDCl_3$) δ 7.73 (d, *J* = 7.2 Hz, 1H), 7.18-7.07 (m, 5H), 6.94-6.89 (m, 4H), 6.56 (d, *J* = 7.6 Hz, 2H), 6.37 (d, *J* = 7.6 Hz, 1H), 5.77 (d, *J* = 8.8 Hz, 1H), 4.98 (d, *J* = 16.0 Hz, 1H), 4.38 (s, 1H), 4.28 (d, *J* = 16.0 Hz, 1H), 4.22-3.97 (m, 4H), 2.30 (s, 3H), 1.30 (t, *J* = 6.8 Hz, 3H), 1.22 (t, *J* = 6.8 Hz, 3H); **^{13}C NMR** (151 MHz, $CDCl_3$) δ 175.0, 143.3, 138.7, 134.8, 131.1 (d, *J* = 3.6 Hz), 130.1, 128.7, 128.4, 127.9, 127.2, 126.6, 126.5, 126.3, 123.0, 109.4, 83.6 (d, *J* = 5.7 Hz), 79.1 (d, *J* = 4.5 Hz), 64.5 (d, *J* = 5.7 Hz), 64.3 (d, *J* = 5.9 Hz), 43.7, 21.3, 16.1 (d, *J* = 7.2 Hz), 16.0 (d, *J* = 6.9 Hz); **^{31}P NMR** (162 MHz, $CDCl_3$) δ -0.3; IR (thin film) ν 3420.1, 2927.5, 1721.2, 1615.1, 1468.5, 1368.3, 1249.7, 1123.3, 1080.9, 1028.8, 909.3 cm^{-1}; **HRMS** (ESI$^+$) Calcd. for $C_{27}H_{30}NO_6P$ 496.1889 (M+H$^+$) found 496.1893; **HPLC** Chiralpak IA column, Hex/^{i}PrOH = 85:15, flow rate = 1.0 mL/min, λ = 210 nm, 11.3 min (minor isomer), 15.3 min (major isomer); **TLC** (2:1 EtOAc/Hexanes): R_f = 0.33.

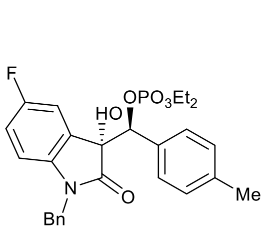

(*R*)-1-Benzyl-3-((*S*)-(((diethyl-λ^3-oxidanyl)(λ^1-oxidanyl)phosphoryl)oxy)(*p*-tolyl)methyl)-5-fluoro-3-hydroxyindolin-2-one (4h): The title compound was prepared according to general procedure. The diastereomeric ratio was determined by ^{1}H NMR spectroscopic analysis of the crude reaction mixture by comparison of the resonances at δ 5.79 (major diastereomer) and δ 5.70 (minor diastereomer). White solid; **^{1}H NMR** (400 MHz, $CDCl_3$) δ 7.50

(dd, J = 2.8, 7.8 Hz, 1H), 7.18-7.07 (m, 3H), 6.96-6.90 (m, 4H), 6.86 (dt, J = 2.7, 8.7 Hz, 1H), 6.51 (d, J = 7.3 Hz, 2H), 6.28 (dd, J = 4.1, 8.7 Hz, 1H), 5.79 (d, J = 8.7 Hz, 1H), 4.98 (d, J = 16.0 Hz, 1H), 4.28-4.09 (m, 3H), 4.00 (quintet, J = 7.3 Hz, 2H), 2.31 (s, 3H), 1.32 (dt, J = 0.9, 7.3 Hz, 3H), 1.22 (dt, J = 0.9, 7.3 Hz, 3H), OH-proton was not found probably due to broadening; **^{13}C NMR** (126 MHz, $CDCl_3$) δ 174.8, 159.2 (d, J = 243.0 Hz), 139.2_0, 139.1_9, 138.9, 134.5, 130.8, 128.8, 128.5, 127.8, 127.4, 126.6, 116.3 (d, J = 23.6 Hz), 114.4 (d, J = 25.0 Hz), 110.0 (d, J = 8.2 Hz), 83.4 (d, J = 5.5 Hz), 79.4 (d, J = 4.6 Hz), 64.6 (d, J = 5.5 Hz), 64.4 (d, J = 5.5 Hz), 43.8, 21.3, 16.1 (d, J = 7.3 Hz), 16.0 (d, J = 7.3 Hz); **^{31}P NMR** (162 MHz, $CDCl_3$) δ 0.48; **^{19}F NMR** (376 MHz, $CDCl_3$) δ -119.6; **IR** (thin film) ν 3275.1, 2980.0, 2247.1, 1714.7, 1620.2, 1485.2, 1257.6, 1176.6, 1006.8, 902.7 cm^{-1}; **HRMS** (ESI^+) Calcd. for $C_{27}H_{29}FNO_6P$ 536.1614 ($M+Na^+$) found 536.1605; **HPLC** Chiralpak IA column, Hex/^{i}PrOH/EtOH = 95:4:1, flow rate = 1.0 mL/min, λ = 210 nm, 28.5 min (minor isomer), 44.2 min (major isomer); **TLC** (1:4 EtOAc/Hexanes): R_f = 0.38.

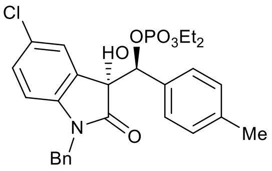

(*R*)-1-Benzyl-3-((*S*)-(((diethyl-λ^3-oxidanyl)(λ^1-oxidanyl)phosphoryl)oxy)(*p*-tolyl)methyl)-5-chloro-3-hydroxyindolin-2-one (4i): The title compound was prepared according to general procedure. The diastereomeric ratio was determined by ^{1}H NMR spectroscopic analysis of the crude reaction mixture by comparison of the resonances at δ 5.80 (major diastereomer) and δ 5.72 (minor diastereomer). White solid; **^{1}H NMR** (400 MHz, $CDCl_3$) δ 7.72 (s, 1H), 7.18-7.06 (m, 4H), 6.99-6.91 (m, 4H), 6.50 (d, J = 7.3 Hz, 2H), 6.27 (d, J = 8.2 Hz, 1H), 5.78 (d, J = 9.2 Hz, 1H), 4.98 (br, 1H), 4.97 (d, J = 16.0 Hz, 1H), 4.28-4.10 (m, 3H), 4.00 (quintet, J = 7.3 Hz, 2H), 2.32 (s, 3H), 1.33 (t, J = 7.3 Hz, 3H), 1.22 (t, J = 7.3 Hz, 3H); **^{13}C NMR** (126 MHz, $CDCl_3$) δ 174.7, 141.9, 139.0, 134.5, 130.9, 130.0, 128.9, 128.6, 128.5, 128.4, 128.0, 127.5, 126.8, 126.7, 110.5, 83.4 (d, J = 6.4 Hz), 79.4 (d, J = 4.6 Hz), 64.8 (d, J = 5.5 Hz), 64.6 (d, J = 6.4 Hz), 43.9, 21.4, 16.2 (d, J = 8.6 Hz), 16.1 (d, J = 6.4 Hz); **^{31}P NMR** (162 MHz, $CDCl_3$) δ 0.51; **IR** (thin film) ν 3269.3, 2987.7, 2245.1, 1726.3, 1612.5, 1483.3, 1253.7, 1172.7, 1006.8, 783.1 cm^{-1}; **HRMS** (ESI^+) Calcd. for $C_{27}H_{29}ClNO_6P$ 552.1319 ($M+Na^+$) found 552.1309; **HPLC** Chiralpak IA column, Hex/^{i}PrOH/EtOH = 95:4:1, flow rate = 1.0 mL/min, λ = 210 nm, 28.8 min (minor isomer), 43.3 min (major isomer); **TLC** (1:4 EtOAc/Hexanes): R_f = 0.41.

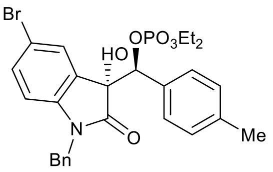

(*R*)-1-Benzyl-3-((*S*)-(((diethyl-λ^3-oxidanyl)(λ^1-oxidanyl)phosphoryl)oxy)(*p*-tolyl)methyl)-5-bromo-3-hydroxyindolin-2-one (4j): The title compound was prepared according to general procedure. The diastereomeric ratio was determined by ^{1}H NMR spectroscopic analysis of the crude reaction mixture by comparison of the resonances at δ 5.78 (major diastereomer) and δ 5.69 (minor diastereomer). White solid; **^{1}H NMR** (400 MHz, $CDCl_3$) δ 7.84 (d, J = 1.8 Hz, 1H), 7.29 (dd, J = 1.8, 8.2 Hz, 1H), 7.68-7.07 (m, 3H), 6.97-6.90 (m, 4H), 6.50 (d, J = 7.8 Hz, 2H), 6.23 (d, J = 8.7 Hz, 1H), 5.77 (d, J = 8.7 Hz, 1H), 4.96 (d, J = 16.0 Hz, 1H), 4.29-4.11 (m, 3H), 4.00 (quintet, J = 7.4 Hz, 2H), 2.32 (s, 3H), 1.35 (t, J = 6.8 Hz, 3H), 1.22 (t, J = 6.8 Hz, 3H), OH-proton was not found probably due to broadening; **^{13}C NMR** (126 MHz, $CDCl_3$) δ 174.6, 142.4, 139.1, 134.4, 133.0, 130.9, 130.8, 129.6, 129.0,

128.0, 127.5, 126.7, 115.8, 111.0, 83.4 (d, *J* = 6.4 Hz), 79.4 (d, *J* = 4.6 Hz), 64.8 (d, *J* = 5.5 Hz), 64.6 (d, *J* = 5.5 Hz), 43.9, 21.5, 16.2 (d, *J* = 7.3 Hz), 16.1 (d, *J* = 6.4 Hz), one carbon atom was not found probably due to overlapping; **^{31}P NMR** (162 MHz, $CDCl_3$) δ 0.67; **IR** (thin film) ν 3292.5, 2982.9, 2908.7, 2245.1, 1726.3, 1608.6, 1479.4, 1247.9, 1028.1, 733.0 cm^{-1}; **HRMS** (ESI^+) Calcd. for $C_{27}H_{29}BrNO_6P$ 596.0814 ($M+Na^+$) found 596.0809; **HPLC** Chiralpak IA column, Hex/^{i}PrOH = 95:5, flow rate = 1.0 mL/min, λ = 210 nm, 29.9 min (minor isomer), 43.0 min (major isomer); **TLC** (1:4 EtOAc/Hexanes): R_f = 0.44.

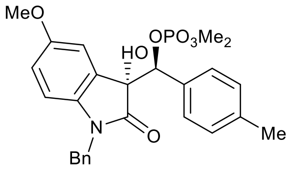

(*R*)-1-Benzyl-3-((*S*)-(((diethyl-λ^3-oxidanyl)(λ^1-oxidanyl)phosphoryl)oxy)(*p*-tolyl) methyl)-5-methoxy-3-hydroxyindolin-2-one (4l): The title compound was prepared according to general procedure. The diastereomeric ratio was determined by ^{1}H NMR spectroscopic analysis of the crude reaction mixture by comparison of the resonances at δ 5.77 (major diastereomer) and δ 5.74 (minor diastereomer). White solid; **^{1}H NMR** (400 MHz, $CDCl_3$) δ 7.36 (d, *J* = 2.3 Hz, 1H), 7.18-7.07 (m, 3H), 6.97-6.92 (m, 4H), 6.69 (dd, *J* = 8.6, 2.5 Hz, 1H), 6.54 (d, *J* = 7.3 Hz, 2H), 6.28 (d, *J* = 8.9 Hz, 1H), 5.77 (d, *J* = 8.7 Hz, 1H), 4.99 (d, *J* = 16.0 Hz, 1H), 4.40 (br, 1H), 4.25 (d, *J* = 16.0 Hz, 1H), 3.81 (s, 3H), 3.78 (d, *J* = 11.4 Hz, 3H), 3.64 (d, *J* = 11.5 Hz, 3H), 2.31 (s, 3H); **^{13}C NMR** (126 MHz, $CDCl_3$) δ 174.9, 156.2, 138.9, 136.8, 135.0, 131.2, 128.9, 128.5, 128.1, 127.7, 127.3, 126.7, 114.7, 113.7, 110.0, 83.9 (d, *J* = 5.5 Hz), 79.5 (d, *J* = 4.6 Hz), 56.0, 54.8 (d, *J* = 5.5 Hz), 54.6 (d, *J* = 6.4 Hz), 43.9, 21.4; **^{31}P NMR** (162 MHz, $CDCl_3$) δ 2.55; **IR** (thin film) ν 3300.2, 2951.1, 2243.2, 1712.8, 1604.8, 1487.1, 1257.6, 1163.1, 1026.1, 810.1 cm^{-1}; **HRMS** (ESI^+) Calcd. for $C_{26}H_{28}NO_7P$ 504.1552 ($M+Na^+$) found 520.1499; **HPLC** Chiralpak IA column, Hex/^{i}PrOH/EtOH = 90:5:5, flow rate = 1.0 mL/min, λ = 210 nm, 34.2 min (minor isomer), 45.4 min (major isomer); **TLC** (1:4 EtOAc/Hexanes): R_f = 0.17.

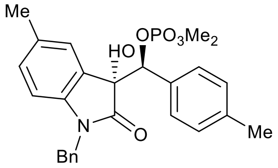

(*R*)-1-Benzyl-3-((*S*)-(((diethyl-λ^3-oxidanyl)(λ^1-oxidanyl)phosphoryl)oxy)(*p*-tolyl)methyl)-5-methyl-3-hydroxyindolin-2-one (4m): The title compound was prepared according to general procedure. The diastereomeric ratio was determined by ^{1}H NMR spectroscopic analysis of the crude reaction mixture by comparison of the resonances at δ 5.76 (major diastereomer) and δ 5.72 (minor diastereomer). White solid; **^{1}H NMR** (400 MHz, $CDCl_3$) δ 7.54 (s, 1H), 7.17-7.06 (m, 3H), 7.00-6.91 (m, 5H), 6.54 (d, *J* = 7.3 Hz, 2H), 6.27 (d, *J* = 7.8 Hz, 1H), 5.75 (d, *J* = 8.7 Hz, 1H), 5.00 (br, 1H), 4.26 (d, *J* = 16.0 Hz, 1H), 3.74 (d, *J* = 11.0 Hz, 3H), 3.66 (d, *J* = 11.4 Hz, 3H), 2.36 (s, 3H), 2.31 (s, 3H); **^{13}C NMR** (126 MHz, $CDCl_3$) δ 175.1, 141.0, 138.9, 135.0, 132.7, 131.2 (d, *J* = 3.6 Hz), 130.5, 128.9, 128.5, 128.1, 127.3, 127.1, 126.7, 126.4, 109.3, 84.0 (d, *J* = 6.4 Hz), 79.2 (d, *J* = 5.5 Hz), 54.8 (d, *J* = 5.5 Hz), 54.6 (d, *J* = 5.5 Hz), 43.8, 21.4, 21.3; **^{31}P NMR** (162 MHz, $CDCl_3$) δ 2.55; **IR** (thin film) ν 3336.9, 2924.1, 2850.8, 2243.2, 2115.9, 1714.7, 1620.2, 1492.9, 1259.5, 1031.9 cm^{-1}; **HRMS** (ESI^+) Calcd. for $C_{26}H_{28}NO_6P$ 504.1552 ($M+Na^+$) found 504.1547; **HPLC** Chiralpak IA column, Hex/^{i}PrOH = 85:15, flow rate = 1.0 mL/min, λ = 210 nm, 13.4 min (minor isomer), 16.5 min (major isomer); **TLC** (1:4 EtOAc/Hexanes): R_f = 0.23.

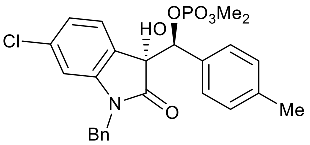

(*R*)-1-Benzyl-3-((*S*)-(((diethyl-λ³-oxidanyl)(λ¹-oxidanyl)phosphoryl)oxy)(*p*-tolyl)methyl)-6-chloro-3-hydroxyindolin-2-one (4n): The title compound was prepared according to general procedure. The diastereomeric ratio was determined by ^{1}H NMR spectroscopic analysis of the crude reaction mixture by comparison of the resonances at δ 5.80 (major diastereomer) and δ 5.74 (minor diastereomer). White solid; **^{1}H NMR** (400 MHz, $CDCl_3$) δ 7.66 (d, *J* = 7.8 Hz, 1H), 7.20-7.16 (m, 1H), 7.12-7.08 (m, 3H), 6.97 (d, *J* = 7.8 Hz, 2H), 6.90 (d, *J* = 8.5 Hz, 2H), 6.53 (d, *J* = 7.3 Hz, 2H), 6.38 (d, *J* = 1.8 Hz, 1H), 5.77 (d, *J* = 9.2 Hz, 1H), 4.98 (d, *J* = 16.0 Hz, 1H), 4.60 (br, 1H), 4.24 (d, *J* = 16.5 Hz, 1H), 3.76 (d, *J* = 11.4 Hz, 3H), 3.65 (d, *J* = 11.4 Hz, 3H), 2.32 (s, 3H); **^{13}C NMR** (126 MHz, $CDCl_3$) δ 175.1, 144.7, 139.2, 136.1, 134.3, 130.8 (d, *J* = 3.6 Hz), 129.1, 128.7, 127.9, 127.6, 127.5, 126.7, 125.0, 123.1, 110.2, 83.8 (d, *J* = 6.4 Hz), 78.9 (d, *J* = 4.5 Hz), 54.9 (d, *J* = 5.5 Hz), 54.7 (d, *J* = 6.4 Hz), 43.9, 21.5; **^{31}P NMR** (162 MHz, $CDCl_3$) δ 2.55; **IR** (thin film) ν 3302.1, 2956.9, 2247.1, 1726.3, 1608.6, 1454.3, 1251.8, 1180.4, 1025.1, 731.0 cm^{-1}; **HRMS** (ESI^+) Calcd. for $C_{25}H_{25}ClNO_6P$ 524.1006 ($M+Na^+$) found 524.1003; **HPLC** Chiralpak IA column, Hex/^{i}PrOH = 90.9:9.1, flow rate = 1.0 mL/min, λ = 210 nm, 20.5 min (minor isomer), 30.6 min (major isomer); **TLC** (1:4 EtOAc/Hexanes): R_f = 0.41.

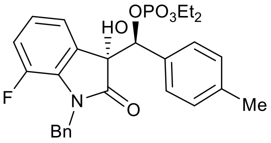

(*R*)-1-Benzyl-3-((*S*)-(((diethyl-λ³-oxidanyl)(λ¹-oxidanyl)phosphoryl)oxy)(*p*-tolyl)methyl)-7-fluoro-3-hydroxyindolin-2-one (4o): The title compound was prepared according to general procedure. The diastereomeric ratio was determined by ^{1}H NMR spectroscopic analysis of the crude reaction mixture by comparison of the resonances at δ 5.75 (major diastereomer) and δ 5.67 (minor diastereomer). White solid; **^{1}H NMR** (400 MHz, $CDCl_3$) δ 7.54 (d, *J* = 7.3 Hz, 1H), 7.18-7.04 (m, 5H), 6.99-6.86 (m, 4H), 6.70 (d, *J* = 7.3 Hz, 2H), 5.74 (d, *J* = 8.7 Hz, 1H), 4.91 (d, *J* = 16.0 Hz, 1H), 4.59 (d, *J* = 16.0 Hz, 1H), 4.21-4.15 (m, 2H), 4.00 (quintet, *J* = 6.9 Hz, 2H), 2.28 (s, 3H), 1.29 (dt, *J* = 0.9, 7.3 Hz, 3H), 1.21 (dt, *J* = 0.9, 7.3 Hz, 3H), OH-proton was not found probably due to broadening; **^{13}C NMR** (126 MHz, $CDCl_3$) δ 174.7, 147.3 (d, *J* = 244.8 Hz) 139.0, 136.2, 130.7 (d, *J* = 3.6 Hz), 129.6 (d, *J* = 2.7 Hz), 128.9, 128.4, 127.9, 127.2, 126.8 (d, *J* = 1.8 Hz), 123.9 (d, *J* = 6.4 Hz), 122.4 (d, *J* = 3.6 Hz), 118.4 (d, *J* = 20.0 Hz), 83.4 (d, *J* = 5.5 Hz), 79.12 (d, *J* = 5.5 Hz), 79.10 (d, *J* = 4.6 Hz), 64.6 (d, *J* = 5.5 Hz), 64.5 (d, *J* = 5.5 Hz), 45.3 (d, *J* = 4.5 Hz), 21.4, 16.2 (d, *J* = 7.3 Hz), 16.1 (d, *J* = 7.3 Hz); **^{31}P NMR** (162 MHz, $CDCl_3$) δ 0.46; ^{19}F NMR (376 MHz, $CDCl_3$) δ -133.6; **IR** (thin film) ν 3296.4, 2985.8, 2237.4, 1730.2, 1631.8, 1487.1, 1247.9, 1022.3, 910.4, 733.0 cm^{-1}; **HRMS** (ESI^+) Calcd. for $C_{27}H_{29}FNO_6P$ 536.1614 ($M+Na^+$) found 536.1608; **HPLC** Chiralpak IA column, Hex/^{i}PrOH/EtOH = 85:5:10, flow rate = 1.0 mL/min, λ = 210 nm, 9.3 min (minor isomer), 12.5 min (major isomer); **TLC** (1:4 EtOAc/Hexanes): R_f = 0.38.

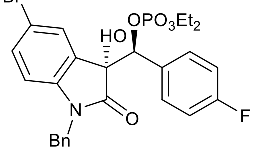

(*R*)-1-Benzyl-5-bromo-3-((*S*)-(((diethyλ³-oxidanyl)(λ¹-oxidanyl)phosphoryl)oxy)(4-fluorophenyl)methyl)-3-hydroxyindolin-2-one (4p): The title compound was prepared according to general procedure. The diastereomeric ratio was determined by ^{1}H NMR

spectroscopic analysis of the crude reaction mixture by comparison of the resonances at δ 5.81 (major diastereomer) and δ 5.75 (minor diastereomer). White solid; ^{1}**H NMR** (400 MHz, $CDCl_3$) δ 7.83 (d, *J* = 1.8 Hz, 1H), 7.31 (dd, *J* = 2.3, 8.7 Hz, 1H), 7.21-7.12 (m, 3H), 7.03-6.99 (m, 2H), 6.86-6.81 (m, 2H), 6.54 (d, *J* = 7.4 Hz, 2H), 6.29 (d, *J* = 8.7 Hz, 1H), 5.79 (d, *J* = 9.2 Hz, 1H), 4.91 (d, *J* = 16.0 Hz, 1H), 4.89 (br-s, 1H), 4.28 (d, *J* = 16.5 Hz, 1H), 4.25-4.13 (m, 2H), 4.02 (quintet, *J* = 7.3 Hz, 2H), 1.37 (t, *J* = 6.9 Hz, 3H), 1.23 (t, *J* = 7.3 Hz, 3H); ^{13}**C NMR** (126 MHz, $CDCl_3$) δ 174.5, 163.2 (d, *J* = 249.4 Hz), 142.4, 134.3, 133.2, 130.0 (d, *J* = 8.2 Hz), 129.6, 128.8, 128.4, 127.8, 127.0, 126.6, 115.9, 115.3 (d, *J* = 21.8 Hz), 111.1, 82.7 (d, *J* = 6.4 Hz), 79.3 (d, *J* = 4.6 Hz), 64.9 (d, *J* = 5.5 Hz), 64.6 (d, *J* = 5.5 Hz), 43.9, 16.3 (d, *J* = 7.3 Hz), 16.1 (d, *J* = 6.3 Hz); ^{31}**P NMR** (162 MHz, $CDCl_3$) δ 0.27; ^{19}**F NMR** (376 MHz, $CDCl_3$) δ -111.6; **IR** (thin film) ν 3252.0, 2985.8, 2245.1, 1726.3, 1606.7, 1510.3, 1479.4, 1346.3, 1228.7, 1022.3 cm^{-1}; **HRMS** (ESI^+) Calcd. for $C_{26}H_{26}BrFNO_6P$ 600.0563 ($M+Na^+$) found 600.0552; **HPLC** Chiralpak IA column, Hex/^{i}PrOH/EtOH = 95:4:1, flow rate = 1.0 mL/min, λ = 262 nm, 29.6 min (minor isomer), 43.8 min (major isomer); **TLC** (1:4 EtOAc/Hexanes): R_f = 0.45.

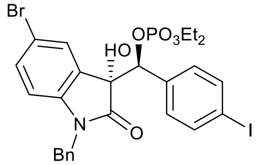

(*R*)-1-Benzyl-5-bromo-3-((*S*)-(((diethyl-λ^3-oxidanyl)(λ^1-oxidanyl)phosphoryl)oxy)(4-iodophenyl)methyl)-3-hydroxyindolin-2-one (4q): The title compound was prepared according to general procedure. The diastereomeric ratio was determined by ^{1}H NMR spectroscopic analysis of the crude reaction mixture by comparison of the resonances at δ 5.76 (major diastereomer) and δ 5.69 (minor diastereomer). White solid**;** ^{1}**H NMR** (400 MHz, $CDCl_3$) δ 7.83 (d, *J* = 1.8 Hz, 1H), 7.48 (d, *J* = 8.2 Hz, 2H), 7.30 (dd, *J* = 1.8, 6.4 Hz, 1H), 7.25-7.23 (m, 3H), 6.78 (d, *J* = 8.7 Hz, 2H), 6.56-6.54 (m, 2H), 6.28 (d, *J* = 8.7 Hz, 1H), 5.75 (d, *J* = 8.7 Hz, 1H), 4.99 (d, *J* = 16.0 Hz, 1H), 4.98 (br-s, 1H), 4.28-4.15 (m, 3H), 4.03 (quintet, *J* = 8.2 Hz, 2H), 1.36 (dt, *J* = 0.9, 6.9 Hz, 3H), 1.24 (dt, *J* = 0.9, 6.9 Hz, 3H); ^{13}**C NMR** (126 MHz, $CDCl_3$) δ 174.3, 142.4, 137.5, 134.2, 133.6, 133.2, 129.8, 129.6, 129.0, 128.3, 127.8, 126.6, 115.9, 111.2, 95.5, 82.8 (d, *J* = 5.5 Hz), 79.1 (d, *J* = 5.3 Hz), 65.0 (d, *J* = 6.4 Hz), 64.7 (d, *J* = 4.6 Hz), 44.0, 16.3 (d, *J* = 6.4 Hz), 16.1 (d, *J* = 6.4 Hz); ^{31}**P NMR** (162 MHz, $CDCl_3$) δ 0.56; **IR** (thin film) ν 3290.6, 2980.0, 2243.2, 1730.2, 1606.7, 1481.3, 1371.4, 1253.7, 1024.2, 910.4 cm^{-1}; **HRMS** (ESI^+) Calcd. for $C_{26}H_{26}BrINO_6P$ 707.9623 ($M+Na^+$) found 707.9618; **HPLC** Chiralpak AD3 column, Hex/EtOH = 88:12 (4 °C), flow rate = 1.0 mL/min, λ = 210 nm, 18.9 min (minor isomer), 29.7 min (major isomer); **TLC** (1:4 EtOAc/Hexanes): R_f = 0.47.

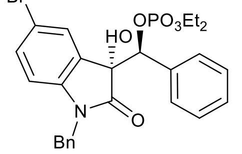

(*R*)-1-Benzyl-5-bromo-3-((*S*)-(((diethyl-λ^3-oxidanyl)(λ^1-oxidanyl)phosphoryl)oxy)(phenyl)methyl)-3-hydroxyindolin-2-one (4r): The title compound was prepared according to general procedure. The diastereomeric ratio was determined by ^{1}H NMR spectroscopic analysis of the crude reaction mixture by comparison of the resonances at δ 5.82 (major diastereomer) and δ 5.77 (minor diastereomer). White solid; ^{1}**H NMR** (400 MHz, $CDCl_3$) δ 7.86 (s, 1H), 7.32-7.26 (m, 3H), 7.18-7.14 (m, 3H), 7.11-7.04 (m, 4H), 6.46 (d, *J* = 7.8 Hz, 2H), 6.21 (d, *J* = 8.2 Hz, 1H), 5.81 (d, *J* = 9.2 Hz, 1H), 5.06 (br, 1H), 4.90 (d, *J* = 16.5 Hz, 1H), 4.29-4.11 (m, 3H), 4.01 (quintet, *J* = 7.8 Hz, 2H),

1.34 (t, J = 7.1 Hz, 3H), 1.21 (t, J = 6.8 Hz, 3H); **^{13}C NMR** (126 MHz, $CDCl_3$) δ 174.5, 142.4, 134.4, 133.9, 133.0, 129.6, 129.2, 128.8, 128.7 (d, J = 2.7 Hz), 128.3, 128.1, 127.5, 126.6, 115.8, 111.1, 83.5 (d, J = 6.4 Hz), 79.4 (d, J = 4.6 Hz), 64.9 (d, J = 5.5 Hz), 65.6 (d, J = 5.5 Hz), 43.9, 16.3 (d, J = 7.3 Hz), 16.1 (d, J = 6.4 Hz); **^{31}P NMR** (162 MHz, $CDCl_3$) δ 0.48; **IR** (thin film) ν 3282.8, 2978.1, 2247.1, 1726.3, 1714.7, 1606.7, 1454.3, 1348.2, 1253.7, 725.2 cm^{-1}; **HRMS** (ESI$^+$) Calcd. for $C_{26}H_{27}BrNO_6P$ 582.0657 (M+Na$^+$) found 582.0652; **HPLC** Chiralpak IA column, Hex/^{i}PrOH = 85:15, flow rate = 1.0 mL/min, λ = 210 nm, 18.1 min (minor isomer), 28.0 min (major isomer); **TLC** (1:4 EtOAc/Hexanes): R_f = 0.44.

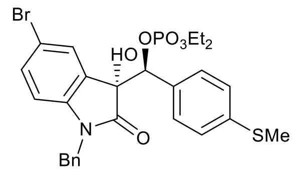

(*R*)-1-Benzyl-5-bromo-3-((*S*)-(((diethyl-λ^3-oxidanyl)(λ^1-oxidanyl)phosphoryl)oxy)(4-(methylthio)phenyl)methyl)-3-hydroxyindolin-2-one (4s): The title compound was prepared according to general procedure. The diastereomeric ratio was determined by ^{1}H NMR spectroscopic analysis of the crude reaction mixture by comparison of the resonances at δ 5.78 (major diastereomer) and δ 5.71 (minor diastereomer). White solid; **^{1}H NMR** (400 MHz, $CDCl_3$) δ 7.85 (d, J = 1.8 Hz, 1H), 7.29 (dd, J = 2.3, 8.7 Hz, 1H), 7.18-7.17 (m, 3H), 7.03-6.93 (m, 4H), 6.47-6.45 (m, 2H), 6.24 (d, J = 8.2 Hz, 1H), 5.77 (d, J = 9.2 Hz, 1H), 5.01 (d, J = 16.0 Hz, 1H), 4.30-4.13 (m, 3H), 4.02 (quintet, J = 7.3 Hz, 2H), 2.42 (s, 3H), 1.36 (t, J = 6.9 Hz, 3H), 1.23 (t, J = 7.3 Hz, 3H), OH-proton was not found probably due to broadening; **^{13}C NMR** (126 MHz, $CDCl_3$) δ 174.5, 142.4, 140.0, 134.2, 133.1, 130.2, 129.6, 128.9, 128.8, 128.6, 128.5, 127.7, 126.5, 125.4, 115.8, 111.1, 83.2 (d, J = 5.5 Hz), 79.3 (d, J = 4.6 Hz), 64.9 (d, J = 5.5 Hz), 64.6 (d, J = 5.5 Hz), 43.9, 16.3 (d, J = 7.3 Hz), 16.1 (d, J = 6.4 Hz); **^{31}P NMR** (162 MHz, $CDCl_3$) δ 0.54; **IR** (thin film) ν 3306.0, 2985.8, 2247.1, 1730.2, 1606.7, 1483.3, 1249.9, 1134.1, 1020.3, 731.0 cm^{-1}; **HRMS** (ESI$^+$) Calcd. for $C_{27}H_{29}BrNO_6PS$ 628.0534 (M+Na$^+$) found 628.0526; **HPLC** Chiralpak IA column, Hex/^{i}PrOH = 85:15, flow rate = 1.0 mL/min, λ = 210 nm, 13.7 min (minor isomer), 20.7 min (major isomer); **TLC** (1:4 EtOAc/Hexanes): R_f = 0.42.

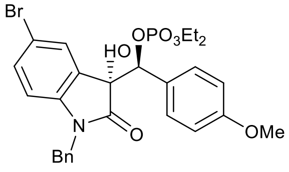

(*R*)-1-Benzyl-5-bromo-3-((*S*)-(((diethyl-λ^3-oxidanyl)(λ^1-oxidanyl)phosphoryl)oxy)(4-methoxyphenyl)methyl)-3-hydroxyindolin-2-one (4t): The title compound was prepared according to general procedure. The diastereomeric ratio was determined by ^{1}H NMR spectroscopic analysis of the crude reaction mixture by comparison of the resonances at δ 5.78 (major diastereomer) and δ 5.71 (minor diastereomer). White solid; **^{1}H NMR** (400 MHz, $CDCl_3$) δ 7.84 (d, J = 1.8 Hz, 1H), 7.29 (dd, J = 1.8, 8.2 Hz, 1H), 7.18-7.07 (m, 3H), 6.94 (d, J = 9.2 Hz, 2H), 6.67 (d, J = 8.7 Hz, 2H), 6.47 (d, J = 7.8 Hz, 2H), 6.24 (d, J = 8.7 Hz, 1H), 5.76 (d, J = 9.2 Hz, 1H), 4.98 (d, J = 16.5 Hz, 1H), 4.88 (br, 1H), 4.28-4.14 (m, 3H), 4.02 (quintet, J = 7.3 Hz, 2H), 3.76 (s, 3H), 1.36 (dt, J = 0.9, 6.9 Hz, 3H), 1.23 (dt, J = 0.9, 6.9 Hz, 3H); **^{13}C NMR** (126 MHz, $CDCl_3$) δ 174.5, 160.3, 142.5, 134.3, 133.0, 129.5, 129.4, 128.7, 128.6, 127.6, 126.6, 125.9 (d, J = 4.5 Hz), 115.8, 113.7, 111.0, 83.2 (d, J = 5.5 Hz), 79.4 (d, J = 4.6 Hz), 64.8 (d, J = 5.5 Hz), 64.6 (d, J = 5.5 Hz), 55.2, 43.8, 16.3 (d, J = 7.3 Hz), 16.1 (d, J = 6.4 Hz); **^{31}P NMR** (162 MHz, $CDCl_3$) δ 0.83; **IR** (thin film) ν 3313.7, 2976.2, 2353.2, 2245.1, 1730.2, 1612.5, 1514.1, 1485.2,

1249.9, 1024.2 cm^{-1}; **HRMS** (ESI$^+$) Calcd. for $C_{27}H_{29}BrNO_7P$ 612.0763 (M+Na$^+$) found 612.0754; **HPLC** Chiralpak IA column, Hex/^{i}PrOH = 85:15, flow rate = 1.0 mL/min, λ = 210 nm, 13.4 min (minor isomer), 18.9 min (major isomer); **TLC** (1:4 EtOAc/Hexanes): R_f = 0.39.

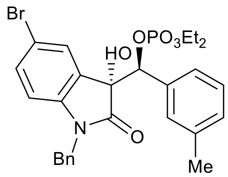

(*R*)-1-Benzyl-5-bromo-3-((*S*)-(((diethyl-λ^3-oxidanyl)(λ^1-oxidanyl)phosphoryl)oxy)(*m*-tolyl)methyl)-3-hydroxyindolin-2-one (4u): The title compound was prepared according to general procedure. The diastereomeric ratio was determined by ^{1}H NMR spectroscopic analysis of the crude reaction mixture by comparison of the resonances at δ 5.76 (major diastereomer) and δ 5.71 (minor diastereomer). White solid; **^{1}H NMR** (400 MHz, $CDCl_3$) δ 7.83 (d, *J* = 1.8 Hz, 1H), 7.28 (dd, *J* = 1.8, 8.2 Hz, 1H), 7.18-7.02 (m, 5H), 6.86 (s, 1H), 6.80 (d, *J* = 7.8 Hz, 1H), 6.45 (d, *J* = 7.3 Hz, 2H), 6.21 (d, *J* = 8.0 Hz, 1H), 5.75 (d, *J* = 8.7 Hz, 1H), 4.94 (d, *J* = 16.5 Hz, 1H), 4.86 (br, 1H), 4.30-4.12 (m, 3H), 4.02 (quintet, *J* = 7.4 Hz, 2H), 2.16 (s, 3H), 1.36 (t, *J* = 7.3 Hz, 3H), 1.24 (t, *J* = 7.1 Hz, 3H); **^{13}C NMR** (126 MHz, $CDCl_3$) δ 174.5, 142.5, 138.0, 134.5, 133.7, 133.0, 130.0, 129.6, 128.8, 128.2, 127.6, 126.5, 125.0, 115.8, 111.0, 83.5 (d, *J* = 5.5 Hz), 79.4 (d, *J* = 3.6 Hz), 64.9 (d, *J* = 6.4 Hz), 64.6 (d, *J* = 8.2 Hz), 43.9, 21.4, 16.3 (d, *J* = 7.3 Hz), 16.1 (d, *J* = 6.4 Hz), two carbon atoms were not found probably due to overlapping; **^{31}P NMR** (162 MHz, $CDCl_3$) δ 0.67; **IR** (thin film) ν 3265.5, 2932.0, 2933.7, 1730.2, 1714.2, 1608.6, 1454.3, 1249.9, 1130.3, 1020.3 cm^{-1}; **HRMS** (ESI$^+$) Calcd. for $C_{27}H_{29}BrNO_6P$ 596.0814 (M+Na$^+$) found 596.0795; **HPLC** Chiralpak IA column, Hex/^{i}PrOH = 90.9:9.1, flow rate = 1.0 mL/min, λ = 210 nm, 14.5 min (minor isomer), 24.7 min (major isomer); **TLC** (1:4 EtOAc/Hexanes): R_f = 0.45.

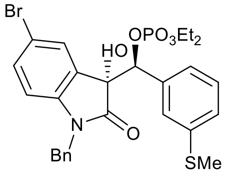

(*R*)-1-Benzyl-5-bromo-3-((*S*)-(((diethyl-λ^3-oxidanyl)(λ^1-oxidanyl)phosphoryl)oxy)(3-(methylthio)phenyl)methyl)-3-hydroxyindolin-2-one (4v): The title compound was prepared according to general procedure. The diastereomeric ratio was determined by ^{1}H NMR spectroscopic analysis of the crude reaction mixture by comparison of the resonances at δ 5.78 (major diastereomer) and δ 5.72 (minor diastereomer). White solid; **^{1}H NMR** (400 MHz, $CDCl_3$) δ 7.87 (s, 1H), 7.29 (dd, *J* = 2.3, 8.7 Hz, 1H), 7.19-7.11 (m, 4H), 7.05 (t, *J* = 7.8 Hz, 1H), 6.85 (d, *J* = 7.8 Hz, 1H), 6.81 (s, 1H), 6.49 (d, *J* = 7.3 Hz, 2H), 6.24 (d, *J* = 8.7 Hz, 1H), 5.78 (d, *J* = 8.7 Hz, 1H), 5.09 (br, 1H), 4.93 (d, *J* = 16.5 Hz, 1H), 4.31-4.13 (m, 3H), 4.03 (quintet, *J* = 6.9 Hz, 2H), 2.14 (s, 3H), 1.36 (t, *J* = 7.3 Hz, 3H), 1.23 (t, *J* = 6.8 Hz, 3H); **^{13}C NMR** (126 MHz, $CDCl_3$) δ 174.4, 142.4, 139.1, 134.7, 134.3, 133.0, 129.6, 128.6, 128.5, 127.9, 127.6, 126.5, 125.0, 124.9, 115.8, 111.2, 83.1 (d, *J* = 5.5 Hz), 79.3 (d, *J* = 4.6 Hz), 64.9 (d, *J* = 6.4 Hz), 64.7 (d, *J* = 6.4 Hz), 43.9, 16.3 (d, *J* = 6.4 Hz), 16.1 (d, *J* = 6.4 Hz), 15.6, one carbon atom was not found probably due to overlapping; **^{31}P NMR** (162 MHz, $CDCl_3$) δ 0.48; **IR** (thin film) ν 3255.8, 2983.9, 2362.8, 1730.2, 1606.7, 1479.4, 1255.7, 1018.4, 972.1, 895.0 cm^{-1}; **HRMS** (ESI$^+$) Calcd. for $C_{27}H_{29}BrNO_6PS$ 628.0534 (M+Na$^+$) found 628.0530; **HPLC** Chiralpak IA column, Hex/^{i}PrOH = 90.9:9.1, flow rate = 1.0 mL/min, λ = 210 nm, 16.4 min (minor isomer), 34.1 min (major isomer); **TLC** (1:4 EtOAc/Hexanes): R_f = 0.41.

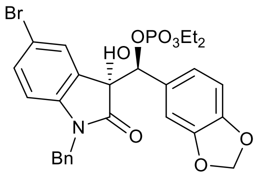

(*R*)-3-((*S*)-Benzo[d][1,3]dioxol-5-yl(((diethyl-λ^3-oxidanyl)(λ^1-oxidanyl)phosphoryl)oxy)methyl)-1-benzyl-5-bromo-3-hydroxyindolin-2-one (4w): The title compound was prepared according to general procedure. The diastereomeric ratio was determined by ^{1}H NMR spectroscopic analysis of the crude reaction mixture by comparison of the resonances at δ 5.72 (major diastereomer) and δ 5.64 (minor diastereomer). White solid; **^{1}H NMR** (400 MHz, CDCl$_3$) δ 7.80 (d, *J* = 1.8 Hz, 1H), 7.31 (dd, *J* = 0.9, 8.2 Hz, 1H), 7.19-7.14 (m, 3H), 6.64-6.59 (m, 4H), 6.40 (s, 1H), 6.32 (d, *J* = 8.7 Hz, 1H), 5.88 (dd, *J* = 1.4, 5.0 Hz, 2H), 5.71 (d, *J* = 8.7 Hz, 1H), 5.01 (d, *J* = 16.0 Hz, 1H), 4.99 (br, 1H), 4.31-4.13 (m, 3H), 4.03 (quintet, *J* = 7.3 Hz, 2H), 1.38 (dt, *J* = 0.9, 6.8 Hz, 3H), 1.25 (dt, *J* = 0.9, 7.3 Hz, 3H); **^{13}C NMR** (126 MHz, CDCl$_3$) δ 174.5, 148.4 (d, *J* = 1.8 Hz), 147.5, 142.5, 134.6, 133.1, 129.5, 128.9, 128.7, 127.7, 127.0, 126.8, 122.4, 115.9, 111.0, 108.2, 108.1, 101.3, 83.3 (d, *J* = 5.5 Hz), 79.4 (d, *J* = 4.6 Hz), 64.9 (d, *J* = 5.5 Hz), 64.7 (d, *J* = 5.5 Hz), 43.9, 16.3 (d, *J* = 7.3 Hz), 16.1 (d, *J* = 7.3 Hz); **^{31}P NMR** (162 MHz, CDCl$_3$) δ 0.56; **IR** (thin film) ν 3280.9, 2989.7, 2249.0, 1726.3, 1608.6, 1483.3, 1444.7, 1244.1, 1018.4, 733.0 cm^{-1}; **HRMS** (ESI$^+$) Calcd. for $C_{27}H_{27}BrNO_8P$ 626.0555 (M+Na$^+$) found 626.0550; **HPLC** Chiralpak IA column, Hex/^{i}PrOH = 90.9:9.1, flow rate = 1.0 mL/min, λ = 210 nm, 26.2min (minor isomer), 43.4 min (major isomer); **TLC** (1:4 EtOAc/Hexanes): R_f = 0.39.

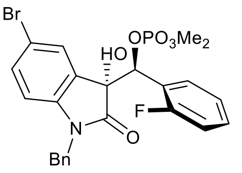

(*R*)-1-Benzyl-5-bromo-3-((*S*)-(((dimethyl-λ^3-oxidanyl)(λ^1-oxidanyl)phosphoryl)oxy)(2-fluorophenyl)methyl)-3-hydroxyindolin-2-one (4x): The title compound was prepared according to general procedure. The diastereomeric ratio was determined by ^{1}H NMR spectroscopic analysis of the crude reaction mixture by comparison of the resonances at δ 6.21 (major diastereomer) and δ 6.16 (minor diastereomer). White solid; **^{1}H NMR** (400 MHz, CDCl$_3$) δ 7.71 (s, 1H), 7.35-7.29 (m, 2H), 7.20-7.11 (m, 3H), 7.00 (t, *J* = 9.2 Hz, 1H), 6.96-6.90 (m, 2H), 6.65 (d, *J* = 7.3 Hz, 2H), 6.35 (d, *J* = 8.2 Hz, 1H), 6.14 (d, *J* = 9.2 Hz, 1H), 5.01 (d, *J* = 16.5 Hz, 1H), 4.35 (br, 1H), 4.33 (d, *J* = 16.0 Hz, 1H), 3.75 (d, *J* = 11.4 Hz, 3H), 3.68 (d, *J* = 11.4 Hz, 3H); **^{13}C NMR** (126 MHz, CDCl$_3$) δ 174.1, 160.0 (d, *J* = 252.1 Hz), 142.5, 134.6, 133.4, 131.2 (d, *J* = 8.3 Hz), 129.6, 128.9, 127.7, 126.7, 124.1 (d, *J* = 3.6 Hz), 121.5 (d, *J* = 3.6 Hz), 121.4 (d, *J* = 3.6 Hz), 115.9, 115.8, 111.1, 78.5 (d, *J* = 5.5 Hz), 75.9$_9$ (d, *J* = 5.5 Hz), 75.5$_6$ (d, *J* = 5.5 Hz), 55.0 (d, *J* = 6.4 Hz), 54.9 (d, *J* = 6.4 Hz), 44.0; **^{31}P NMR** (162 MHz, CDCl$_3$) δ 2.39; **^{19}F NMR** (376 MHz, CDCl$_3$) δ -114.0; **IR** (thin film) ν 3273.2, 2355.1, 1730.2, 1608.6, 1483.3, 1454.3, 1344.4, 1263.4, 1180.4, 1028.1 cm^{-1}; **HRMS** (ESI$^+$) Calcd. for $C_{24}H_{22}BrFNO_6P$ 572.0250 (M+Na$^+$) found 572.0245; **HPLC** Chiralpak IA column, Hex/^{i}PrOH/EtOH = 93:5:2 (4 ºC), flow rate = 1.0 mL/min, λ = 210 nm, 76.3 min (minor isomer), 92.1 min (major isomer); **TLC** (1:4 EtOAc/Hexanes): R_f = 0.42.

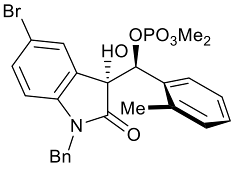

(*R*)-1-Benzyl-5-bromo-3-((*S*)-(((dimethyl-λ^3-oxidanyl)(λ^1-oxidanyl)phosphoryl)oxy)(*o*-tolyl)methyl)-3-hydroxyindolin-2-one (4y): The title compound was prepared according to general procedure. The diastereomeric ratio was determined by ^{1}H NMR spectroscopic

analysis of the crude reaction mixture by comparison of the resonances at δ 6.15 (major diastereomer) and δ 6.04 (minor diastereomer). White solid; **^{1}H NMR** (400 MHz, $CDCl_3$) δ 7.81 (s, 1H), 7.33 (d, *J* = 8.7 Hz, 1H), 7.23-7.06 (m, 5H), 6.89 (t, *J* = 7.8 Hz, 1H), 6.69 (d, *J* = 8.2 Hz, 1H), 6.42 (d, *J* = 6.9 Hz, 2H), 6.29 (dd, *J* = 1.4, 8.7 Hz, 1H), 6.14 (d, *J* = 8.7 Hz, 1H), 5.05 (d, *J* = 16.0 Hz, 1H), 4.18 (d, *J* = 16.5 Hz, 1H), 3.74 (dd, *J* = 1.8, 11.5 Hz, 3H), 3.57 (dd, *J* = 1.4, 11.4 Hz, 3H), 2.44 (s, 3H), OH-proton was not found probably due to broadening; **^{13}C NMR** (126 MHz, $CDCl_3$) δ 174.7, 142.6, 137.6, 134.4, 133.2, 132.4 (d, *J* = 2.7 Hz), 130.8, 129.8, 129.3, 128.9, 128.7, 127.7, 127.5, 126.4, 125.9, 116.0, 111.0, 79.2 (d, *J* = 6.4 Hz), 79.0 (d, *J* = 6.4 Hz), 54.8 (d, *J* = 5.5 Hz), 54.5 (d, *J* = 5.5 Hz), 43.9, 19.8; **^{31}P NMR** (162 MHz, $CDCl_3$) δ 2.60; **IR** (thin film) ν 3203.6, 2956.9, 2245.1, 1725.3, 1606.7, 1454.3, 1346.3, 1222.9, 1003.0, 812.0 cm^{-1}; **HRMS** (ESI^+) Calcd. for $C_{25}H_{25}BrNO_6P$ 596.0501 ($M+Na^+$) found 596.0495; **HPLC** Chiralpak IA column, Hex/^{i}PrOH = 90.9:9.1, flow rate = 1.0 mL/min, λ = 210 nm, 15.9 min (minor isomer), 21.6 min (major isomer); **TLC** (1:4 EtOAc/Hexanes): R_f = 0.40.

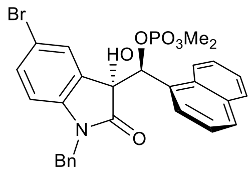

(*R*)-1-Benzyl-5-bromo-3-((*S*)-(((dimethyl-λ^3-oxidanyl)(λ^1-oxidanyl)phosphoryl)oxy)(naphthalen-1-yl)methyl)-3-hydroxyindolin-2-one (4z): The title compound was prepared according to general procedure. The diastereomeric ratio was determined by ^{1}H NMR spectroscopic analysis of the crude reaction mixture by comparison of the resonances at δ 6.41 (minor diastereomer) and δ 6.17 (major diastereomer). White solid; **^{1}H NMR** (400 MHz, $CDCl_3$) δ 8.29 (d, *J* = 8.7 Hz, 1H), 7.90 (d, *J* = 2.3 Hz, 1H), 7.82 (dd, *J* = 5.0, 8.2 Hz, 2H), 7.59-7.51 (m, 1H), 7.49-7.46 (m, 1H), 7.30 (dd, *J* = 1.4, 8.7 Hz, 1H), 7.17 (t, *J* = 7.8 Hz, 1H), 7.09-7.05 (m, 2H), 6.96-6.93 (m, 2H), 6.76 (d, *J* = 8.7 Hz, 1H), 6.22 (d, *J* = 8.2 Hz, 1H), 6.12, (m, 2H), 4.81 (d, *J* = 16.0 Hz, 1H), 4.65 (br, 1H), 4.01 (d, *J* = 16.0 Hz, 1H), 3.75 (dd, *J* = 1.8, 9.6 Hz, 3H), 3.57 (d, *J* = 11.4 Hz, 3H); **^{13}C NMR** (126 MHz, $CDCl_3$) δ 174.3, 142.7, 134.3, 133.5, 133.2, 130.8, 130.0, 129.7, 129.6, 129.0, 128.8, 128.7, 128.6, 127.5, 127.0, 126.6, 126.4, 126.2, 124.7, 123.5, 115.9, 110.9, 79.4 (d, *J* = 4.5 Hz), 55.0 (d, *J* = 5.5 Hz), 54.7 (d, *J* = 6.4 Hz), 43.9; **^{31}P NMR** (162 MHz, $CDCl_3$) δ 2.84; **IR** (thin film) ν 3277.1, 2955.0, 2247.1, 1730.2, 1608.6, 1481.3, 1259.5, 1174.7, 1026.1, 731.0 cm^{-1}; **HRMS** (ESI^+) Calcd. for $C_{28}H_{25}BrNO_6P$ 604.0501 ($M+Na^+$) found 604.0496; **HPLC** Chiralpak IA column, Hex/^{i}PrOH = 85:15, flow rate = 1.0 mL/min, λ = 210 nm, 16.3 min (minor isomer), 30.0 min (major isomer); **TLC** (1:4 EtOAc/Hexanes): R_f = 0.38.

Details of crossover experiment:

Three oven-dried test tubes were evacuated and filled with argon, then charged sequentially with the cross pinacol product **(±)-4aa** (49.6 mg, 0.1 mmol) and THF (1.0 mL), followed by 4-fluorobenzaldehyde (42.1 μL, 0.4 mmol, 4.0 equiv). The reaction was stirred at 0 ºC, or –40 ºC, or –78 ºC in an appropriate cooling apparatus, then the iminophosphorane catalyst **C1** (5.81 mg, 0.01 mmol, 0.1 equiv) was added. The reaction was then stirred at the same temperatures for 24 h. Trifluoroacetic acid in toluene (40 μL of an 0.5 M solution) was added to quench the reaction and the reaction was concentrated on a rotatory evaporator.

Analysis of crossover experiment:

The reactions were analyzed by ^{1}H NMR spectroscopy on an 800 MHz spectrometer. The ^{1}H NMR trace show below details the results of the above crossover experiment. The trace on bottom shows racemic starting material (3.2:1 d.r.). The major and minor diastereomer peaks of the starting material are identified with red wedges. As the temperature is increased from –78 ºC to –40 ºC, crossover starts to occur; the major and minor diastereomers of the crossover product are identified with green wedges. Additionally, at 0 ºC the d.r. of the starting material was observed to sharply increase; we attribute this to a relatively rapid retro reaction of the minor diastereomer of starting material (which could also be responsible for the low diastereoselectivity of crossover product at that temperature). These results suggest that the crossover process shuts down between –40 ºC and -78 ºC.

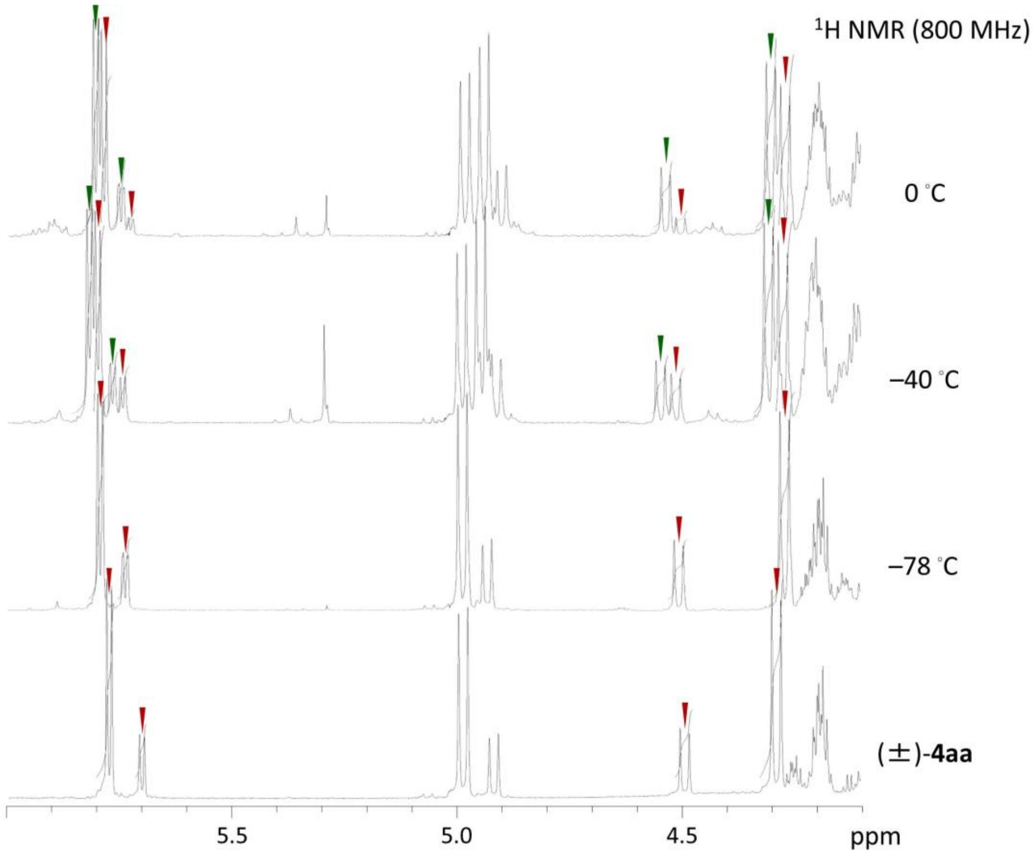

Crystallographic structure determination:

The single crystal, which was obtained by the procedure described below, was mounted on MicroMesh. Data of X-ray diffraction were collected at 93 K on a Bruker D8 QUEST with fine-focus sealed tube Mo/Kα radiation (λ = 0.71073 Å). An absorption correction was made using Bruker SMART APEXII ULTRA. The structure was solved by direct methods and Fourier syntheses, and refined by full-matrix least squares on F^2 by using SHELXTL-2014.[3] All non-hydrogen atoms were refined with anisotropic displacement parameters. Hydrogen atom bonded to oxygen atom was located from a difference synthesis and their coordinates and isotropic thermal parameters refined. The other hydrogen atoms were placed in calculated positions and isotropic thermal parameters refined.

Recrystallization of 4j:

Recrystallization from a solution of **4j** in hexane/CH_2Cl_2 solvent system at room temperature afforded single crystals of **4j**. The crystallographic data were summarized in Table S1 and ORTEP diagram was shown in Fig. S1.

Table S1. Crystal data and structure refinement for **4j** (CCDC-1055582).

Empirical formula	$C_{27}H_{29}BrNO_6P$
Formula weight	574.38
Temperature	93(2) K
Wavelength	0.71073 Å
Crystal system	triclinic
Space group	P1
Unit cell dimensions	a = 10.2391(6) Å α = 91.3040(13)°.
	b = 11.4922(6) Å β = 98.3180(17)°.
	c = 24.9637(12) Å γ = 114.9350(14)°.
Volume	2624.1(2) $Å^3$
Z	4
Density (calculated)	1.454 g/cm^3
Absorption coefficient	1.670 mm^{-1}
F(000)	1184
Crystal size	0.080 x 0.160 x 0.410 mm^3
Theta range for data collection	2.20 to 24.96°.
Index ranges	–12<=h<=10, –12<=k<=13, –28<=l<=29
Reflections collected	17679
Independent reflections	13136 [R(int) = 0.0359]
Completeness to theta = 27.46°	95.0 %
Absorption correction	multi-scan
Max. and min. transmission	0.8780 and 0.5480
Refinement method	Full-matrix least-squares on F^2
Data / restraints / parameters	13136 / 1803 / 1313
Goodness-of-fit on F^2	1.077
Final R indices [I>2sigma(I)]	R_1 = 0.0399, wR_2 = 0.1044
R indices (all data)	R_1 = 0.0453, wR_2 = 0.1249
Absolute structure parameter	0.1(0)
Largest diff. peak and hole	0.955 and –0.754 e.$Å^{-3}$

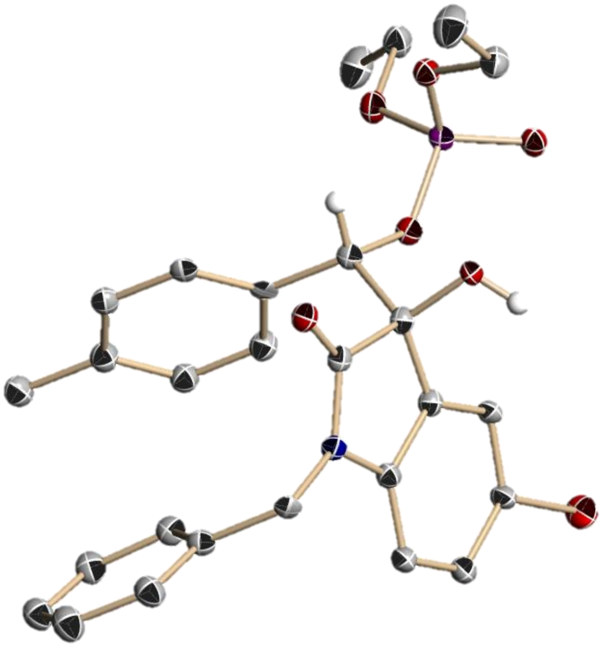

Fig. S1. ORTEP diagram of **4j**. All calculated hydrogen atoms are omitted for clarity except for that attached to the olefinic carbons. Blue = nitrogen, red = oxygen, vermilion = bromine, gray = carbon.

References and notes:

1. a) L. E. Overman, E. A. Peterson, *Tetrahedron* 2003, **59**, 6905-6919. b) D. J. Vyas, R. Froehlich, M. Oestreich, *J. Org. Chem.* 2010, 75, 6720-6723. c) R. Bouhfid, N. Joly, E. M. Essassi, V. Lequart, M. Massoui, P. Martin, *Synth. Commun.* 2011, **41**, 2096-2102.
2. D. Uraguchi, K. Yoshioka, Y. Ueki, T. Ooi, *J. Am. Chem. Soc.* 2012, **134**, 19370-19373.
3. G. M. Sheldrick, *Acta Cryst.* 2008, **A64**, 112.

Crude ^{1}H NMR spectra for the racemic reaction:

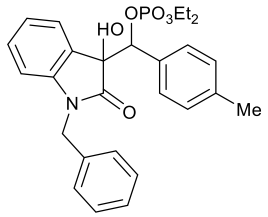

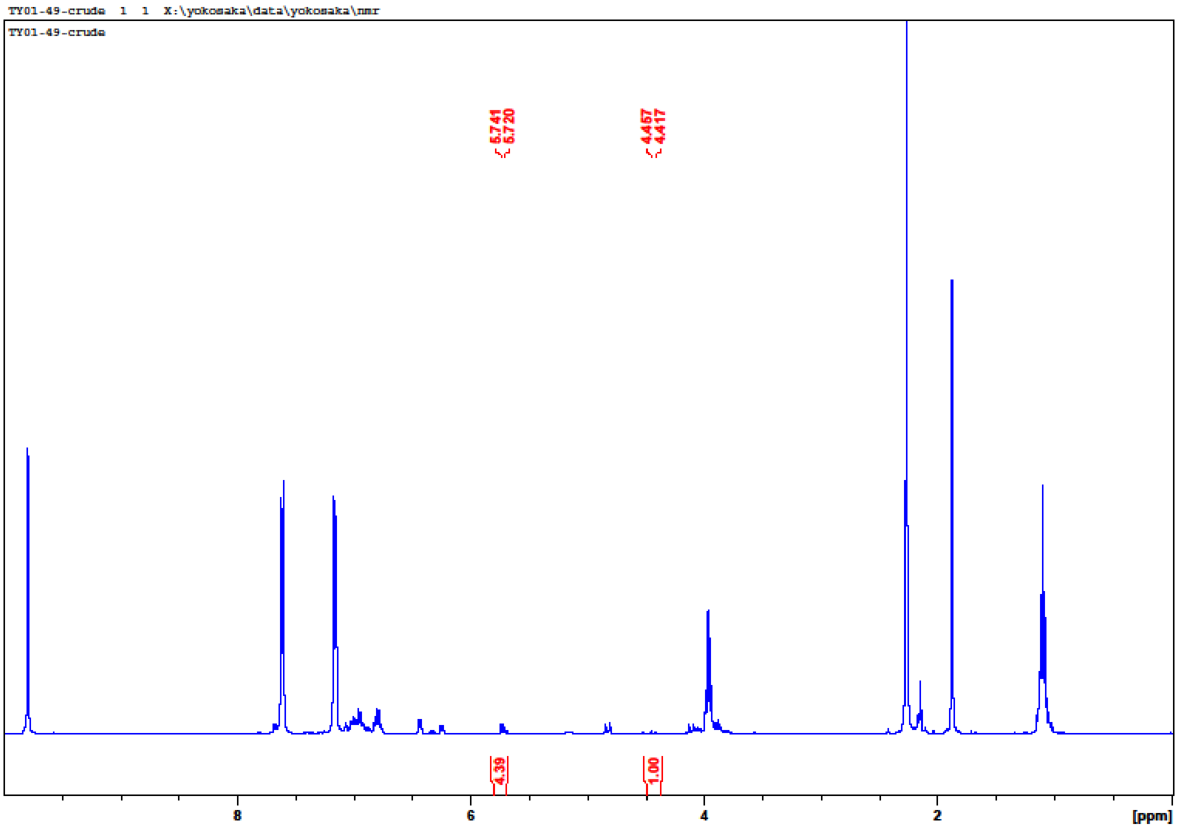

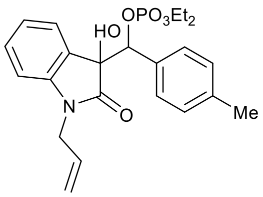
OPO3Et2
HO
N
O
Me

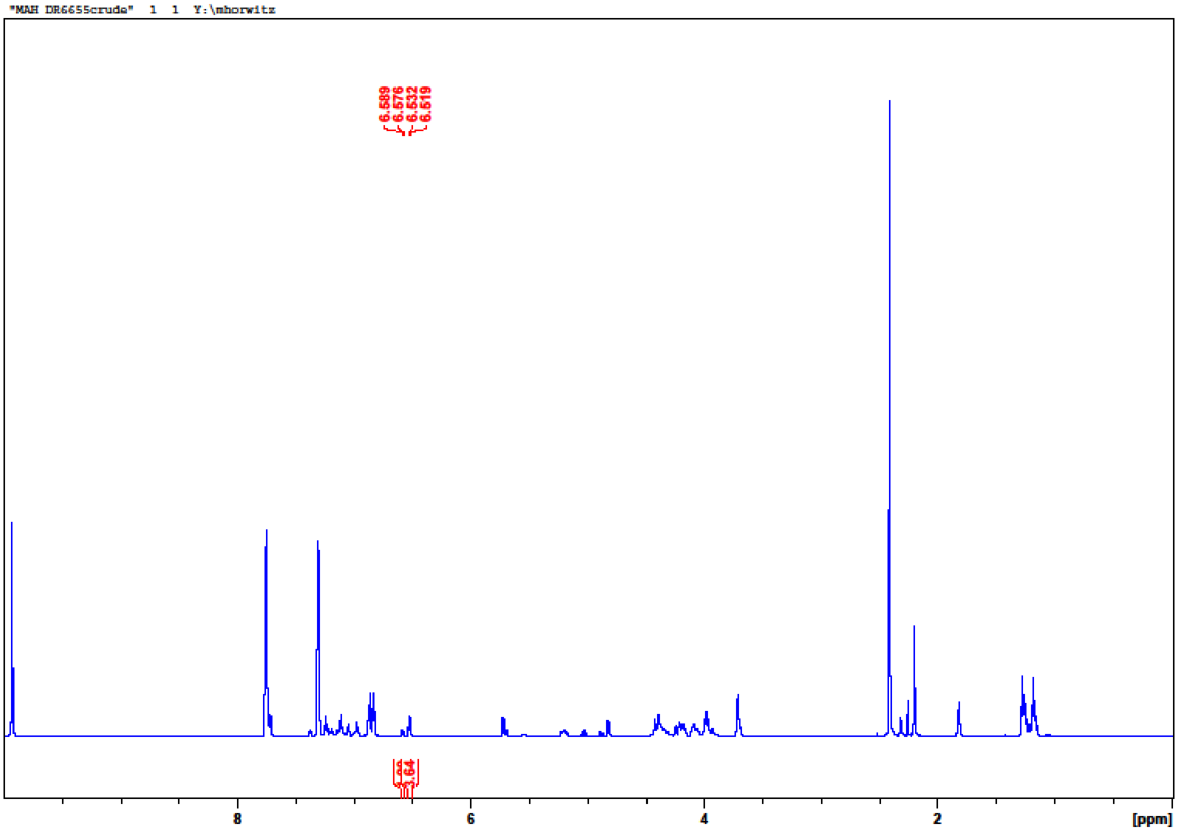
"MAH DR6655crude" 1 1 Y:\mhorwitz
6.589
6.576
6.532
6.519
1.64
8
6
4
2
[ppm]

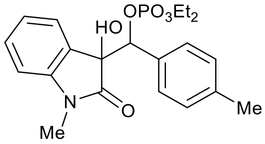
OPO3Et2
HO
N
O
Me
Me

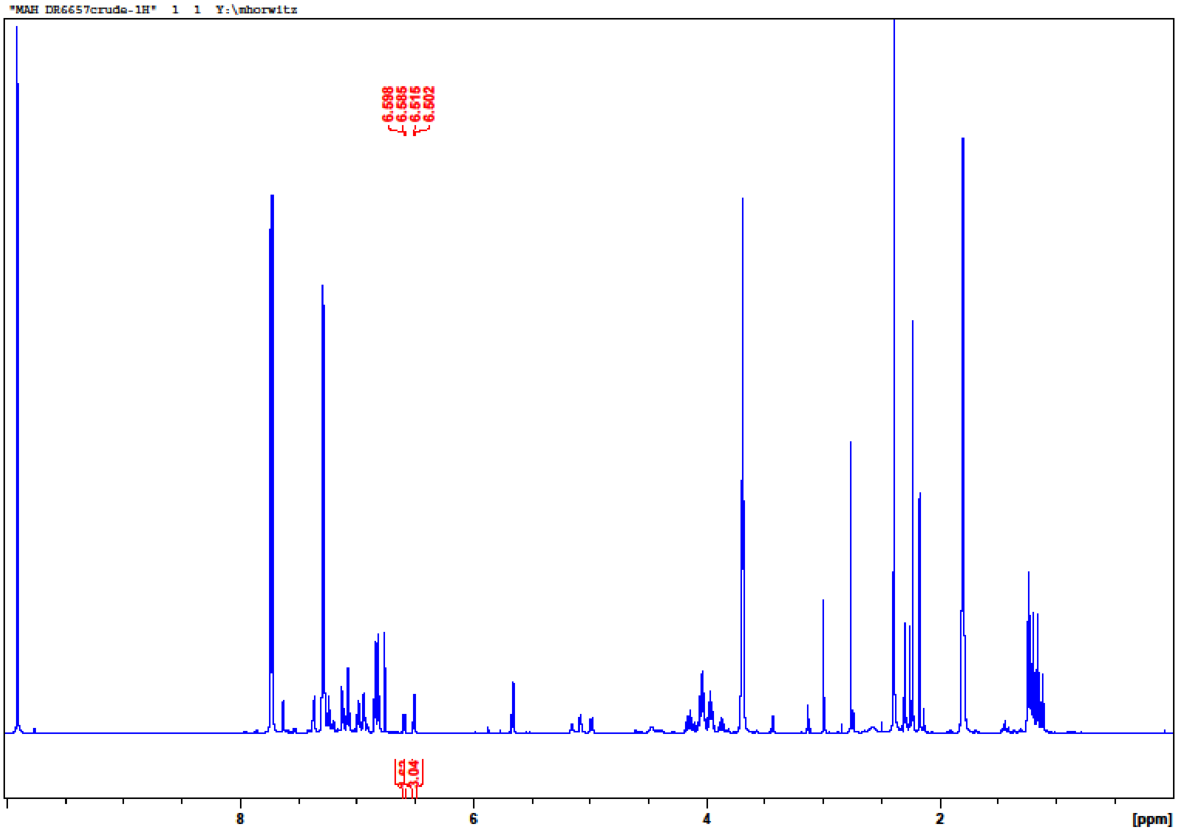
"MAH DR6657crude-1H" 1 1 Y:\mhorwitz
6.598
6.585
6.515
6.502
1.04
8
6
4
2
[ppm]

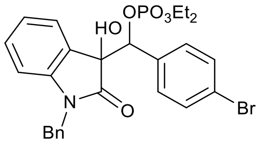
OPO3Et2
HO
N
O
Bn
Br

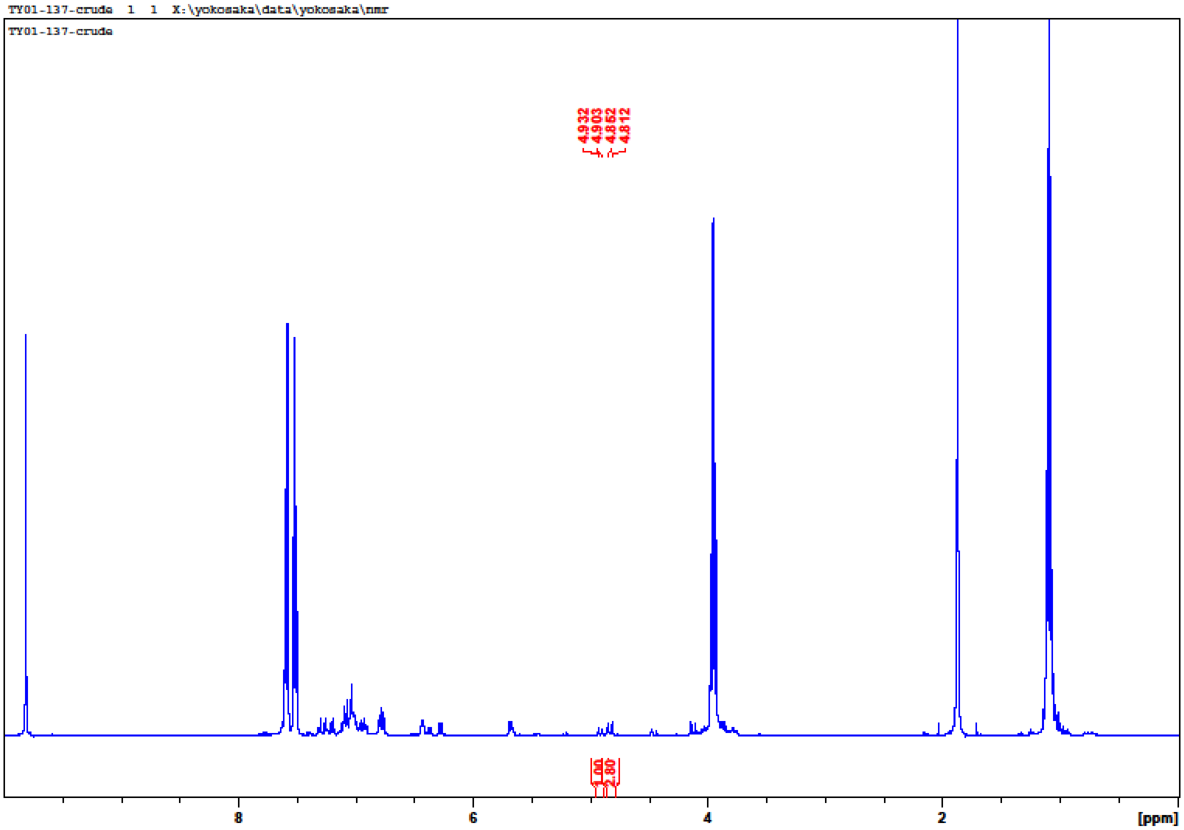
TY01-137-crude 1 1 X:\yokosaka\data\yokosaka\nmr
TY01-137-crude
4.932
4.903
4.852
4.812
8
6
4
2
[ppm]

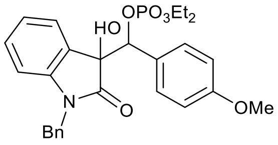
OPO3Et2
HO
N
O
Bn
OMe

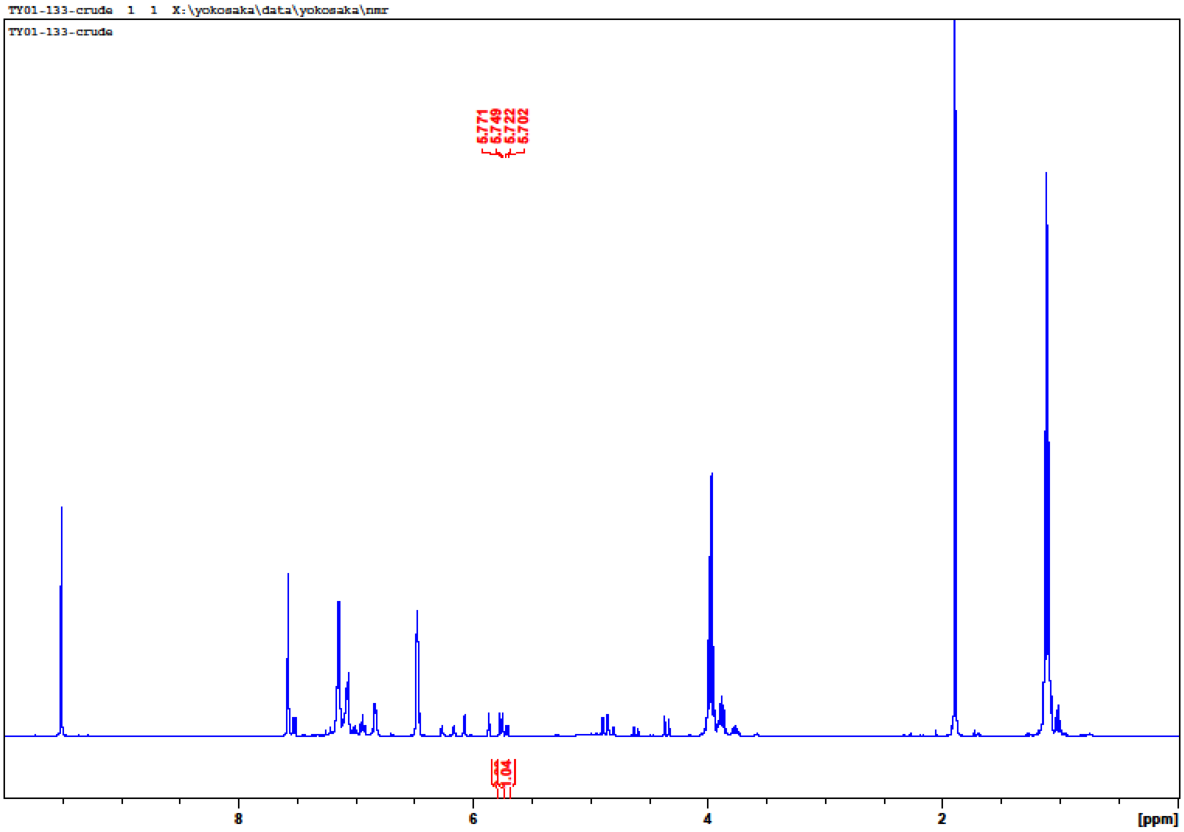
TY01-133-crude 1 1 X:\yokosaka\data\yokosaka\nmr
TY01-133-crude
5.771
5.749
5.722
5.702
1.04
8
6
4
2
[ppm]

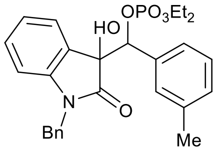
OPO3Et2
HO
N
O
Bn
Me

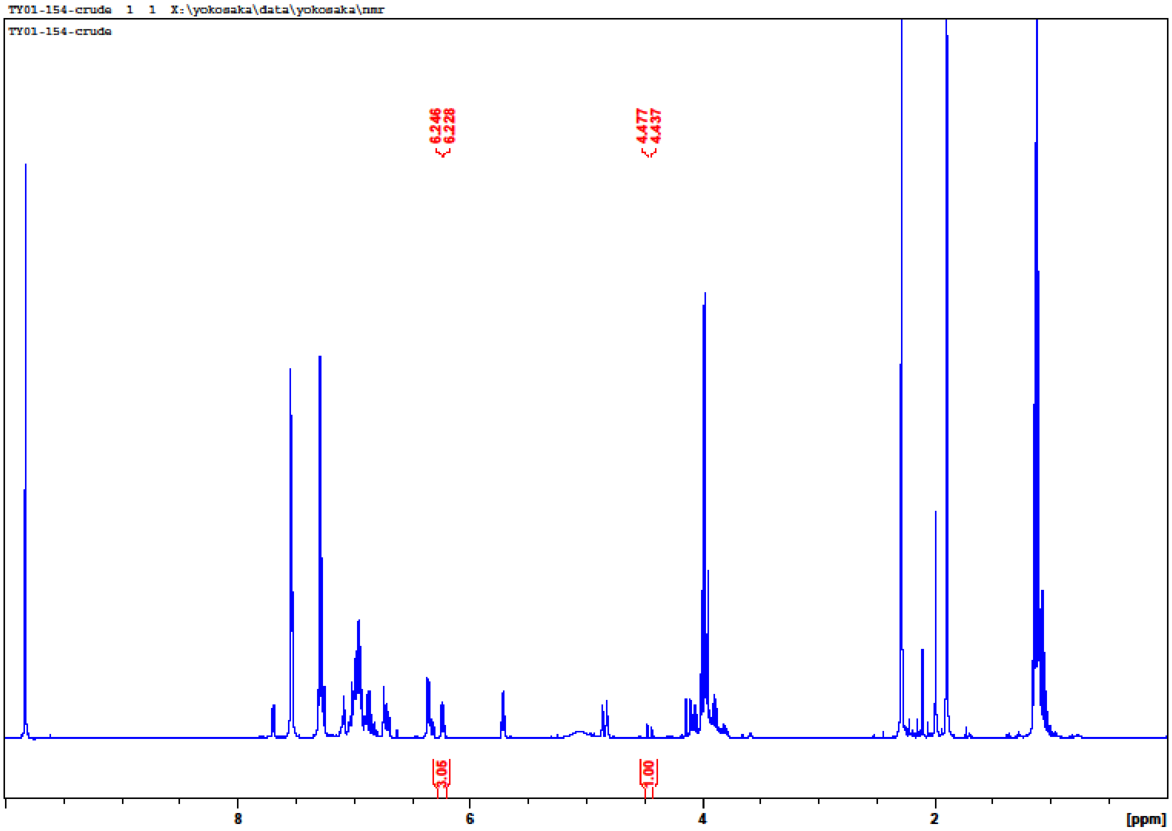
TY01-154-crude 1 1 X:\yokosaka\data\yokosaka\nmr
TY01-154-crude
6.246
6.228
4.477
4.437
3.05
1.00
8
6
4
2
[ppm]

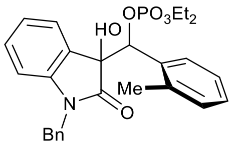
OPO3Et2
HO
Me
N
O
Bn

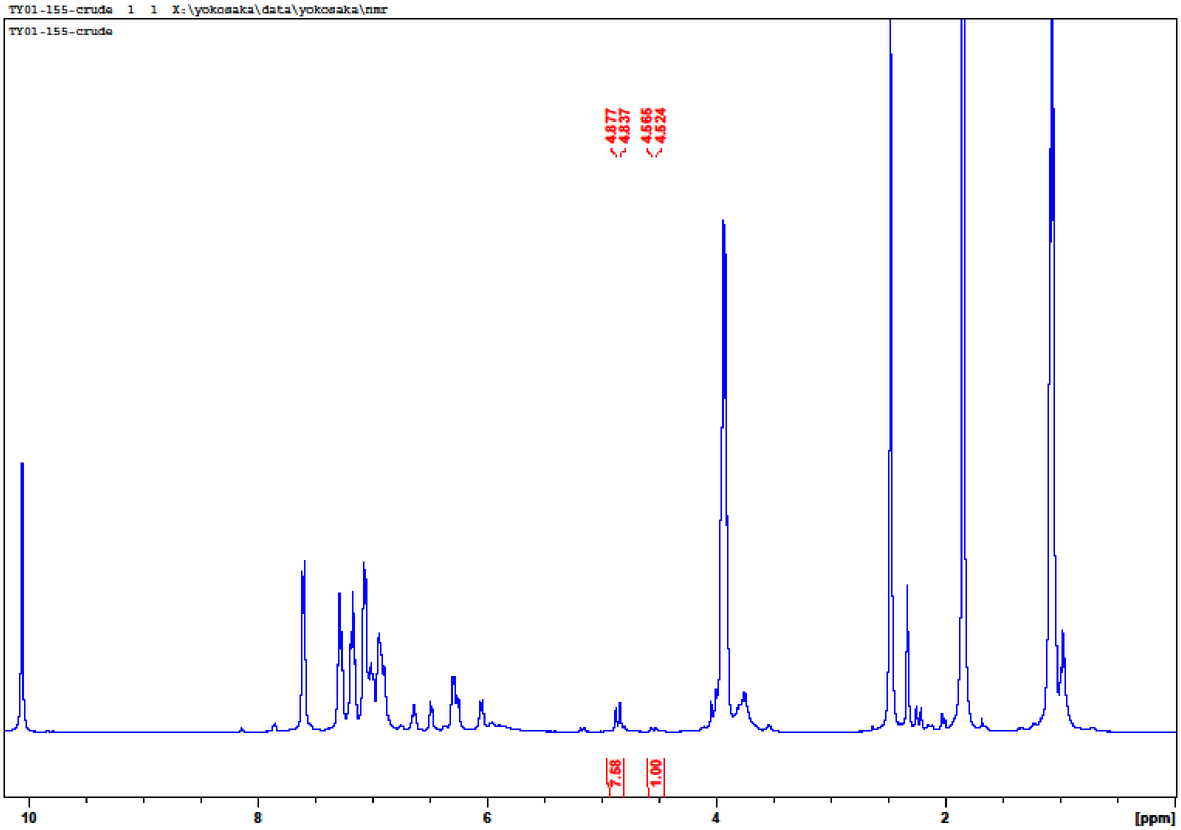
TY01-155-crude 1 1 X:\yokosaka\data\yokosaka\nmr
TY01-155-crude
4.877
4.837
4.565
4.524
7.58
1.00
10
8
6
4
2
[ppm]

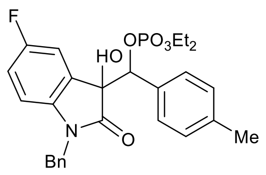
F
HO
OPO3Et2
N
O
Bn
Me

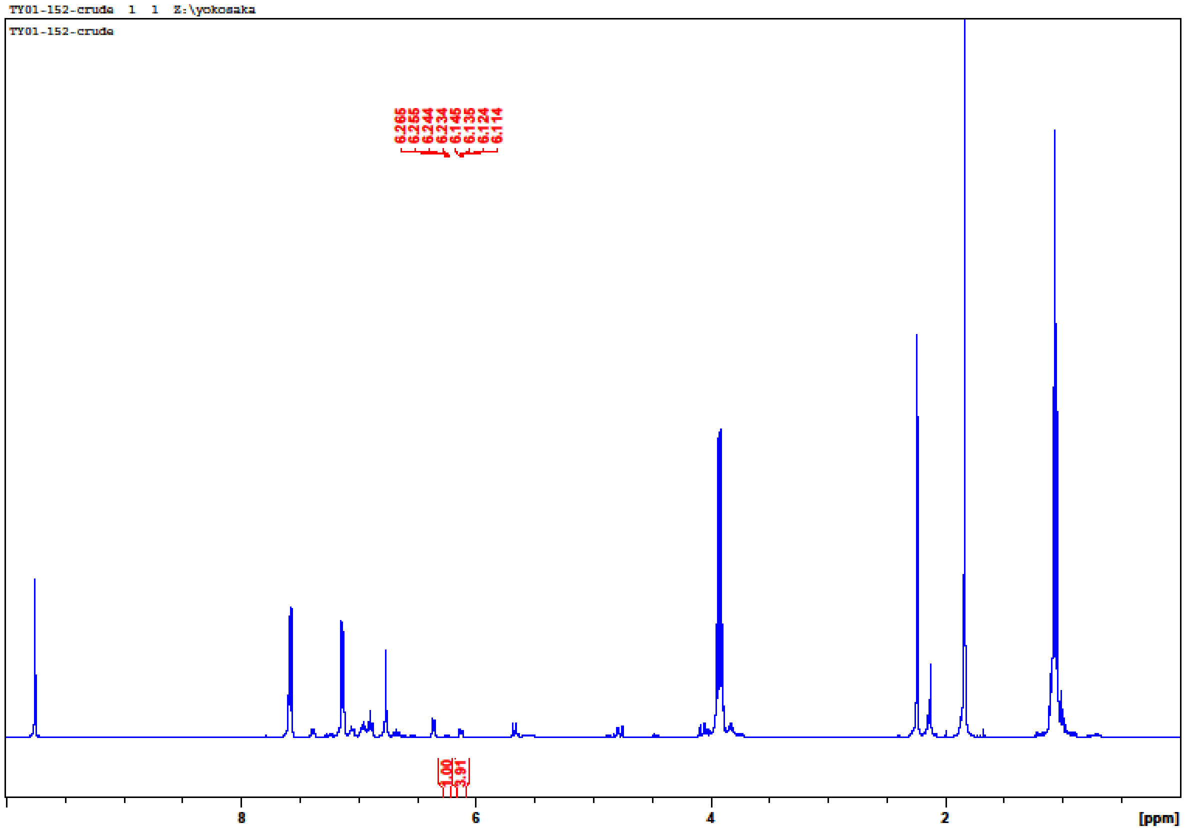
TY01-152-crude 1 1 Z:\yokosaka
TY01-152-crude
6.265
6.255
6.244
6.234
6.145
6.135
6.124
6.114
1.00
3.91
8
6
4
2
[ppm]

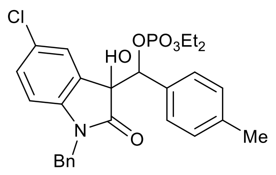
Cl
HO
OPO3Et2
N
O
Bn
Me

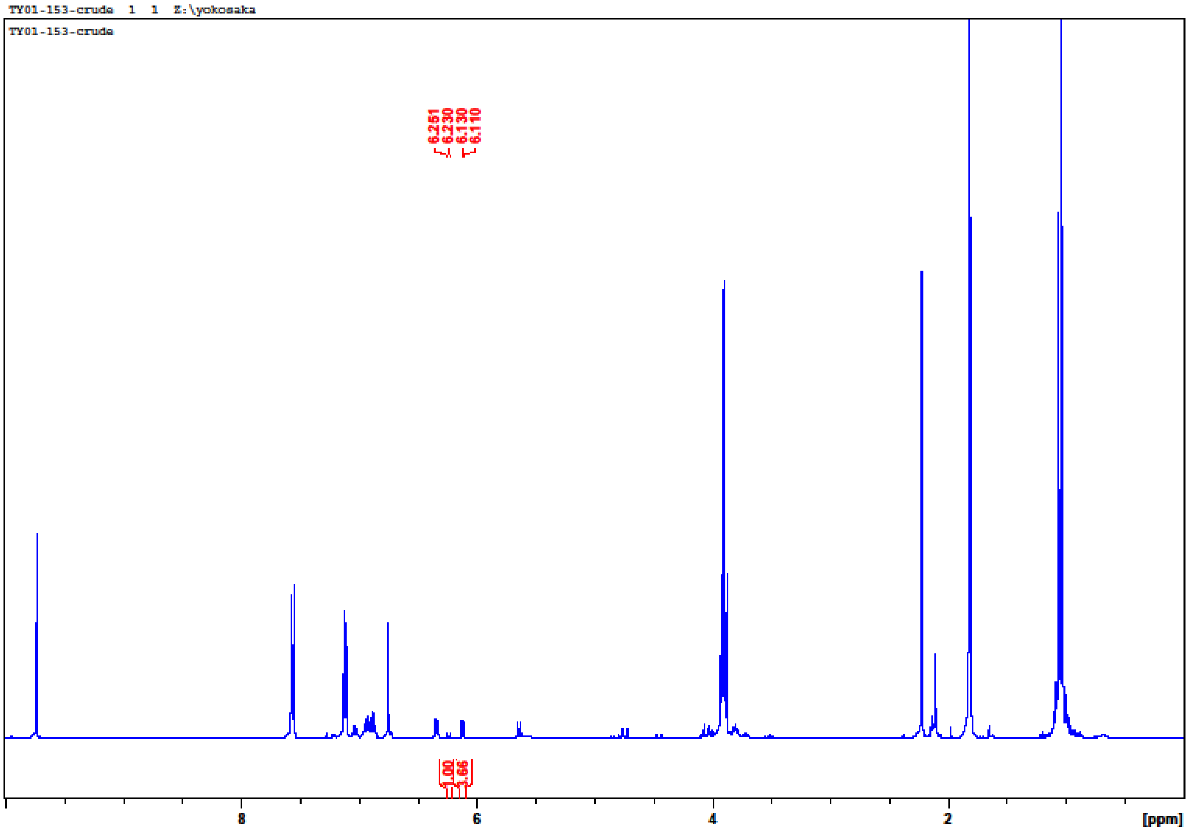
TY01-153-crude 1 1 E:\yokosaka
TY01-153-crude
6.251
6.230
6.130
6.110
1.00
1.66
8
6
4
2
[ppm]

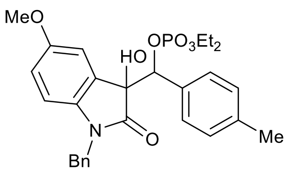

MeO
HO
OPO3Et2
N
O
Bn
Me

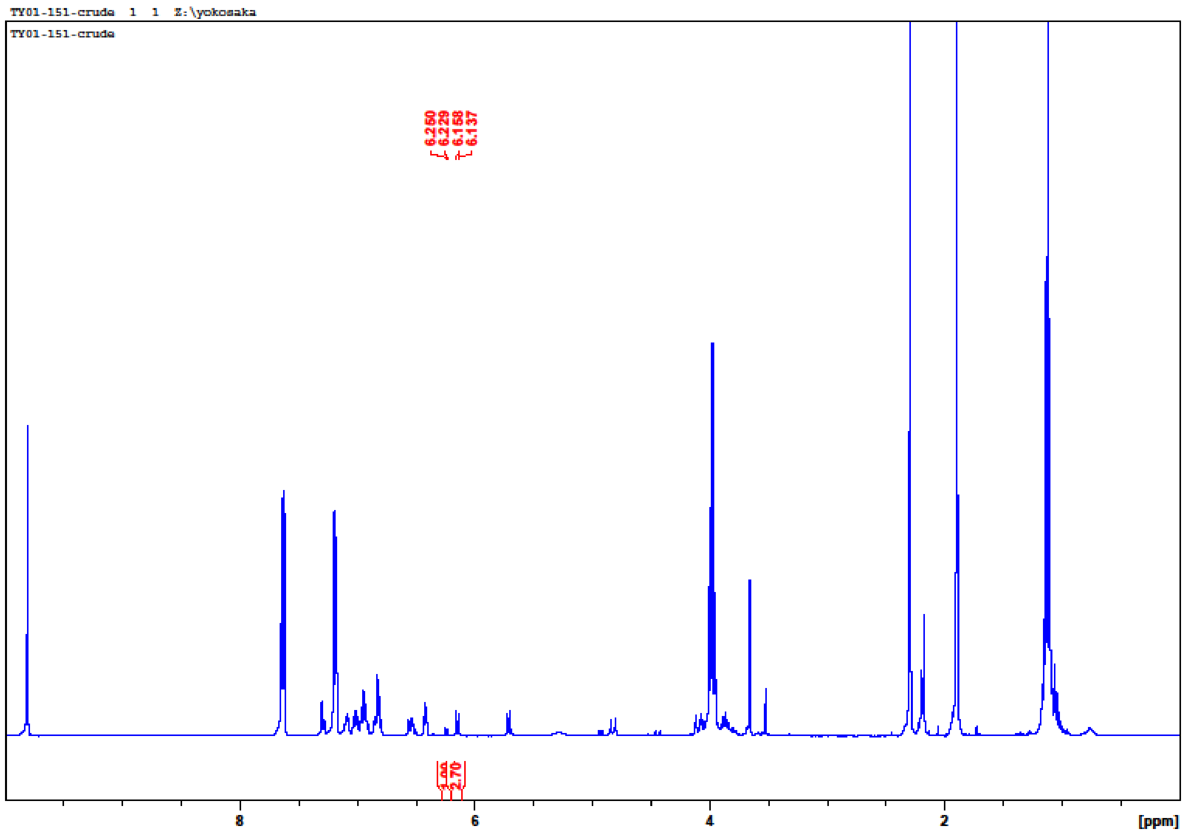

TY01-151-crude 1 1 E:\yokosaka
TY01-151-crude
6.250
6.229
6.158
6.137
8
6
4
2
[ppm]

Crude ^{1}H NMR spectra for the asymmetric reaction:

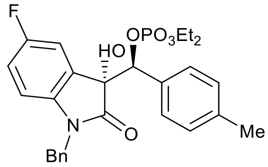

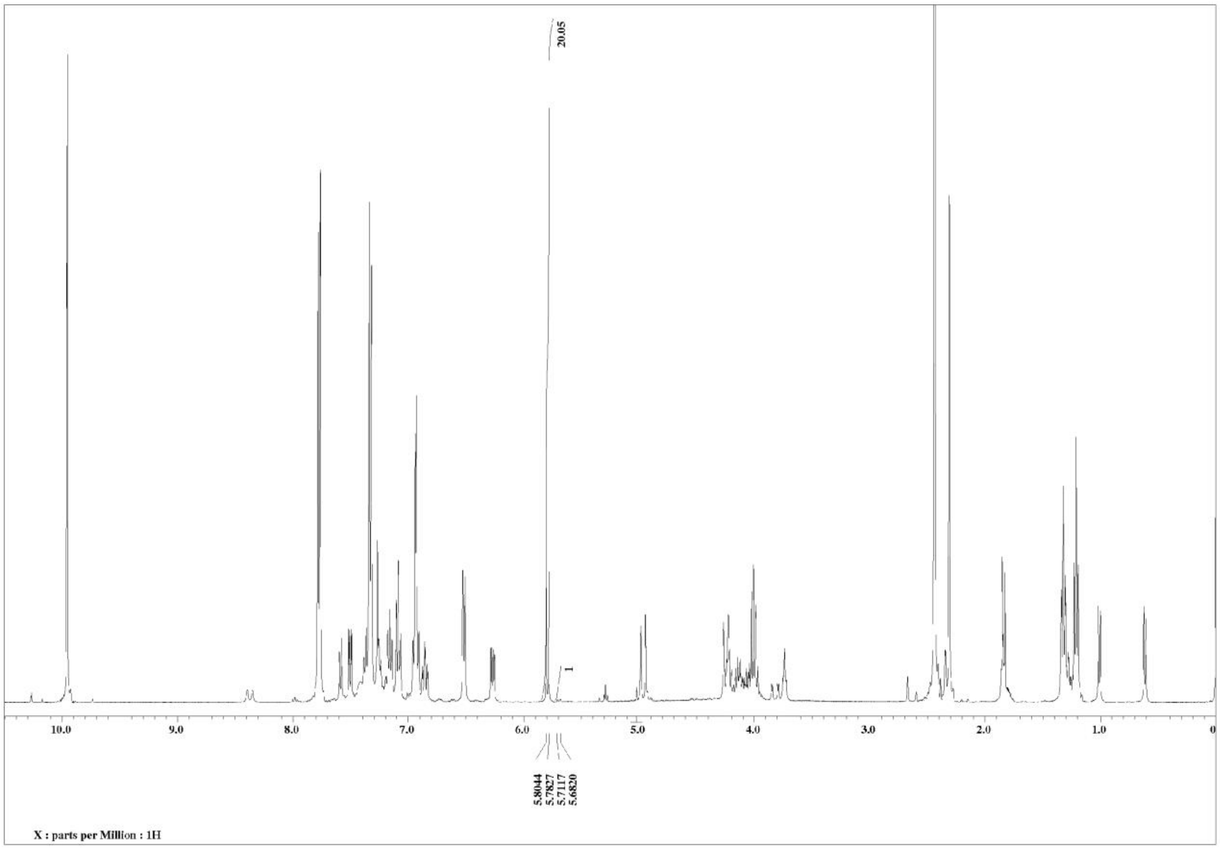

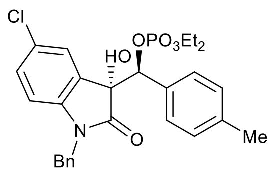
Cl
HO
OPO$_3$Et$_2$
N
O
Bn
Me

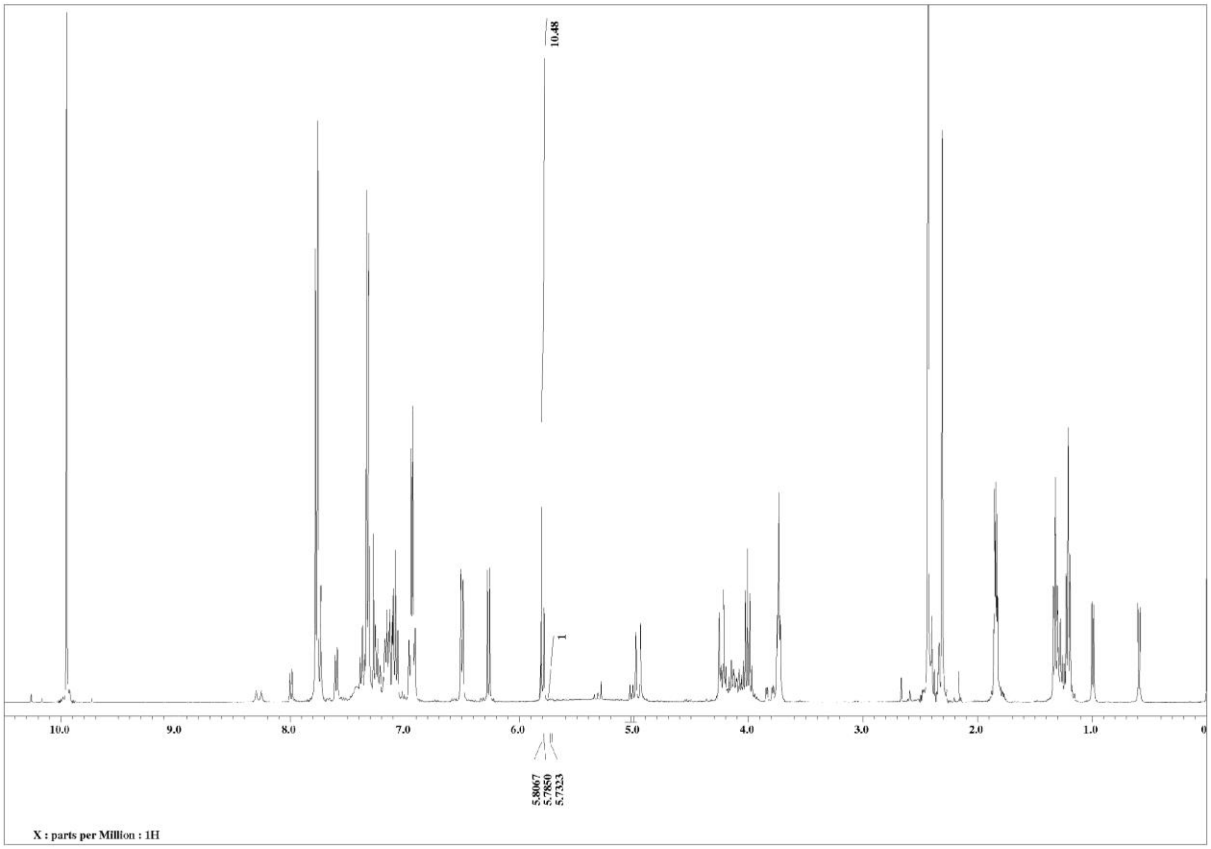
10.48
1
10.0
9.0
8.0
7.0
6.0
5.0
4.0
3.0
2.0
1.0
5.8067
5.7850
5.7323
X : parts per Million : 1H

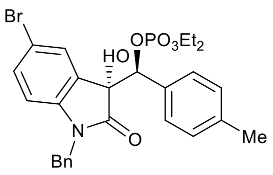
Br
OPO3Et2
HO
N
O
Bn
Me

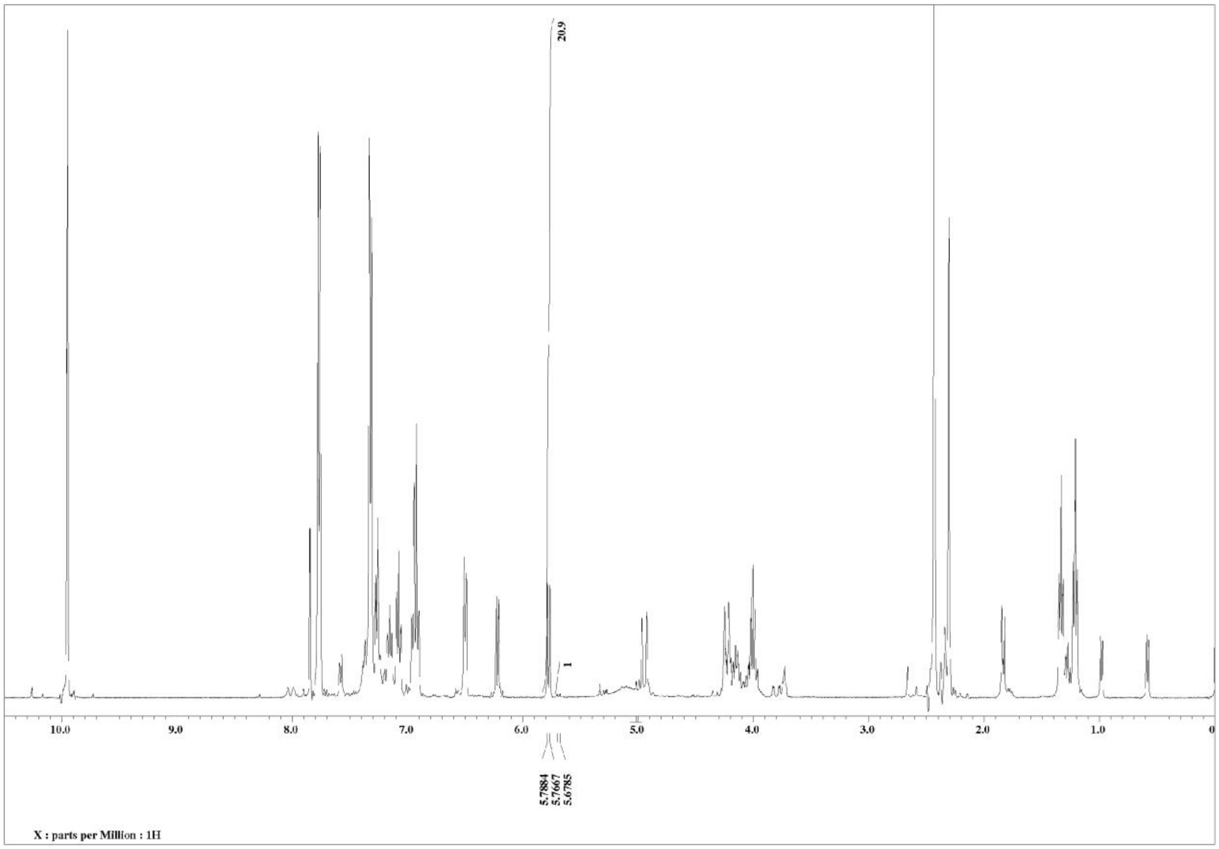
20.9
1
10.0
9.0
8.0
7.0
6.0
5.0
4.0
3.0
2.0
1.0
0
5.7884
5.7667
5.6785
X : parts per Million : 1H

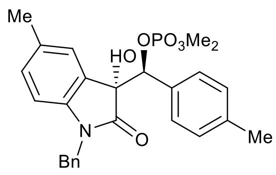

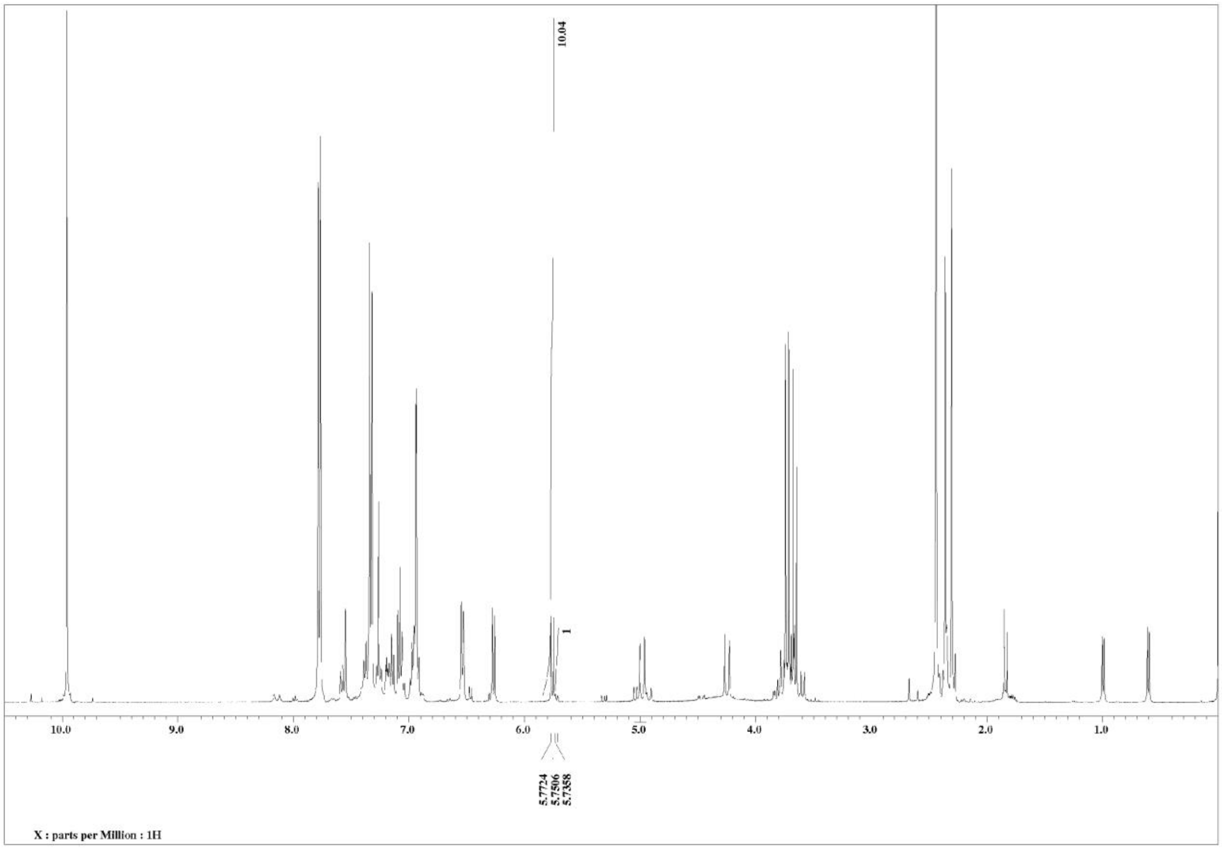
10.04
1
10.0
9.0
8.0
7.0
6.0
5.0
4.0
3.0
2.0
1.0
5.7724
5.7506
5.7358
X : parts per Million : 1H

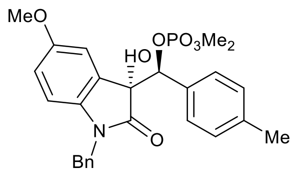
MeO
OPO3Me2
HO
N
O
Me
Bn

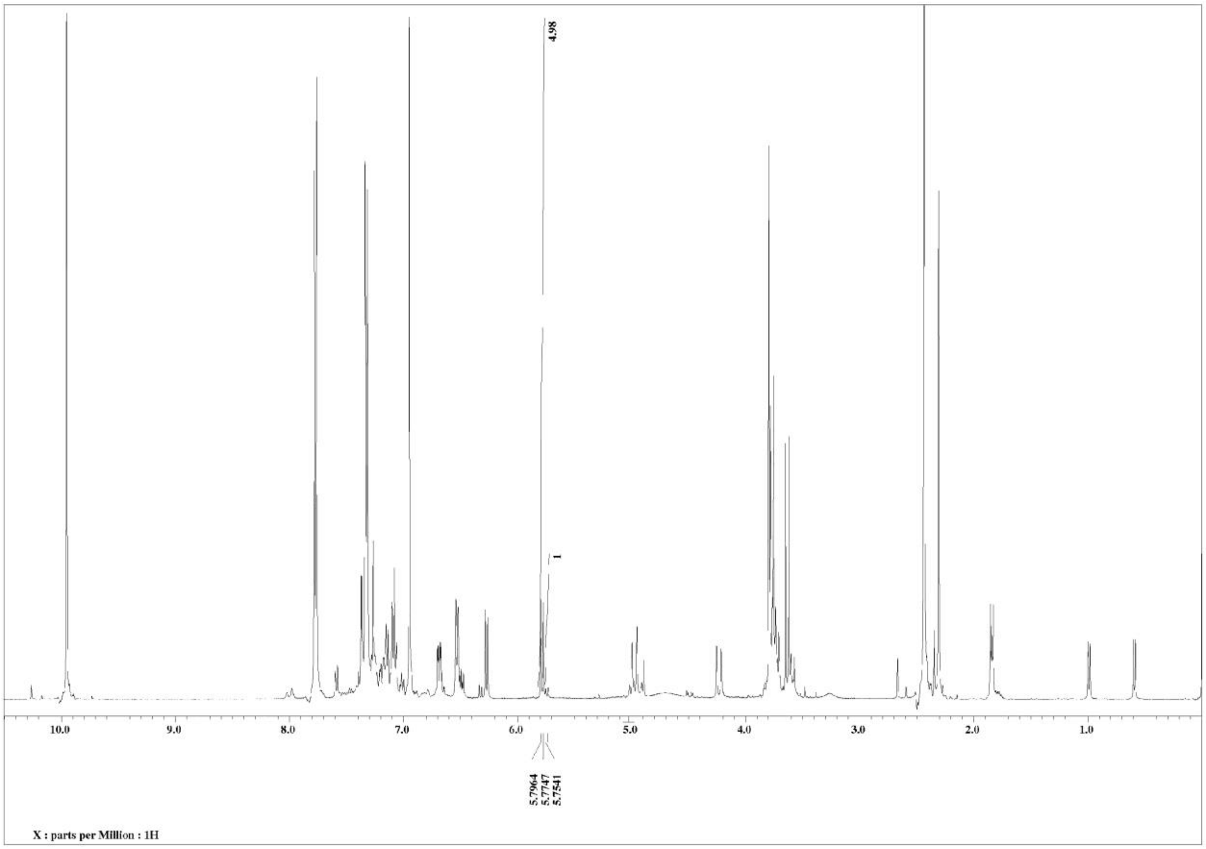
4.98
1
10.0
9.0
8.0
7.0
6.0
5.0
4.0
3.0
2.0
1.0
5.7964
5.7747
5.7541
X : parts per Million : 1H

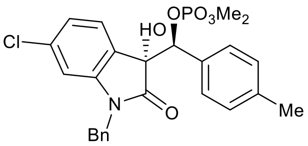

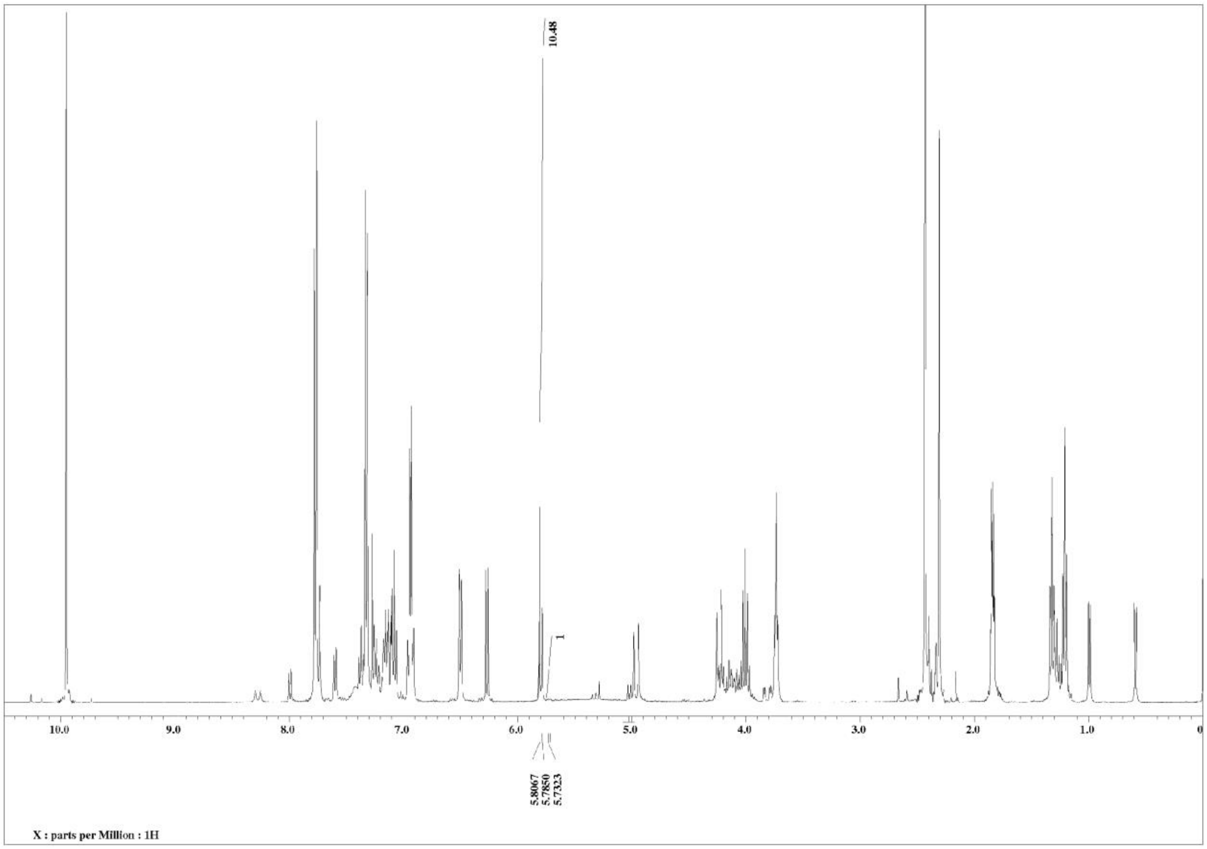
10.48
1
10.0
9.0
8.0
7.0
6.0
5.0
4.0
3.0
2.0
1.0
5.8067
5.7850
5.7323
X : parts per Million : 1H

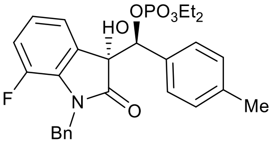

OPO3Et2
HO
F
N
O
Bn
Me

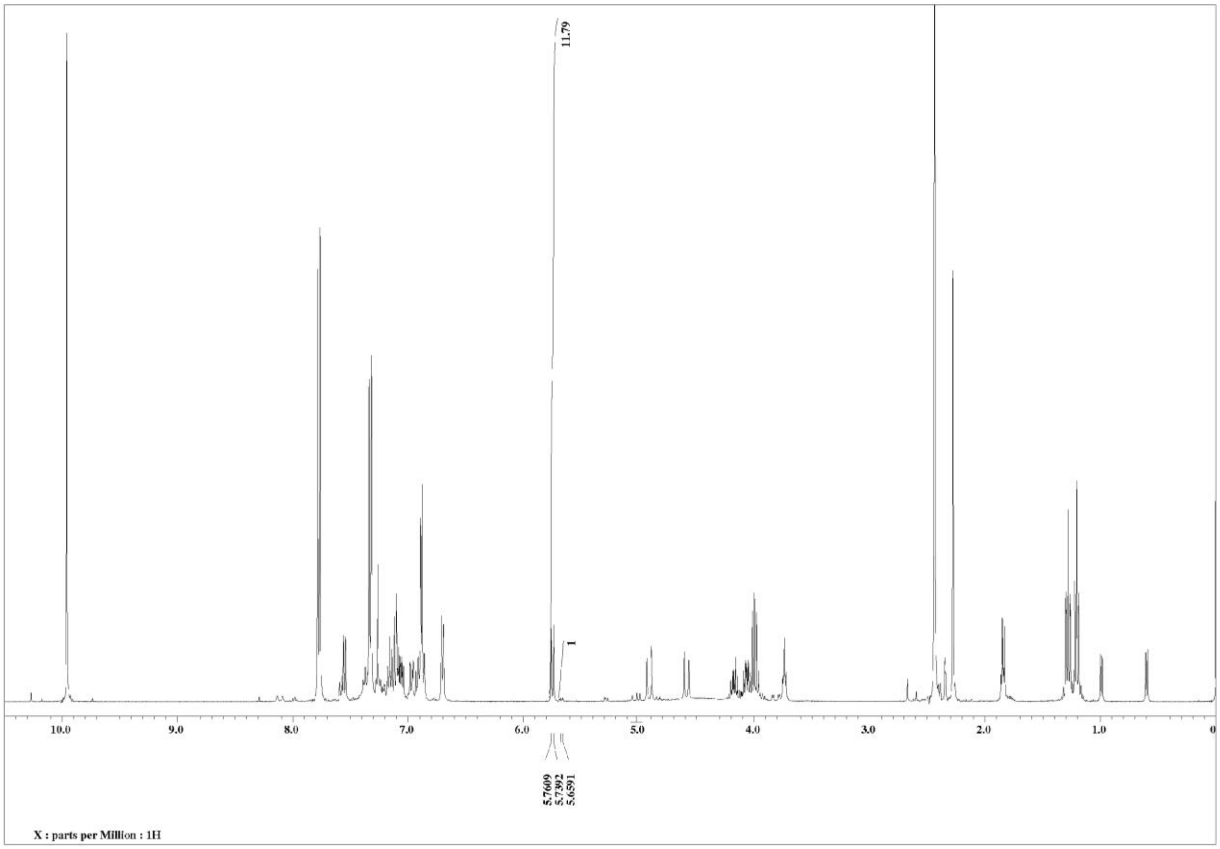

11.79
1
10.0
9.0
8.0
7.0
6.0
5.0
4.0
3.0
2.0
1.0
5.7609
5.7392
5.6591
X : parts per Million : 1H

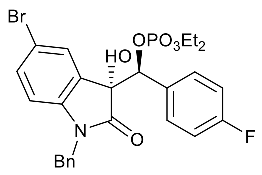
Br
OPO3Et2
HO
N
O
F
Bn

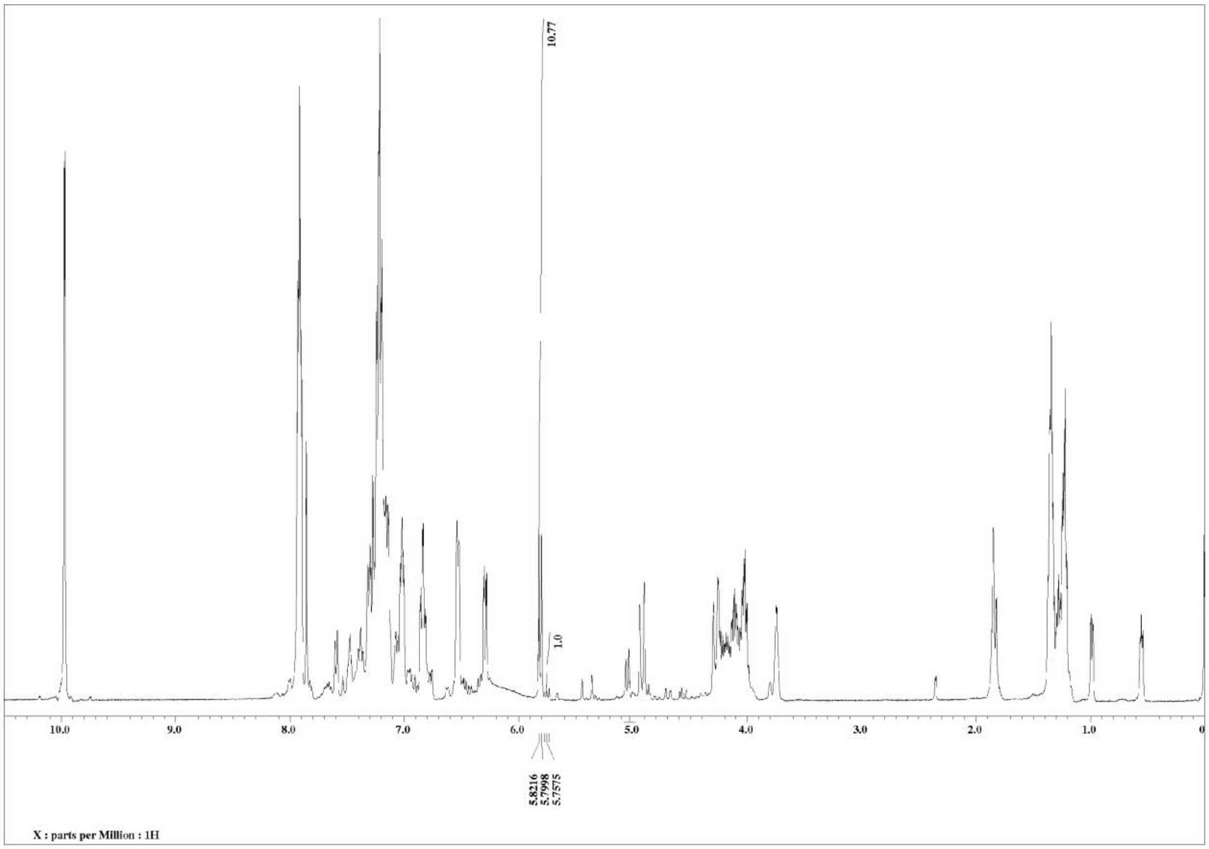
10.77
1.0
10.0
9.0
8.0
7.0
6.0
5.0
4.0
3.0
2.0
1.0
5.8216
5.7998
5.7575
X : parts per Million : 1H

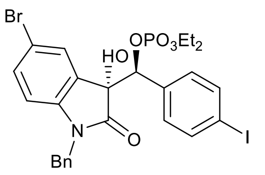

Br
OPO3Et2
HO
N
O
I
Bn

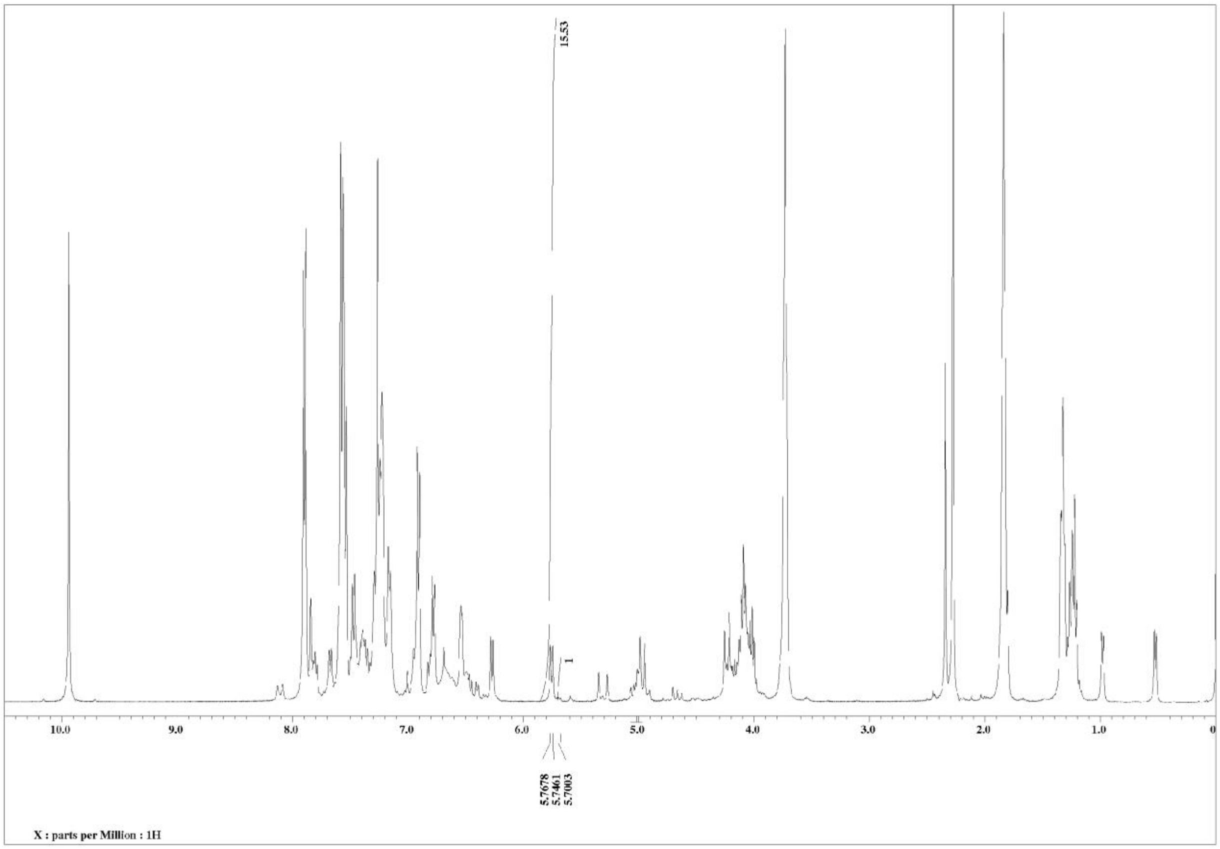

15.53
1
10.0
9.0
8.0
7.0
6.0
5.0
4.0
3.0
2.0
1.0
0
5.7678
5.7461
5.7003
X : parts per Million : 1H

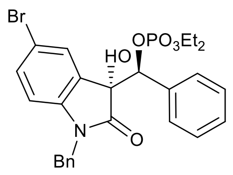

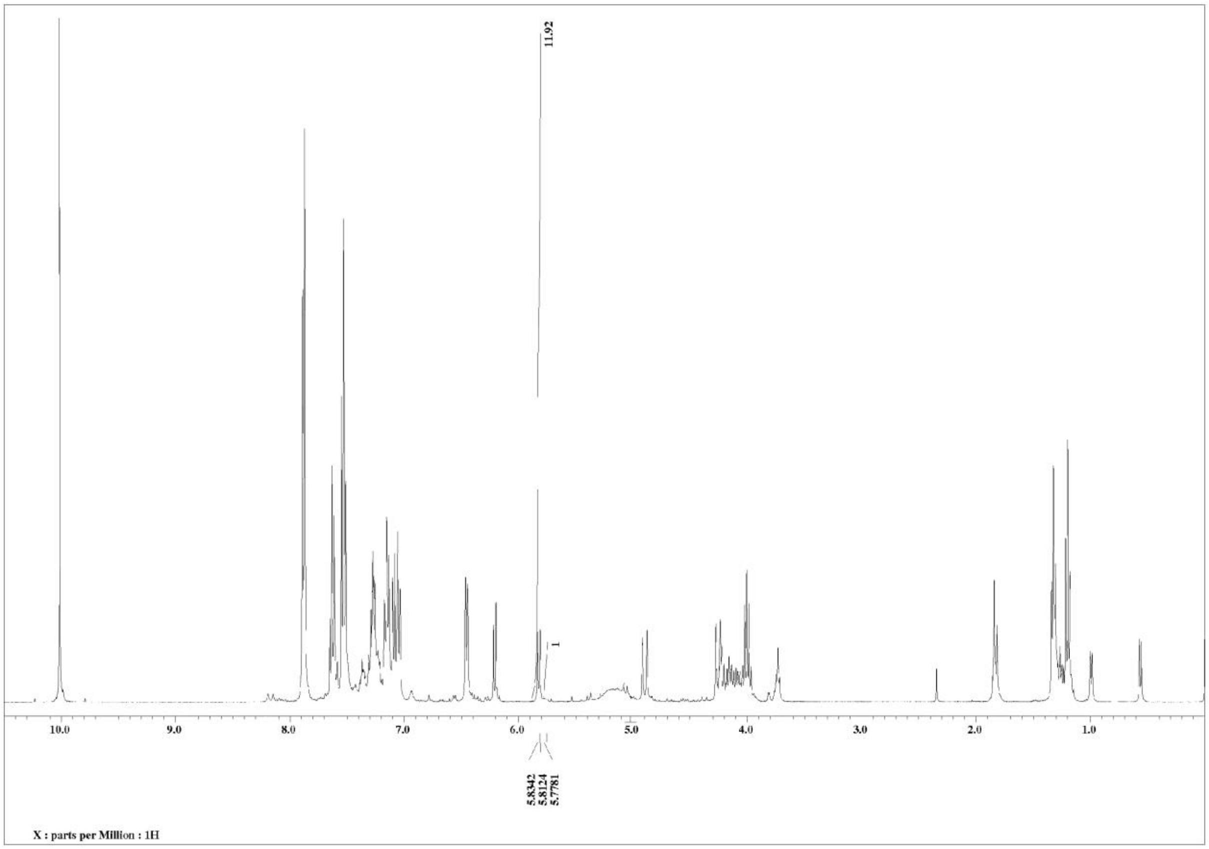

11.92
1
10.0
9.0
8.0
7.0
6.0
5.0
4.0
3.0
2.0
1.0
5.8342
5.8124
5.7781
X : parts per Million : 1H

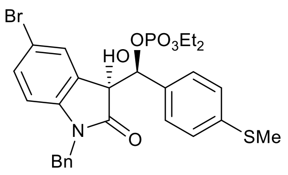
Br
OPO3Et2
HO
N
O
Bn
SMe

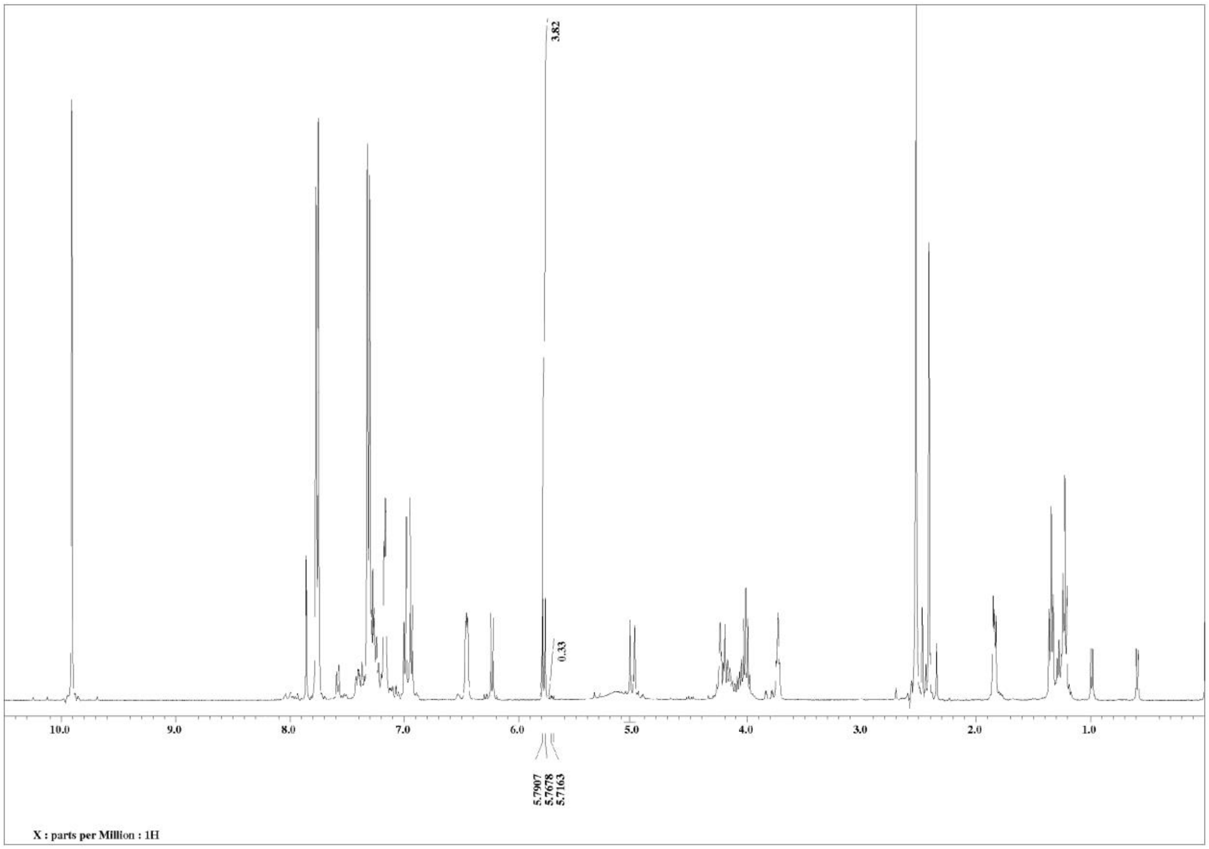
3.82
0.33
10.0
9.0
8.0
7.0
6.0
5.0
4.0
3.0
2.0
1.0
5.7907
5.7678
5.7163
X : parts per Million : 1H

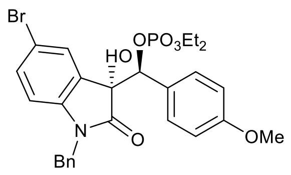

Br
OPO3Et2
HO
N
O
Bn
OMe

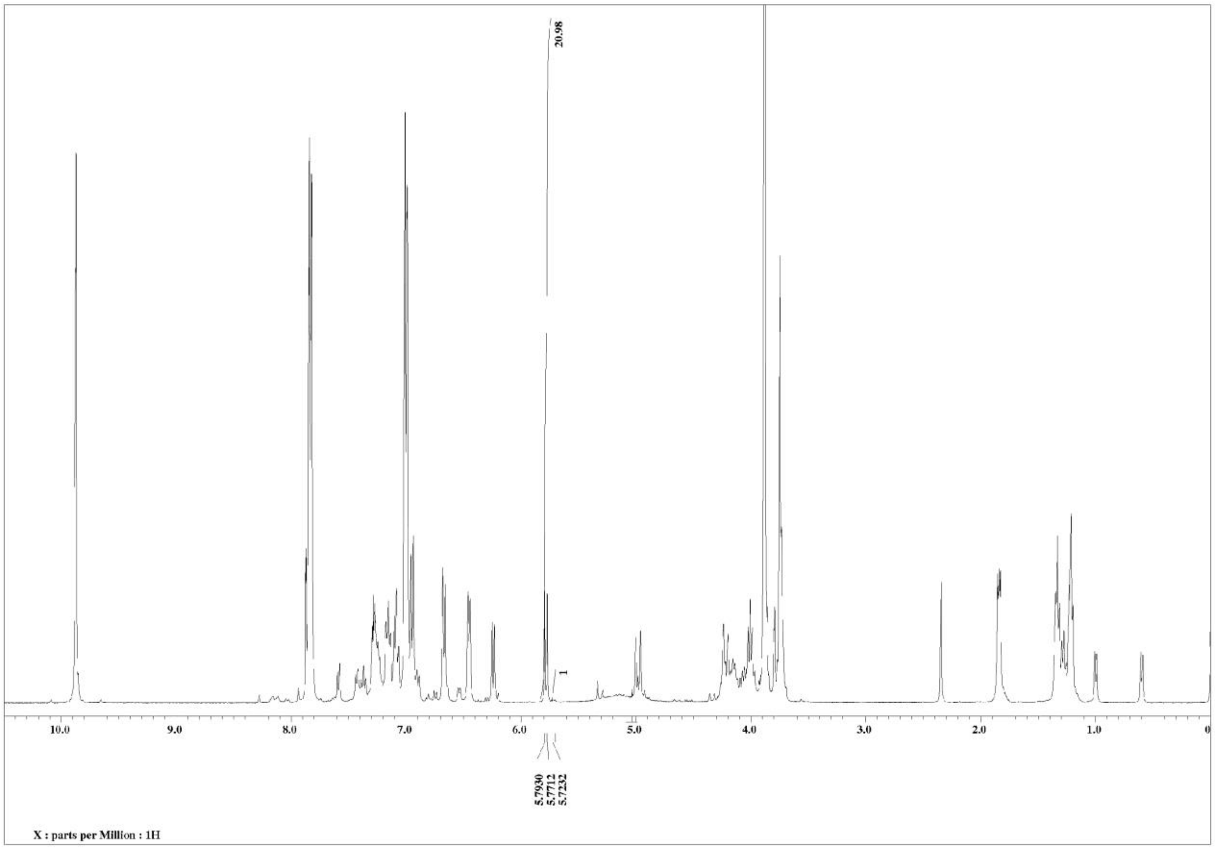

20.98
1
10.0
9.0
8.0
7.0
6.0
5.0
4.0
3.0
2.0
1.0
0
5.7930
5.7712
5.7232
X : parts per Million : 1H

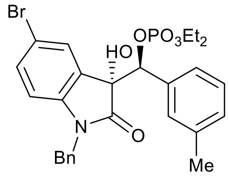

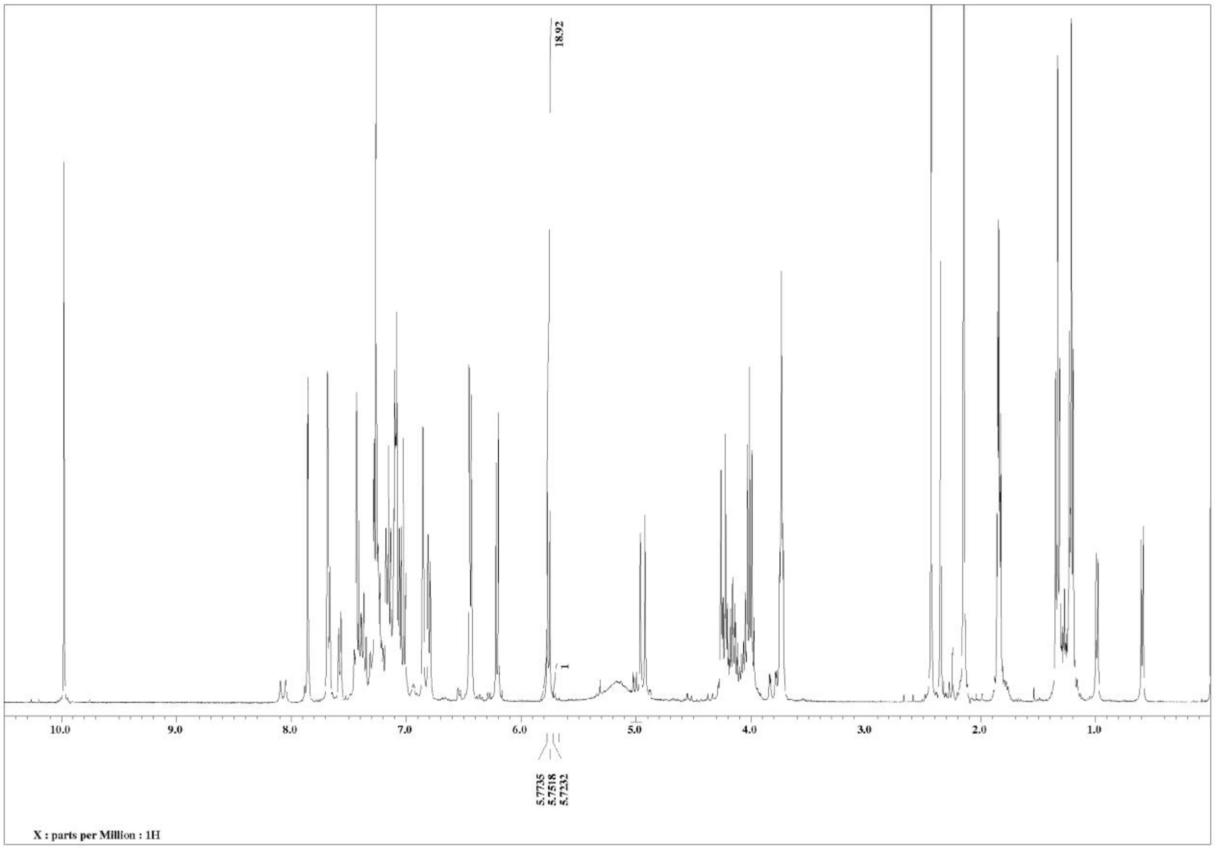

18.92
10.0
9.0
8.0
7.0
6.0
5.0
4.0
3.0
2.0
1.0
5.7735
5.7518
5.7232
X : parts per Million : 1H

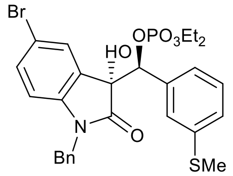
Br
OPO3Et2
HO
N
O
Bn
SMe

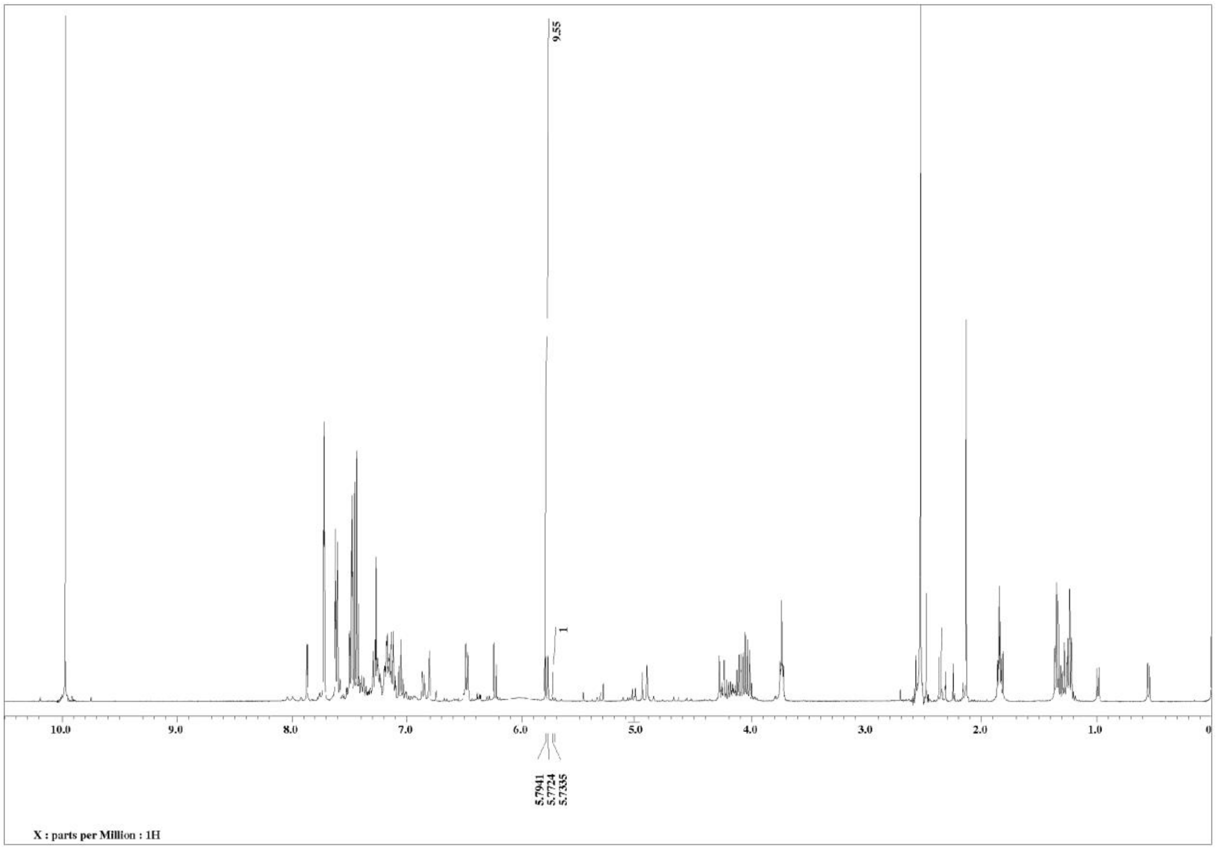
9.55
1
10.0
9.0
8.0
7.0
6.0
5.0
4.0
3.0
2.0
1.0
0
5.7941
5.7724
5.7335
X : parts per Million : 1H

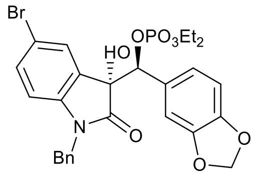
Br
OPO3Et2
HO
N
O
Bn
O
O

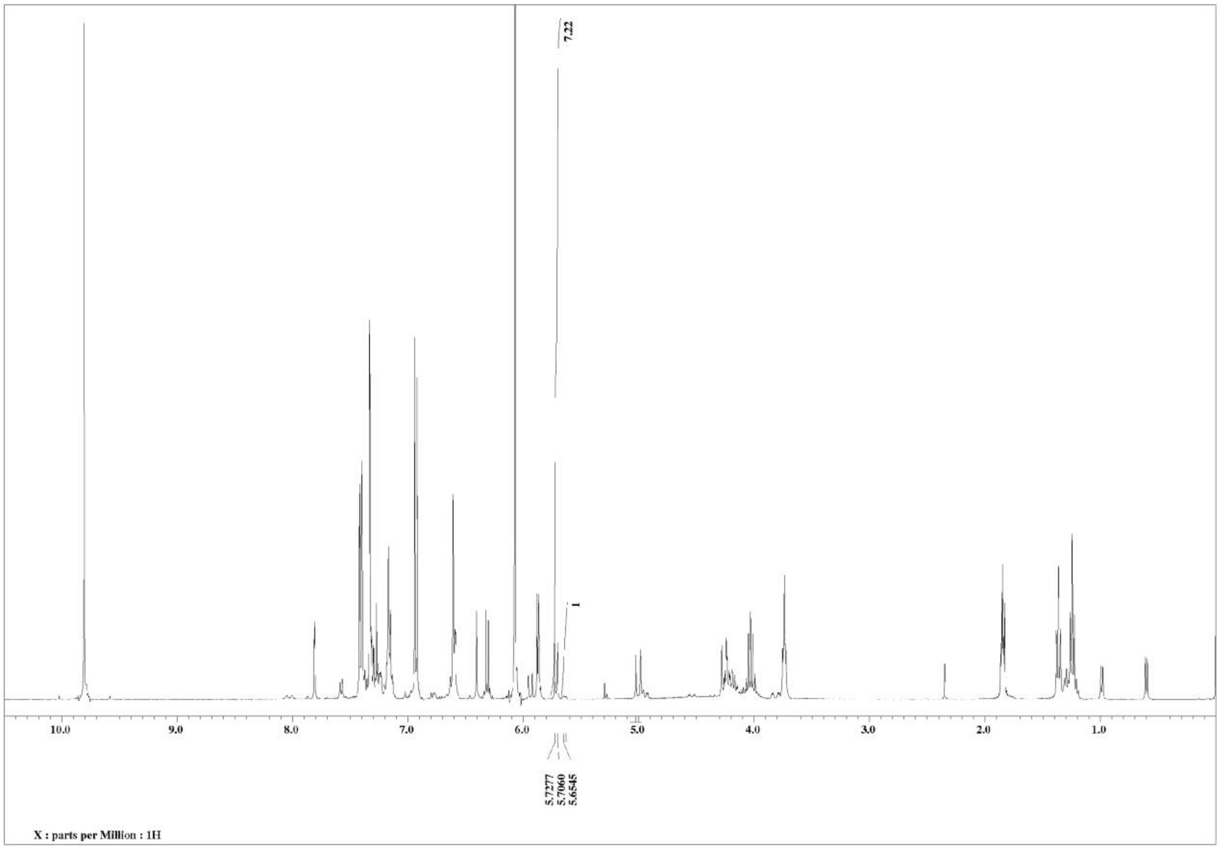
7.22
1
10.0
9.0
8.0
7.0
6.0
5.0
4.0
3.0
2.0
1.0
5.7277
5.7060
5.6545
X : parts per Million : 1H

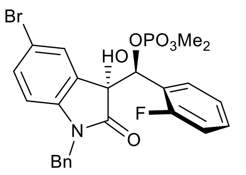
Br
OPO3Me2
HO
F
N
O
Bn

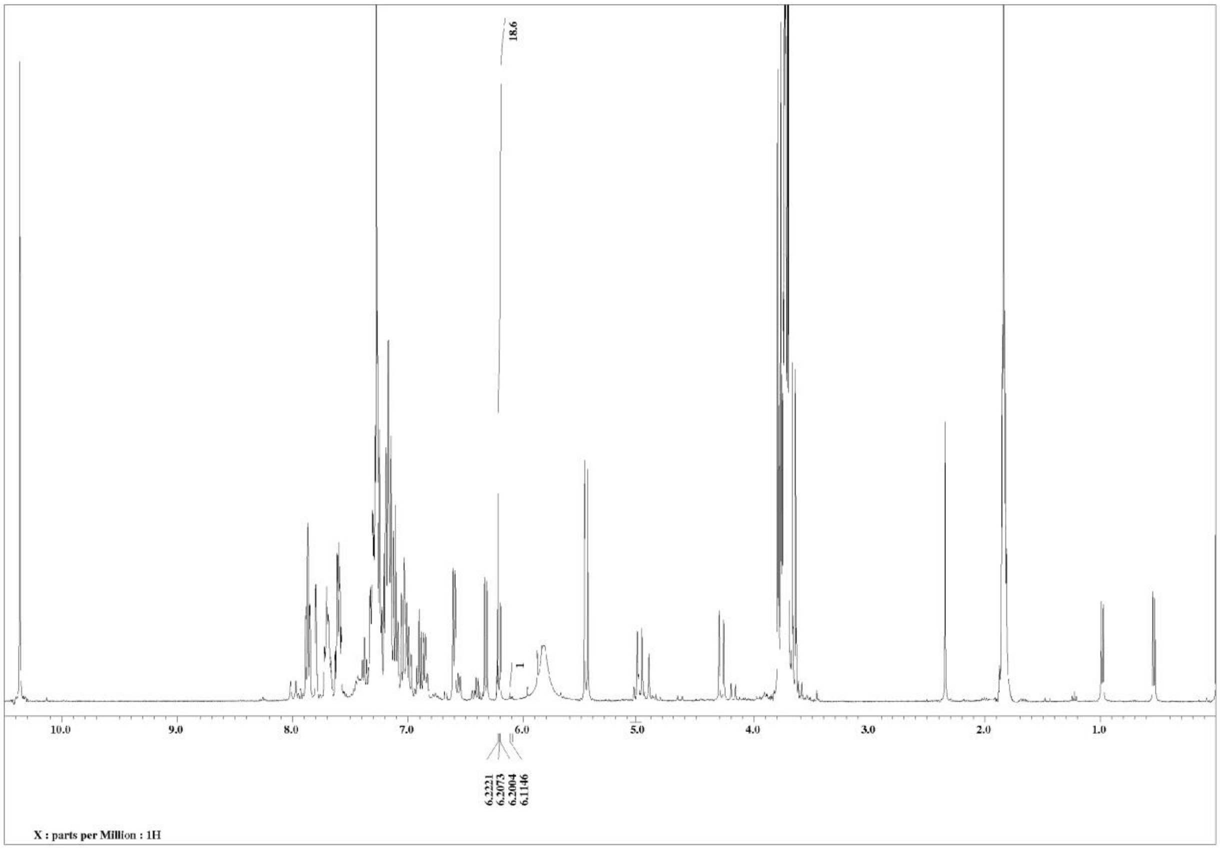
18.6
1
10.0
9.0
8.0
7.0
6.0
5.0
4.0
3.0
2.0
1.0
6.2221
6.2073
6.2004
6.1146
X : parts per Million : 1H

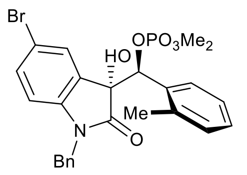
Br
OPO3Me2
HO
Me
N
O
Bn

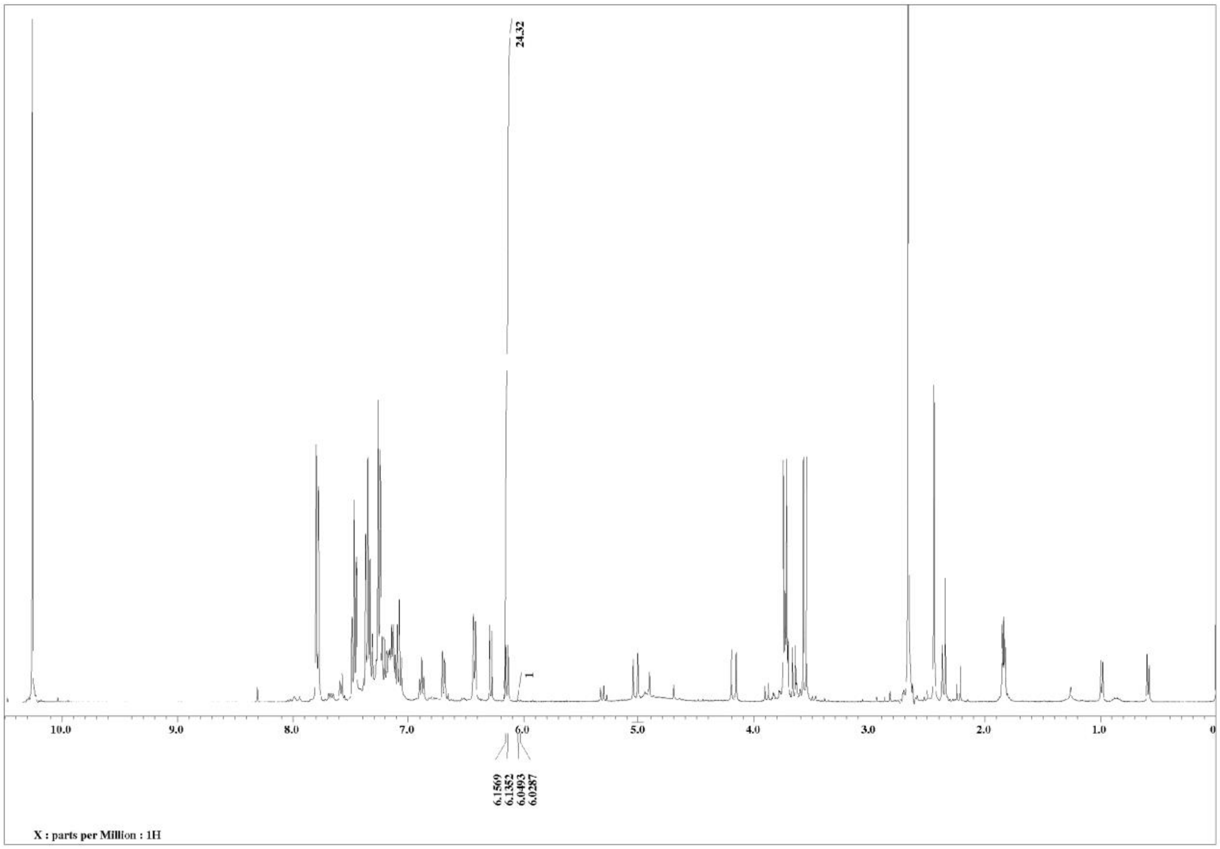
24.32
1
10.0
9.0
8.0
7.0
6.0
5.0
4.0
3.0
2.0
1.0
0
6.1569
6.1352
6.0493
6.0287
X : parts per Million : 1H

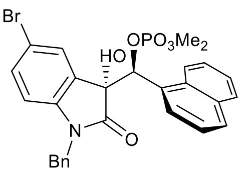
Br
OPO3Me2
HO
N
O
Bn

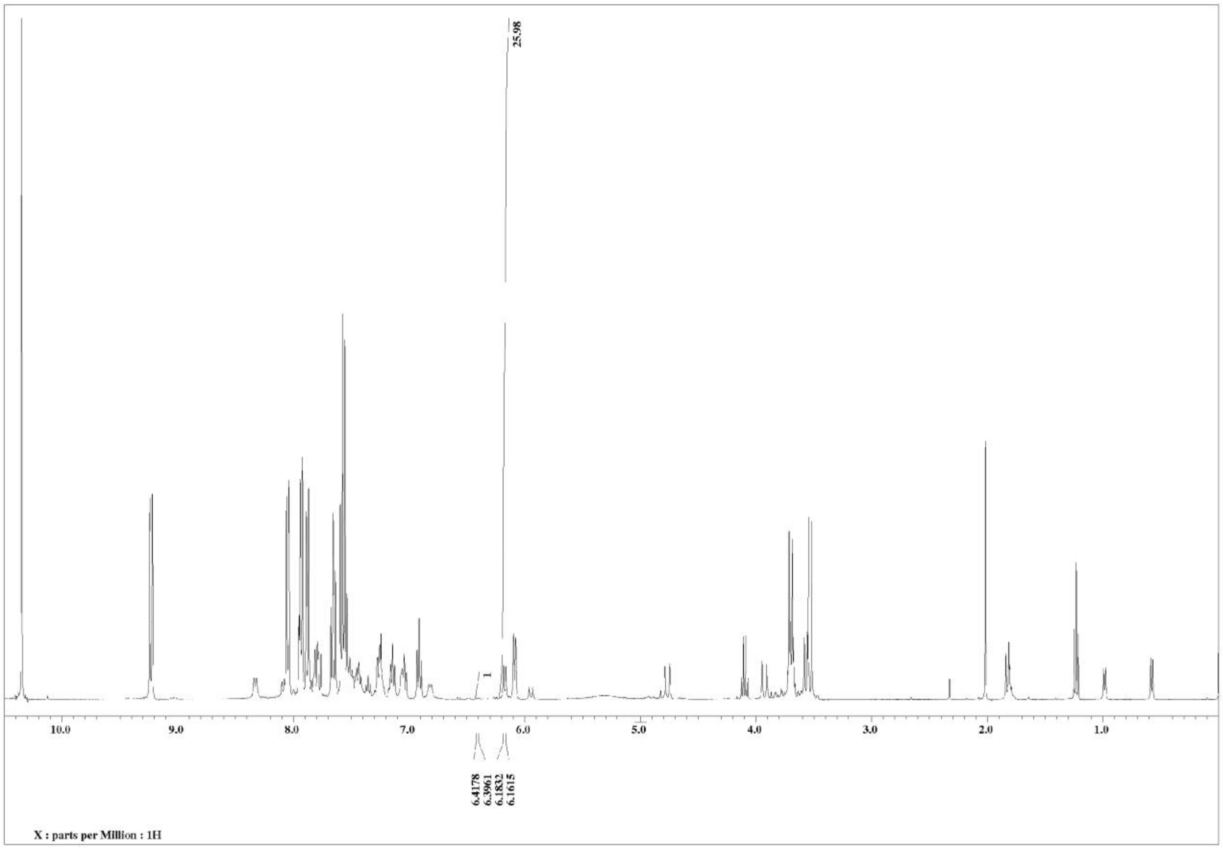
25.98
1
10.0
9.0
8.0
7.0
6.0
5.0
4.0
3.0
2.0
1.0
6.4178
6.3961
6.1832
6.1615
X : parts per Million : 1H

HPLC traces:

4a:

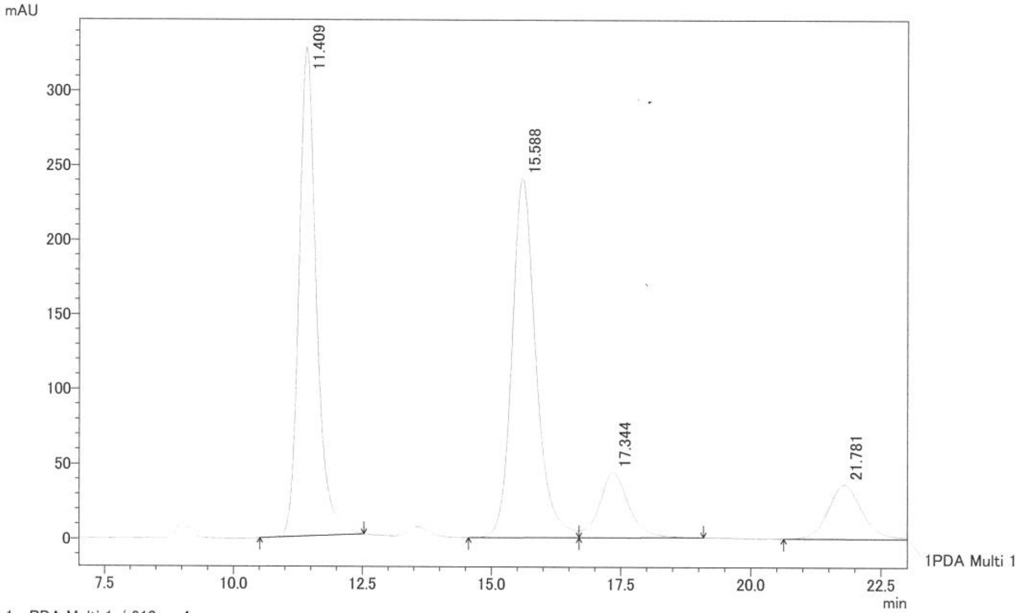

1 PDA Multi 1 / 210nm 4nm

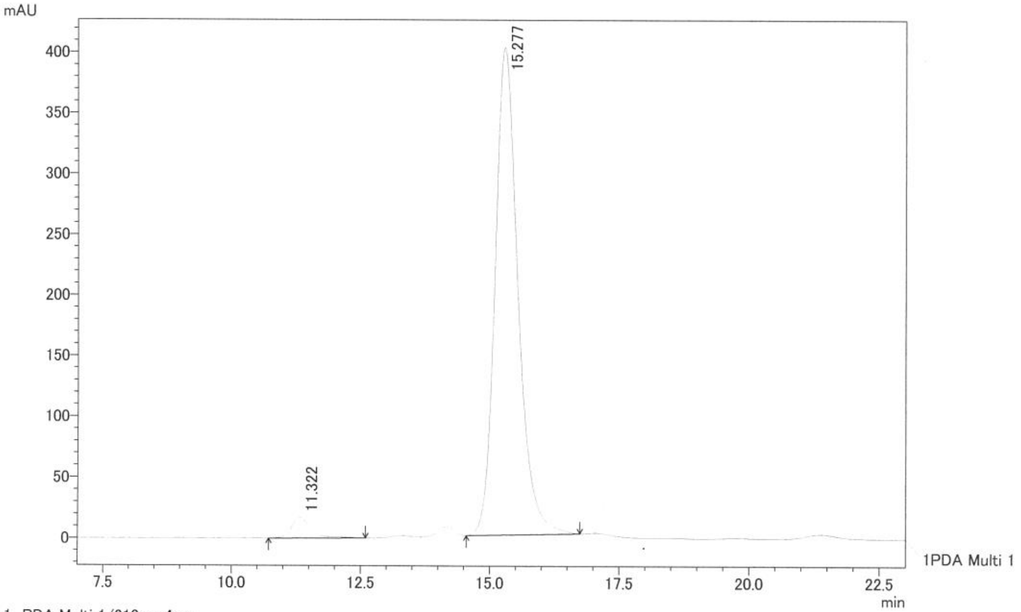

1 PDA Multi 1/210nm 4nm

PDA Ch1 210nm 4nm

ピーク#	保持時間	面積%
1	11.322	3.367
2	15.277	96.633
合計		100.000

4h:

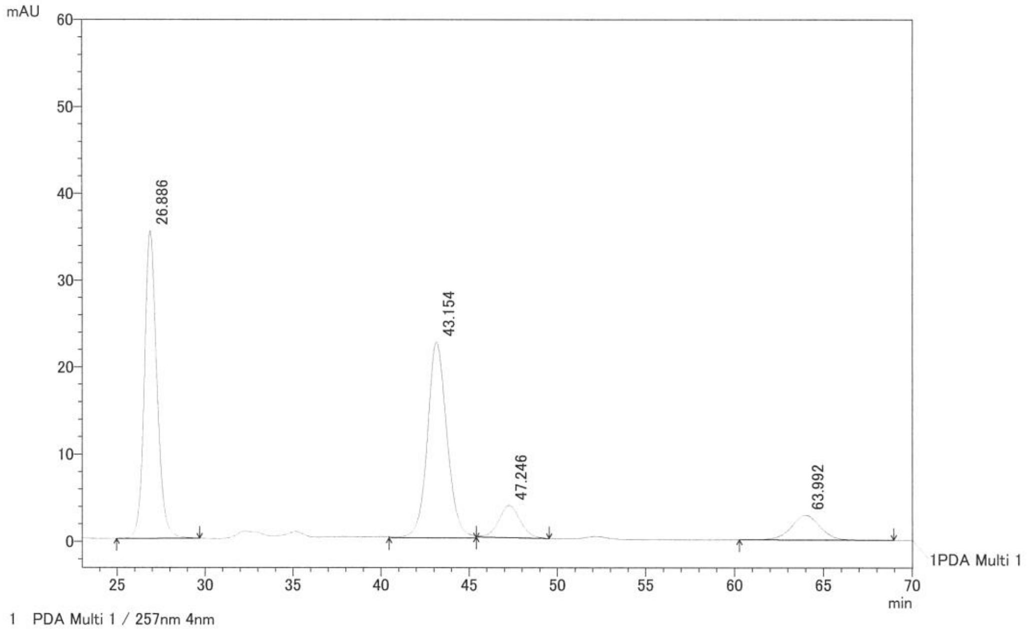

1 PDA Multi 1 / 257nm 4nm

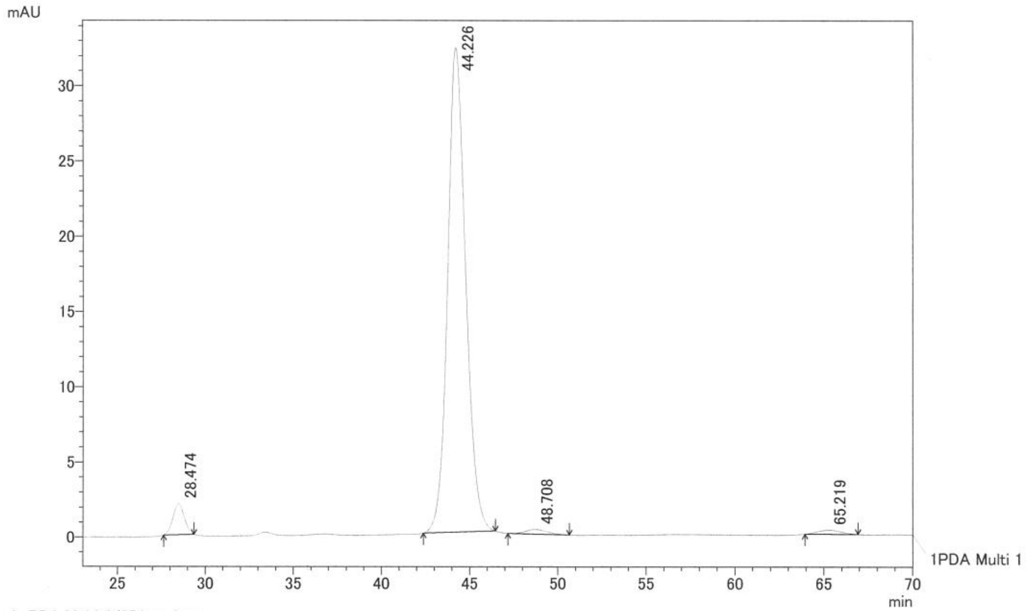

1 PDA Multi 1/254nm 4nm

PDA Ch1 254nm 4nm

ピーク#	保持時間	面積%
1	28.474	3.805
2	44.226	93.986
3	48.708	1.137
4	65.219	1.073
合計		100.000

4i:

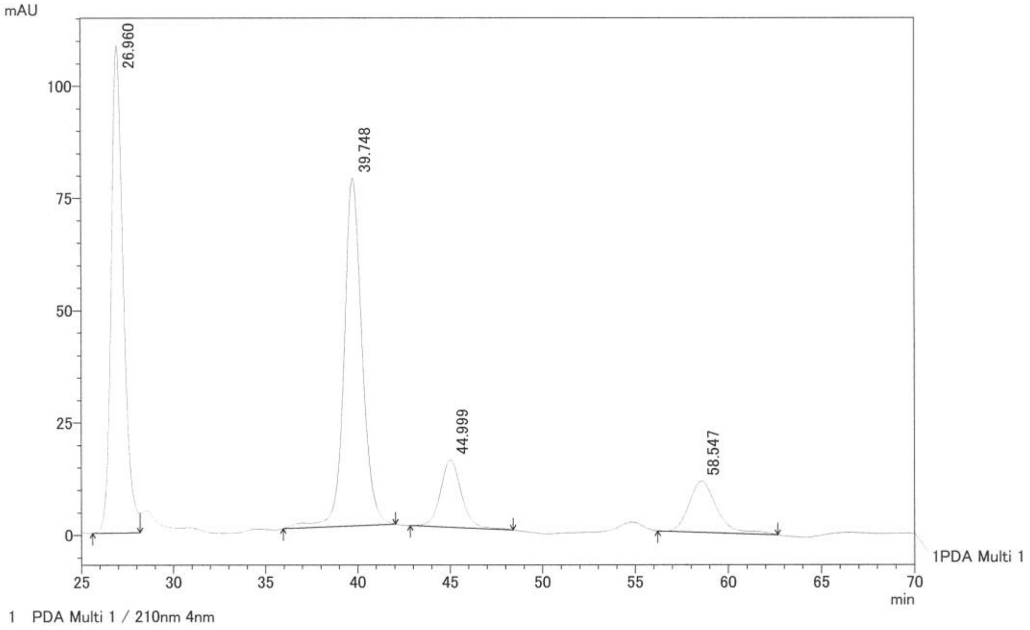

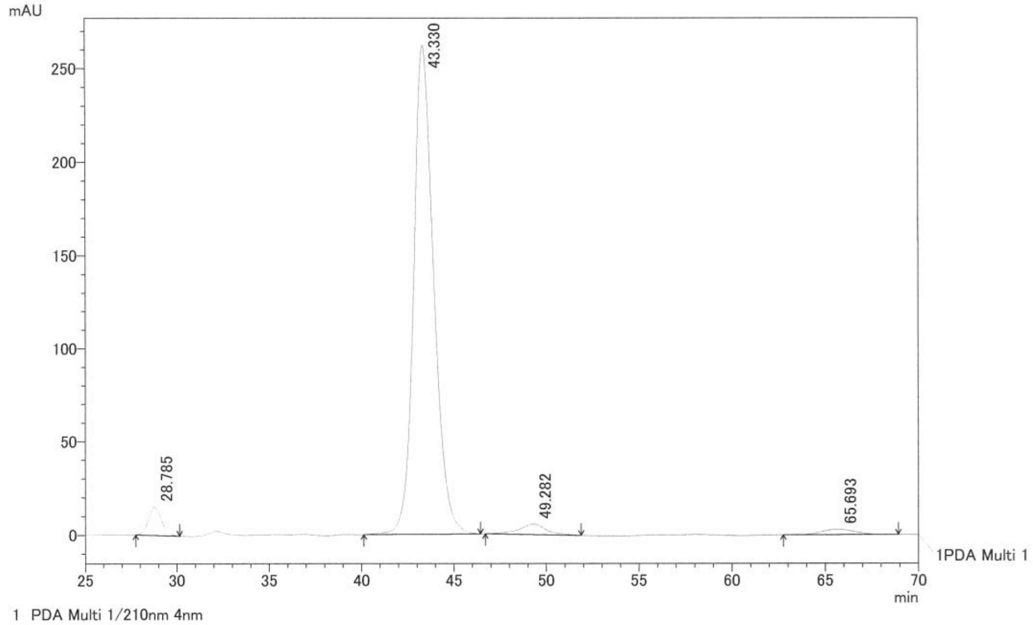

PDA Ch1 210nm 4nm

ピーク#	保持時間	面積%
1	28.785	3.481
2	43.330	92.263
3	49.282	2.722
4	65.693	1.534
合計		100.000

4j:

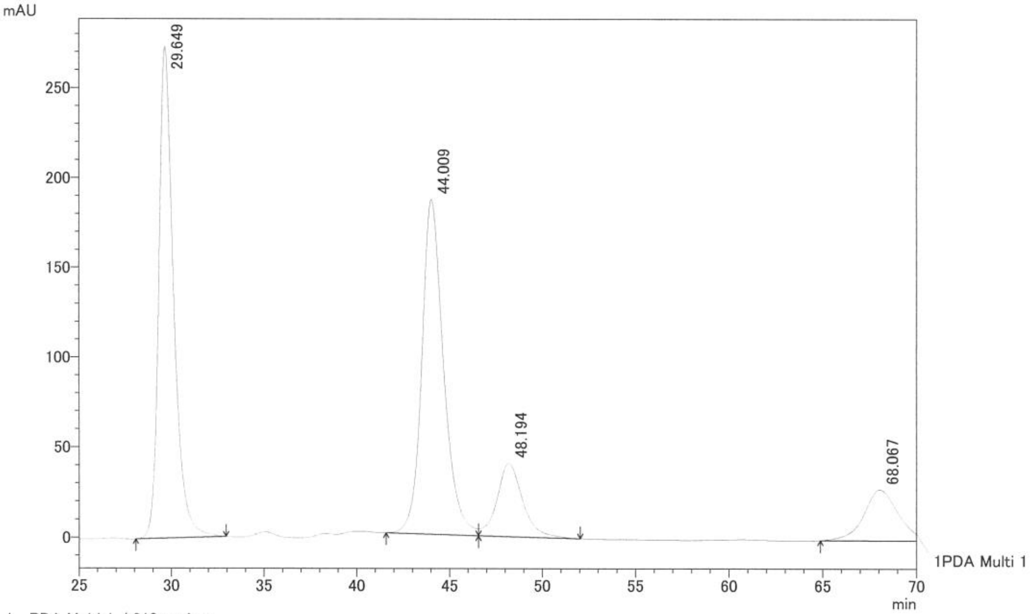

1 PDA Multi 1 / 210nm 4nm

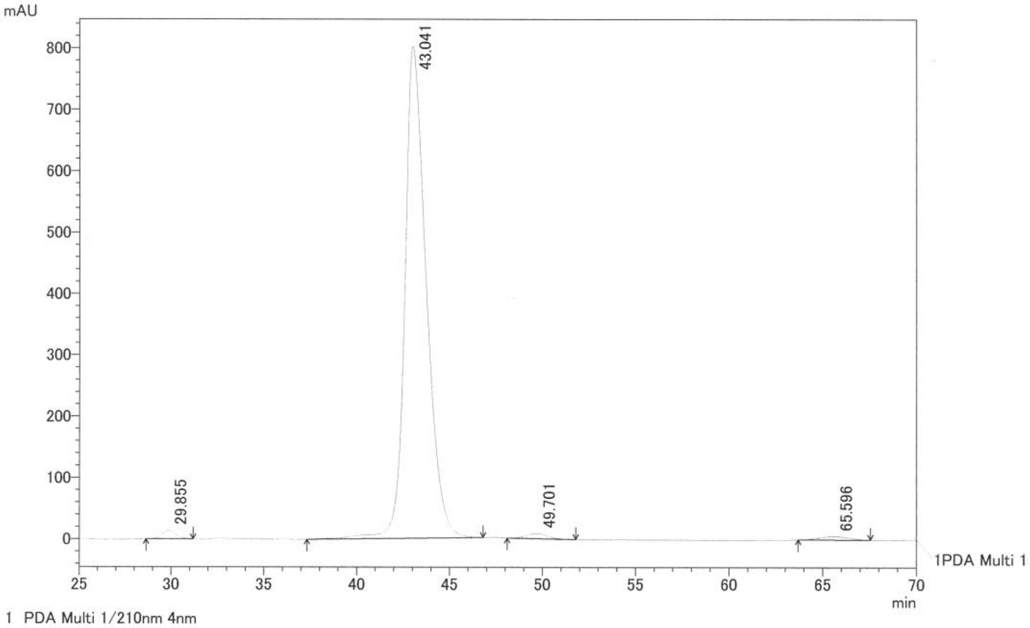

1 PDA Multi 1/210nm 4nm

PDA Ch1 210nm 4nm

ピーク#	保持時間	面積%
1	29.855	1.065
2	43.041	97.073
3	49.701	0.992
4	65.596	0.870
合計		100.000

4l:

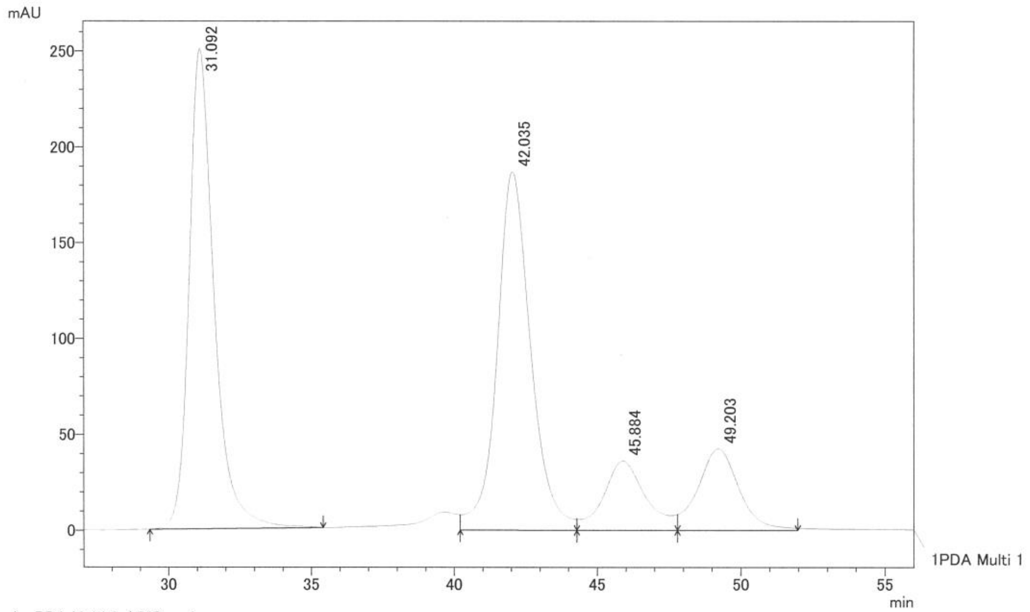

1 PDA Multi 1 / 210nm 4nm

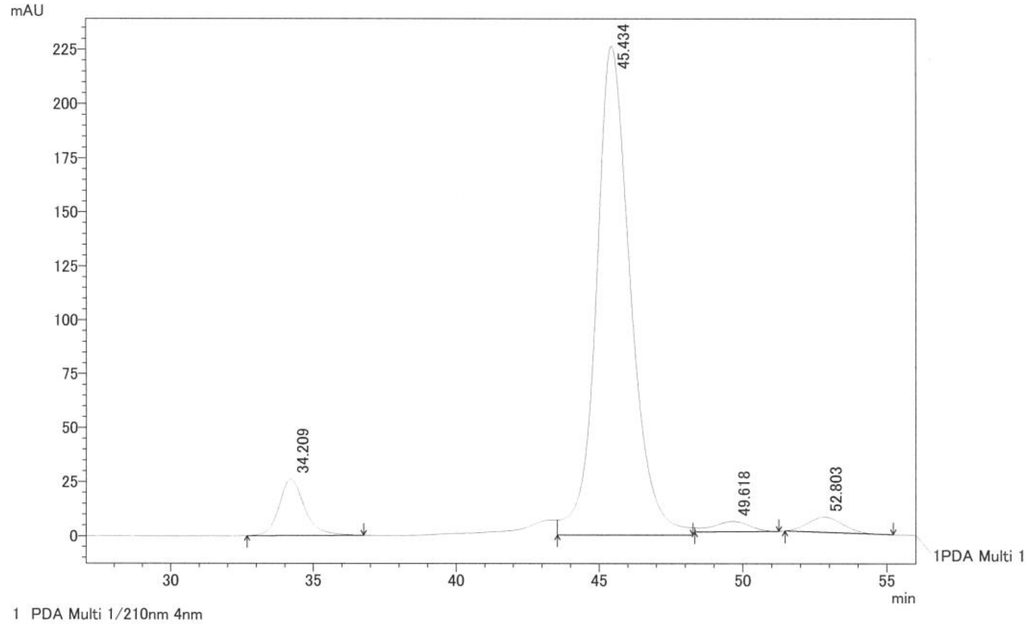

1 PDA Multi 1/210nm 4nm

PDA Ch1 210nm 4nm

ピーク#	保持時間	面積%
1	34.209	7.624
2	45.434	87.663
3	49.618	1.962
4	52.803	2.752
合計		100.000

4m:

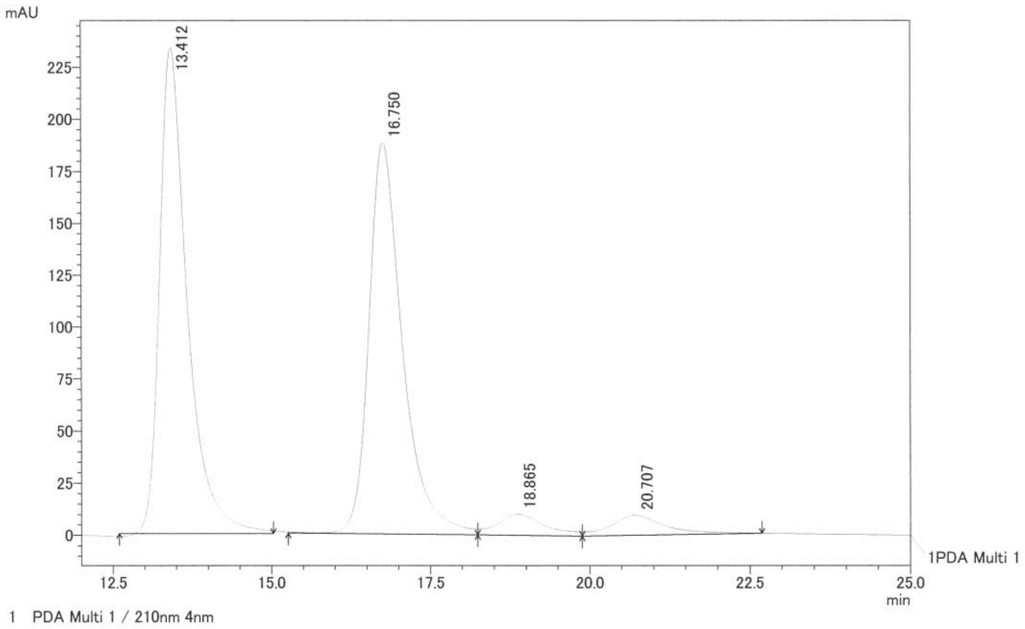

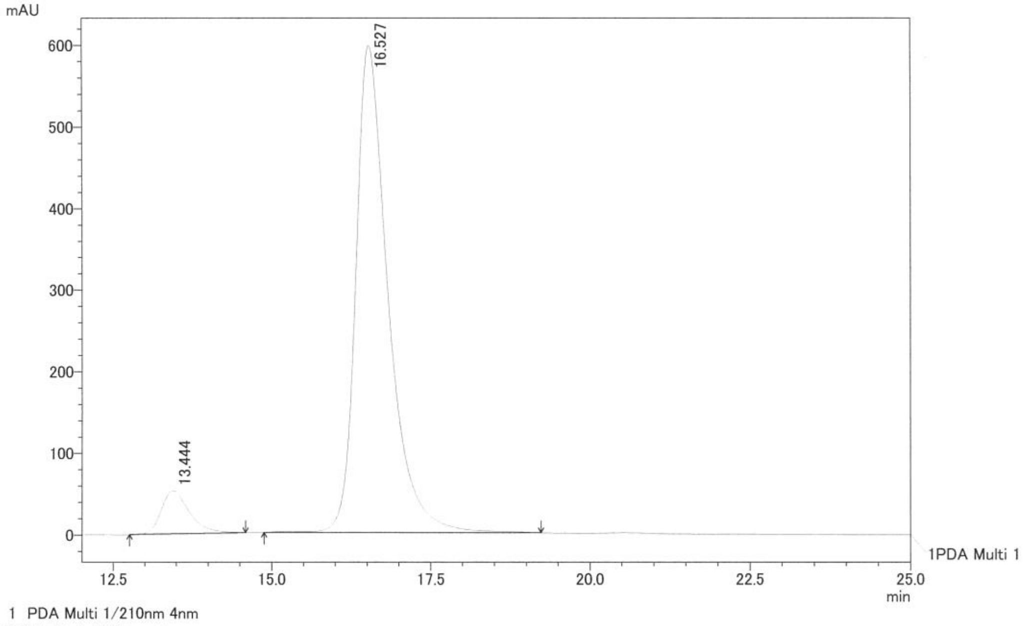

PDA Ch1 210nm 4nm

ピーク#	保持時間	面積%
1	13.444	7.057
2	16.527	92.943
合計		100.000

4n:

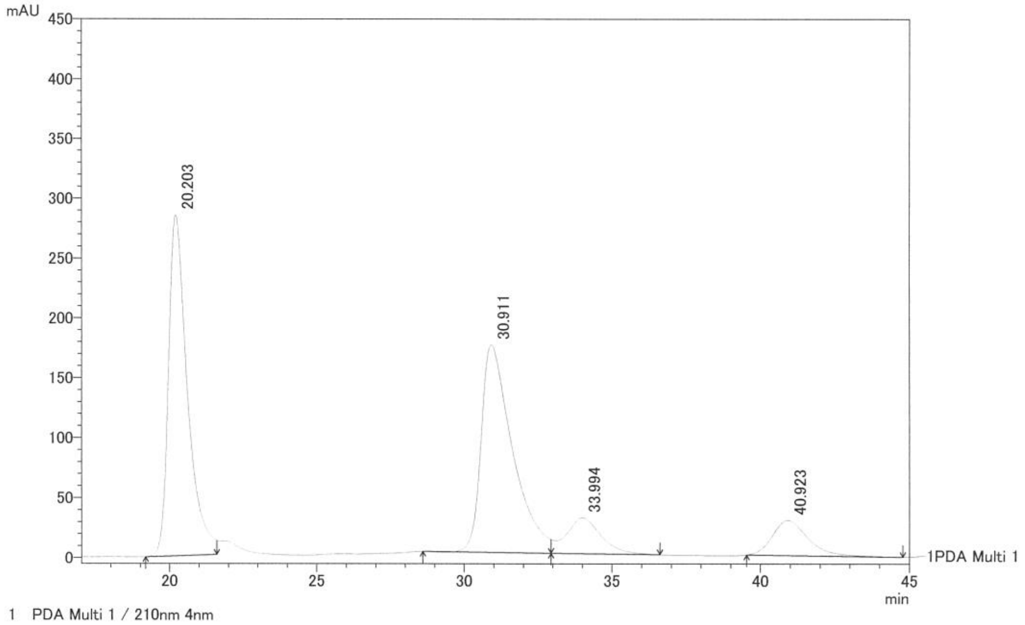

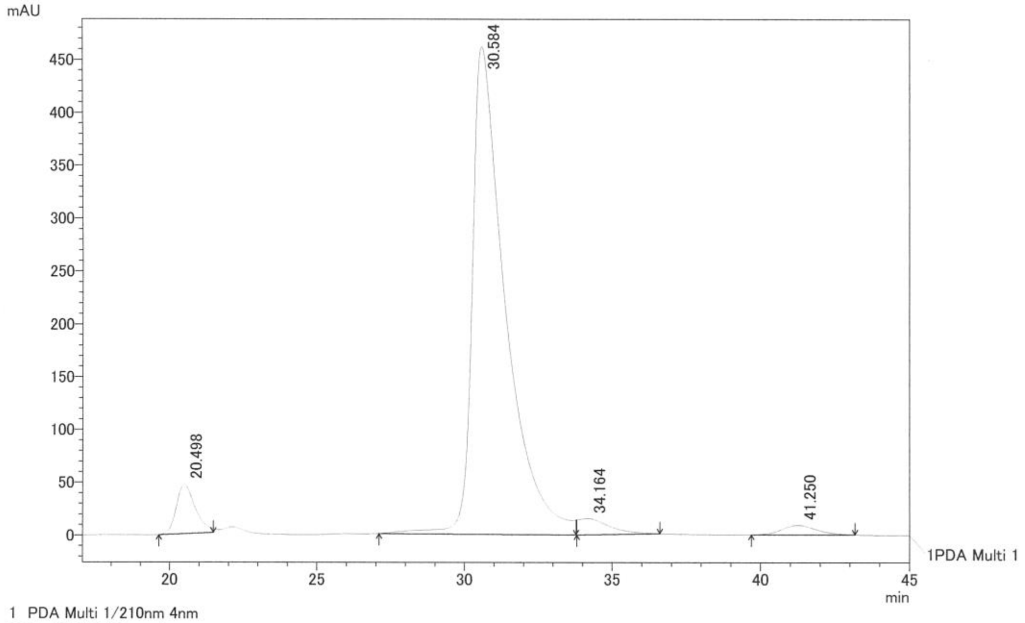

PDA Ch1 210nm 4nm

ピーク#	保持時間	面積%
1	20.498	5.244
2	30.584	90.112
3	34.164	2.821
4	41.250	1.823
合計		100.000

4o:

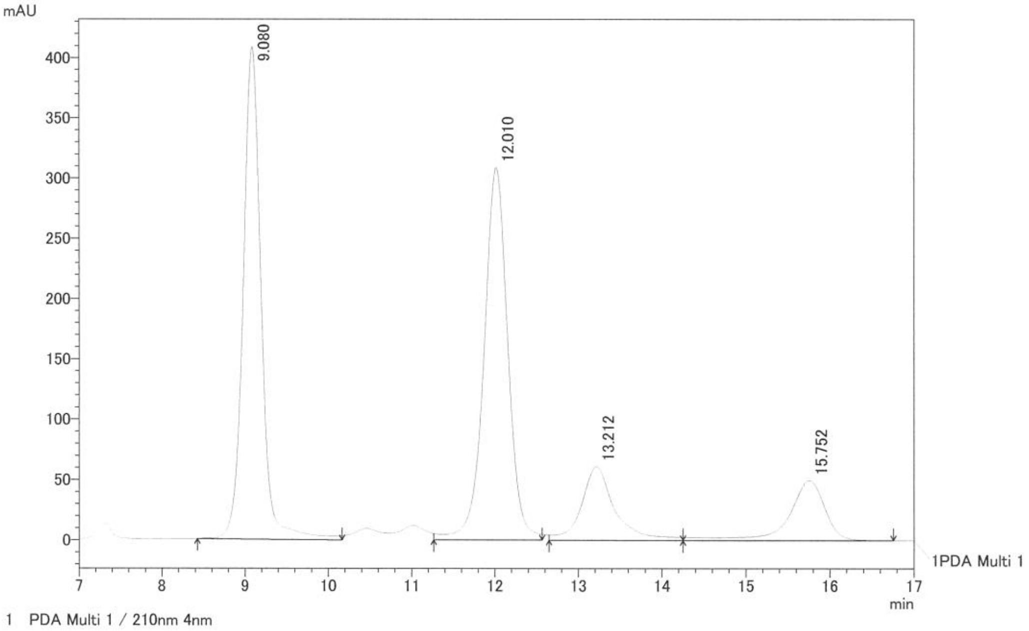

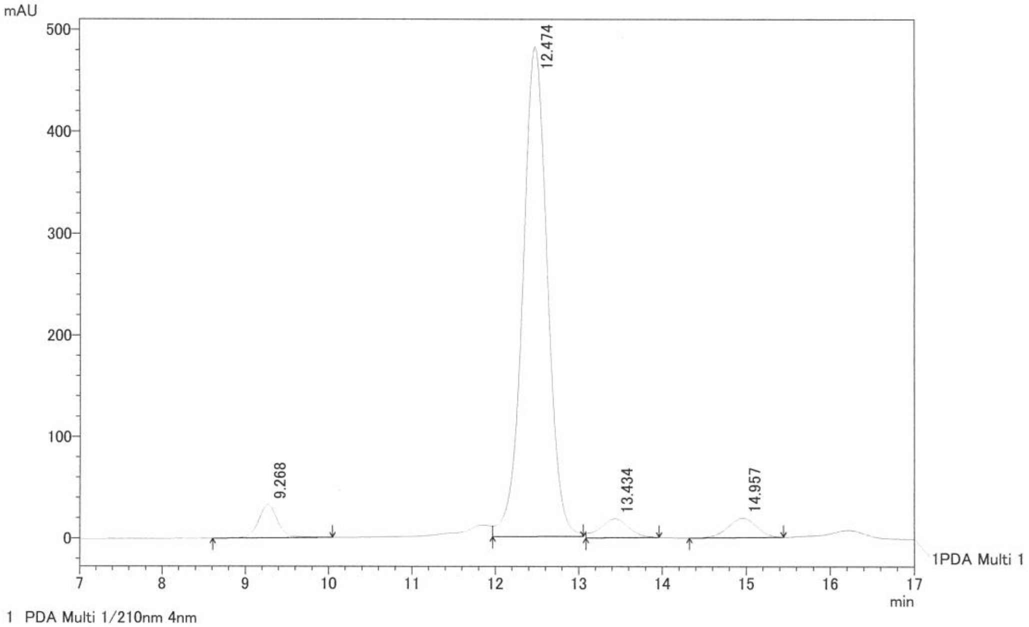

PDA Ch1 210nm 4nm

ピーク#	保持時間	面積%
1	9.268	4.674
2	12.474	87.350
3	13.434	3.919
4	14.957	4.057
合計		100.000

4p:

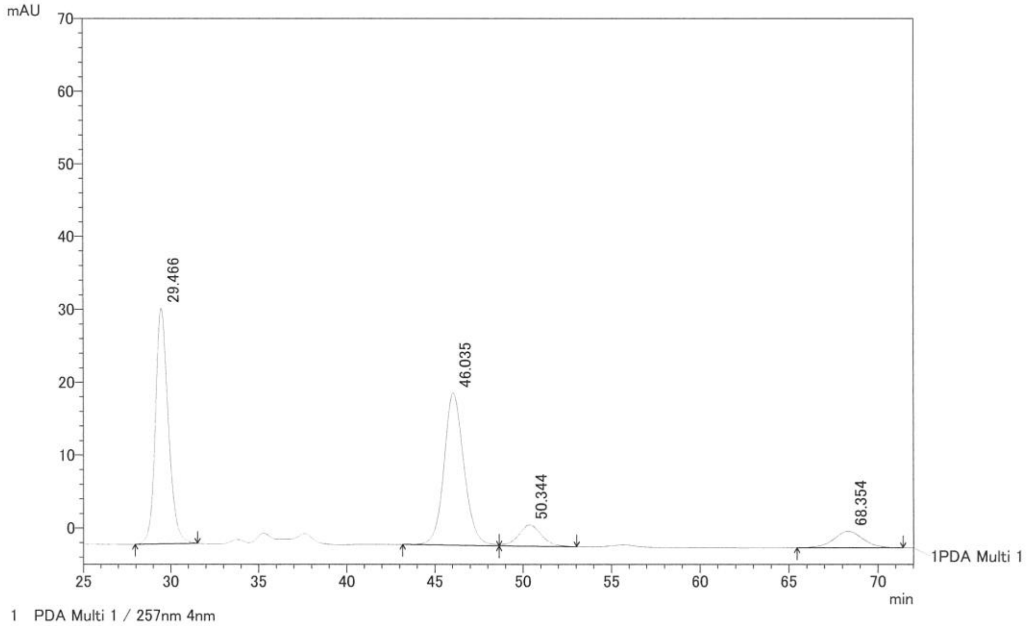

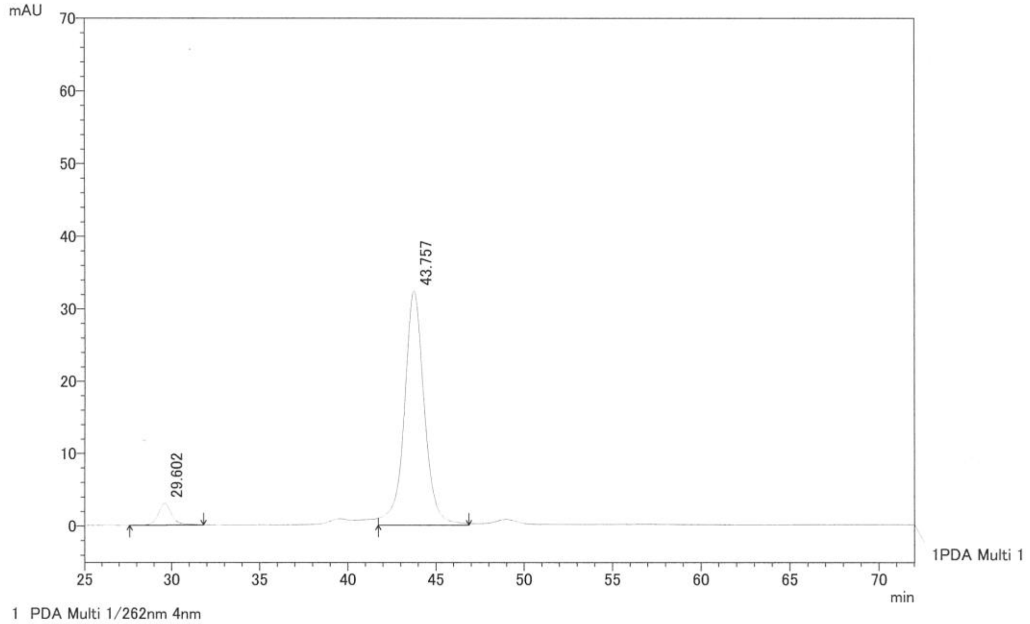

PDA Ch1 262nm 4nm

ピーク#	保持時間	面積%
1	29.602	5.873
2	43.757	94.127
合計		100.000

4q:

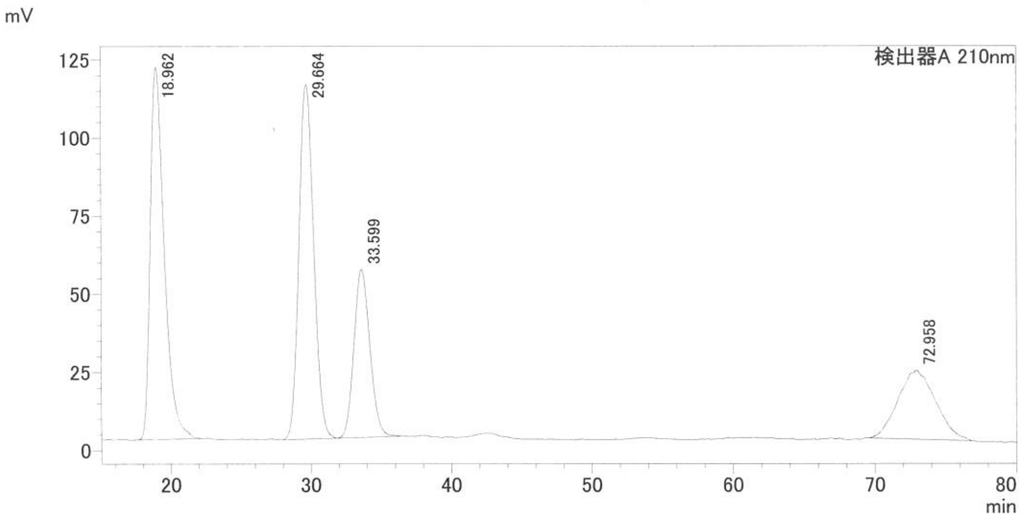

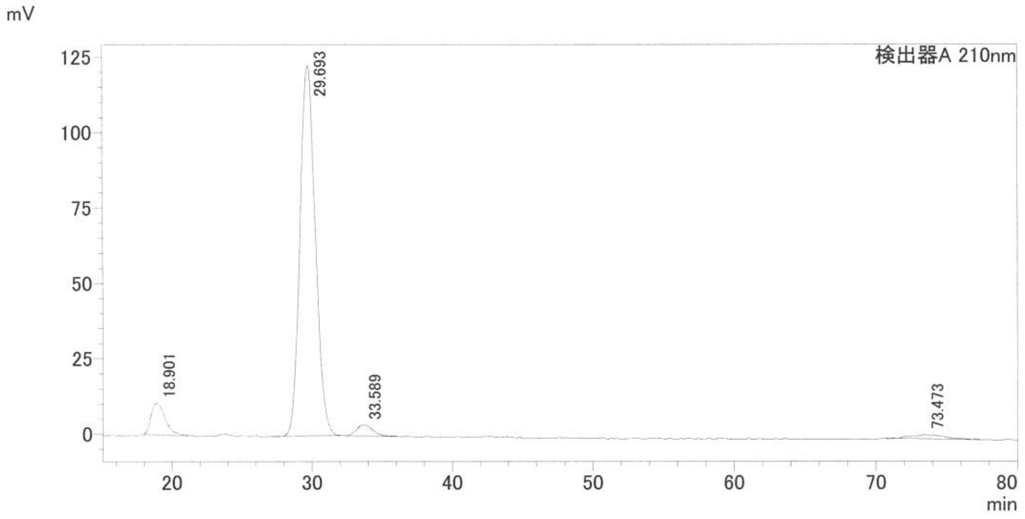

検出器A 210nm

ピーク#	保持時間	高さ	面積	濃度
1	18.901	10403	742794	6.985
2	29.693	122705	9370729	88.115
3	33.589	3604	296383	2.787
4	73.473	1450	224722	2.113
合計		138162	10634629	

4r:

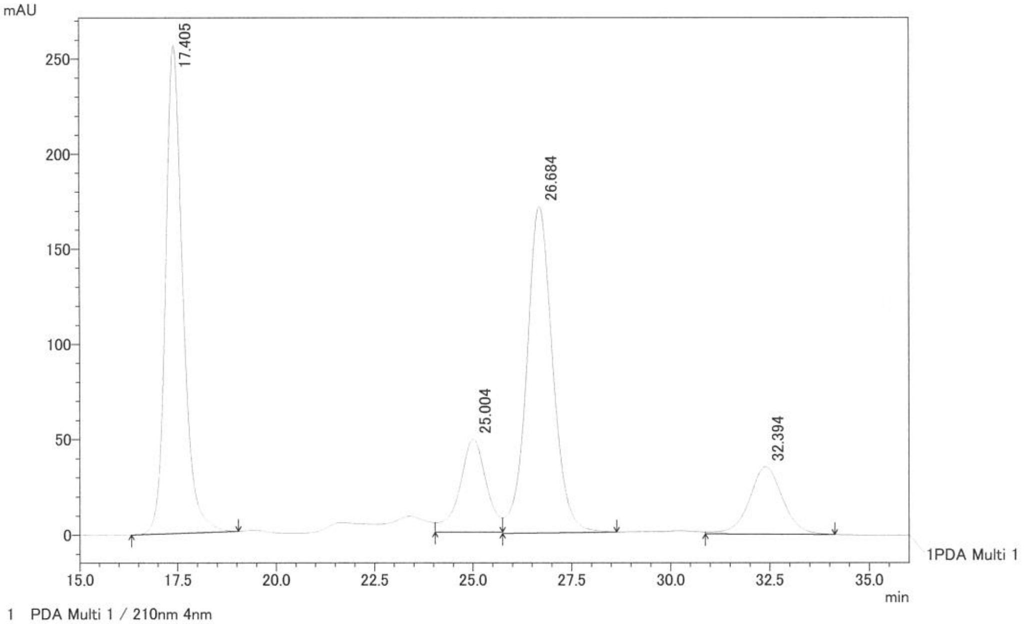

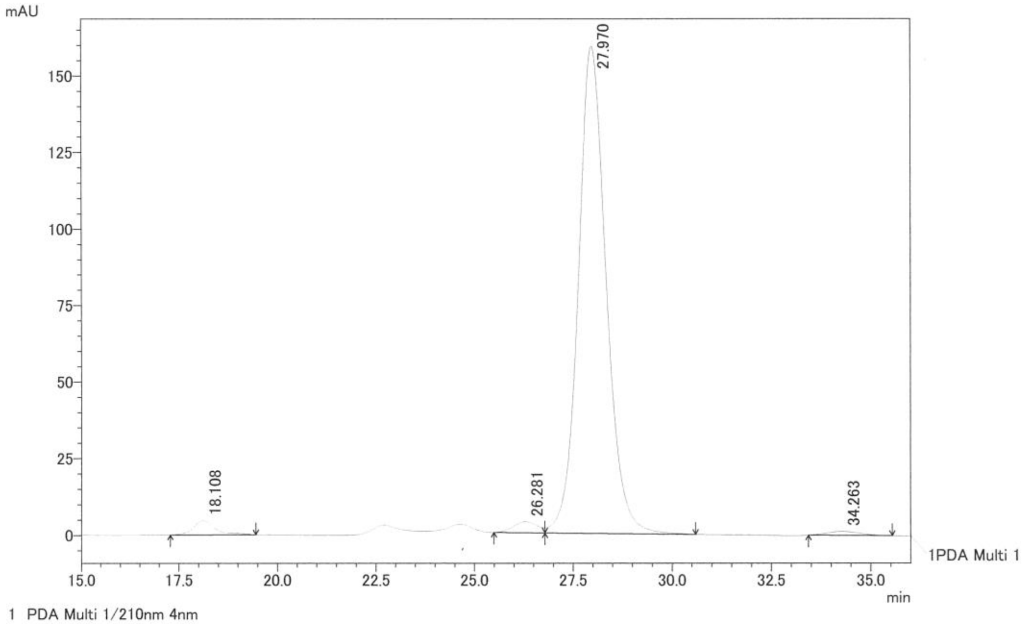

PDA Ch1 210nm 4nm

ピーク#	保持時間	面積%
1	18.108	2.188
2	26.281	1.857
3	27.970	95.035
4	34.263	0.919
合計		100.000

4s:

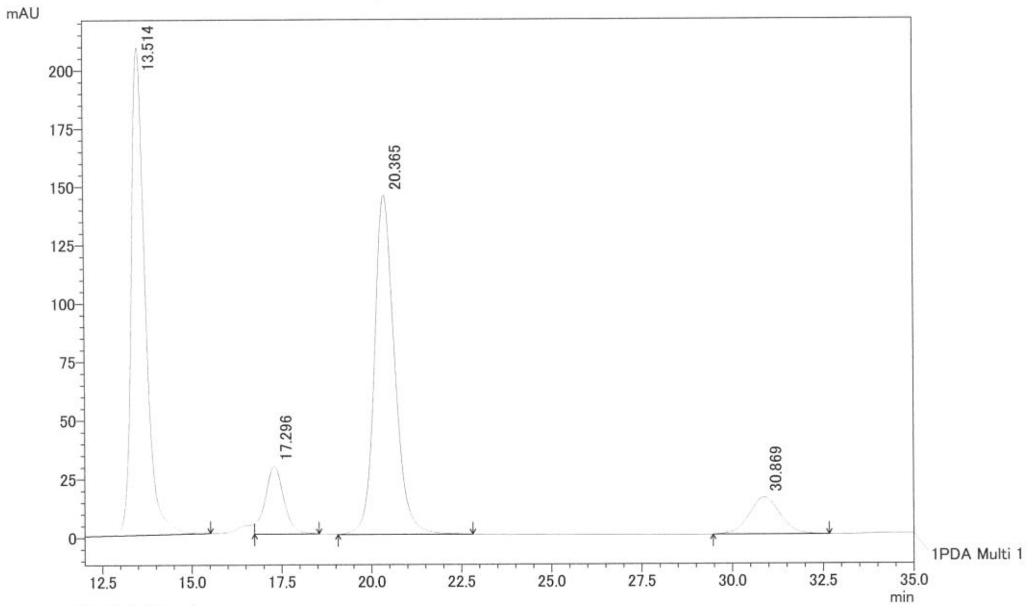

1 PDA Multi 1 / 210nm 4nm

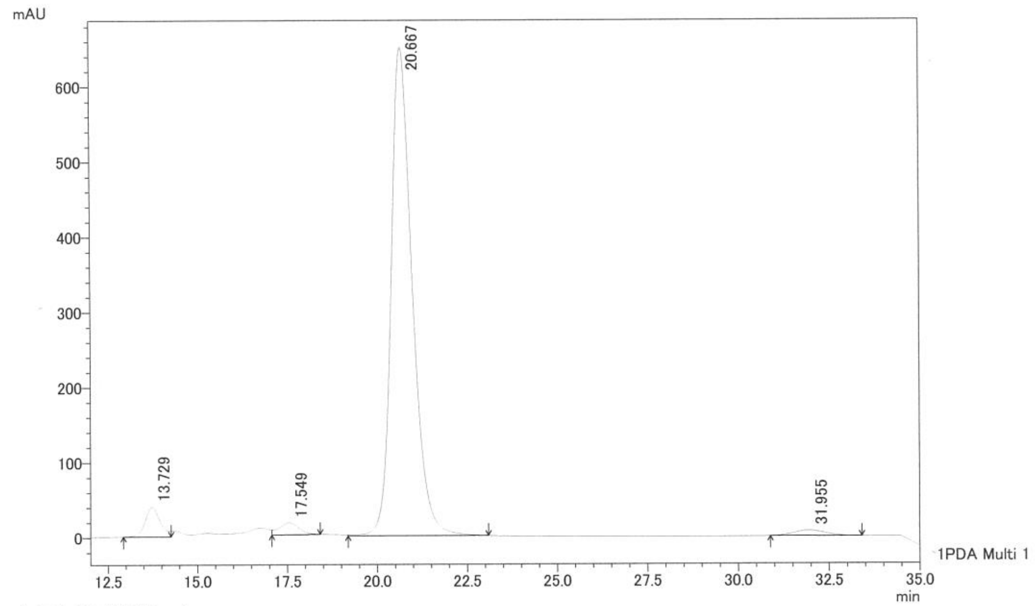

1 PDA Multi 1/210nm 4nm

PDA Ch1 210nm 4nm

ピーク#	保持時間	面積%
1	13.729	4.091
2	17.549	2.178
3	20.667	92.169
4	31.955	1.562
合計		100.000

4t:

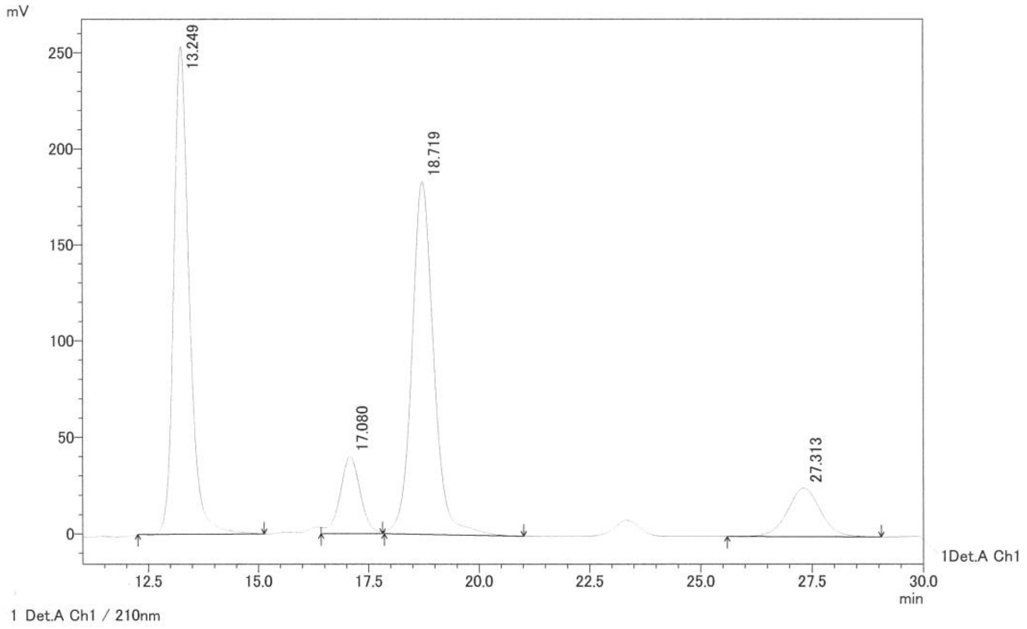

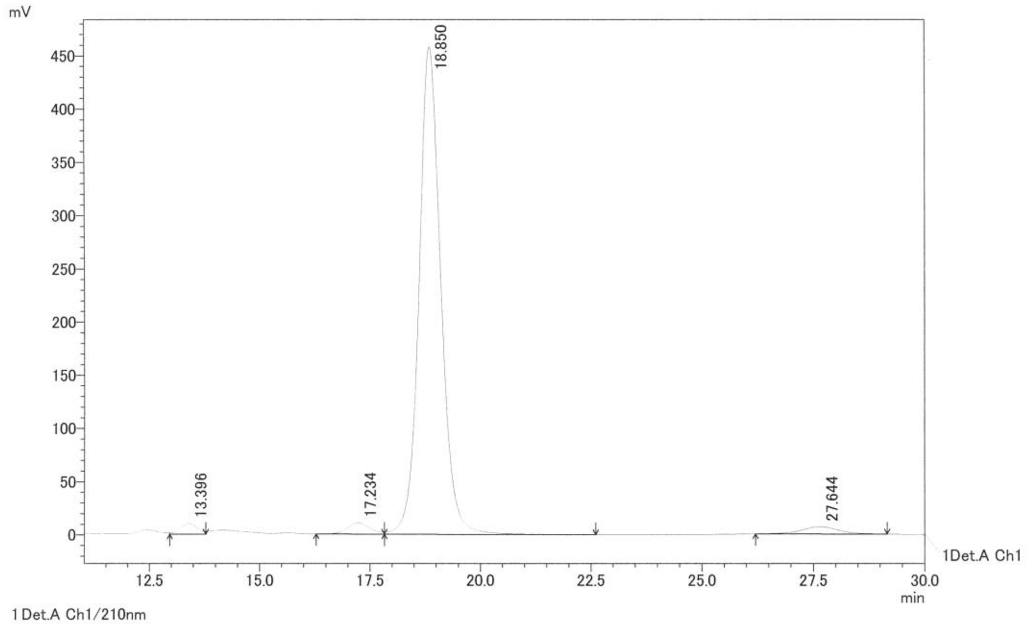

1 Det.A Ch1/210nm

検出器A Ch1 210nm

ピーク#	保持時間	面積%
1	13.396	1.564
2	17.234	2.240
3	18.850	93.888
4	27.644	2.308
合計		100.000

4u:

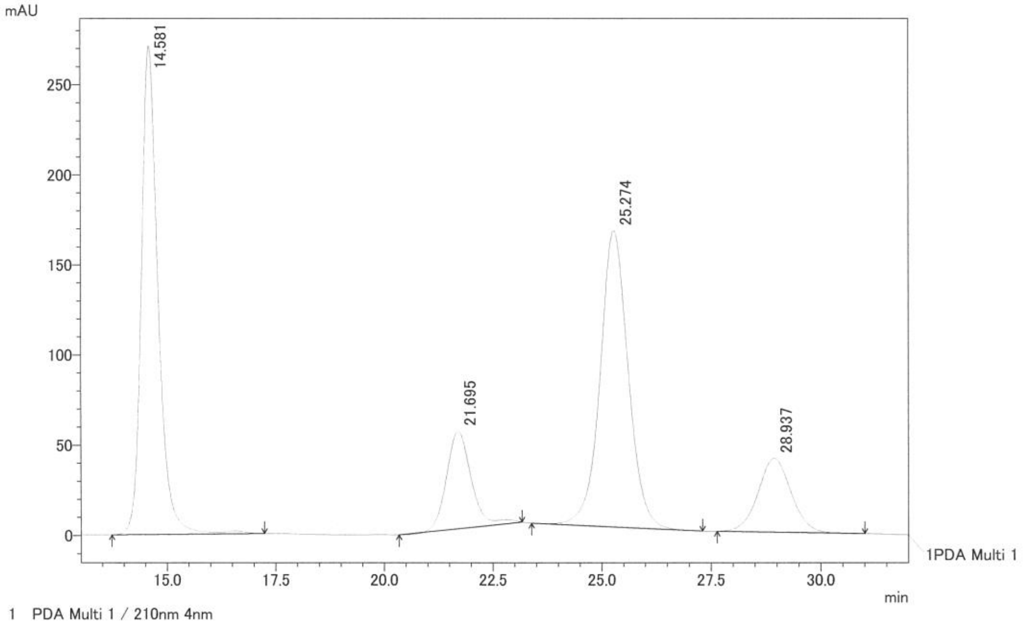

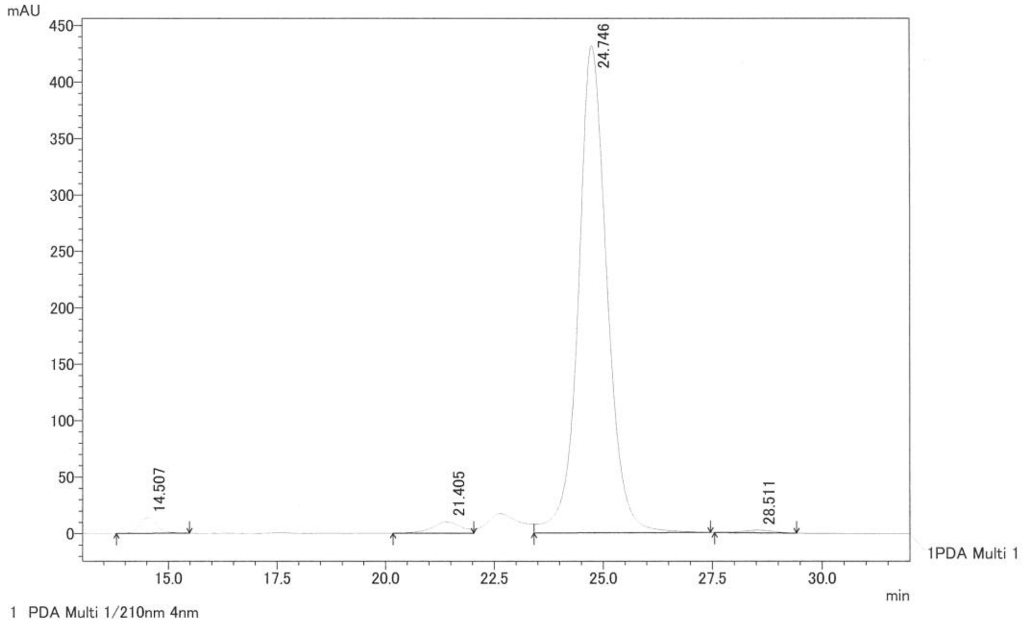

PDA Ch1 210nm 4nm

ピーク#	保持時間	面積%
1	14.507	1.834
2	21.405	2.325
3	24.746	95.291
4	28.511	0.550
合計		100.000

4v:

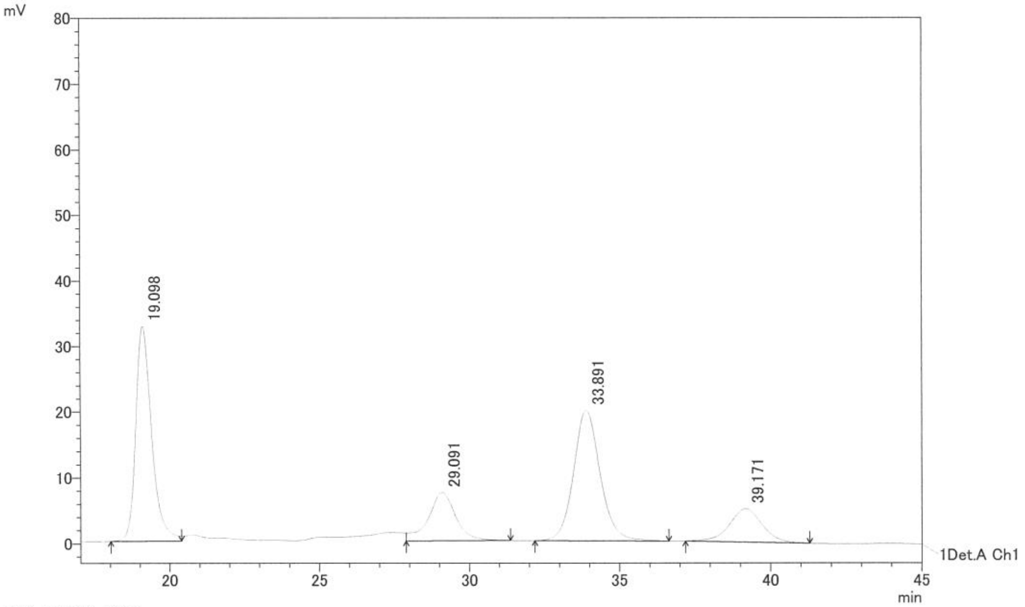

1 Det.A Ch1 / 254nm

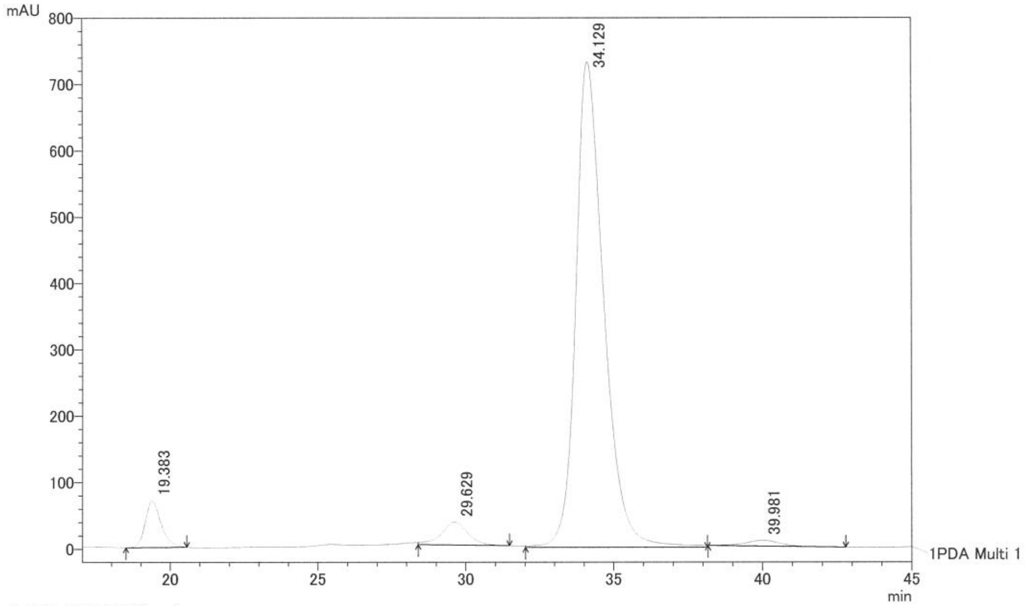

1 PDA Multi 1/210nm 4nm

PDA Ch1 210nm 4nm

ピーク#	保持時間	面積%
1	19.383	4.937
2	29.629	4.069
3	34.129	89.739
4	39.981	1.255
合計		100.000

4w:

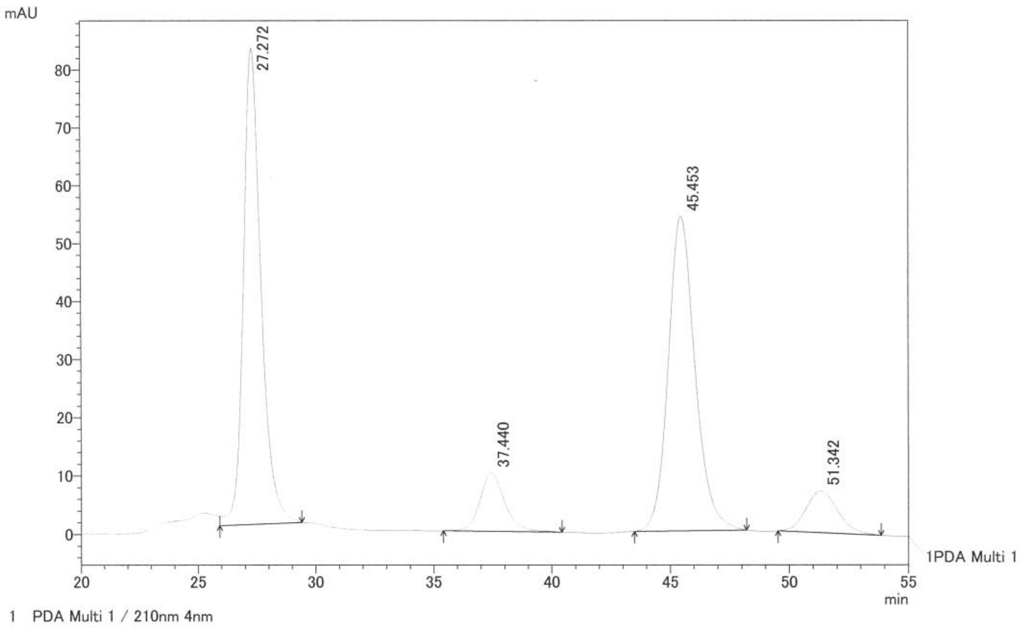

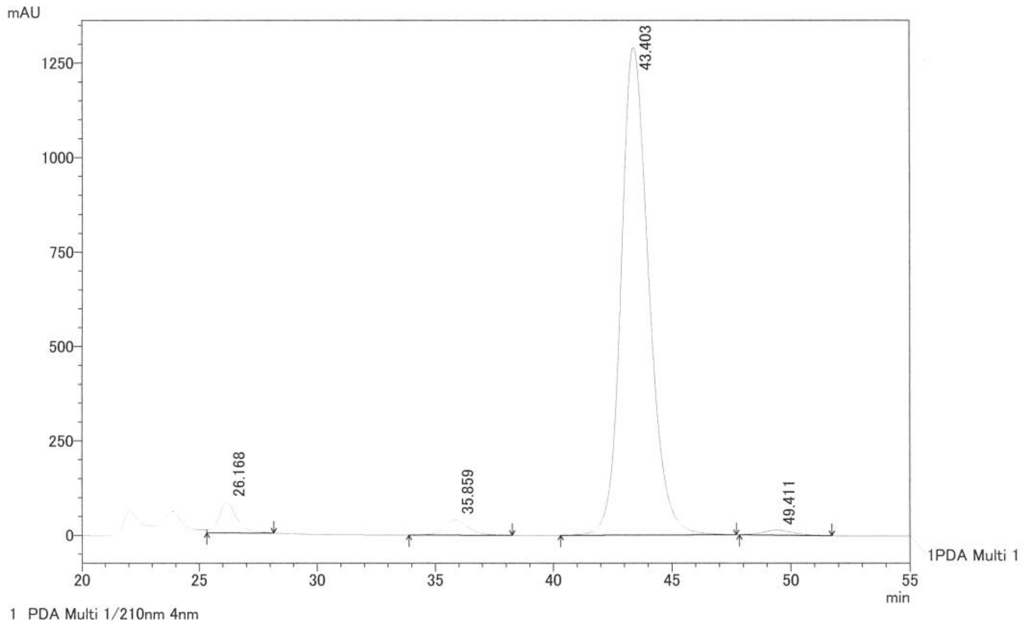

PDA Ch1 210nm 4nm

ピーク#	保持時間	面積%
1	26.168	3.803
2	35.859	2.531
3	43.403	92.625
4	49.411	1.040
合計		100.000

4x:

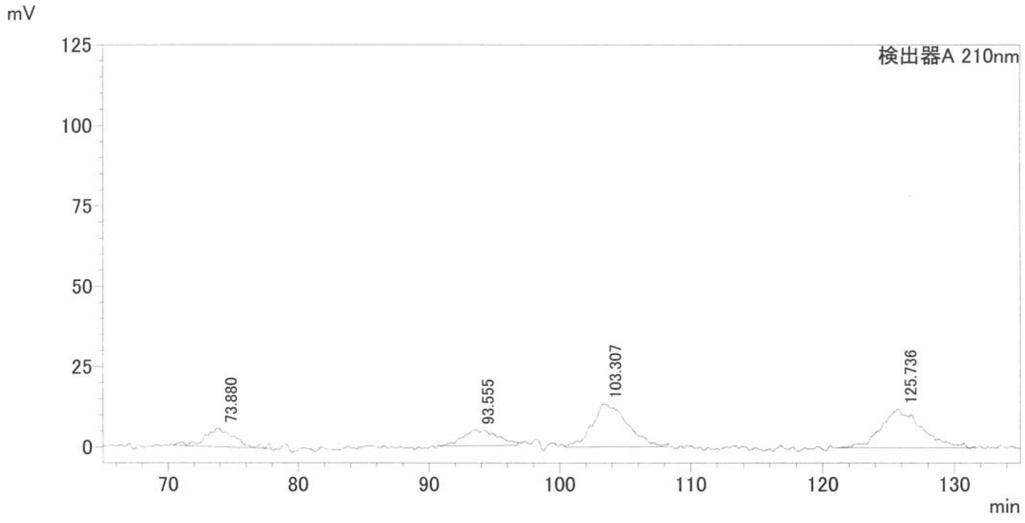

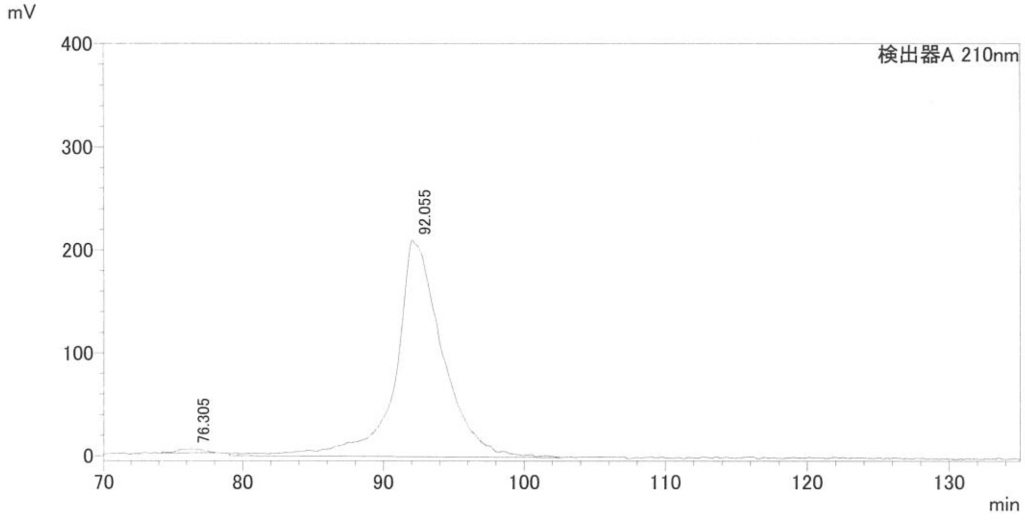

検出器A 210nm

ﾋﾟｰｸ#	保持時間	高さ	面積
1	76.305	3882	485233
2	92.055	210105	49009898
合計		213987	49495132

4y:

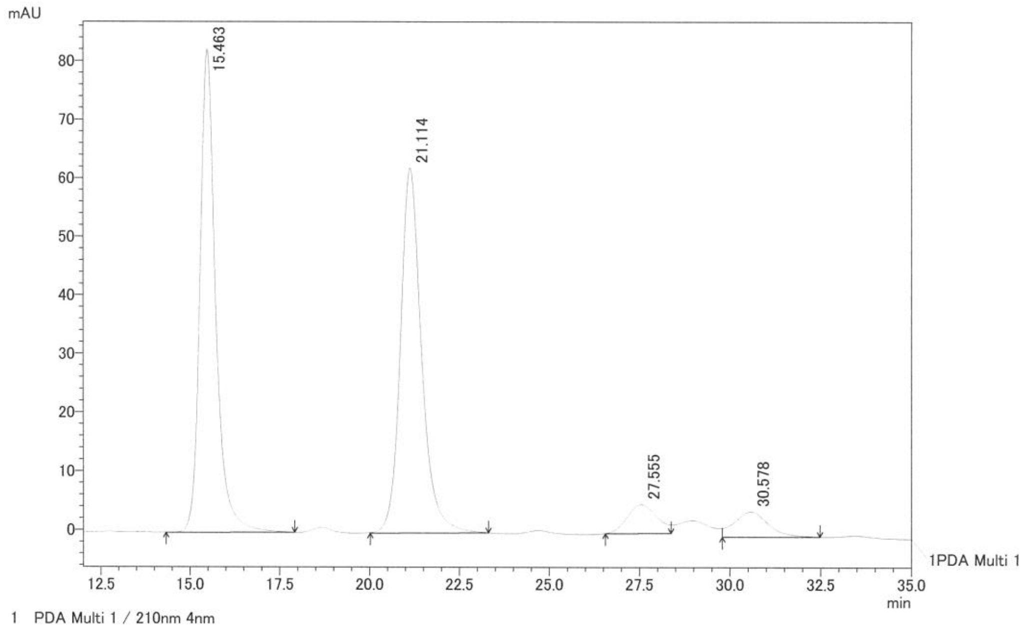

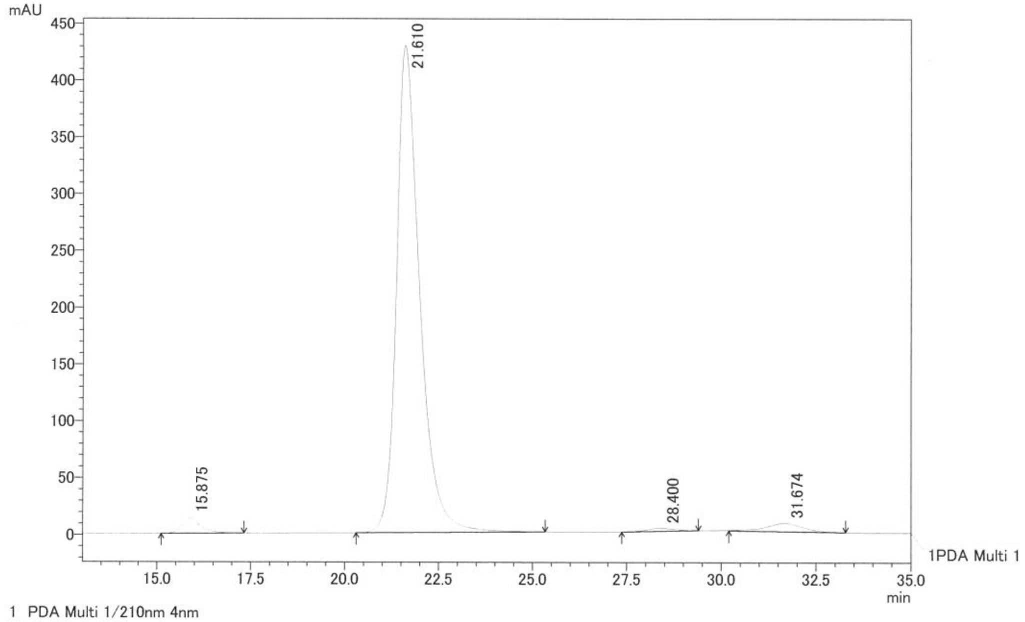

PDA Ch1 210nm 4nm

ピーク#	保持時間	面積%
1	15.875	2.428
2	21.610	94.194
3	28.400	0.708
4	31.674	2.670
合計		100.000

4z:

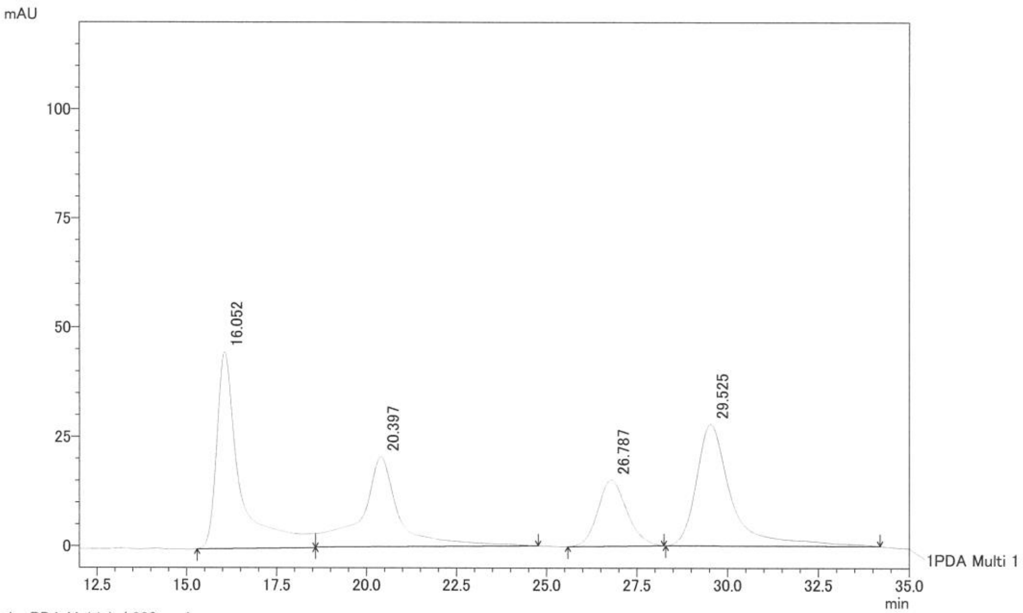

1 PDA Multi 1 / 220nm 4nm

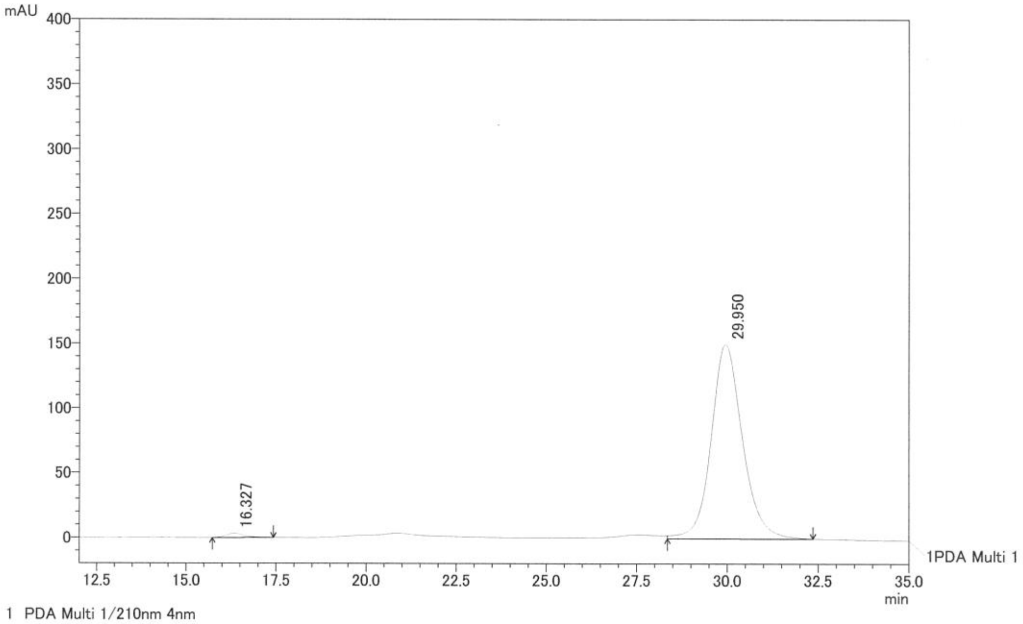

1 PDA Multi 1/210nm 4nm

PDA Ch1 210nm 4nm

ピーク#	保持時間	面積%
1	16.327	1.244
2	29.950	98.756
合計		100.000

Copies of ^{1}H and ^{13}C NMR:

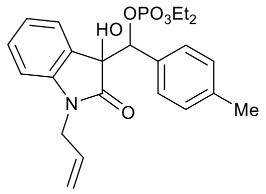

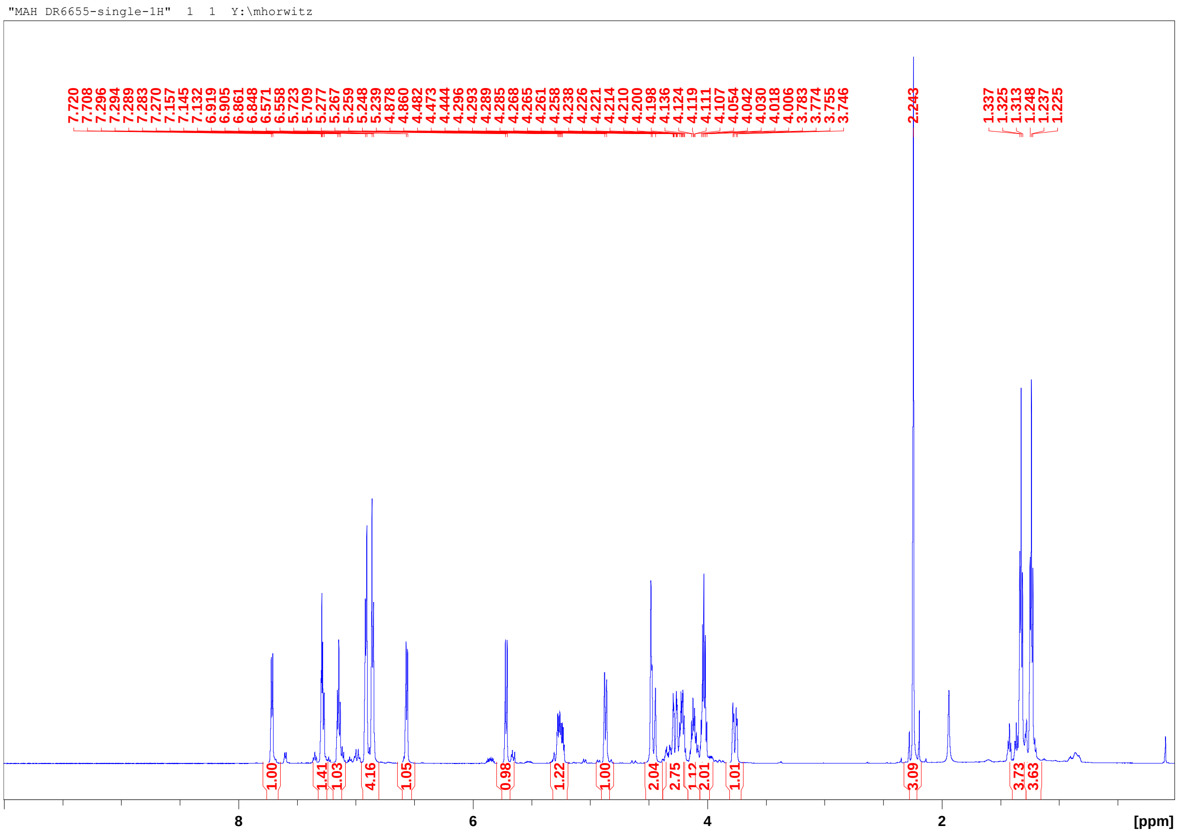

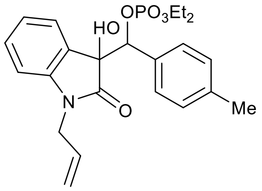
HO
OPO3Et2
N
O
Me

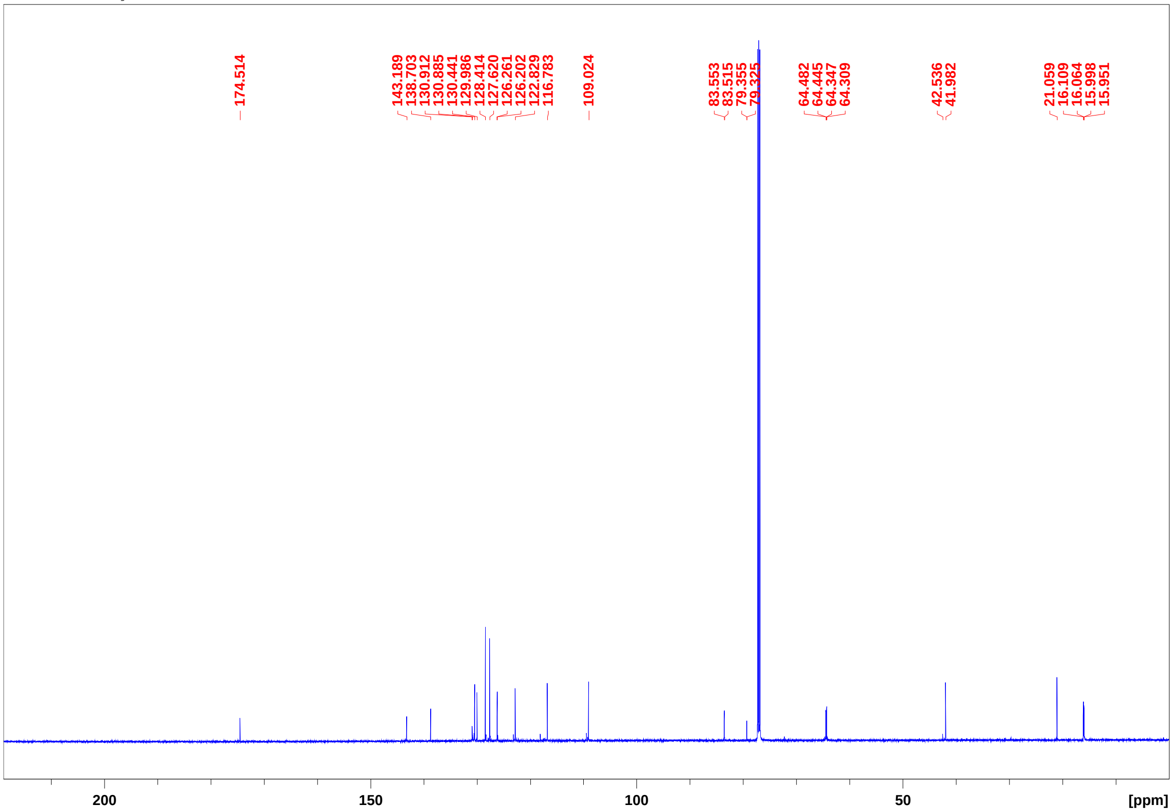
"MAH DR6655-single-13C" 1 1 Y:\mhorwitz
174.514
143.189
138.703
130.912
130.885
130.441
129.986
128.414
127.620
126.261
126.202
122.829
116.783
109.024
83.553
83.515
79.355
79.325
64.482
64.445
64.347
64.309
42.536
41.982
21.059
16.109
16.064
15.998
15.951
200
150
100
50
[ppm]

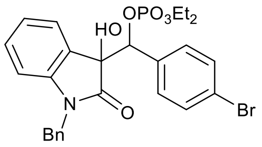
HO
OPO3Et2
N
O
Bn
Br

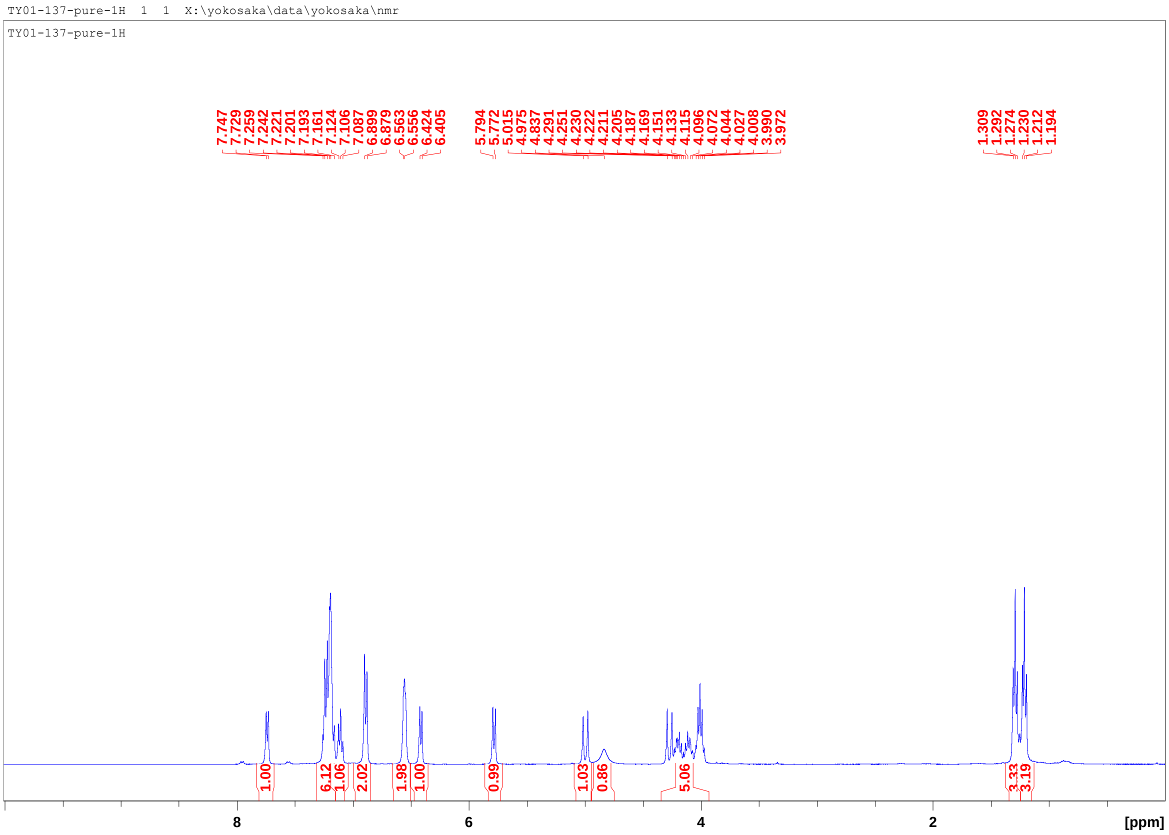
TY01-137-pure-1H 1 1 X:\yokosaka\data\yokosaka\nmr
TY01-137-pure-1H
7.747
7.729
7.259
7.242
7.221
7.201
7.193
7.161
7.124
7.106
7.087
6.899
6.879
6.563
6.556
6.424
6.405
5.794
5.772
5.015
4.975
4.837
4.291
4.251
4.230
4.222
4.211
4.205
4.187
4.169
4.151
4.133
4.115
4.096
4.072
4.044
4.027
4.008
3.990
3.972
1.309
1.292
1.274
1.230
1.212
1.194
1.00
6.12
1.06
2.02
1.98
1.00
0.99
1.03
0.86
5.06
3.33
3.19
8
6
4
2
[ppm]

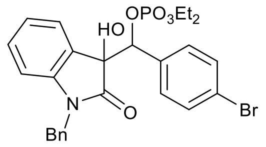

HO
OPO3Et2
N
O
Bn
Br

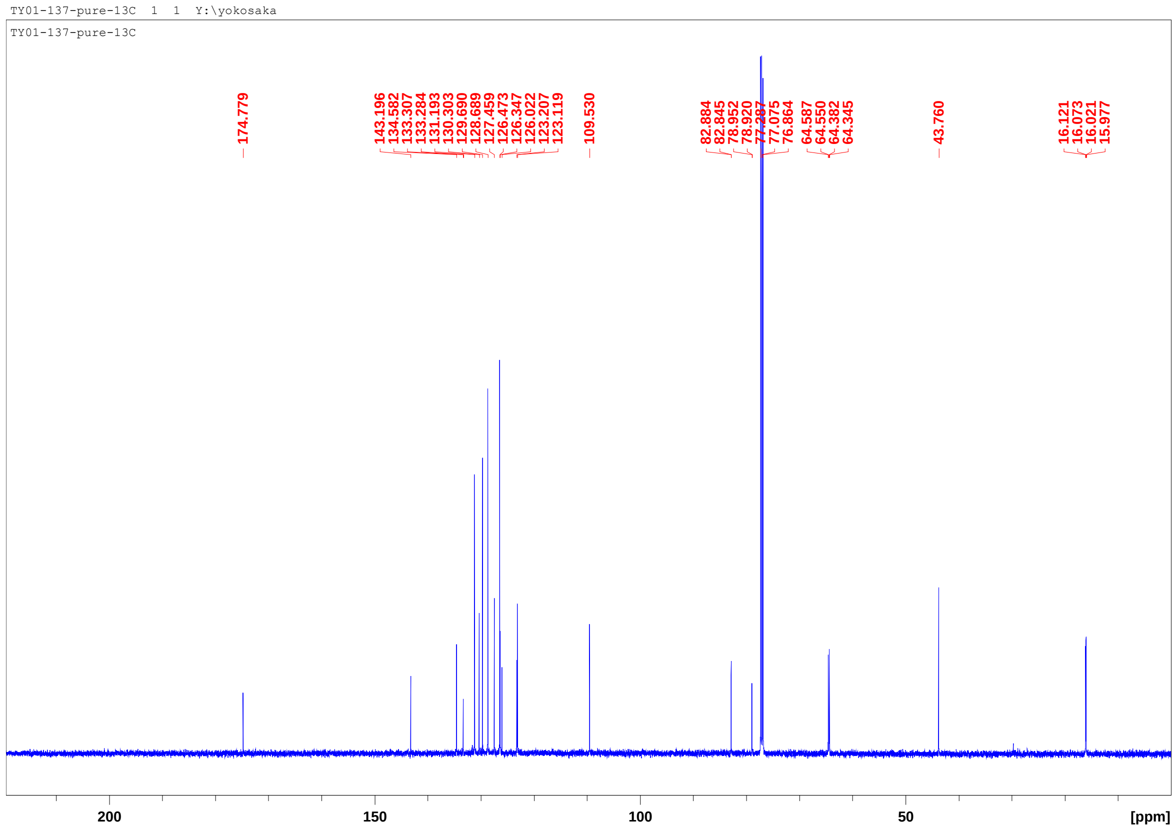

TY01-137-pure-13C 1 1 Y:\yokosaka
TY01-137-pure-13C
174.779
143.196
134.582
133.307
133.284
131.193
130.303
129.690
128.689
127.459
126.473
126.347
126.022
123.207
123.119
109.530
82.884
82.845
78.952
78.920
77.287
77.075
76.864
64.587
64.550
64.382
64.345
43.760
16.121
16.073
16.021
15.977
200
150
100
50
[ppm]

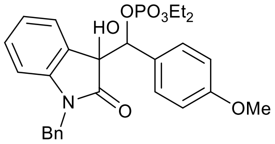

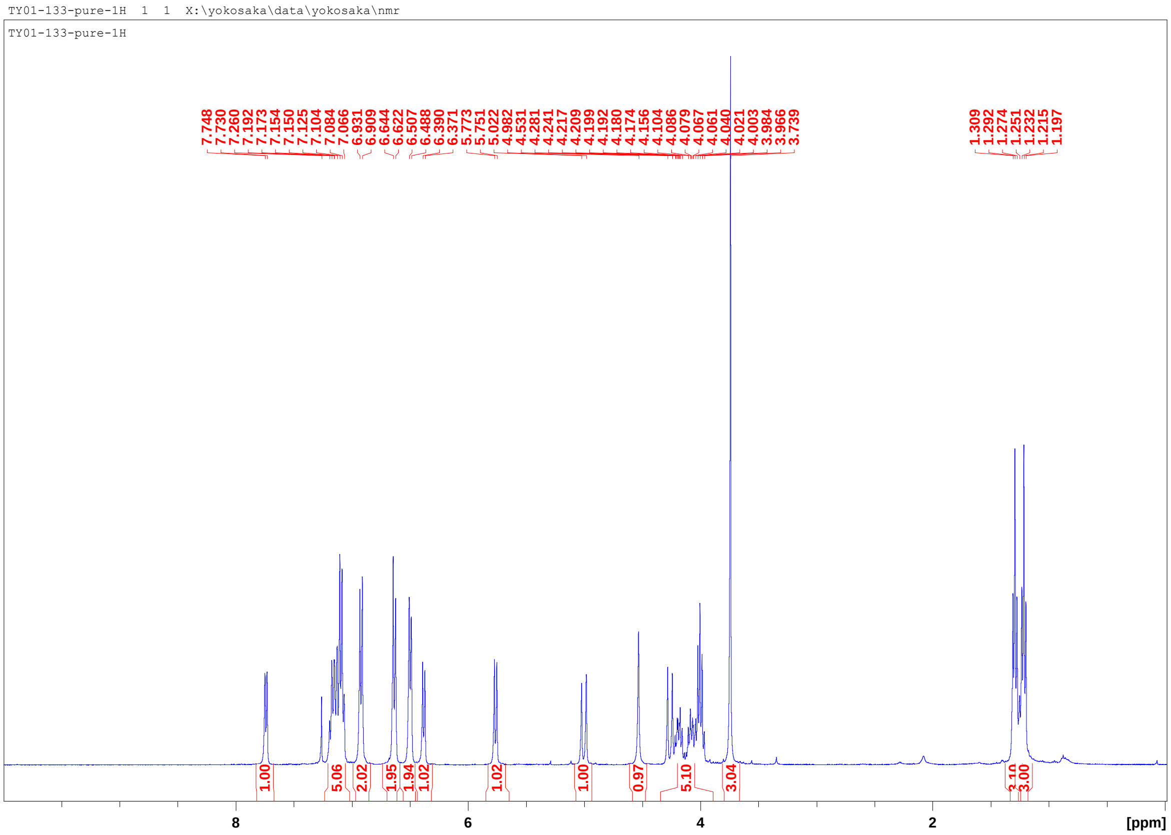
TY01-133-pure-1H 1 1 X:\yokosaka\data\yokosaka\nmr
TY01-133-pure-1H
[ppm]

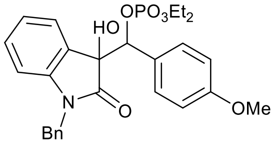
OPO3Et2
HO
N
O
Bn
OMe

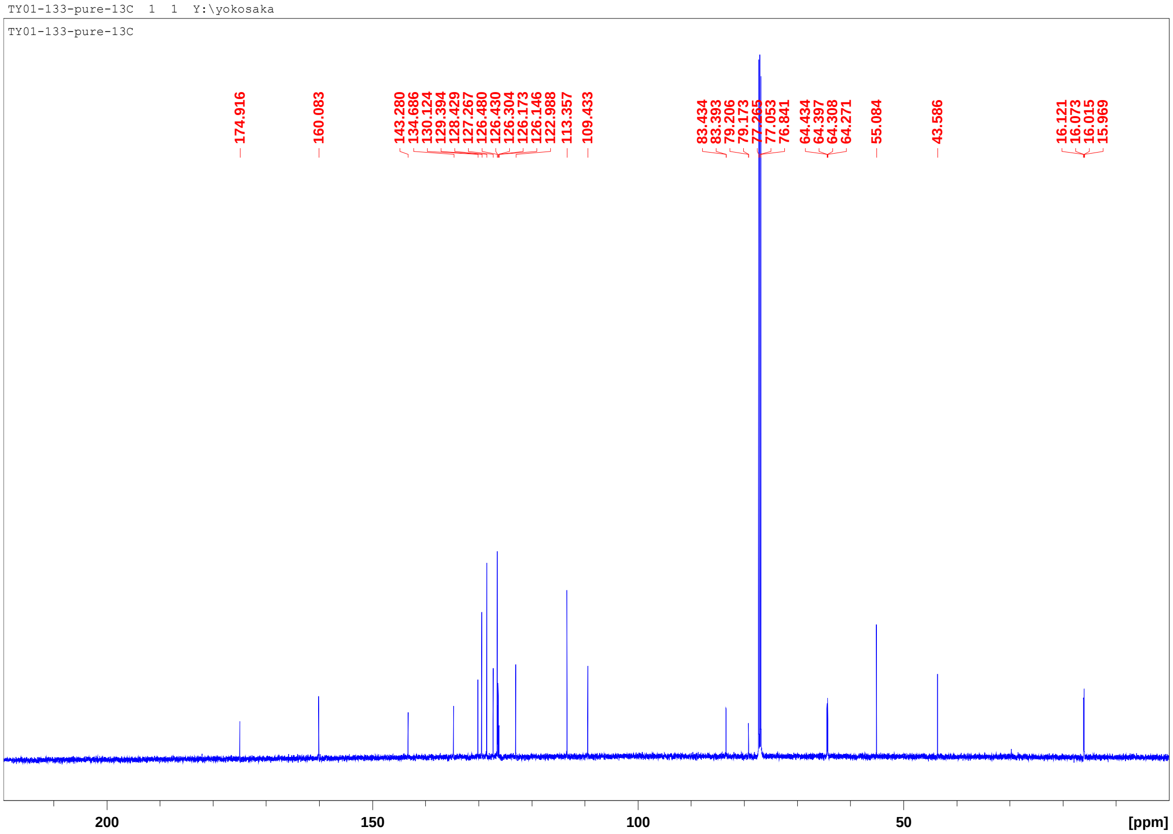
TY01-133-pure-13C 1 1 Y:\yokosaka
TY01-133-pure-13C
174.916
160.083
143.280
134.686
130.124
129.394
128.429
127.267
126.480
126.430
126.304
126.173
126.146
122.988
113.357
109.433
83.434
83.393
79.206
79.173
77.265
77.053
76.841
64.434
64.397
64.308
64.271
55.084
43.586
16.121
16.073
16.015
15.969
200
150
100
50
[ppm]

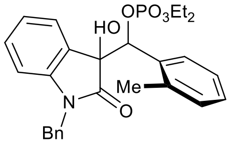

OPO3Et2
HO
Me
N
O
Bn

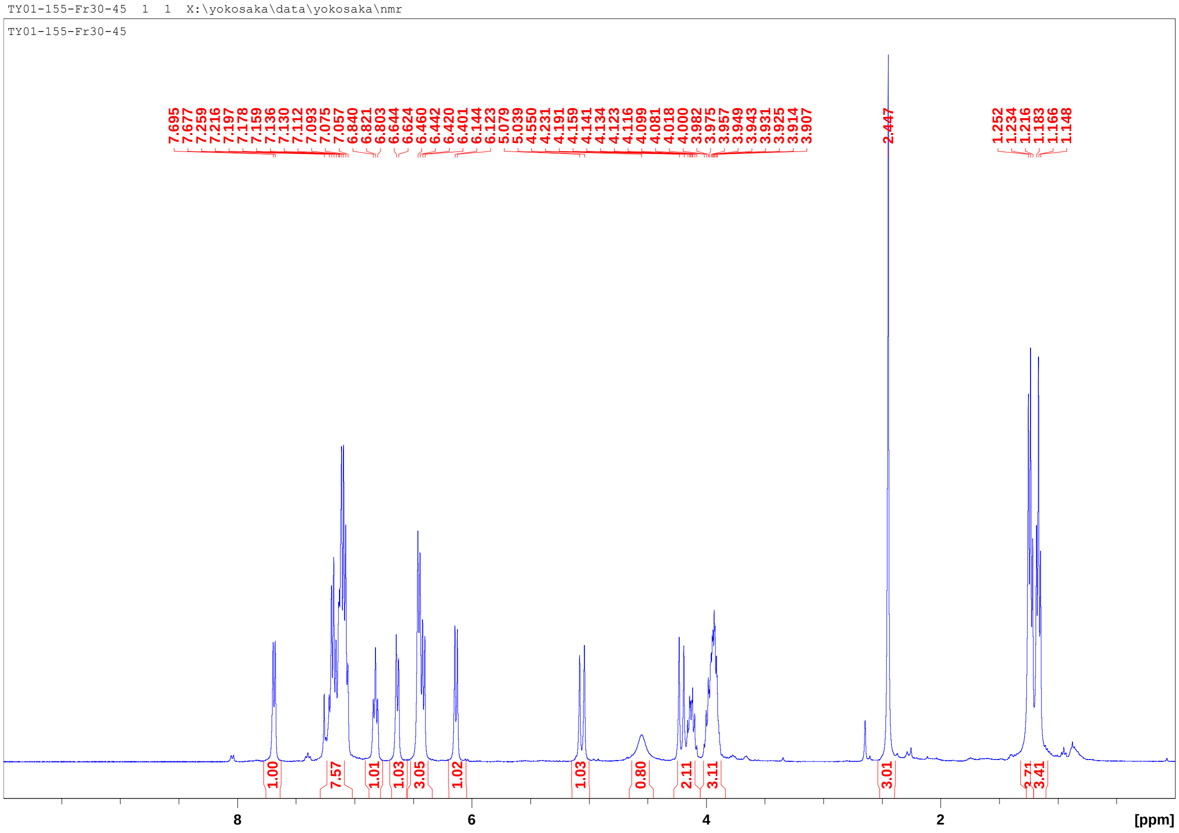

TY01-155-Fr30-45 1 1 X:\yokosaka\data\yokosaka\nmr
TY01-155-Fr30-45
7.695 7.677 7.259 7.216 7.197 7.178 7.159 7.136 7.130 7.112 7.093 7.075 7.057 6.840 6.821 6.803 6.644 6.624 6.460 6.442 6.420 6.401 6.144 6.123 5.079 5.039 4.550 4.231 4.191 4.159 4.141 4.134 4.123 4.116 4.099 4.081 4.018 4.000 3.982 3.975 3.957 3.949 3.943 3.931 3.925 3.914 3.907
2.447
1.252 1.234 1.216 1.183 1.166 1.148
1.00 7.57 1.01 1.03 3.05 1.02 1.03 0.80 2.11 3.11 3.01 2.71 3.41
8
6
4
2
[ppm]

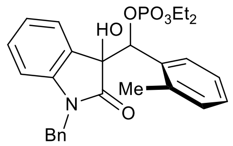

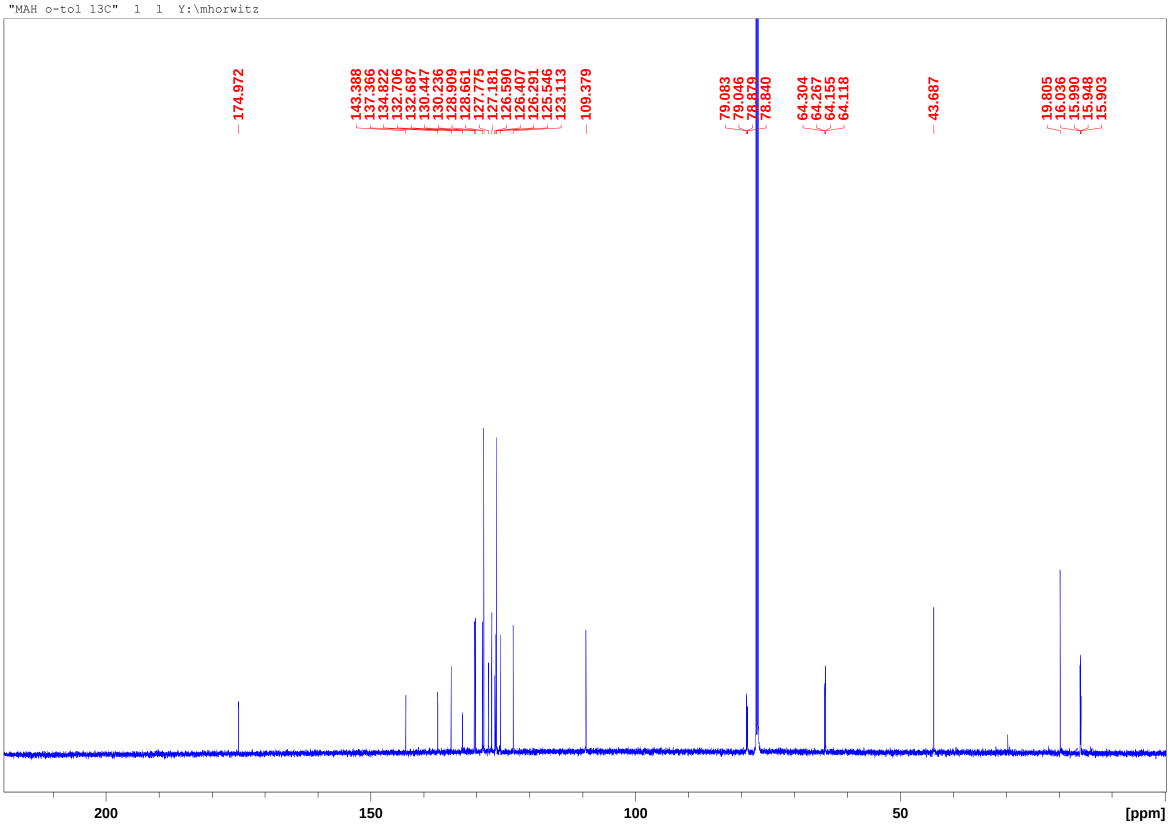
"MAH o-tol 13C" 1 1 Y:\mhorwitz
174.972
143.388
137.366
134.822
132.706
132.687
130.447
130.236
128.909
128.661
127.775
127.181
126.590
126.407
126.291
125.546
123.113
109.379
79.083
79.046
78.879
78.840
64.304
64.267
64.155
64.118
43.687
19.805
16.036
15.990
15.948
15.903
200
150
100
50
[ppm]

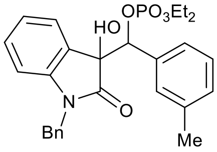

HO
OPO3Et2
N
O
Bn
Me

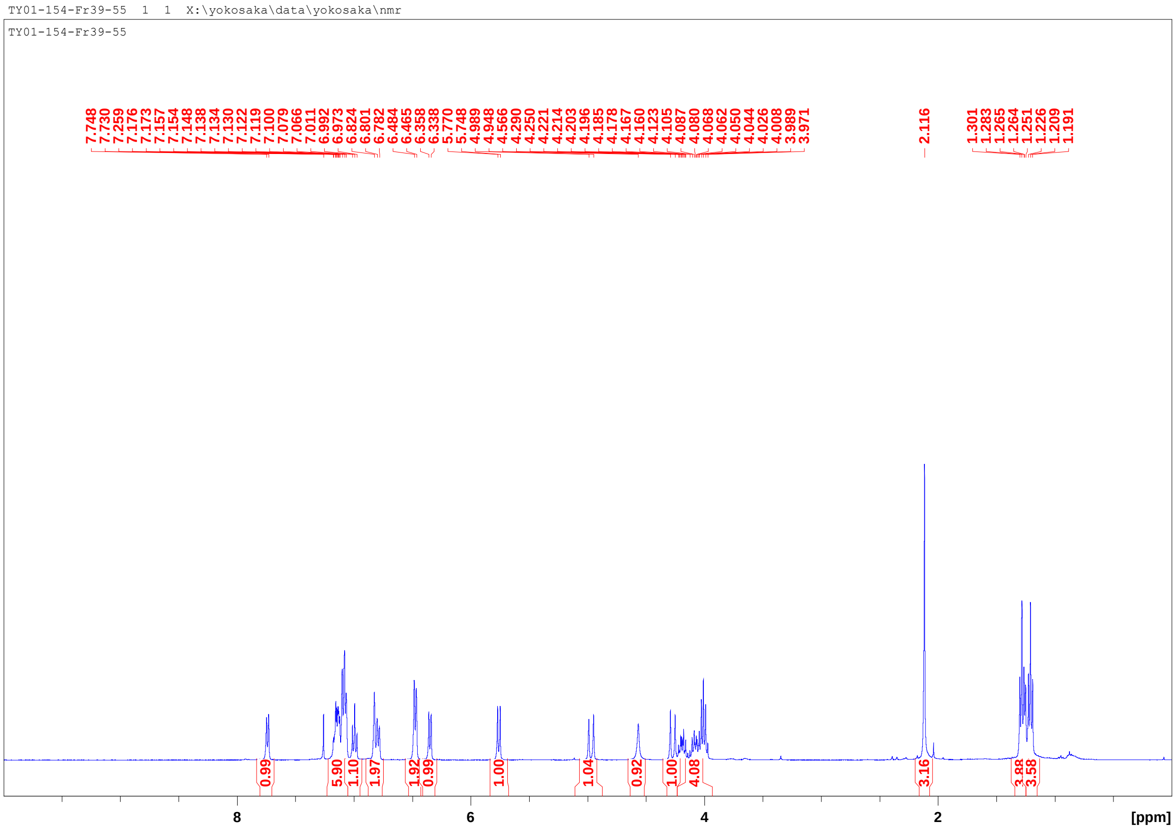

TY01-154-Fr39-55 1 1 X:\yokosaka\data\yokosaka\nmr
TY01-154-Fr39-55
[ppm]

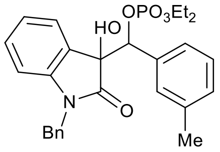

OPO3Et2
HO
N
O
Bn
Me

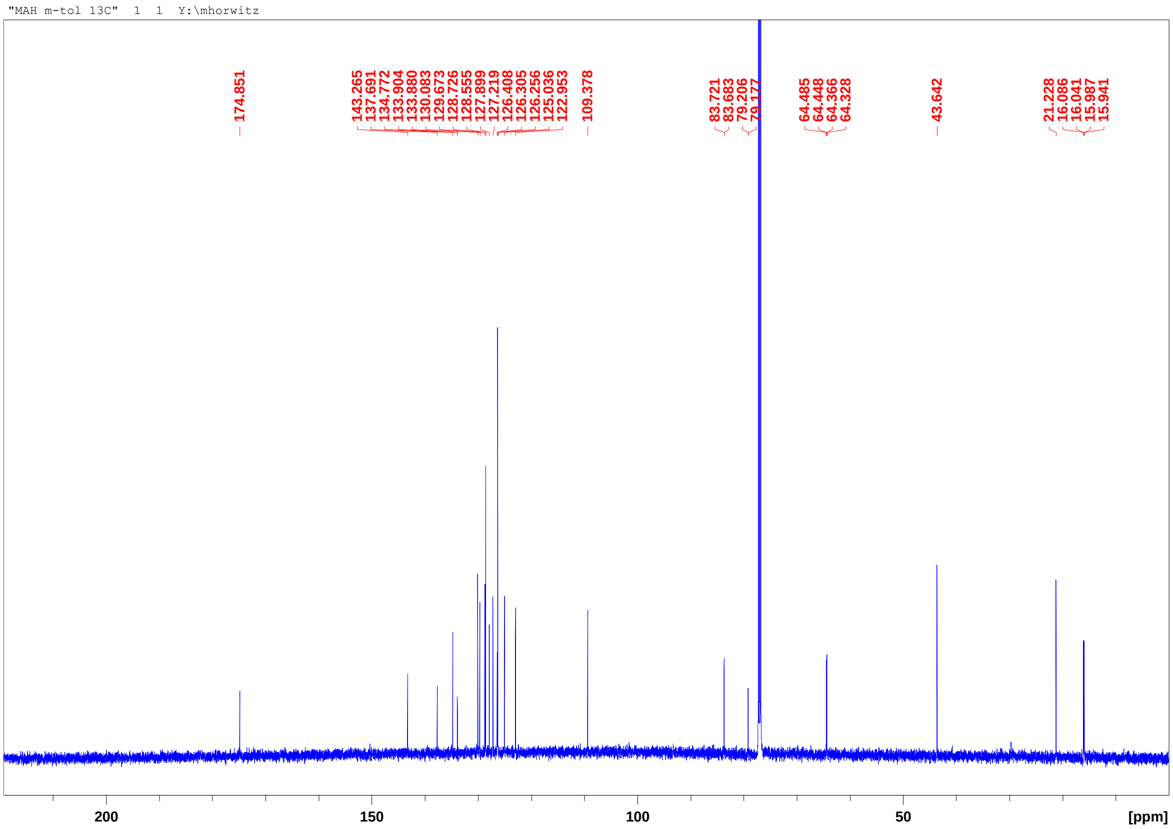

"MAH m-tol 13C" 1 1 Y:\mhorwitz
174.851
143.265
137.691
134.772
133.904
133.880
130.083
129.673
128.726
128.555
127.899
127.219
126.408
126.305
126.256
125.036
122.953
109.378
83.721
83.683
79.206
79.177
64.485
64.448
64.366
64.328
43.642
21.228
16.086
16.041
15.987
15.941
200
150
100
50
[ppm]

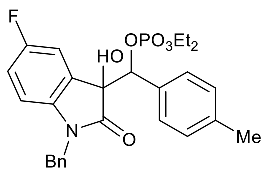

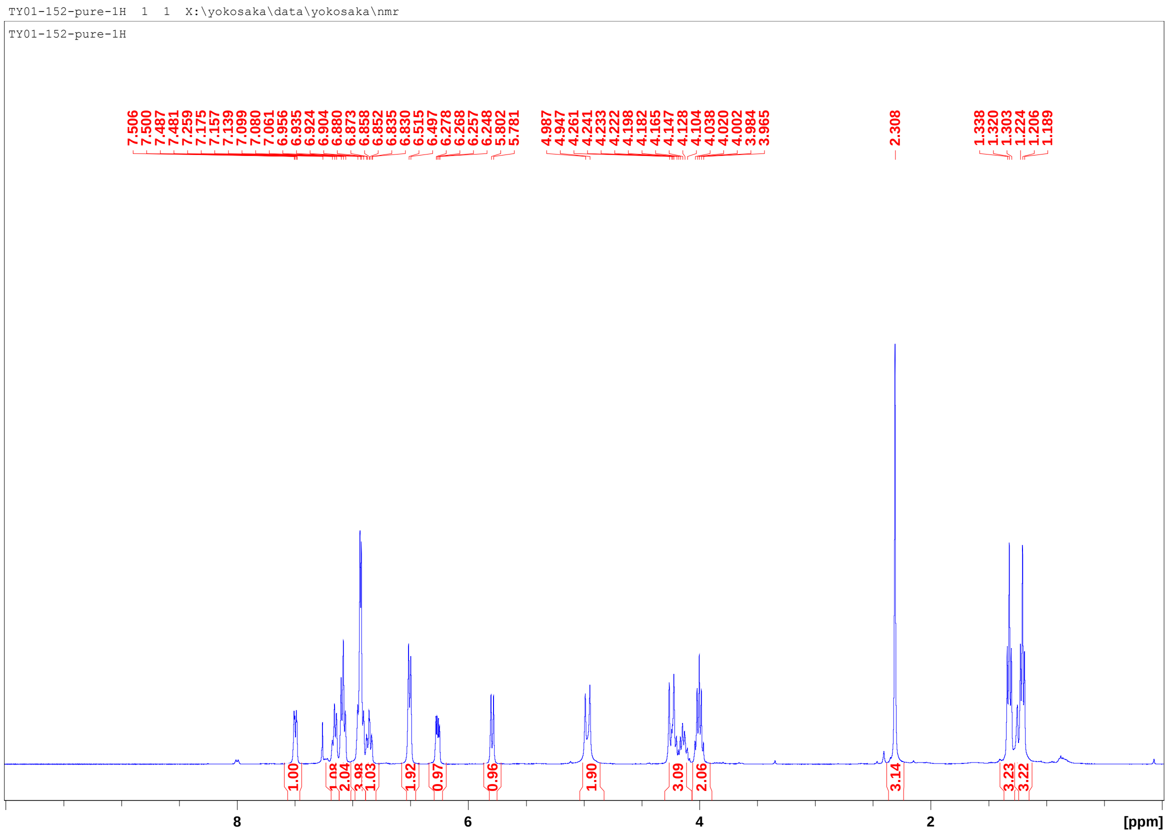

TY01-152-pure-1H 1 1 X:\yokosaka\data\yokosaka\nmr
TY01-152-pure-1H
7.506
7.500
7.487
7.481
7.259
7.175
7.157
7.139
7.099
7.080
7.061
6.956
6.935
6.924
6.904
6.880
6.873
6.858
6.852
6.835
6.830
6.515
6.497
6.278
6.268
6.257
6.248
5.802
5.781
4.987
4.947
4.261
4.241
4.233
4.222
4.198
4.182
4.165
4.147
4.128
4.104
4.038
4.020
4.002
3.984
3.965
2.308
1.338
1.320
1.303
1.224
1.206
1.189
1.00
1.08
2.04
3.98
1.03
1.92
0.97
0.96
1.90
3.09
2.06
3.14
3.23
3.22
8
6
4
2
[ppm]

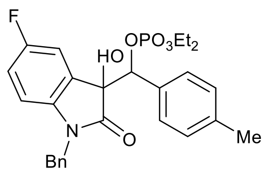
F
HO
OPO3Et2
N
O
Bn
Me

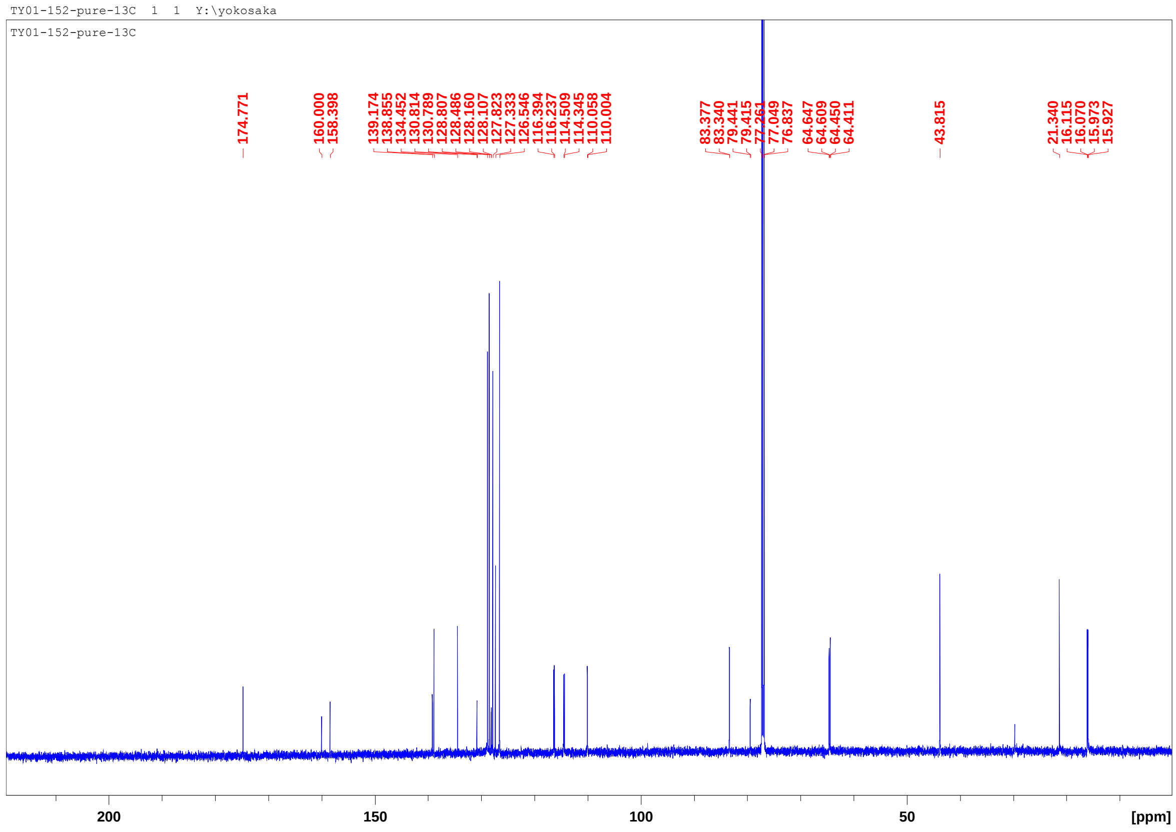
TY01-152-pure-13C 1 1 Y:\yokosaka
TY01-152-pure-13C
174.771
160.000
158.398
139.174
138.855
134.452
130.814
130.789
128.807
128.486
128.160
128.107
127.823
127.333
126.546
116.394
116.237
114.509
114.345
110.058
110.004
83.377
83.340
79.441
79.415
77.261
77.049
76.837
64.647
64.609
64.450
64.411
43.815
21.340
16.115
16.070
15.973
15.927
200
150
100
50
[ppm]

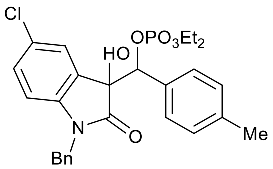
Cl
OPO3Et2
HO
N
O
Bn
Me

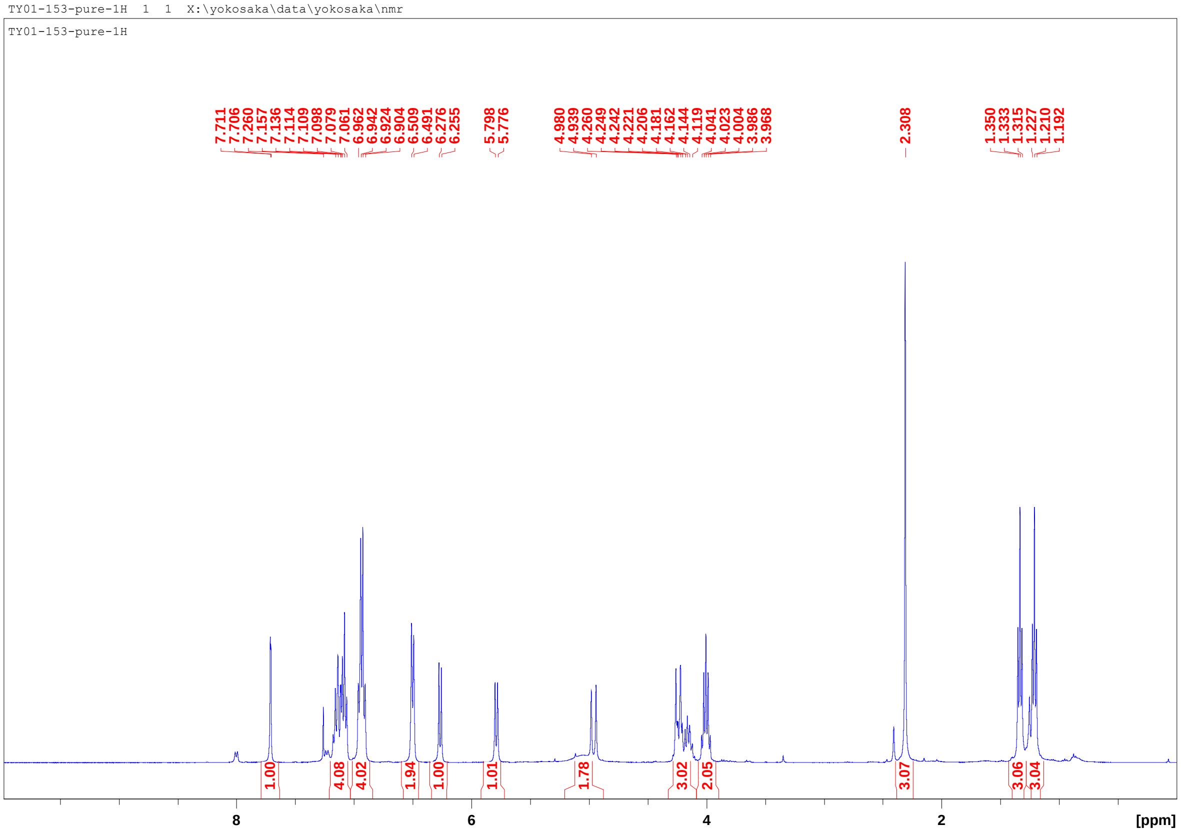
TY01-153-pure-1H 1 1 X:\yokosaka\data\yokosaka\nmr
TY01-153-pure-1H
7.711
7.706
7.260
7.157
7.136
7.114
7.109
7.098
7.079
7.061
6.962
6.942
6.924
6.904
6.509
6.491
6.276
6.255
5.798
5.776
4.980
4.939
4.260
4.249
4.242
4.221
4.206
4.181
4.162
4.144
4.119
4.041
4.023
4.004
3.986
3.968
2.308
1.350
1.333
1.315
1.227
1.210
1.192
1.00
4.08
4.02
1.94
1.00
1.01
1.78
3.02
2.05
3.07
3.06
3.04
8
6
4
2
[ppm]

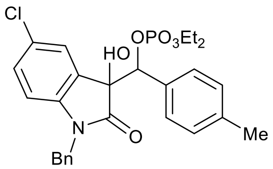
Cl
HO
OPO3Et2
N
O
Bn
Me

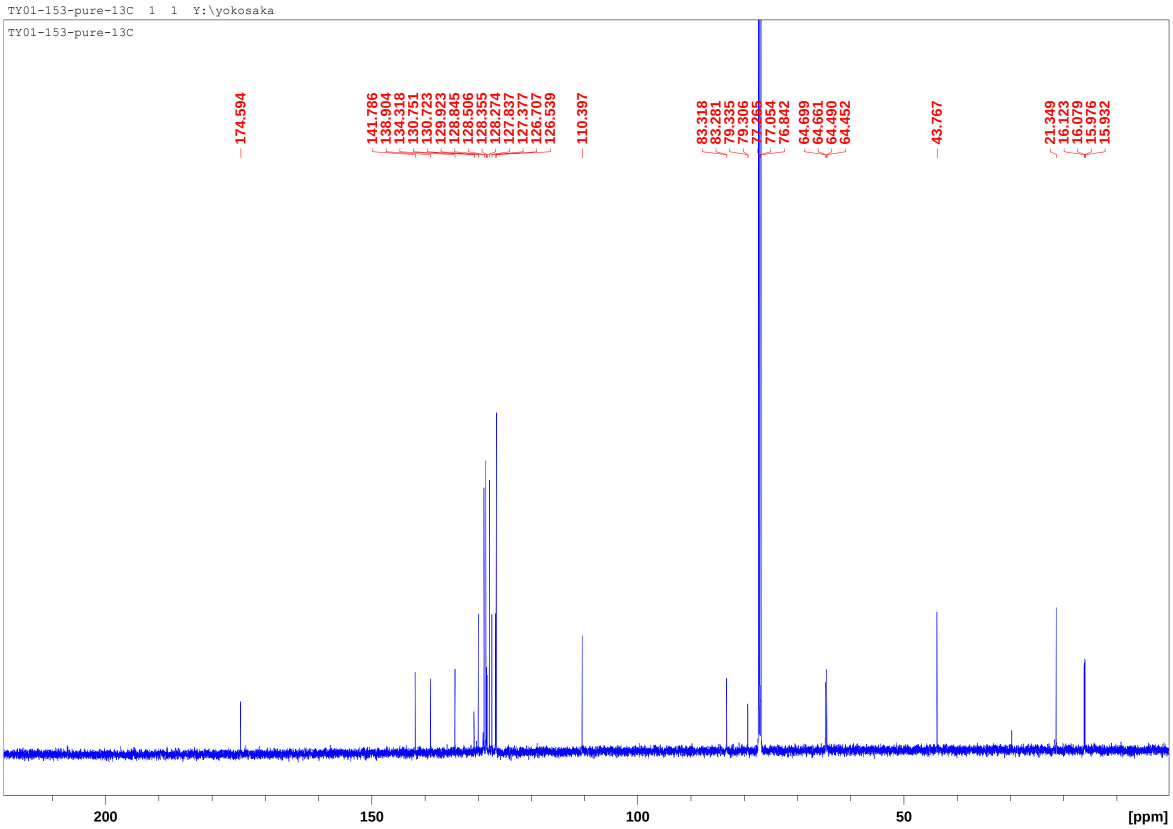
TY01-153-pure-13C 1 1 Y:\yokosaka
TY01-153-pure-13C
174.594
141.786
138.904
134.318
130.751
130.723
129.923
128.845
128.506
128.355
128.274
127.837
127.377
126.707
126.539
110.397
83.318
83.281
79.335
79.306
77.265
77.054
76.842
64.699
64.661
64.490
64.452
43.767
21.349
16.123
16.079
15.976
15.932
200
150
100
50
[ppm]

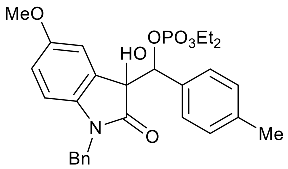
MeO
HO
OPO3Et2
N
O
Bn
Me

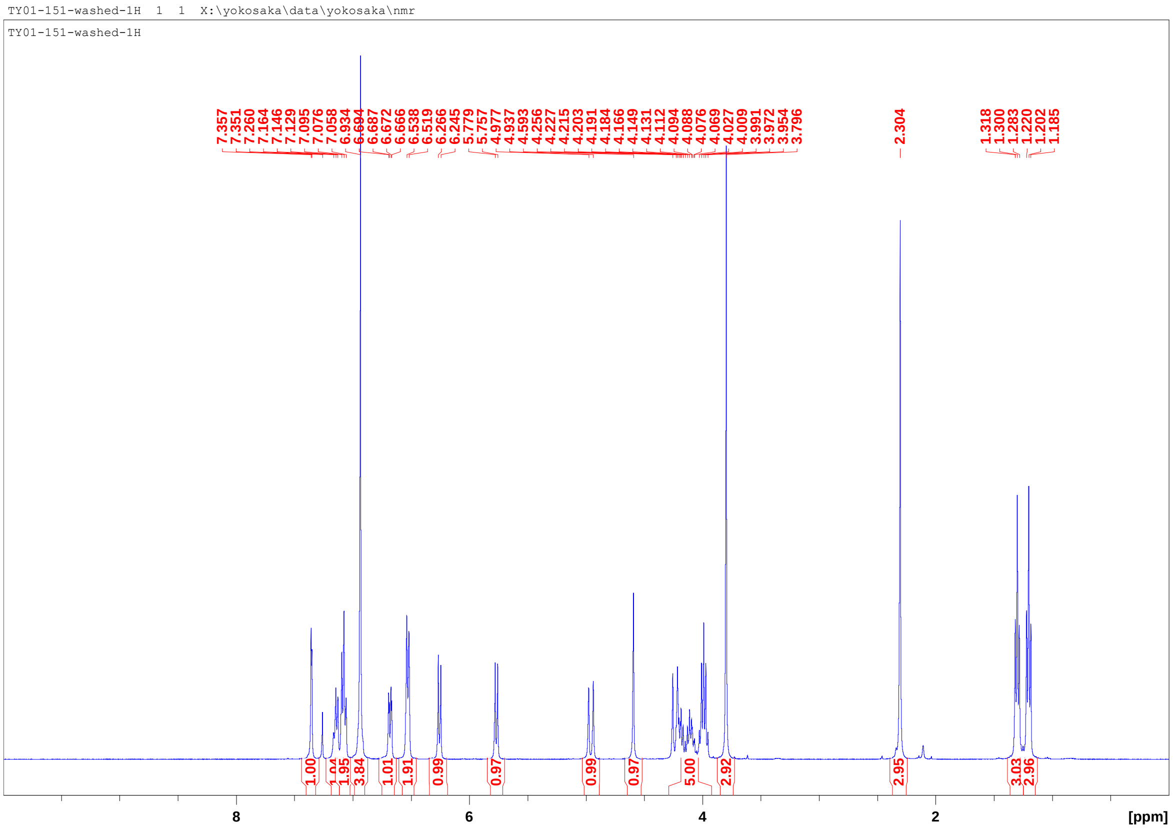
TY01-151-washed-1H 1 1 X:\yokosaka\data\yokosaka\nmr
TY01-151-washed-1H
7.357
7.351
7.260
7.164
7.146
7.129
7.095
7.076
7.058
6.934
6.694
6.687
6.672
6.666
6.538
6.519
6.266
6.245
5.779
5.757
4.977
4.937
4.593
4.256
4.227
4.215
4.203
4.191
4.184
4.166
4.149
4.131
4.112
4.094
4.088
4.076
4.069
4.027
4.009
3.991
3.972
3.954
3.796
2.304
1.318
1.300
1.283
1.220
1.202
1.185
1.00
1.04
1.95
3.84
1.01
1.91
0.99
0.97
0.99
0.97
5.00
2.92
2.95
3.03
2.96
8
6
4
2
[ppm]

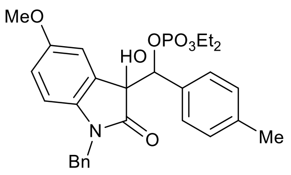
MeO
HO
OPO3Et2
N
O
Bn
Me

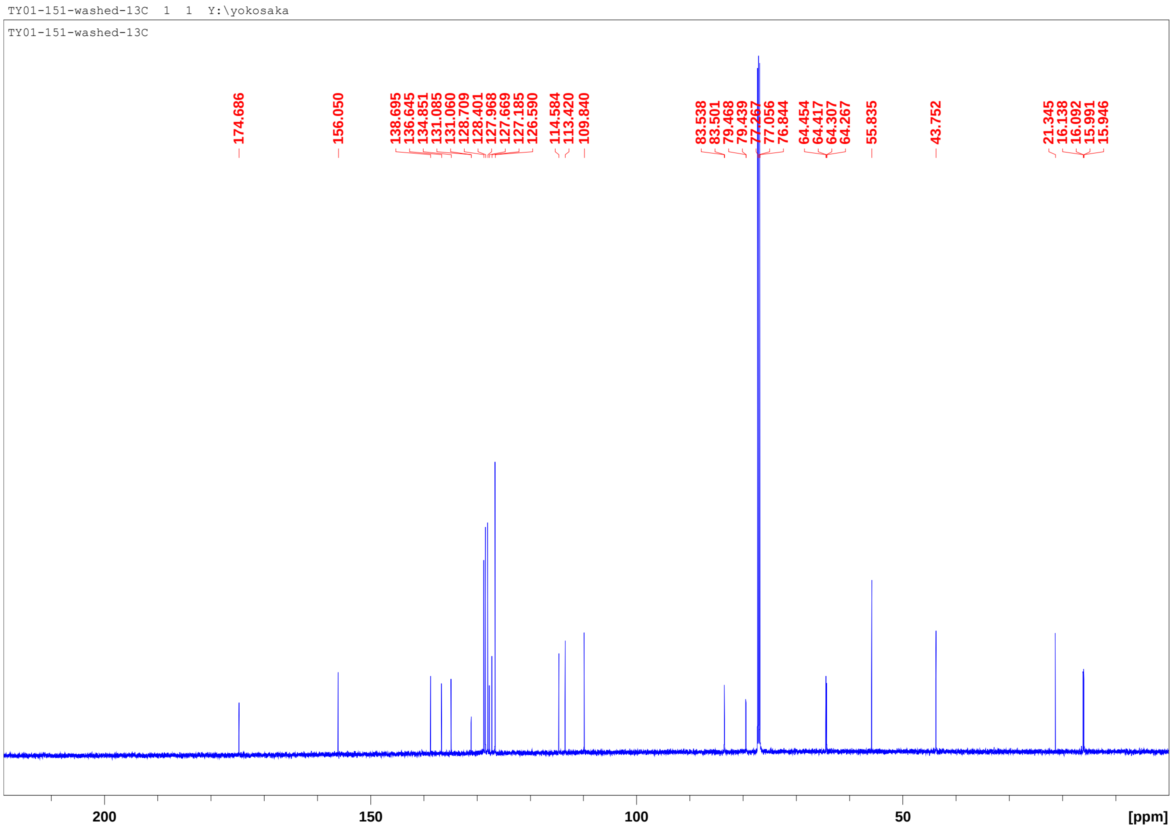
TY01-151-washed-13C 1 1 Y:\yokosaka
TY01-151-washed-13C
174.686
156.050
138.695
136.645
134.851
131.085
131.060
128.709
128.401
127.968
127.669
127.185
126.590
114.584
113.420
109.840
83.538
83.501
79.468
79.439
77.267
77.056
76.844
64.454
64.417
64.307
64.267
55.835
43.752
21.345
16.138
16.092
15.991
15.946
200
150
100
50
[ppm]

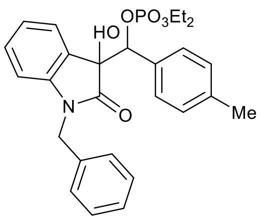

OPO3Et2
HO
N
O
Me

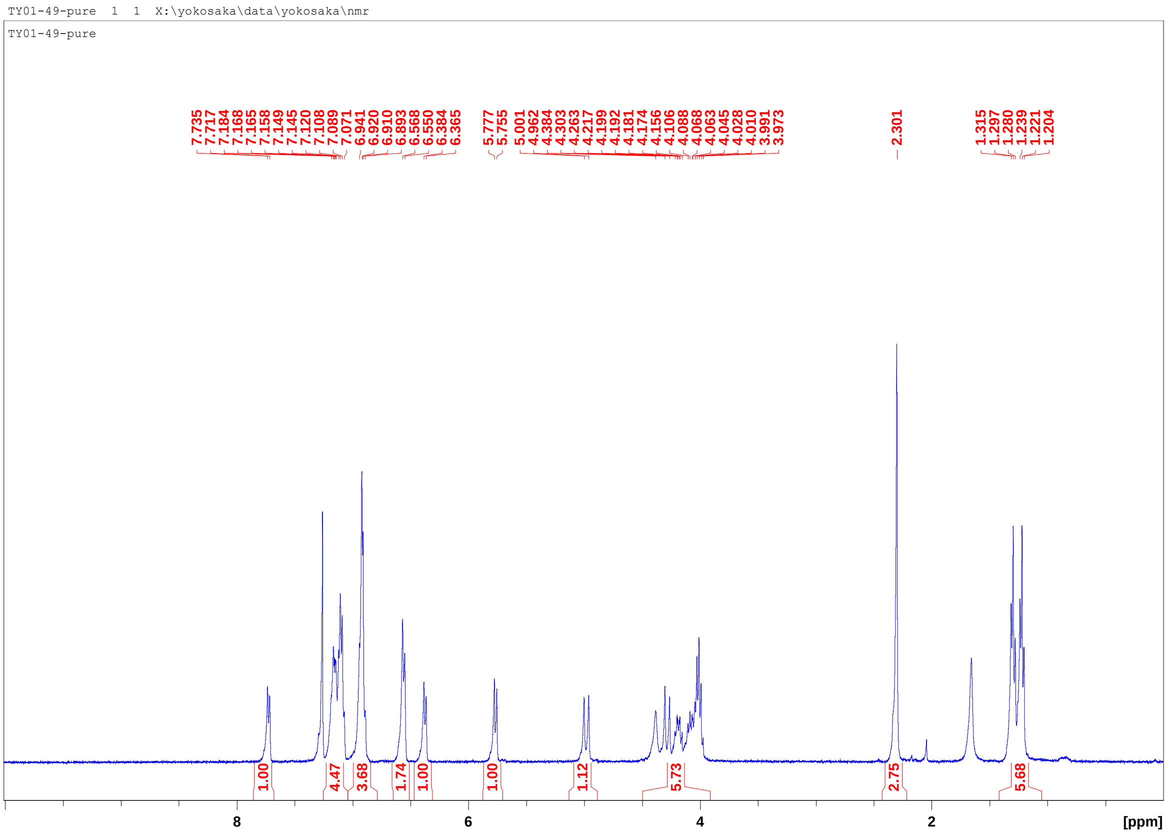

TY01-49-pure 1 1 X:\yokosaka\data\yokosaka\nmr
TY01-49-pure
7.735
7.717
7.184
7.168
7.165
7.158
7.149
7.145
7.120
7.108
7.089
7.071
6.941
6.920
6.910
6.893
6.568
6.550
6.384
6.365
5.777
5.755
5.001
4.962
4.384
4.303
4.263
4.217
4.199
4.192
4.181
4.174
4.156
4.106
4.088
4.068
4.063
4.045
4.028
4.010
3.991
3.973
2.301
1.315
1.297
1.280
1.239
1.221
1.204
1.00
4.47
3.68
1.74
1.00
1.00
1.12
5.73
2.75
5.68
8
6
4
2
[ppm]

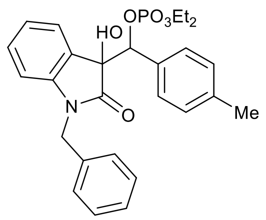

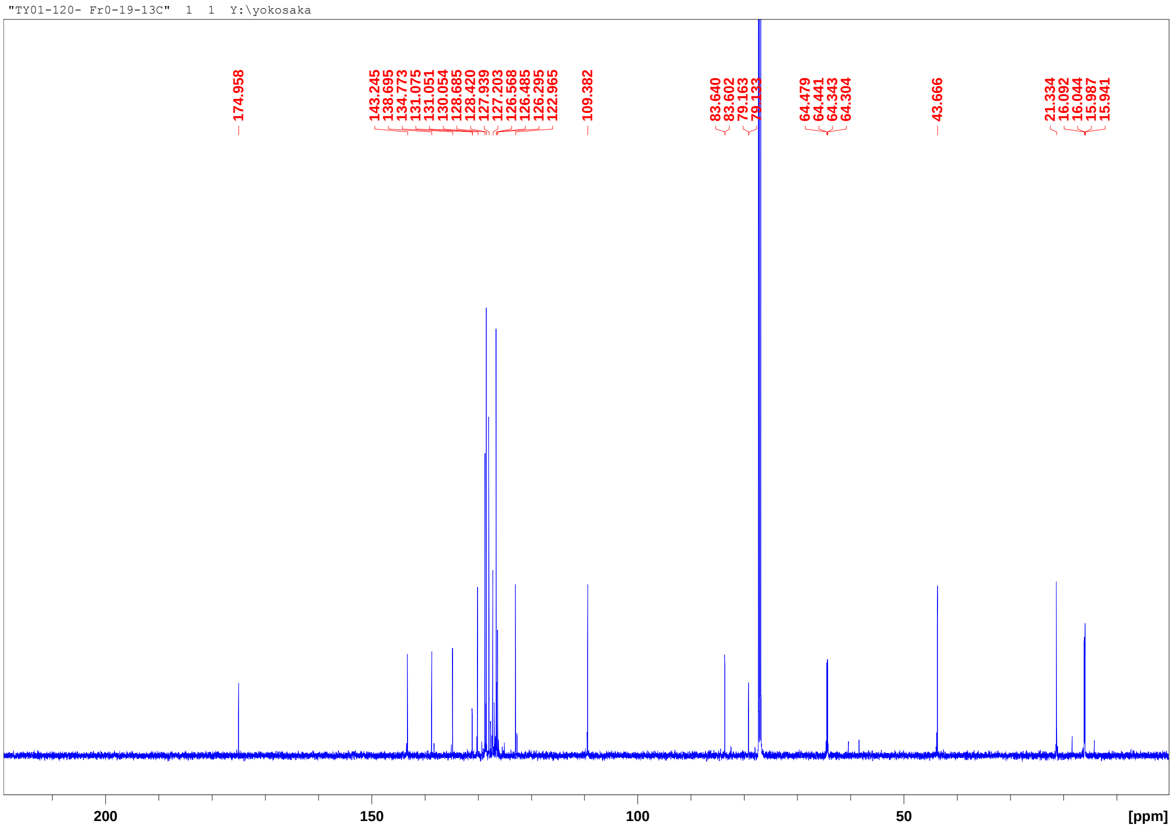
"TY01-120- Fr0-19-13C" 1 1 Y:\yokosaka
174.958
143.245
138.695
134.773
131.075
131.051
130.054
128.685
128.420
127.939
127.203
126.568
126.485
126.295
122.965
109.382
83.640
83.602
79.163
79.133
64.479
64.441
64.343
64.304
43.666
21.334
16.092
16.044
15.987
15.941
200
150
100
50
[ppm]

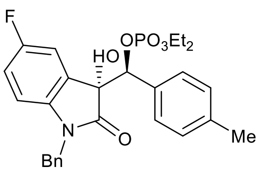

F
OPO3Et2
HO
N
O
Me
Bn

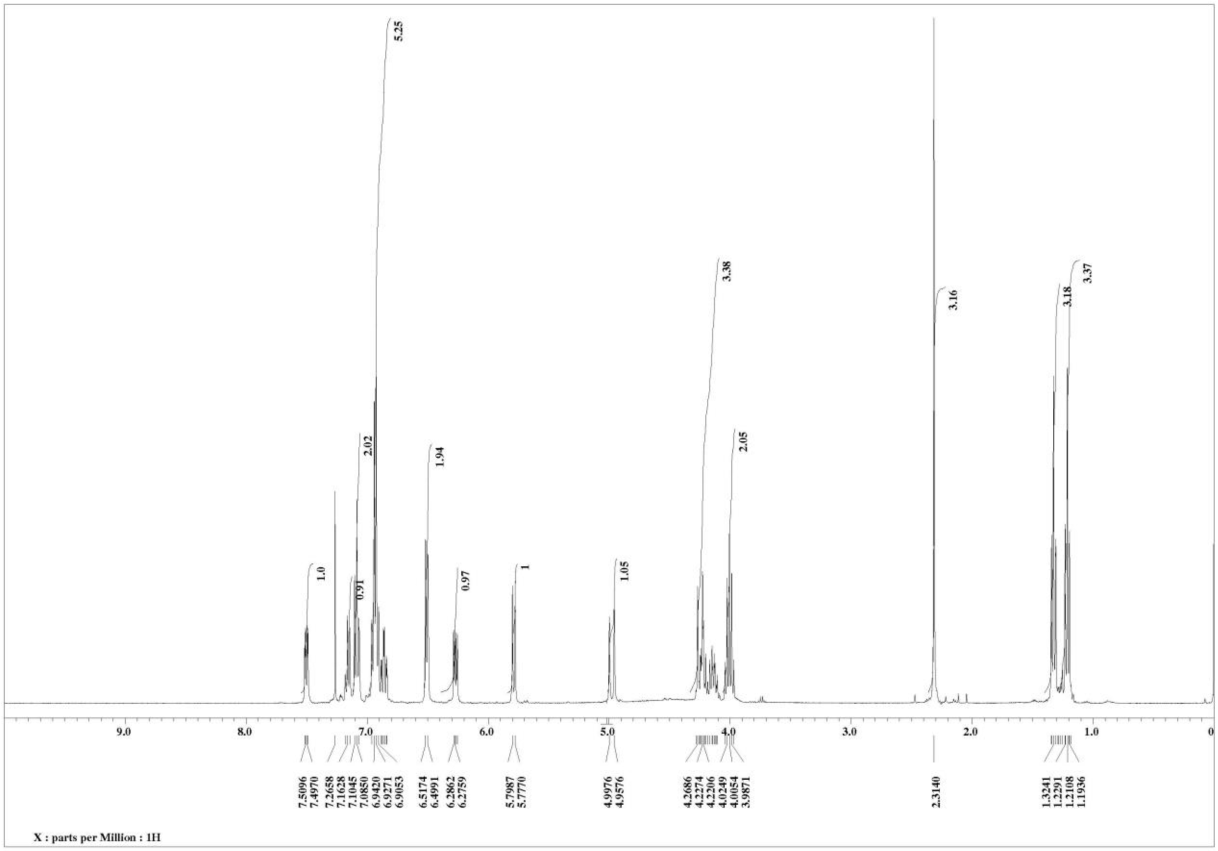

X : parts per Million : 1H

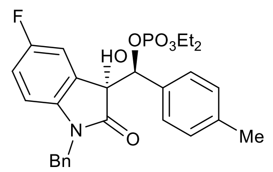
F
OPO3Et2
HO
N
O
Bn
Me

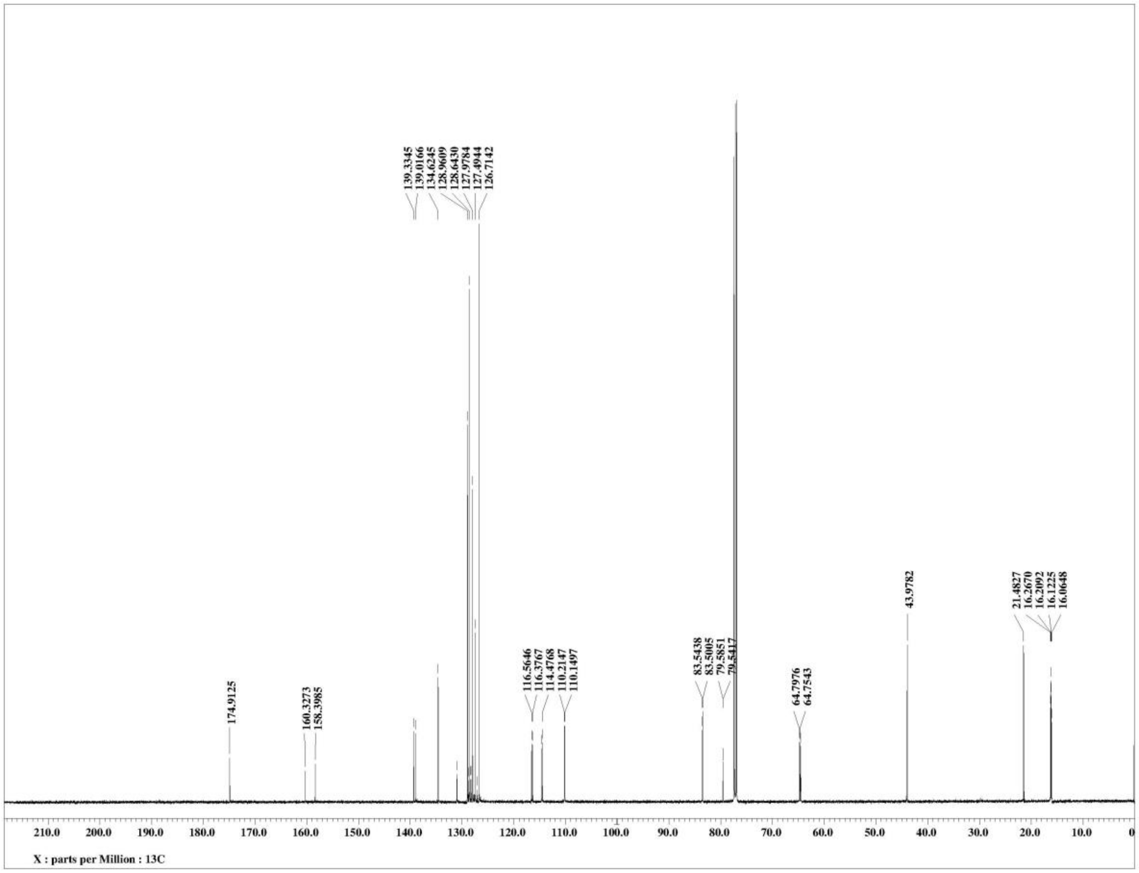
139.3345
139.0166
134.6245
128.9609
128.6430
127.9784
127.4944
126.7142
174.9125
160.3273
158.3985
116.5646
116.3767
114.4768
110.2147
110.1497
83.5438
83.5005
79.5851
79.5417
64.7976
64.7543
43.9782
21.4827
16.2670
16.2092
16.1225
16.0648
210.0
200.0
190.0
180.0
170.0
160.0
150.0
140.0
130.0
120.0
110.0
100.0
90.0
80.0
70.0
60.0
50.0
40.0
30.0
20.0
10.0
0
X : parts per Million : 13C

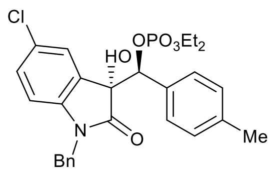
Cl
OPO3Et2
HO
N
O
Bn
Me

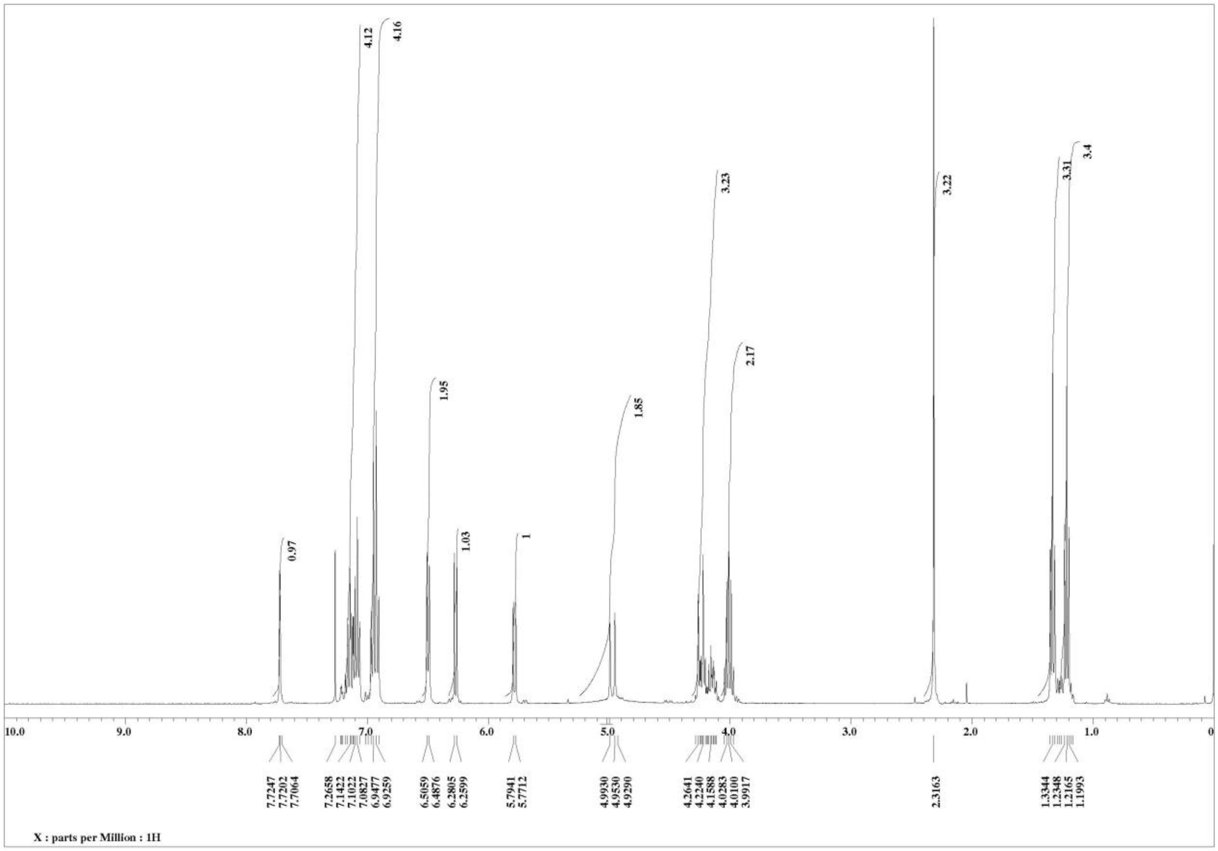
X : parts per Million : 1H
10.0
9.0
8.0
7.0
6.0
5.0
4.0
3.0
2.0
1.0
0
0.97
4.12
4.16
1.95
1.03
1
1.85
3.23
2.17
3.22
3.31
3.4
7.7247
7.7202
7.7064
7.2658
7.1422
7.1022
7.0827
6.9477
6.9259
6.5059
6.4876
6.2805
6.2599
5.7941
5.7712
4.9930
4.9530
4.9290
4.2641
4.2240
4.1588
4.0283
4.0100
3.9917
2.3163
1.3344
1.2348
1.2165
1.1993

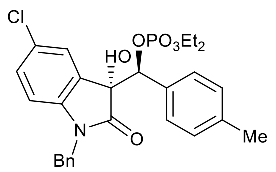
Cl
OPO3Et2
HO
N
O
Bn
Me

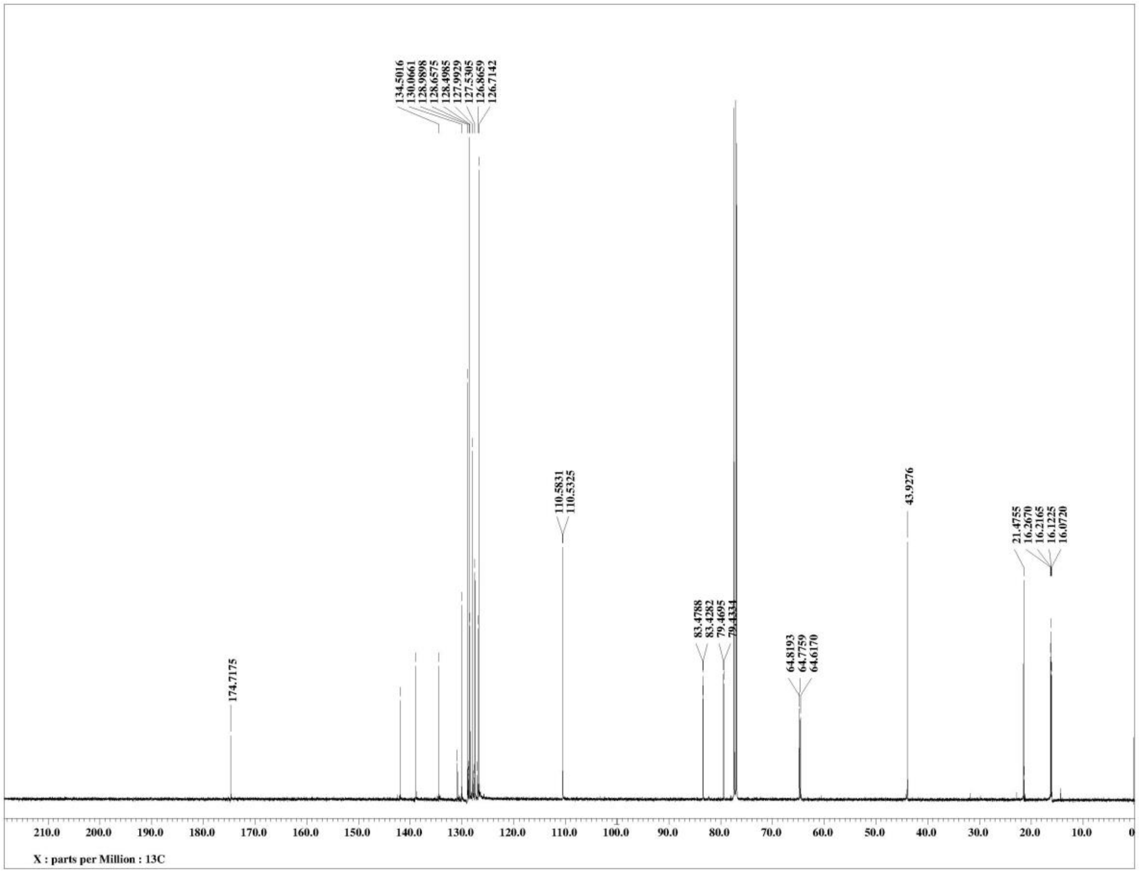
134.5016
130.0661
128.9898
128.6575
128.4985
127.9929
127.5305
126.8659
126.7142
110.5831
110.5325
83.4788
83.4282
79.4695
79.4334
64.8193
64.7759
64.6170
43.9276
21.4755
16.2670
16.2165
16.1225
16.0720
174.7175
X : parts per Million : 13C

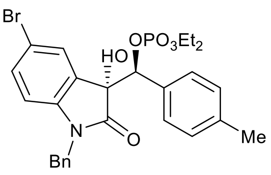
Br
OPO3Et2
HO
N
O
Bn
Me

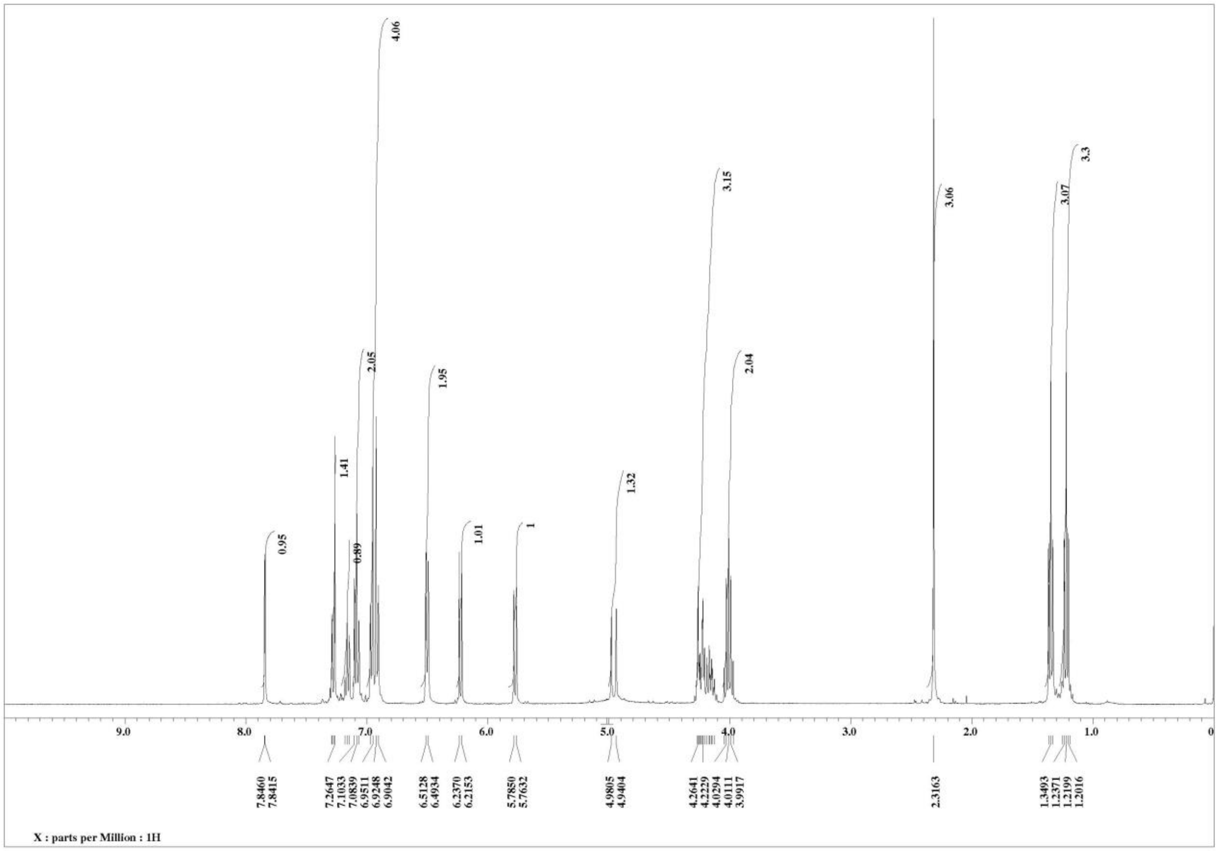
0.95
1.41
0.89
2.05
4.06
1.95
1.01
1
1.32
3.15
2.04
3.06
3.07
3.3
9.0
8.0
7.0
6.0
5.0
4.0
3.0
2.0
1.0
7.8460
7.8415
7.2647
7.1033
7.0839
6.9511
6.9248
6.9042
6.5128
6.4934
6.2370
6.2153
5.7850
5.7632
4.9805
4.9404
4.2641
4.2229
4.0294
4.0111
3.9917
2.3163
1.3493
1.2371
1.2199
1.2016
X : parts per Million : 1H

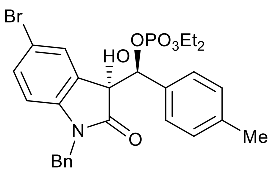
Br
OPO3Et2
HO
N
O
Bn
Me

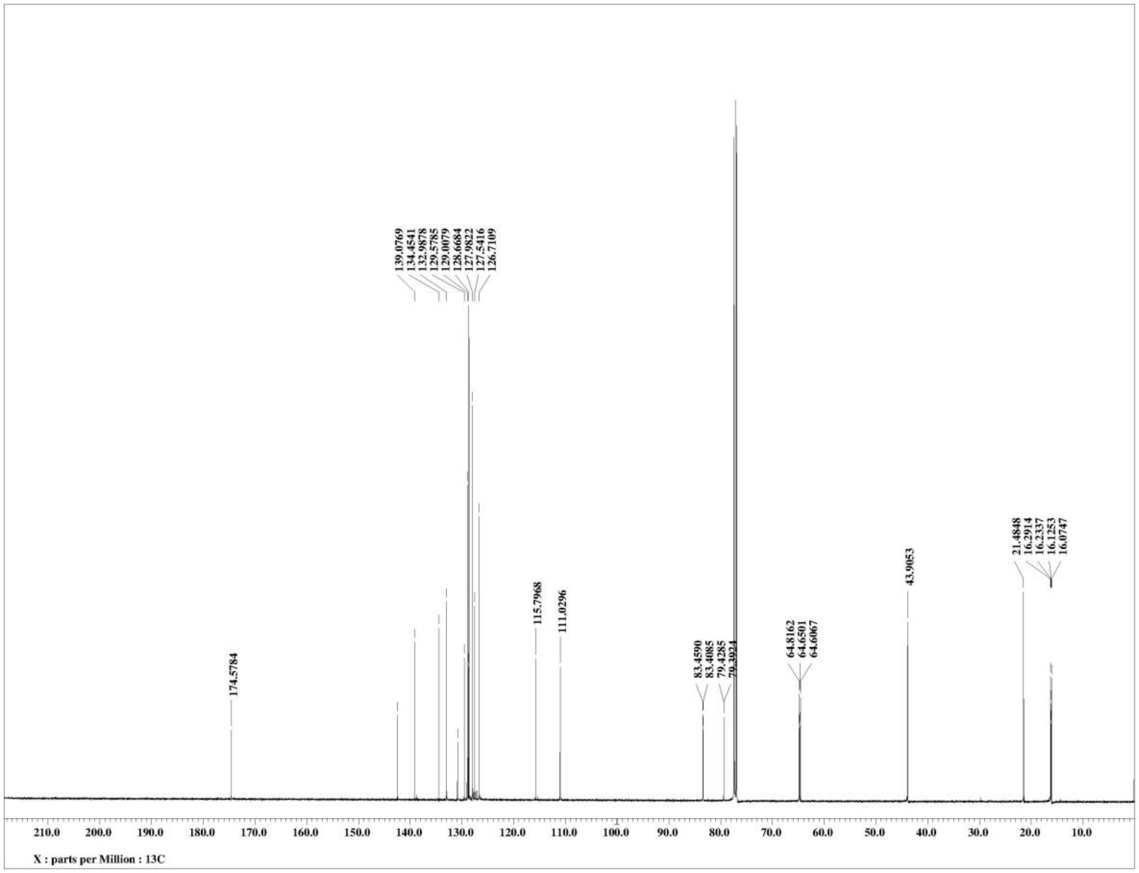
139.0769
134.4541
132.9878
129.5785
129.0079
128.6684
127.9822
127.5416
126.7109
174.5784
115.7968
111.0296
83.4590
83.4085
79.4285
79.3924
64.8162
64.6501
64.6067
43.9053
21.4848
16.2914
16.2337
16.1253
16.0747
210.0
200.0
190.0
180.0
170.0
160.0
150.0
140.0
130.0
120.0
110.0
100.0
90.0
80.0
70.0
60.0
50.0
40.0
30.0
20.0
10.0
X : parts per Million : 13C

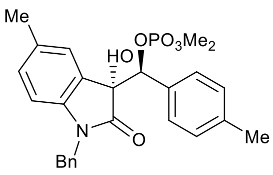
Me
OPO3Me2
HO
N
O
Bn
Me

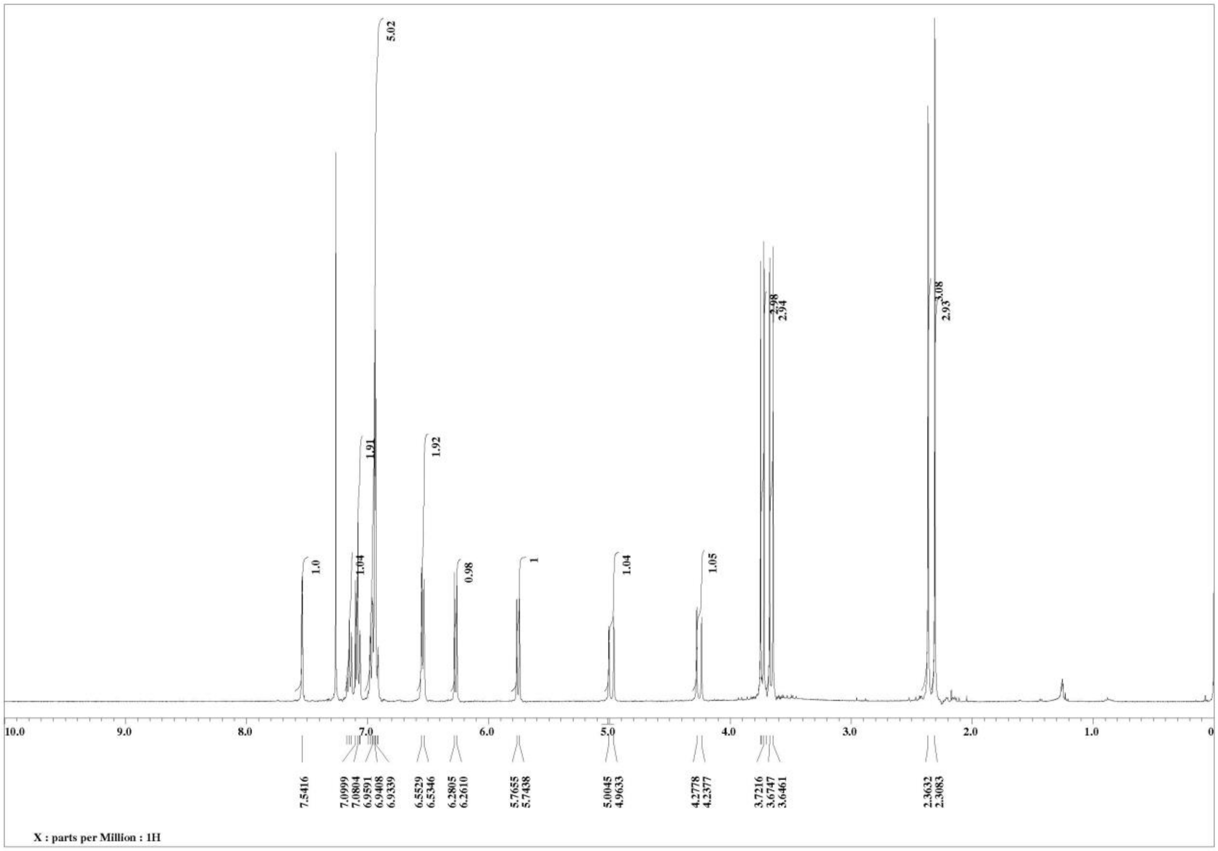
X : parts per Million : 1H
10.0
9.0
8.0
7.0
6.0
5.0
4.0
3.0
2.0
1.0
0
7.5416
7.0999
7.0804
6.9591
6.9408
6.9339
6.5529
6.5346
6.2805
6.2610
5.7655
5.7438
5.0045
4.9633
4.2778
4.2377
3.7216
3.6747
3.6461
2.3632
2.3083
1.0
1.04
1.91
5.02
1.92
0.98
1
1.04
1.05
2.98
2.94
3.08
2.93

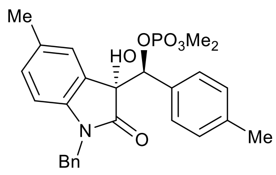
Me
OPO3Me2
HO
N
O
Me
Bn

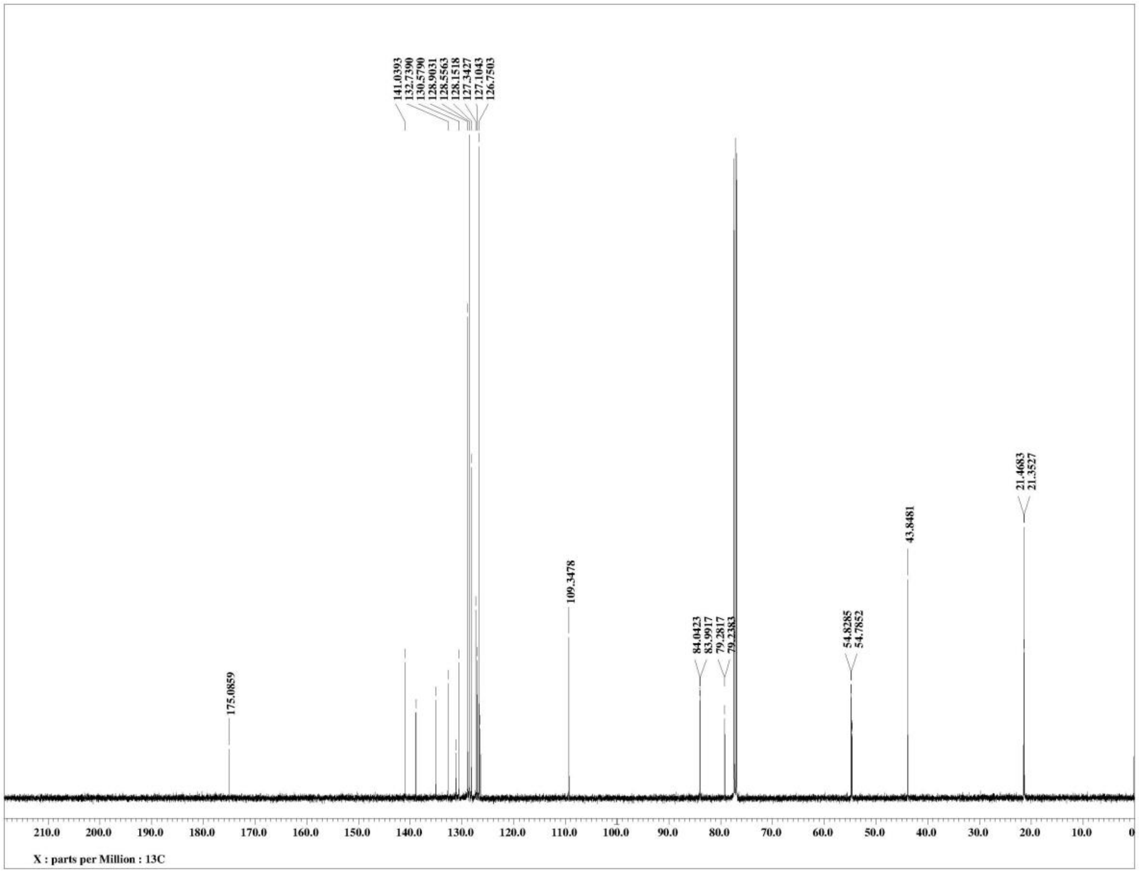
141.0393
132.7390
130.5790
128.9031
128.5563
128.1518
127.3427
127.1043
126.7503
175.0859
109.3478
84.0423
83.9917
79.2817
79.2383
54.8285
54.7852
43.8481
21.4683
21.3527
210.0
200.0
190.0
180.0
170.0
160.0
150.0
140.0
130.0
120.0
110.0
100.0
90.0
80.0
70.0
60.0
50.0
40.0
30.0
20.0
10.0
0
X : parts per Million : 13C

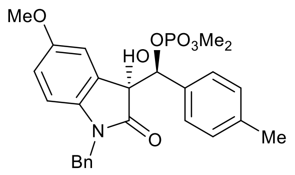
MeO
OPO3Me2
HO
N
O
Me
Bn

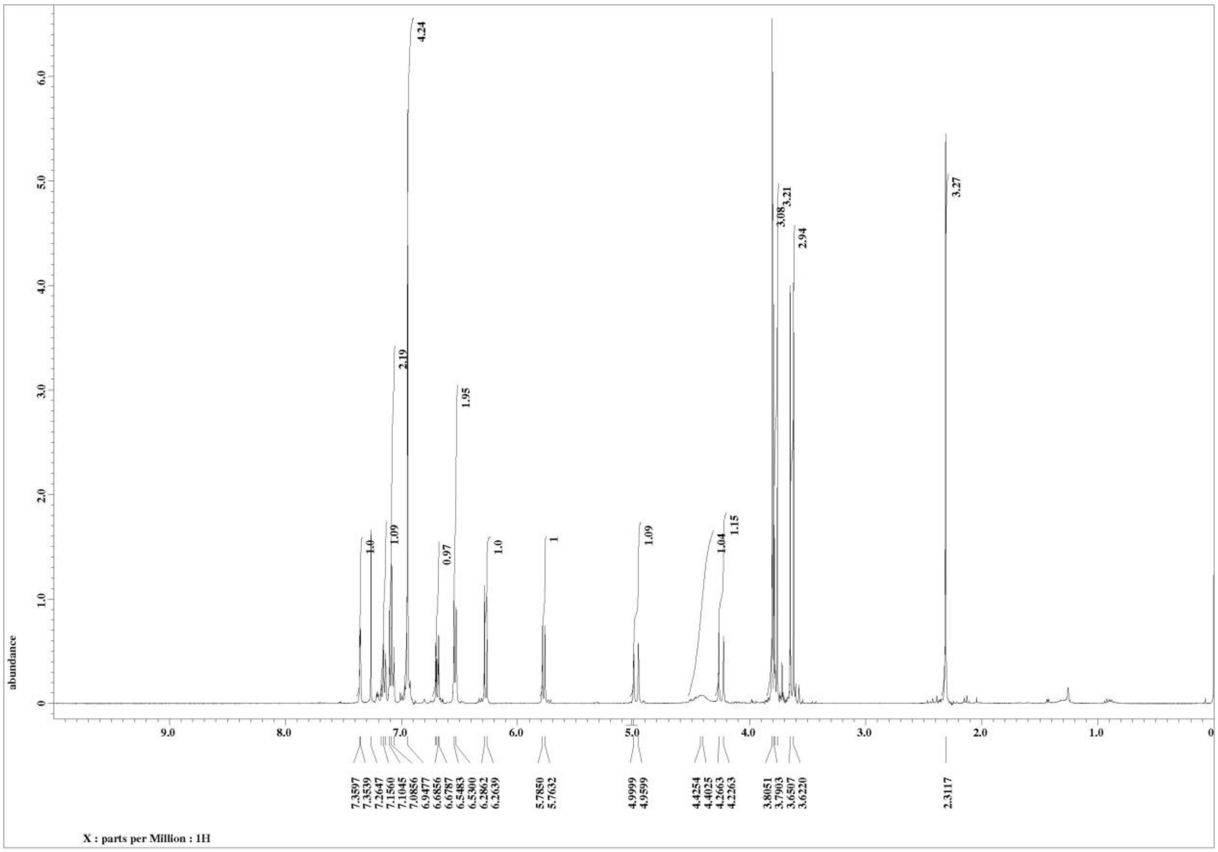
abundance
X : parts per Million : 1H

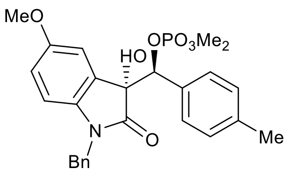
MeO
OPO3Me2
HO
N
O
Bn
Me

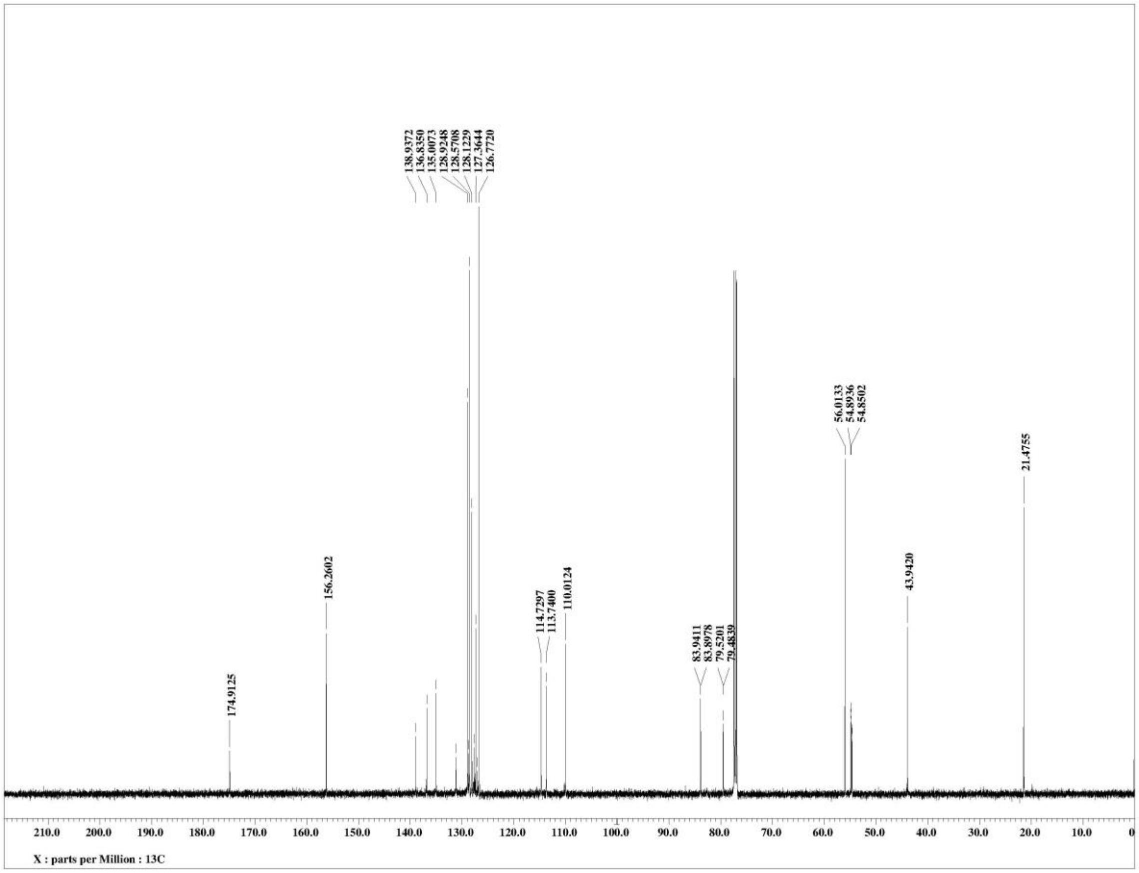
138.9372
136.8350
135.0073
128.9248
128.5708
128.1229
127.3644
126.7720
174.9125
156.2602
114.7297
113.7400
110.0124
83.9411
83.8978
79.5201
79.4839
56.0133
54.8936
54.8502
43.9420
21.4755
210.0
200.0
190.0
180.0
170.0
160.0
150.0
140.0
130.0
120.0
110.0
100.0
90.0
80.0
70.0
60.0
50.0
40.0
30.0
20.0
10.0
0
X : parts per Million : 13C

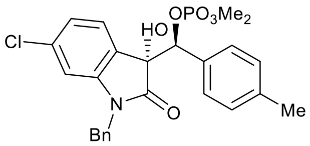
Cl
HO
OPO3Me2
N
O
Bn
Me

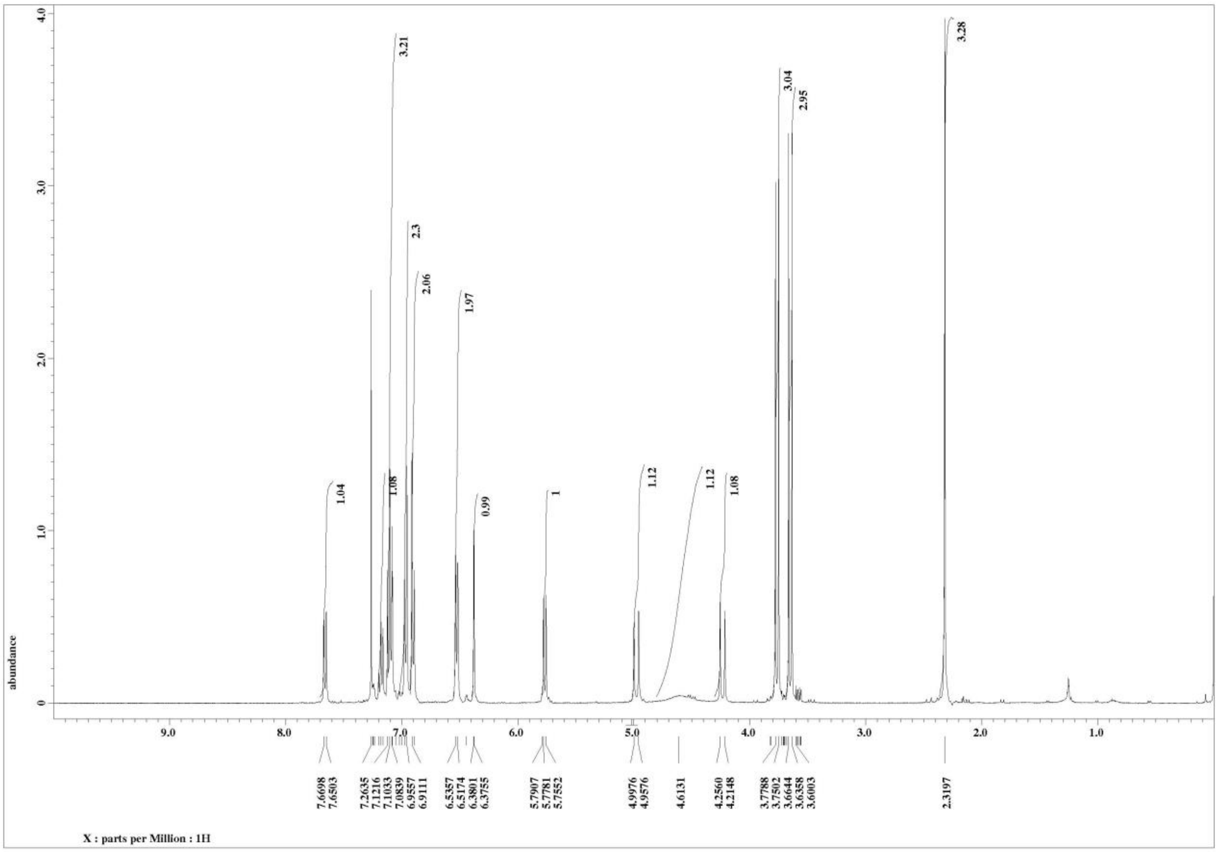
abundance
X : parts per Million : 1H
1.04
3.21
1.08
2.3
2.06
1.97
0.99
1
1.12
1.12
1.08
3.04
2.95
3.28
7.6698
7.6503
7.2635
7.1216
7.1033
7.0839
6.9557
6.9111
6.5357
6.5174
6.3801
6.3755
5.7907
5.7781
5.7552
4.9976
4.9576
4.6131
4.2560
4.2148
3.7788
3.7502
3.6644
3.6358
3.6003
2.3197

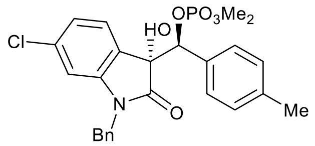
OPO3Me2
HO
Cl
N
O
Bn
Me

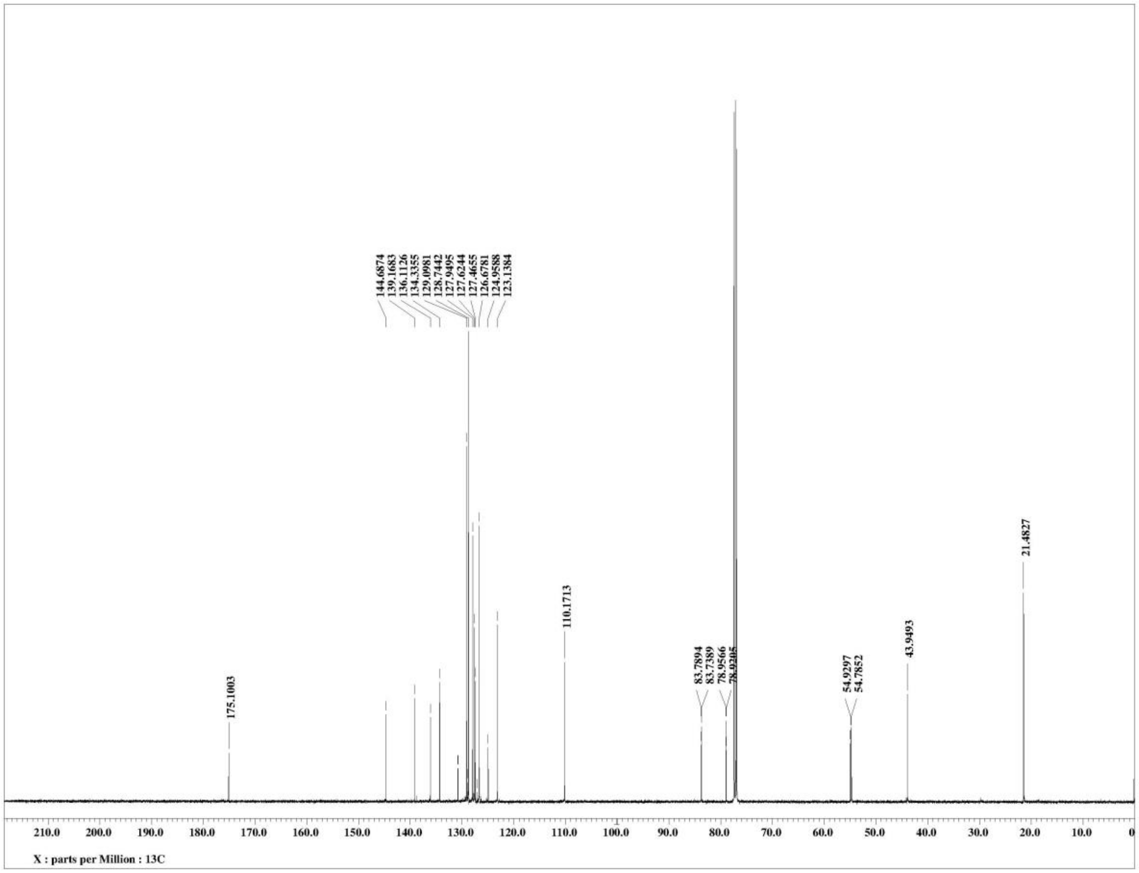
144.6874
139.1683
136.1126
134.3355
129.0981
128.7442
127.9495
127.6244
127.4655
126.6781
124.9588
123.1384
175.1003
110.1713
83.7894
83.7389
78.9566
78.9205
54.9297
54.7852
43.9493
21.4827
210.0 200.0 190.0 180.0 170.0 160.0 150.0 140.0 130.0 120.0 110.0 100.0 90.0 80.0 70.0 60.0 50.0 40.0 30.0 20.0 10.0 0
X : parts per Million : 13C

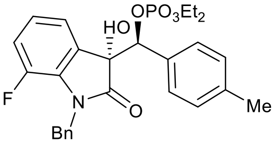

HO
OPO3Et2
F
N
O
Bn
Me

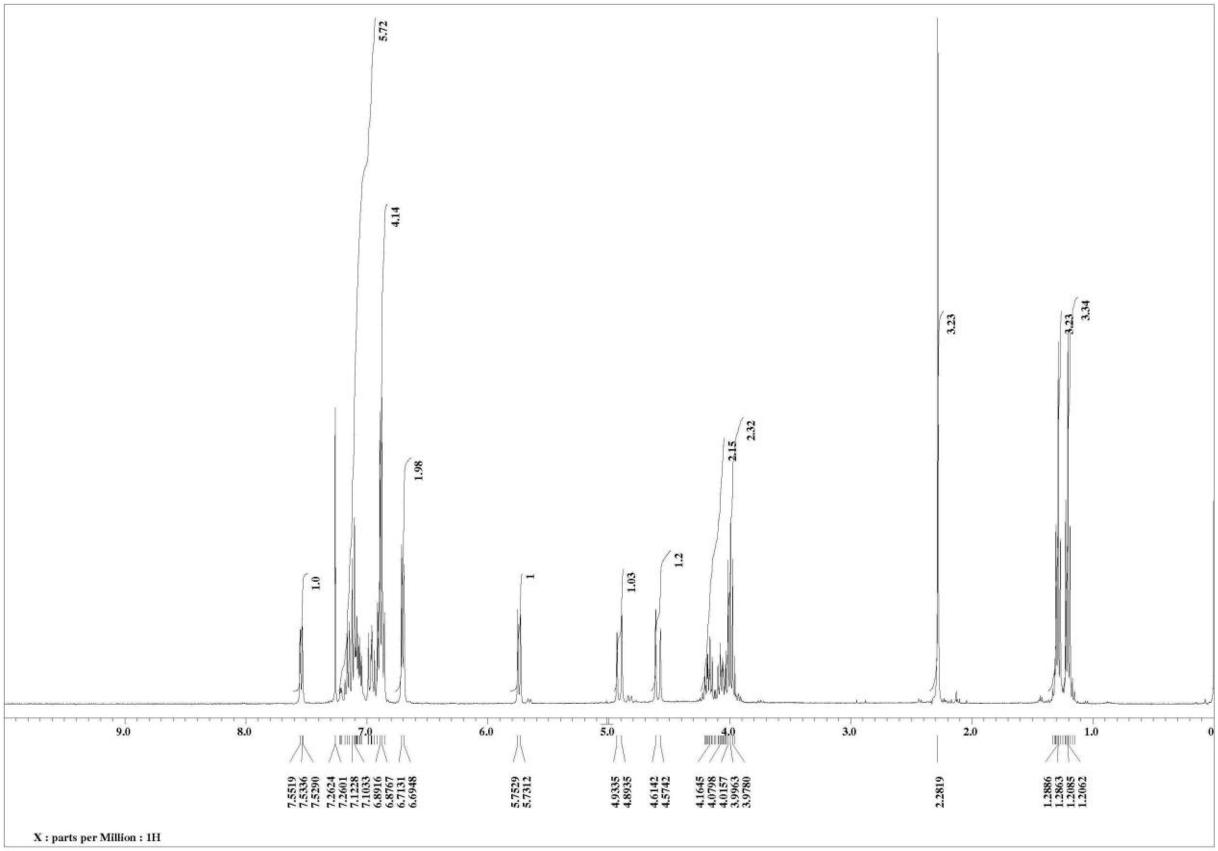

X : parts per Million : 1H

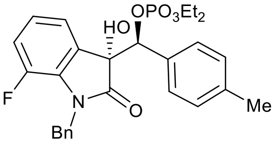
OPO3Et2
HO
F
N
O
Bn
Me

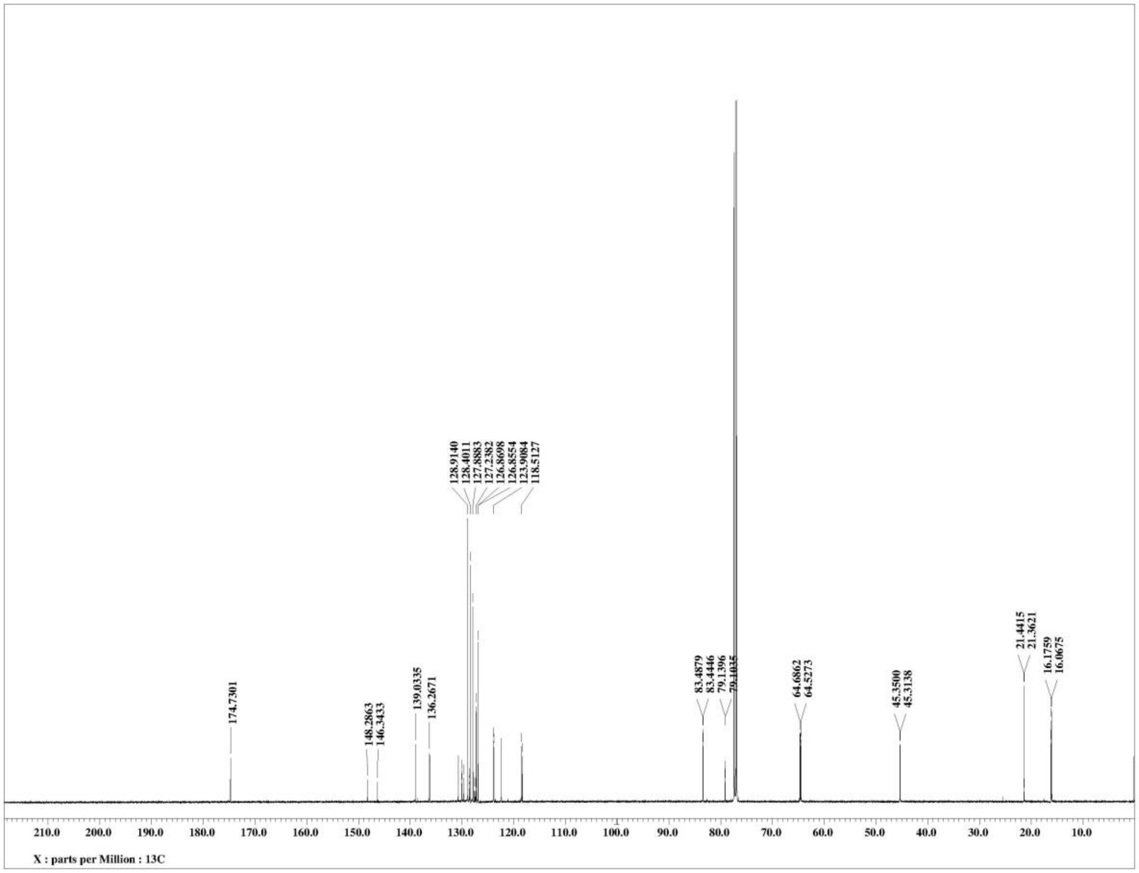
174.7301
148.2863
146.3433
139.0335
136.2671
128.9140
128.4011
127.8883
127.2382
126.8698
126.8554
123.9084
118.5127
83.4879
83.4446
79.1396
79.1035
64.6862
64.5273
45.3500
45.3138
21.4415
21.3621
16.1759
16.0675
210.0 200.0 190.0 180.0 170.0 160.0 150.0 140.0 130.0 120.0 110.0 100.0 90.0 80.0 70.0 60.0 50.0 40.0 30.0 20.0 10.0
X : parts per Million : 13C

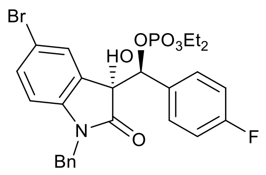

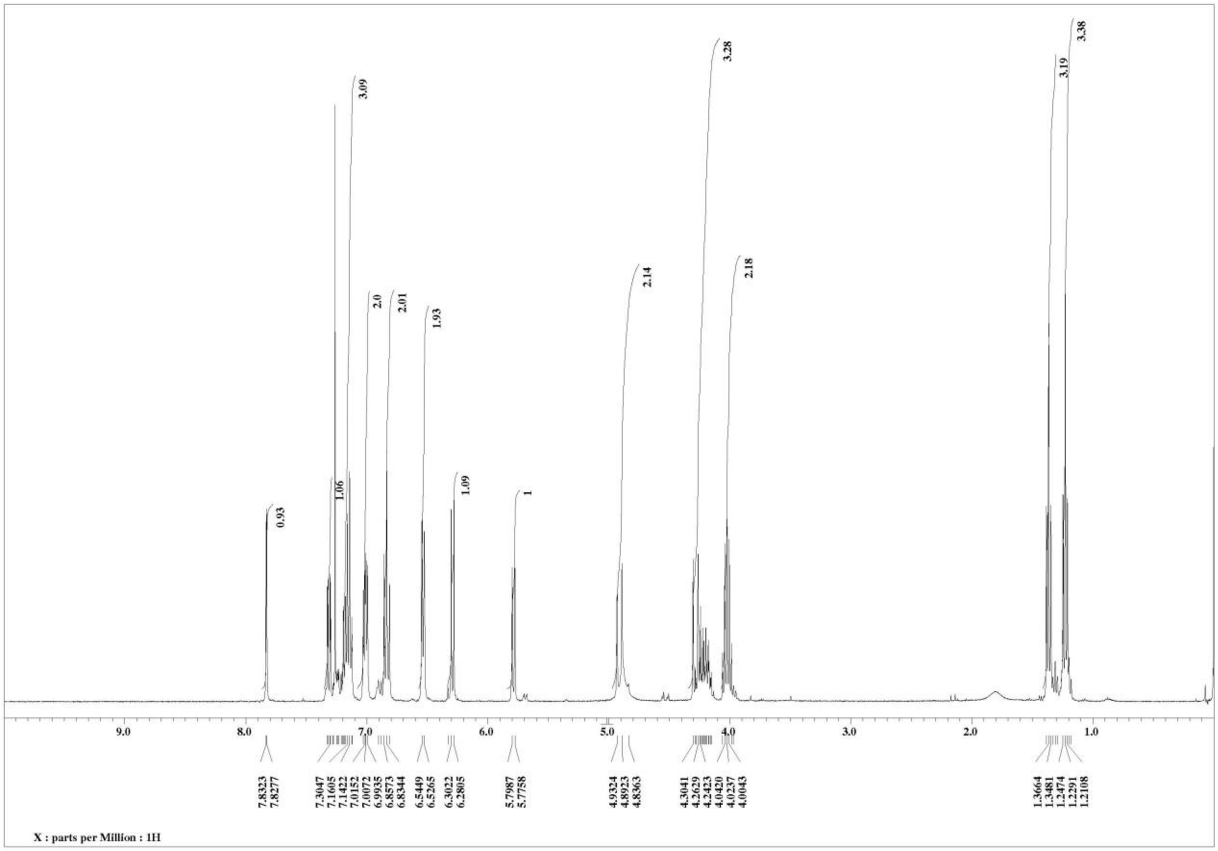
X : parts per Million : 1H

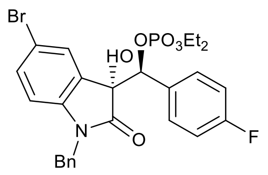
Br
OPO3Et2
HO
N
O
Bn
F

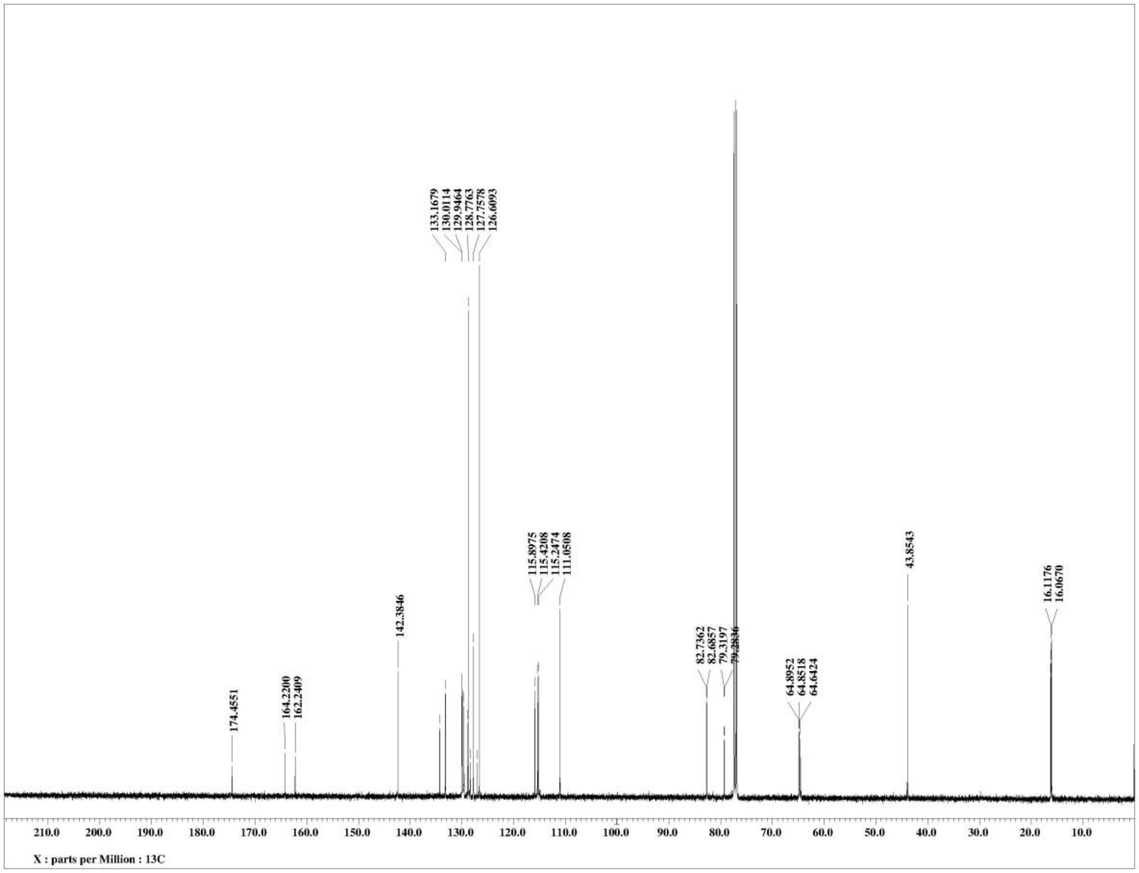
174.4551
164.2200
162.2409
142.3846
133.1679
130.0114
129.9464
128.7763
127.7578
126.6093
115.8975
115.4208
115.2474
111.0508
82.7362
82.6857
79.3197
79.2836
64.8952
64.8518
64.6424
43.8543
16.1176
16.0670
210.0 200.0 190.0 180.0 170.0 160.0 150.0 140.0 130.0 120.0 110.0 100.0 90.0 80.0 70.0 60.0 50.0 40.0 30.0 20.0 10.0
X : parts per Million : 13C

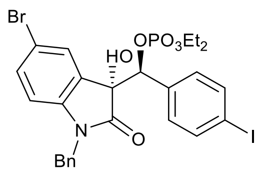
Br
OPO3Et2
HO
N
O
Bn
I

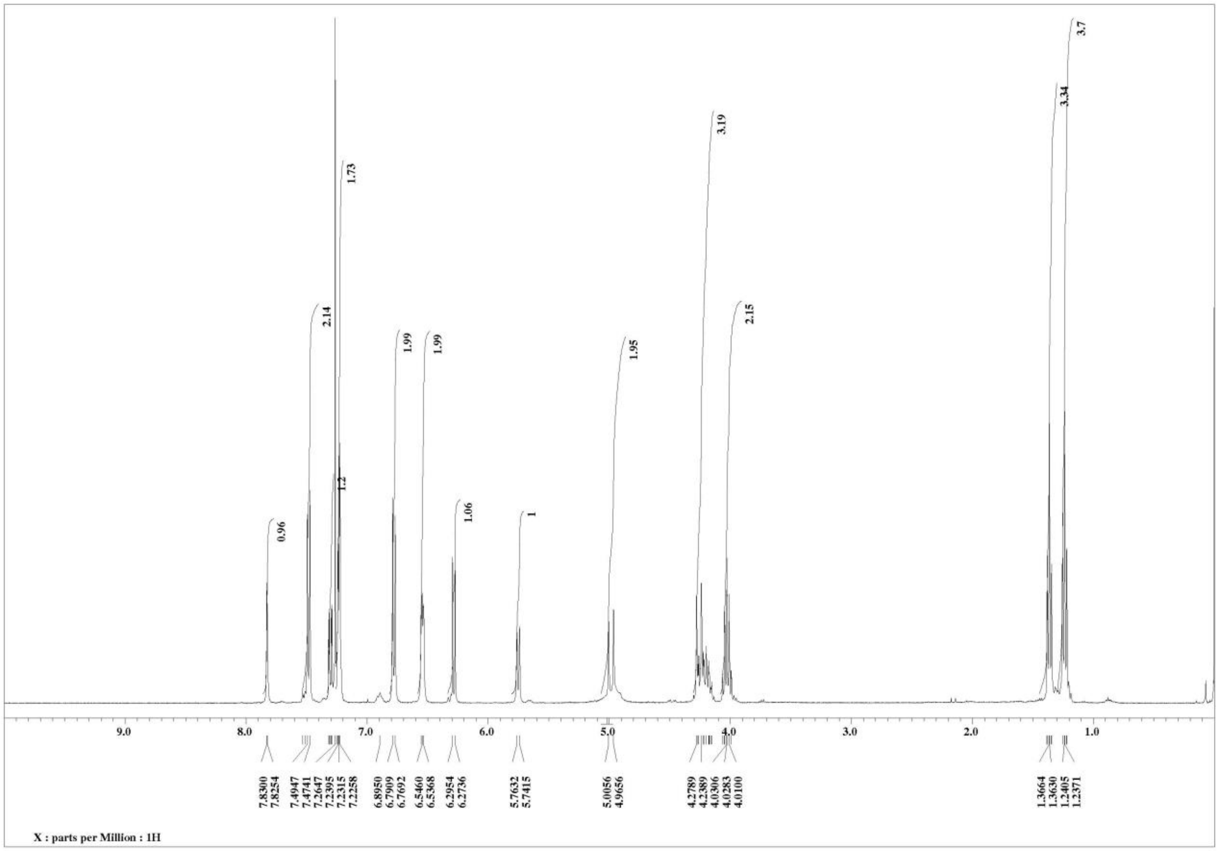
0.96
2.14
1.2
1.73
1.99
1.99
1.06
1
1.95
3.19
2.15
3.34
3.7
9.0
8.0
7.0
6.0
5.0
4.0
3.0
2.0
1.0
7.8300
7.8254
7.4947
7.4741
7.2647
7.2395
7.2315
7.2258
6.8950
6.7909
6.7692
6.5460
6.5368
6.2954
6.2736
5.7632
5.7415
5.0056
4.9656
4.2789
4.2389
4.0306
4.0283
4.0100
1.3664
1.3630
1.2405
1.2371
X : parts per Million : 1H

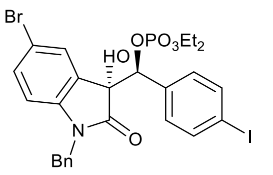
Br
OPO3Et2
HO
N
O
Bn
I

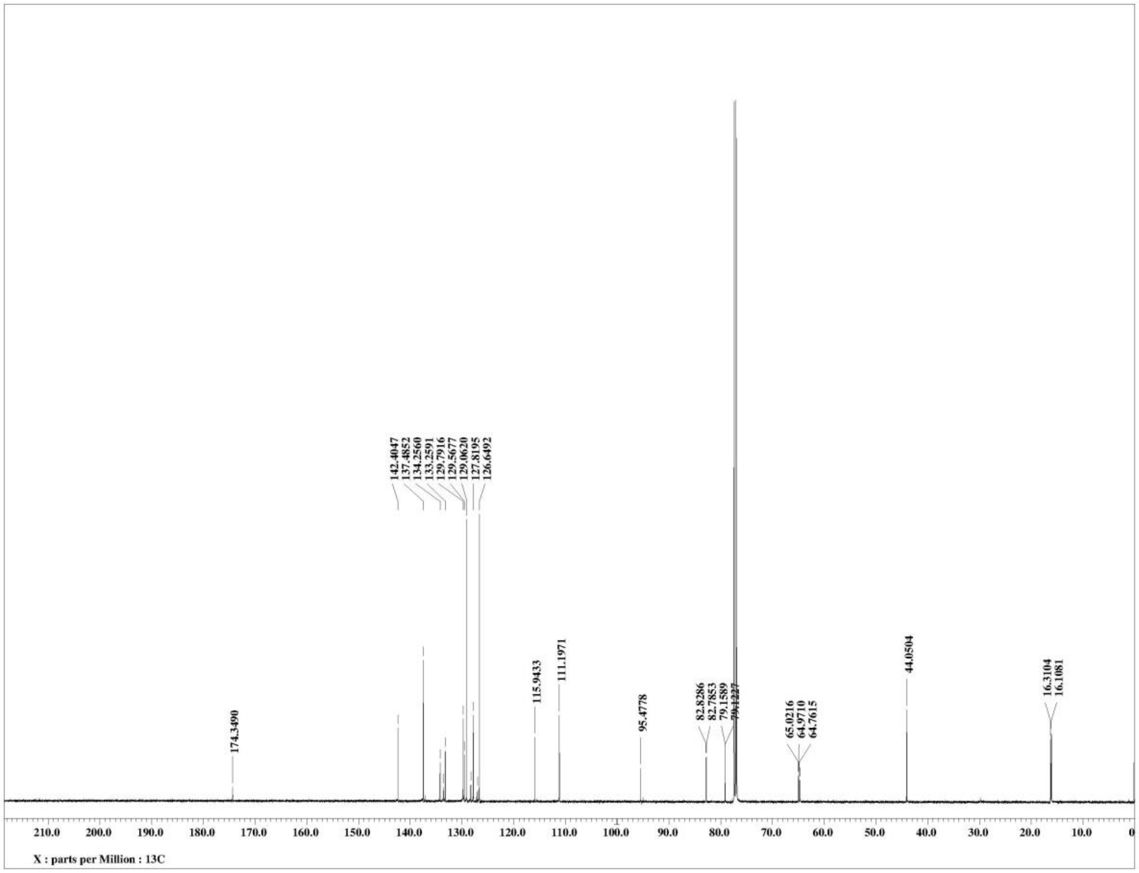
174.3490
142.4047
137.4852
134.2560
133.2591
129.7916
129.5677
129.0620
127.8195
126.6492
115.9433
111.1971
95.4778
82.8286
82.7853
79.1589
79.1227
65.0216
64.9710
64.7615
44.0504
16.3104
16.1081
210.0 200.0 190.0 180.0 170.0 160.0 150.0 140.0 130.0 120.0 110.0 100.0 90.0 80.0 70.0 60.0 50.0 40.0 30.0 20.0 10.0 0
X : parts per Million : 13C

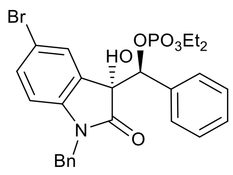
Br
OPO3Et2
HO
N
O
Bn

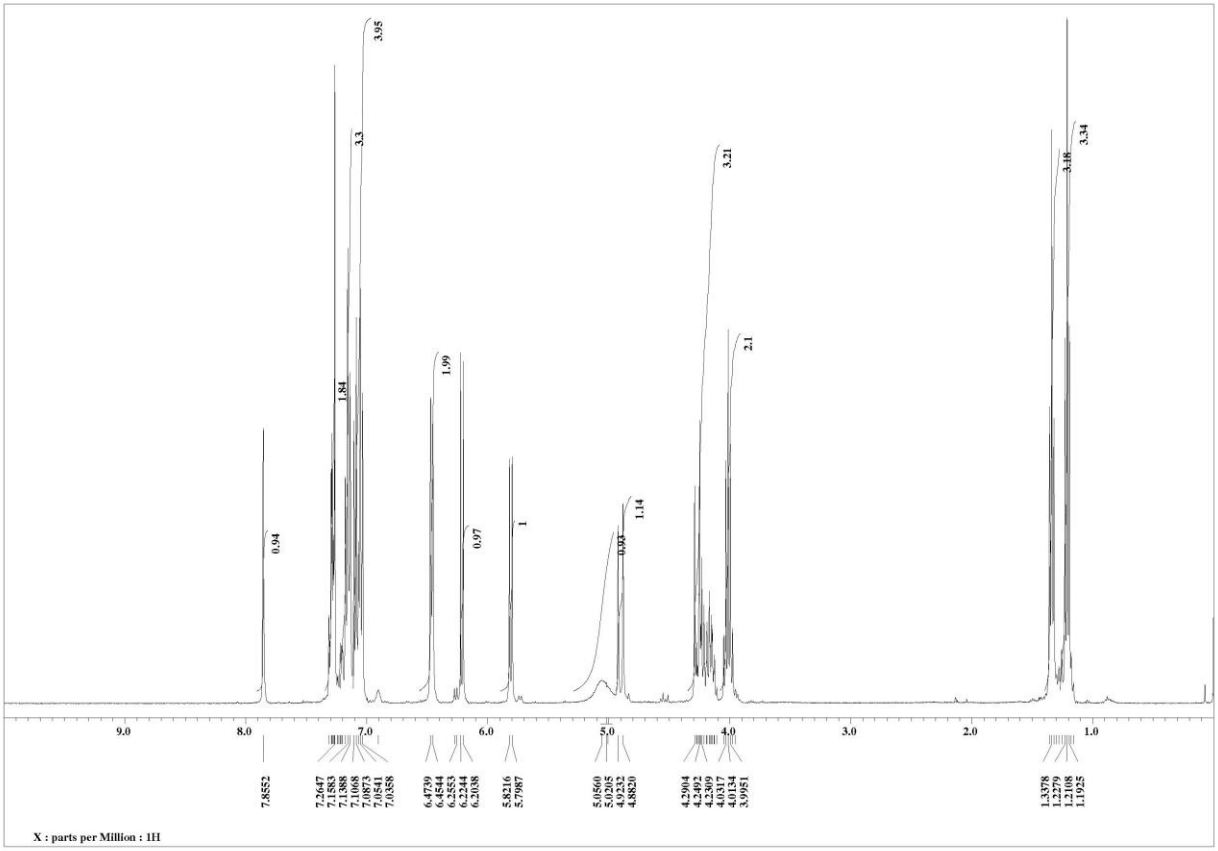
X : parts per Million : 1H

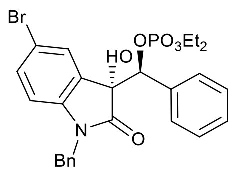

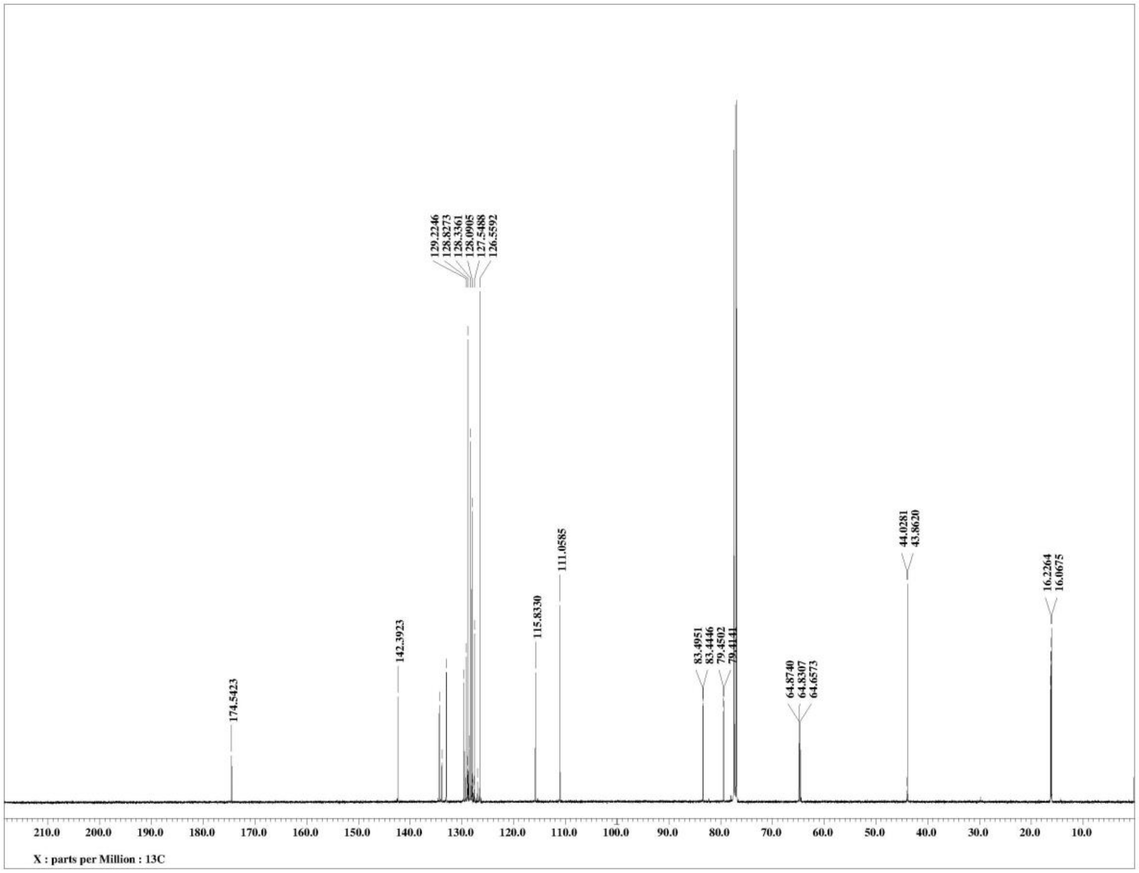

174.5423
142.3923
129.2246
128.8273
128.3361
128.0905
127.5488
126.5592
115.8330
111.0585
83.4951
83.4446
79.4502
79.4141
64.8740
64.8307
64.6573
44.0281
43.8620
16.2264
16.0675
210.0 200.0 190.0 180.0 170.0 160.0 150.0 140.0 130.0 120.0 110.0 100.0 90.0 80.0 70.0 60.0 50.0 40.0 30.0 20.0 10.0
X : parts per Million : 13C

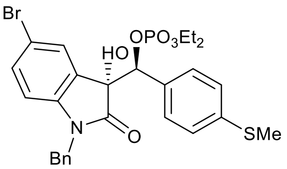
Br
OPO3Et2
HO
N
O
Bn
SMe

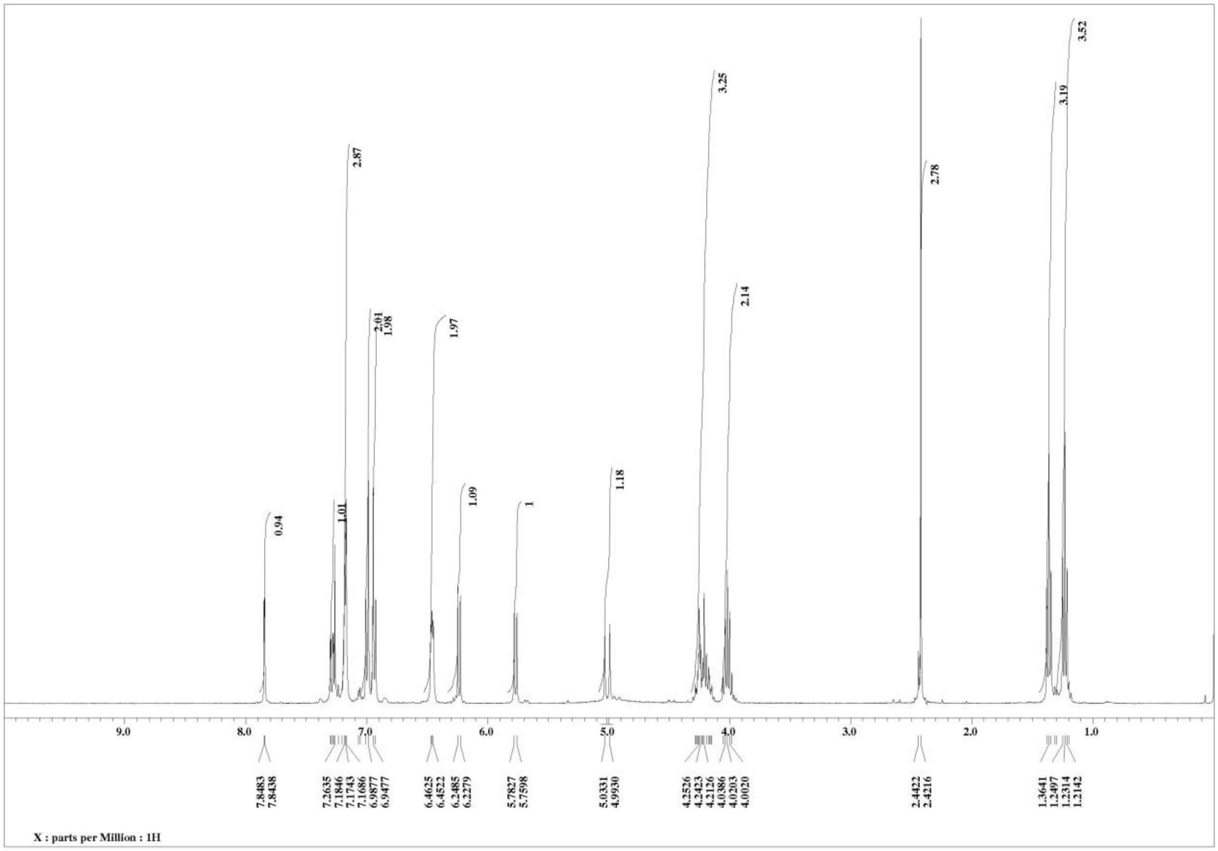
0.94
1.01
2.87
2.01
1.98
1.97
1.09
1
1.18
3.25
2.14
2.78
3.19
3.52
9.0
8.0
7.0
6.0
5.0
4.0
3.0
2.0
1.0
7.8483
7.8438
7.2635
7.1846
7.1743
7.1686
6.9877
6.9477
6.4625
6.4522
6.2485
6.2279
5.7827
5.7598
5.0331
4.9930
4.2526
4.2423
4.2126
4.0386
4.0203
4.0020
2.4422
2.4216
1.3641
1.2497
1.2314
1.2142
X : parts per Million : 1H

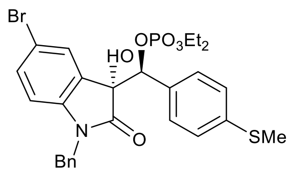
Br
OPO3Et2
HO
N
O
Bn
SMe

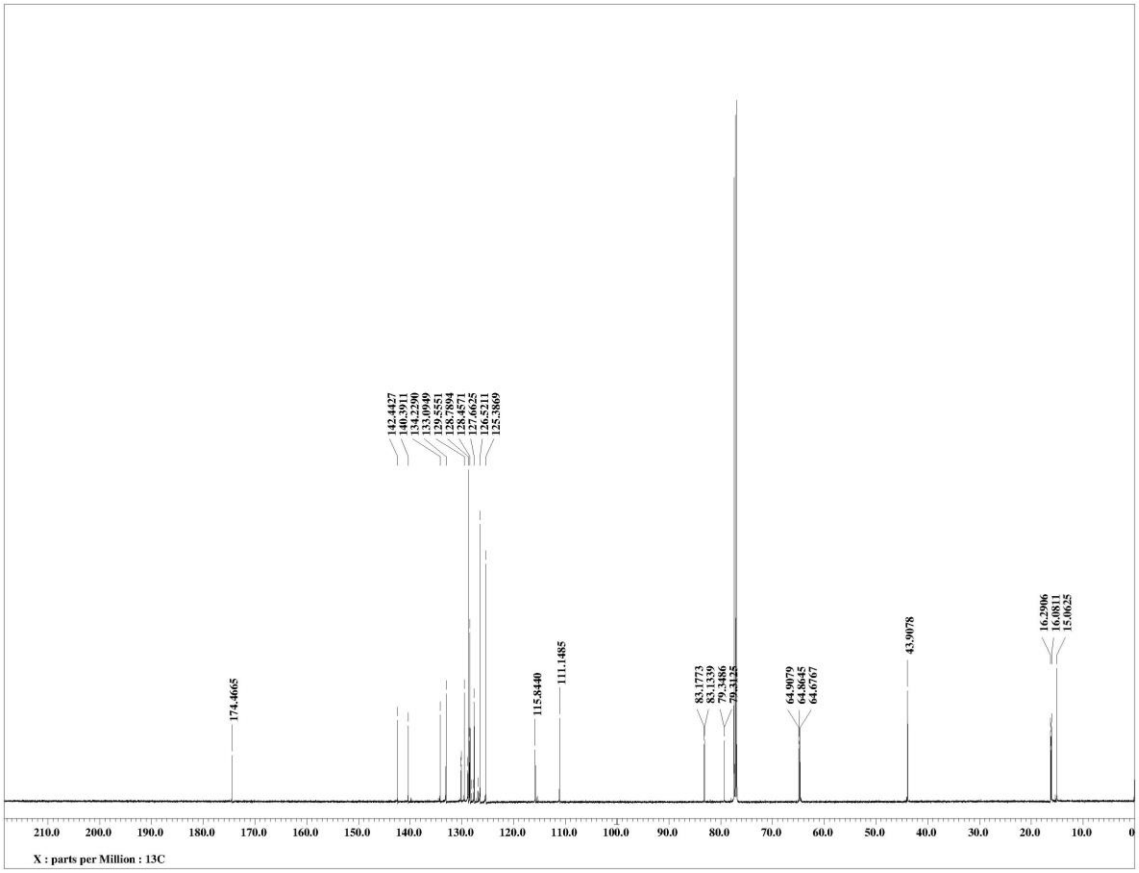
142.4427
140.3911
134.2290
133.0949
129.5551
128.7894
128.4571
127.6625
126.5211
125.3869
174.4665
115.8440
111.1485
83.1773
83.1339
79.3486
79.3125
64.9079
64.8645
64.6767
43.9078
16.2906
16.0811
15.0625
210.0
200.0
190.0
180.0
170.0
160.0
150.0
140.0
130.0
120.0
110.0
100.0
90.0
80.0
70.0
60.0
50.0
40.0
30.0
20.0
10.0
0
X : parts per Million : 13C

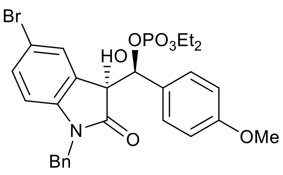

Br
OPO3Et2
HO
N
O
OMe
Bn

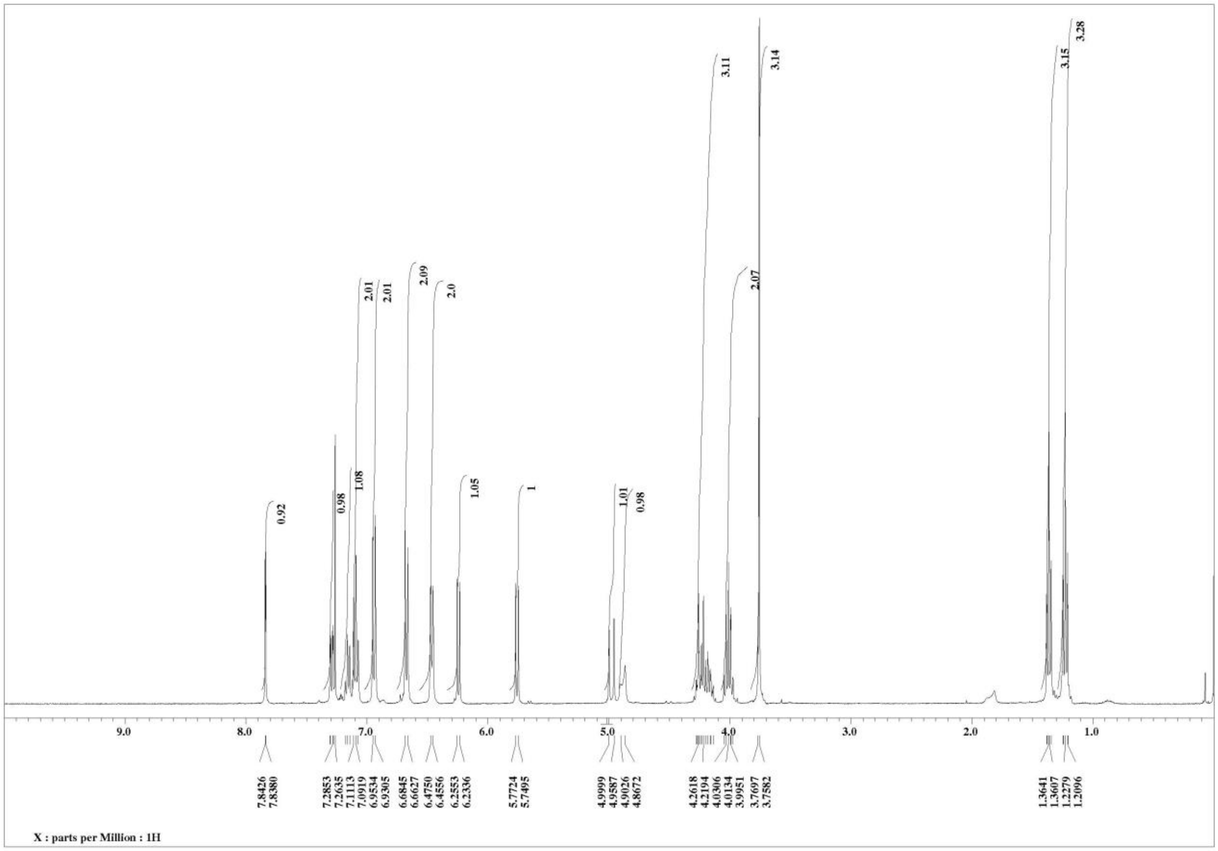

0.92
0.98
1.08
2.01
2.01
2.09
2.0
1.05
1
1.01
0.98
3.11
2.07
3.14
3.15
3.28
9.0
8.0
7.0
6.0
5.0
4.0
3.0
2.0
1.0
7.8426
7.8380
7.2853
7.2635
7.1113
7.0919
6.9534
6.9305
6.6845
6.6627
6.4750
6.4556
6.2553
6.2336
5.7724
5.7495
4.9999
4.9587
4.9026
4.8672
4.2618
4.2194
4.0306
4.0134
3.9951
3.7697
3.7582
1.3641
1.3607
1.2279
1.2096
X : parts per Million : 1H

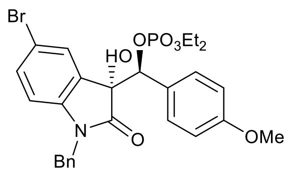

Br
OPO3Et2
HO
N
O
Bn
OMe

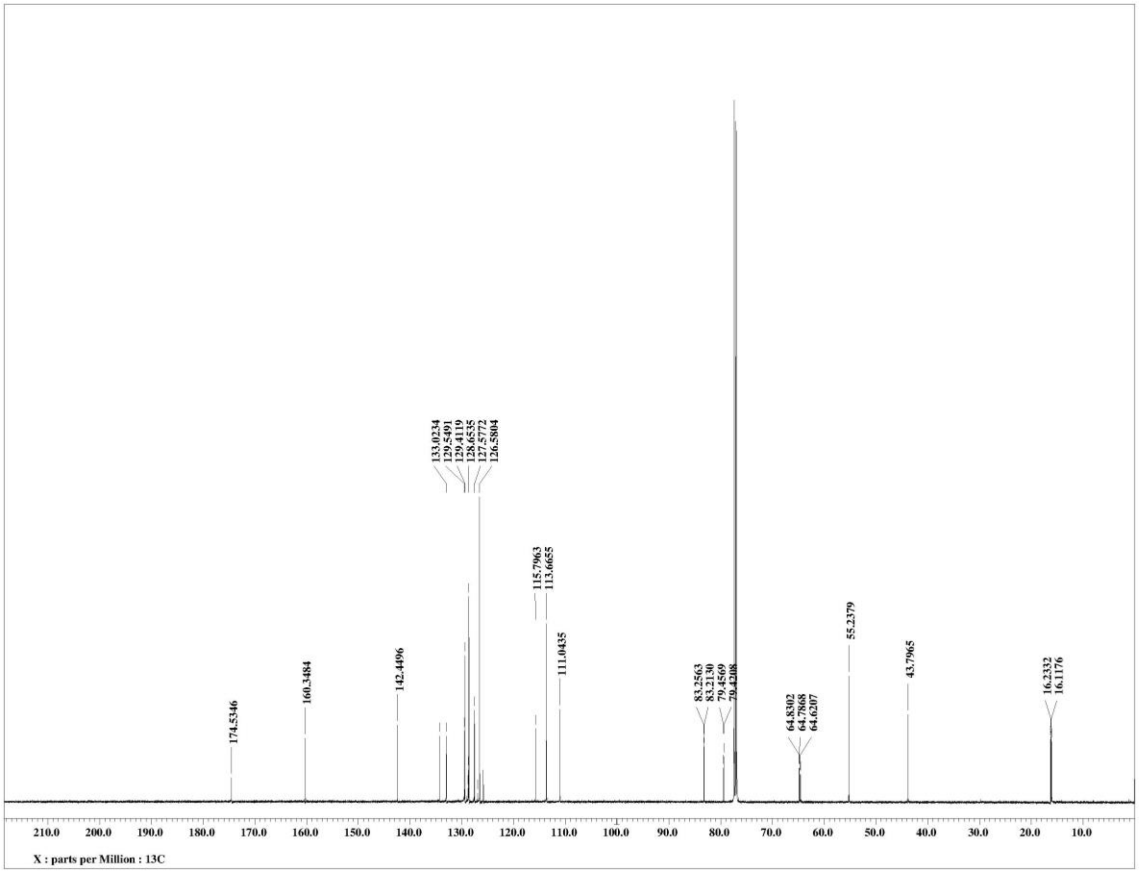

174.5346
160.3484
142.4496
133.0234
129.5491
129.4119
128.6535
127.5772
126.5804
115.7963
113.6655
111.0435
83.2563
83.2130
79.4569
79.4208
64.8302
64.7868
64.6207
55.2379
43.7965
16.2332
16.1176
210.0 200.0 190.0 180.0 170.0 160.0 150.0 140.0 130.0 120.0 110.0 100.0 90.0 80.0 70.0 60.0 50.0 40.0 30.0 20.0 10.0
X : parts per Million : 13C

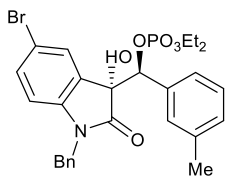
Br
OPO3Et2
HO
N
O
Bn
Me

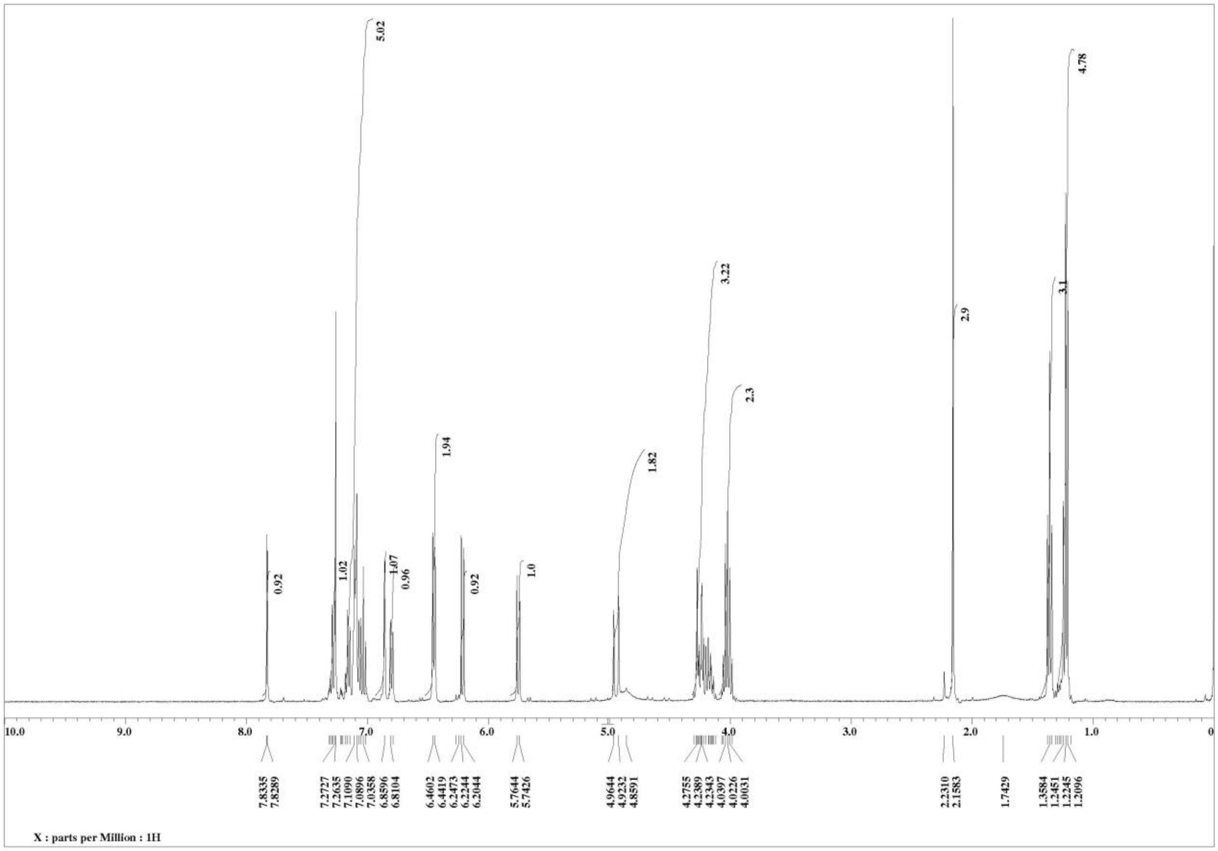
X : parts per Million : 1H

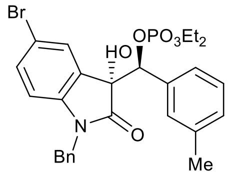

Br
OPO3Et2
HO
N
O
Bn
Me

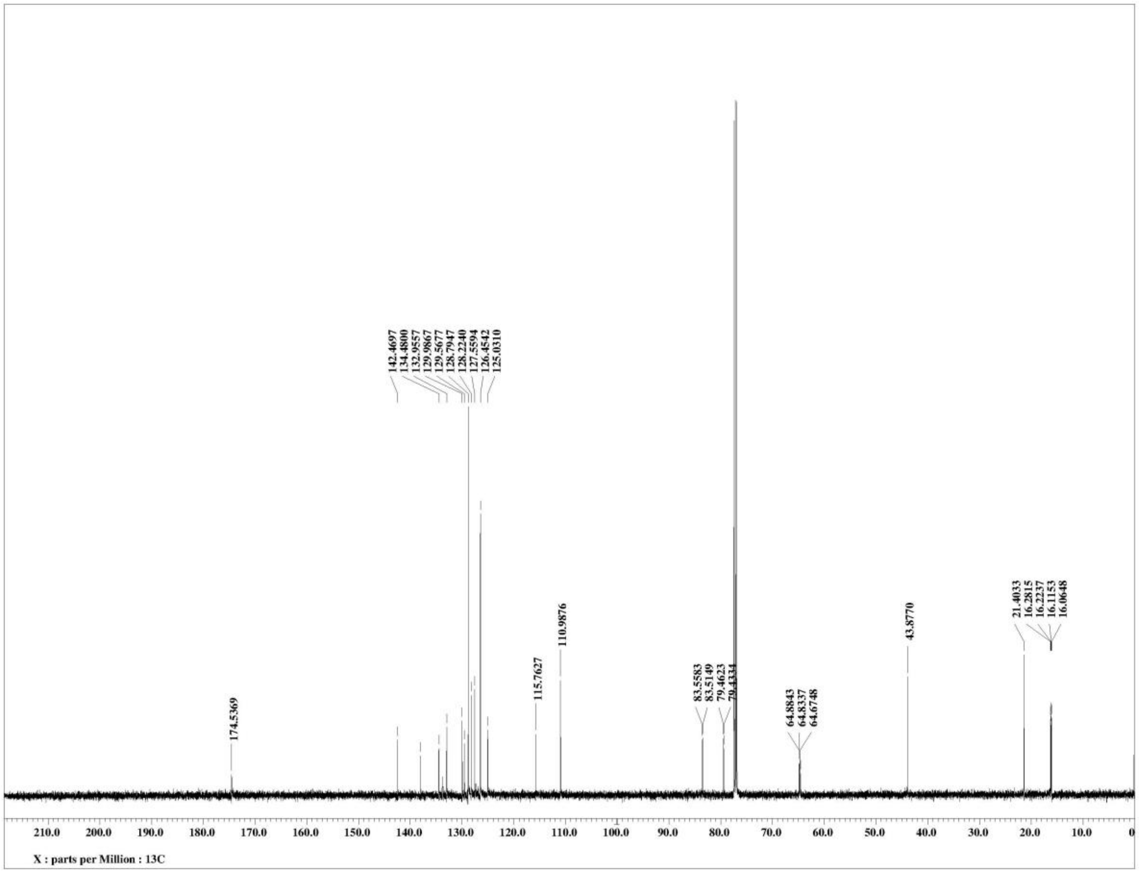

142.4697
134.4800
132.9557
129.9867
129.5677
128.7947
128.2240
127.5594
126.4542
125.0310
174.5369
115.7627
110.9876
83.5583
83.5149
79.4623
79.4334
64.8843
64.8337
64.6748
43.8770
21.4033
16.2815
16.2237
16.1153
16.0648
210.0
200.0
190.0
180.0
170.0
160.0
150.0
140.0
130.0
120.0
110.0
100.0
90.0
80.0
70.0
60.0
50.0
40.0
30.0
20.0
10.0
0
X : parts per Million : 13C

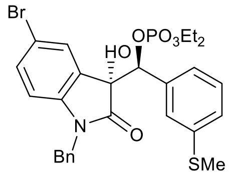
Br
OPO3Et2
HO
N
O
Bn
SMe

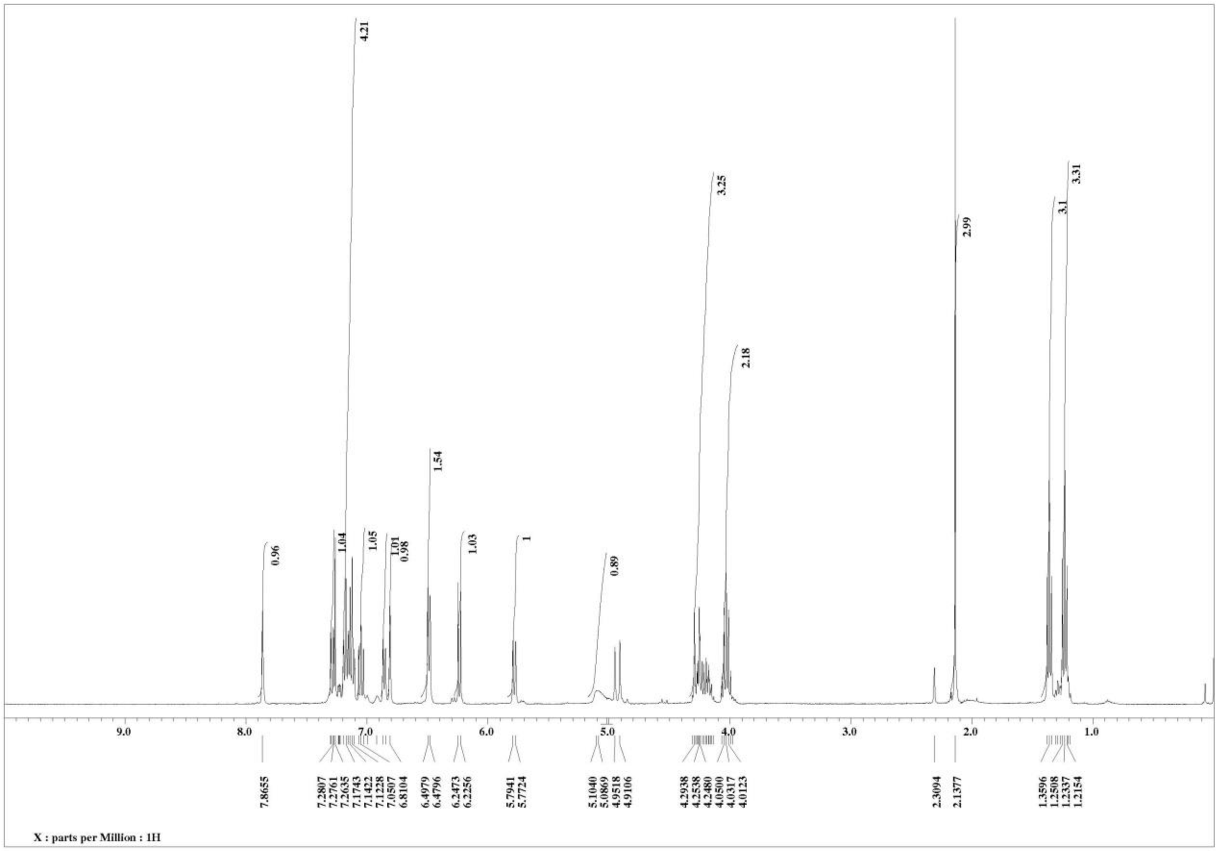
4.21
3.25
3.31
3.1
2.99
2.18
1.54
0.96
1.04
1.05
1.01
0.98
1.03
1
0.89
9.0
8.0
7.0
6.0
5.0
4.0
3.0
2.0
1.0
7.8655
7.2807
7.2761
7.2635
7.1743
7.1422
7.1228
7.0507
6.8104
6.4979
6.4796
6.2473
6.2256
5.7941
5.7724
5.1040
5.0869
4.9518
4.9106
4.2938
4.2538
4.2480
4.0500
4.0317
4.0123
2.3094
2.1377
1.3596
1.2508
1.2337
1.2154
X : parts per Million : 1H

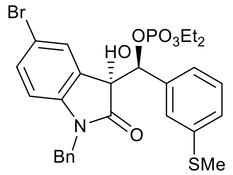

Br
OPO3Et2
HO
N
O
Bn
SMe

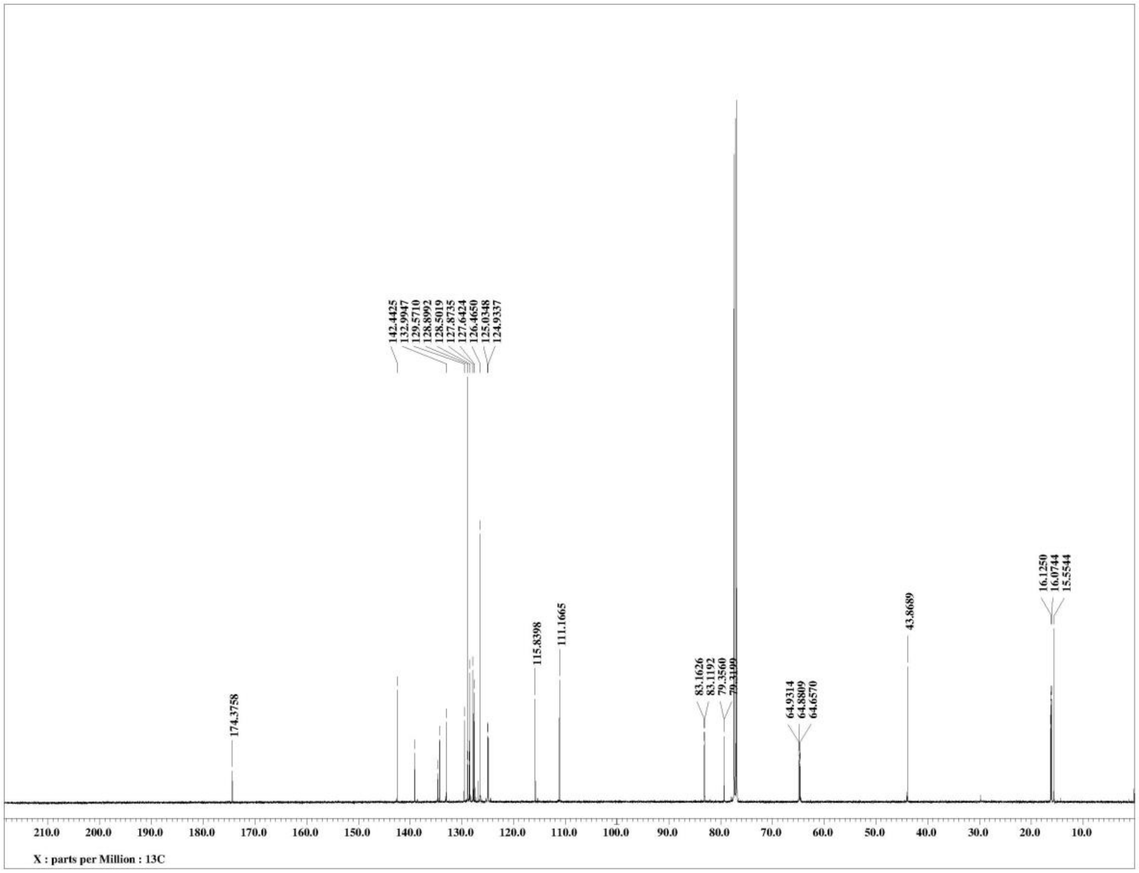

142.4425
132.9947
129.5710
128.8992
128.5019
127.8735
127.6424
126.4650
125.0348
124.9337
174.3758
115.8398
111.1665
83.1626
83.1192
79.3560
79.3199
64.9314
64.8809
64.6570
43.8689
16.1250
16.0744
15.5544
210.0
200.0
190.0
180.0
170.0
160.0
150.0
140.0
130.0
120.0
110.0
100.0
90.0
80.0
70.0
60.0
50.0
40.0
30.0
20.0
10.0
X : parts per Million : 13C

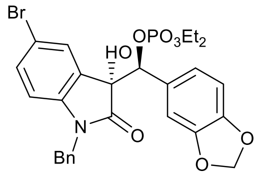

Br
OPO3Et2
HO
N
O
Bn
O
O

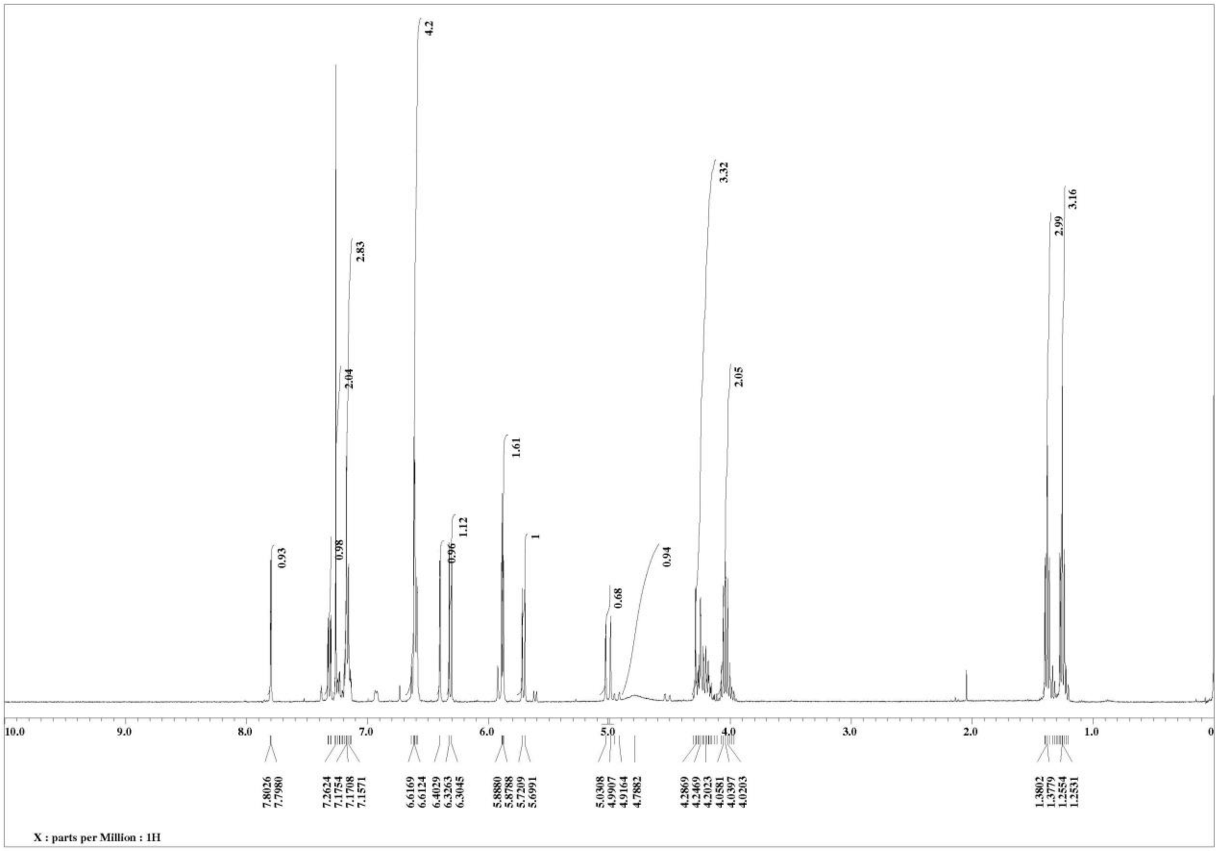

X : parts per Million : 1H

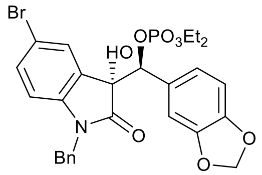
Br
OPO3Et2
HO
N
O
Bn
O
O

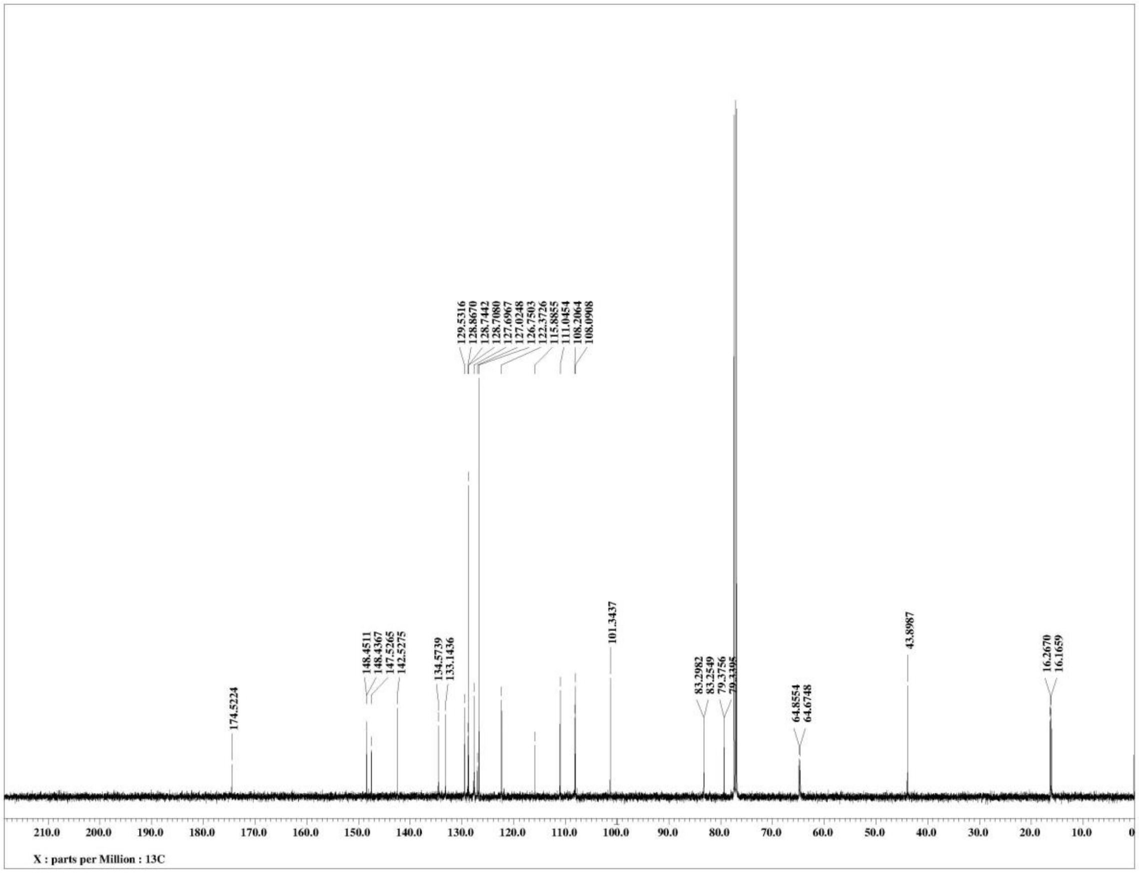
174.5224
148.4511
148.4367
147.5265
142.5275
134.5739
133.1436
129.5316
128.8670
128.7442
128.7080
127.6967
127.0248
126.7503
122.3726
115.8855
111.0454
108.2064
108.0908
101.3437
83.2982
83.2549
79.3756
64.8554
64.6748
43.8987
16.2670
16.1659
210.0
200.0
190.0
180.0
170.0
160.0
150.0
140.0
130.0
120.0
110.0
100.0
90.0
80.0
70.0
60.0
50.0
40.0
30.0
20.0
10.0
0
X : parts per Million : 13C

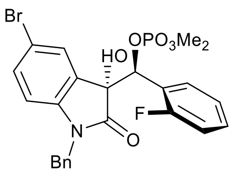

Br
OPO3Me2
HO
F
N
O
Bn

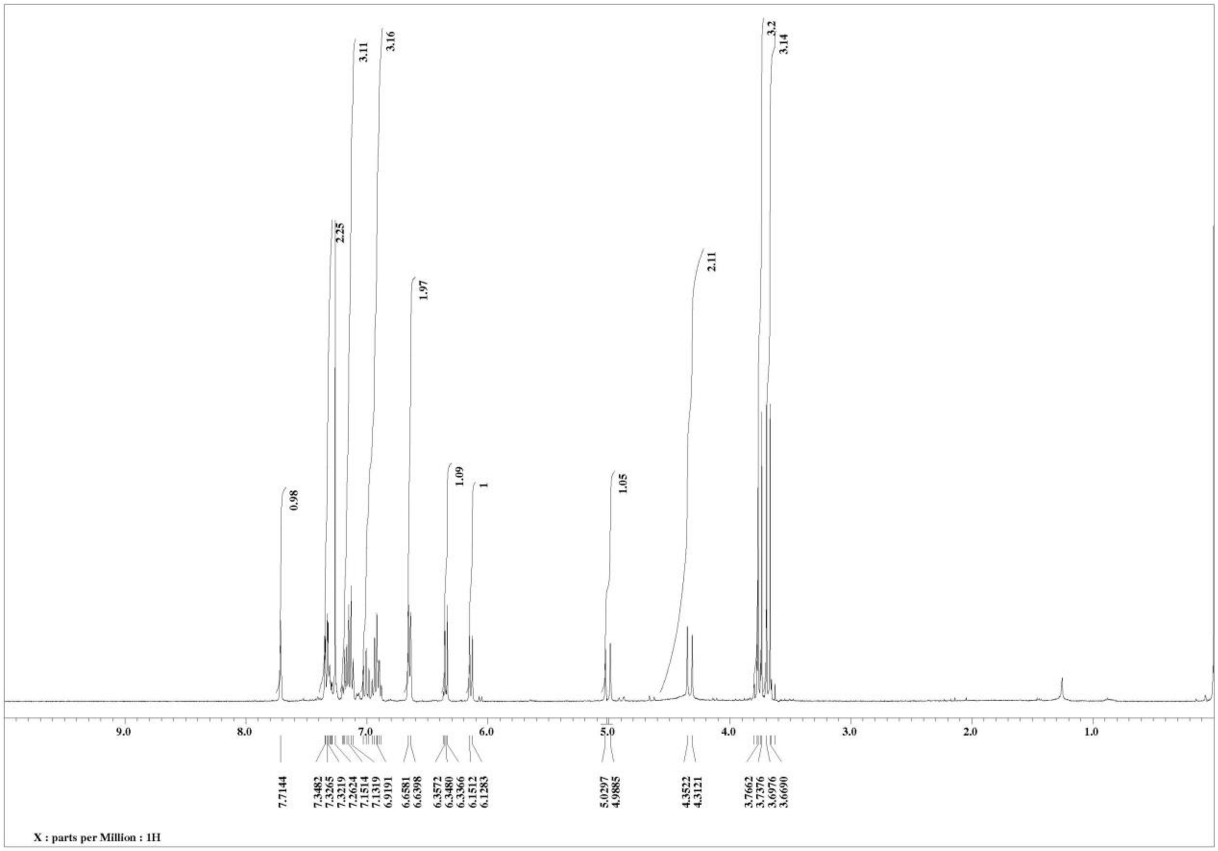

X : parts per Million : 1H

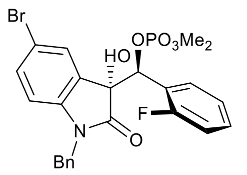
Br
OPO3Me2
HO
F
N
O
Bn

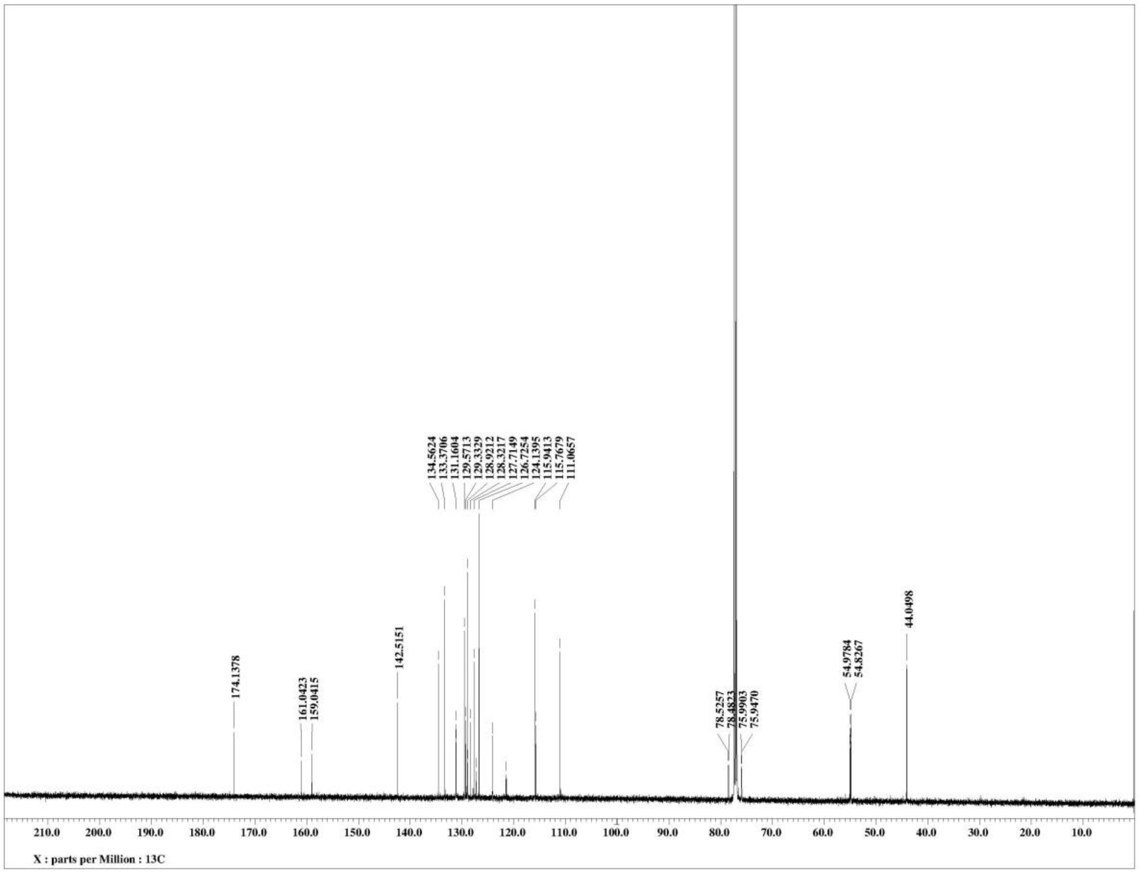
134.5624
133.3706
131.1604
129.5713
129.3329
128.9212
128.3217
127.7149
126.7254
124.1395
115.9413
115.7679
111.0657
174.1378
161.0423
159.0415
142.5151
78.5257
78.4823
75.9903
75.9470
54.9784
54.8267
44.0498
210.0
200.0
190.0
180.0
170.0
160.0
150.0
140.0
130.0
120.0
110.0
100.0
90.0
80.0
70.0
60.0
50.0
40.0
30.0
20.0
10.0
X : parts per Million : 13C

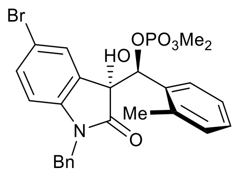

Br
OPO3Me2
HO
Me
N
O
Bn

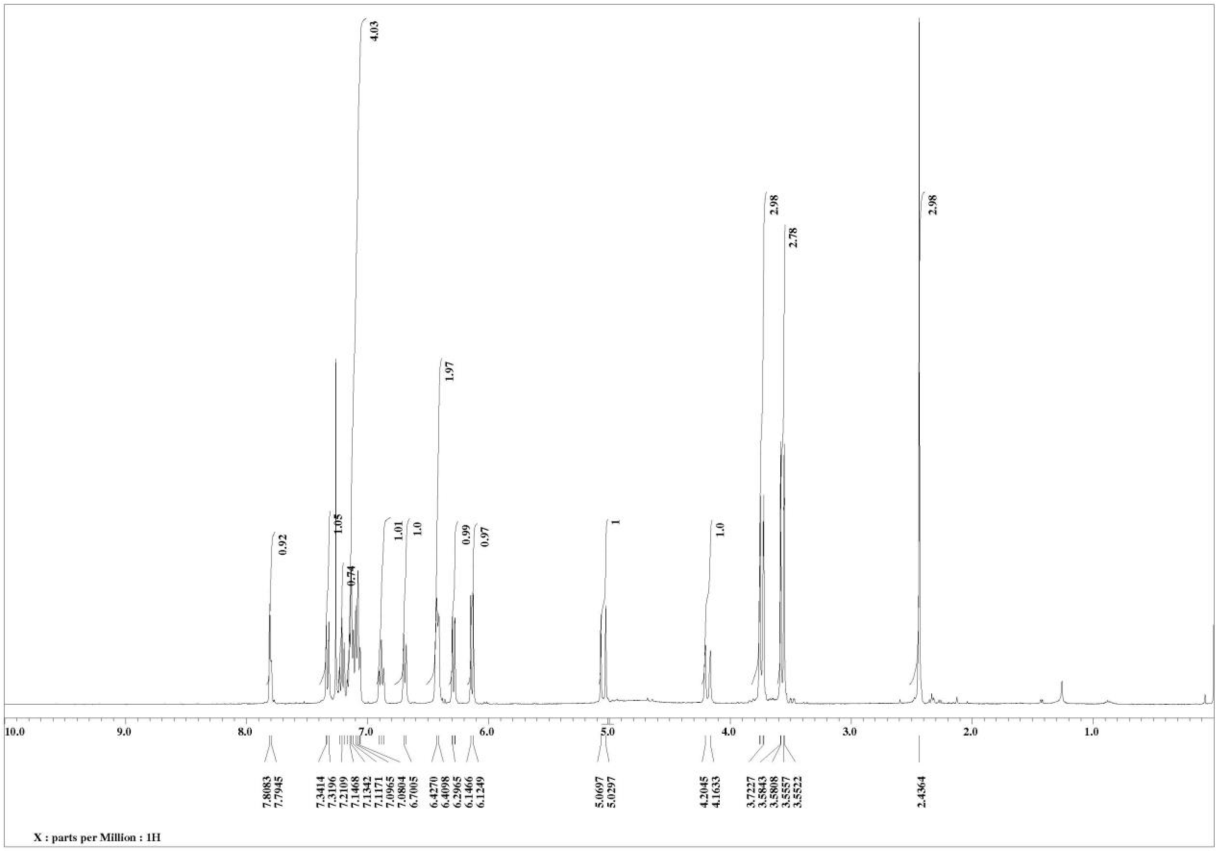

X : parts per Million : 1H

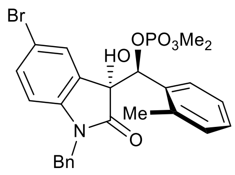
Br
OPO3Me2
HO
Me
N
O
Bn

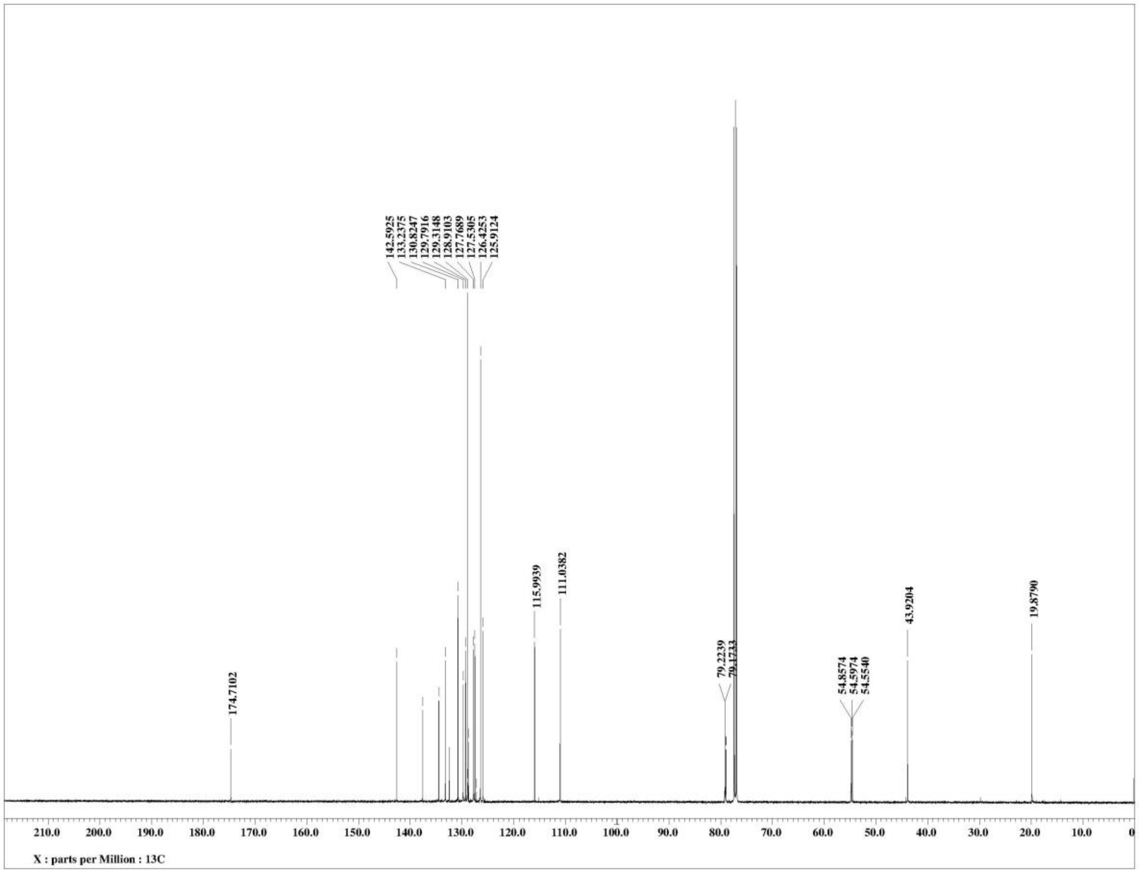
142.5925
133.2375
130.8247
129.7916
129.3148
128.9103
127.7689
127.5305
126.4253
125.9124
174.7102
115.9939
111.0382
79.2239
79.1733
54.8574
54.5974
54.5540
43.9204
19.8790
210.0 200.0 190.0 180.0 170.0 160.0 150.0 140.0 130.0 120.0 110.0 100.0 90.0 80.0 70.0 60.0 50.0 40.0 30.0 20.0 10.0 0
X : parts per Million : 13C

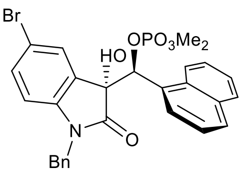

Br
OPO3Me2
HO
N
O
Bn

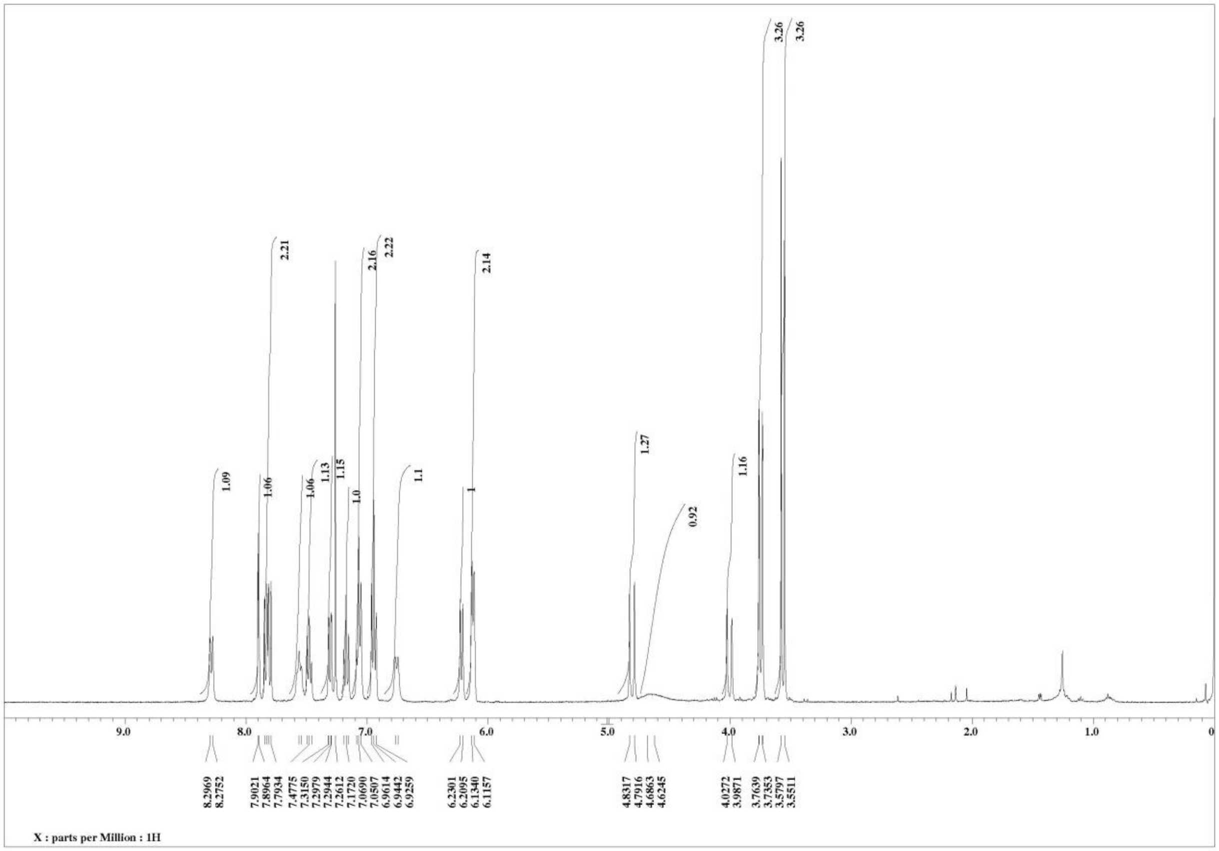

1.09
1.06
2.21
1.06
1.13
1.15
1.0
2.16
2.22
1.1
1
2.14
1.27
0.92
1.16
3.26
3.26
9.0
8.0
7.0
6.0
5.0
4.0
3.0
2.0
1.0
0
8.2969
8.2752
7.9021
7.8964
7.7934
7.4775
7.3150
7.2979
7.2944
7.2612
7.1720
7.0690
7.0507
6.9614
6.9442
6.9259
6.2301
6.2095
6.1340
6.1157
4.8317
4.7916
4.6863
4.6245
4.0272
3.9871
3.7639
3.7353
3.5797
3.5511
X : parts per Million : 1H

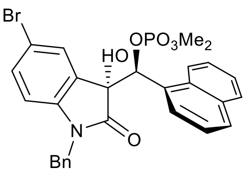

Br
OPO3Me2
HO
N
O
Bn

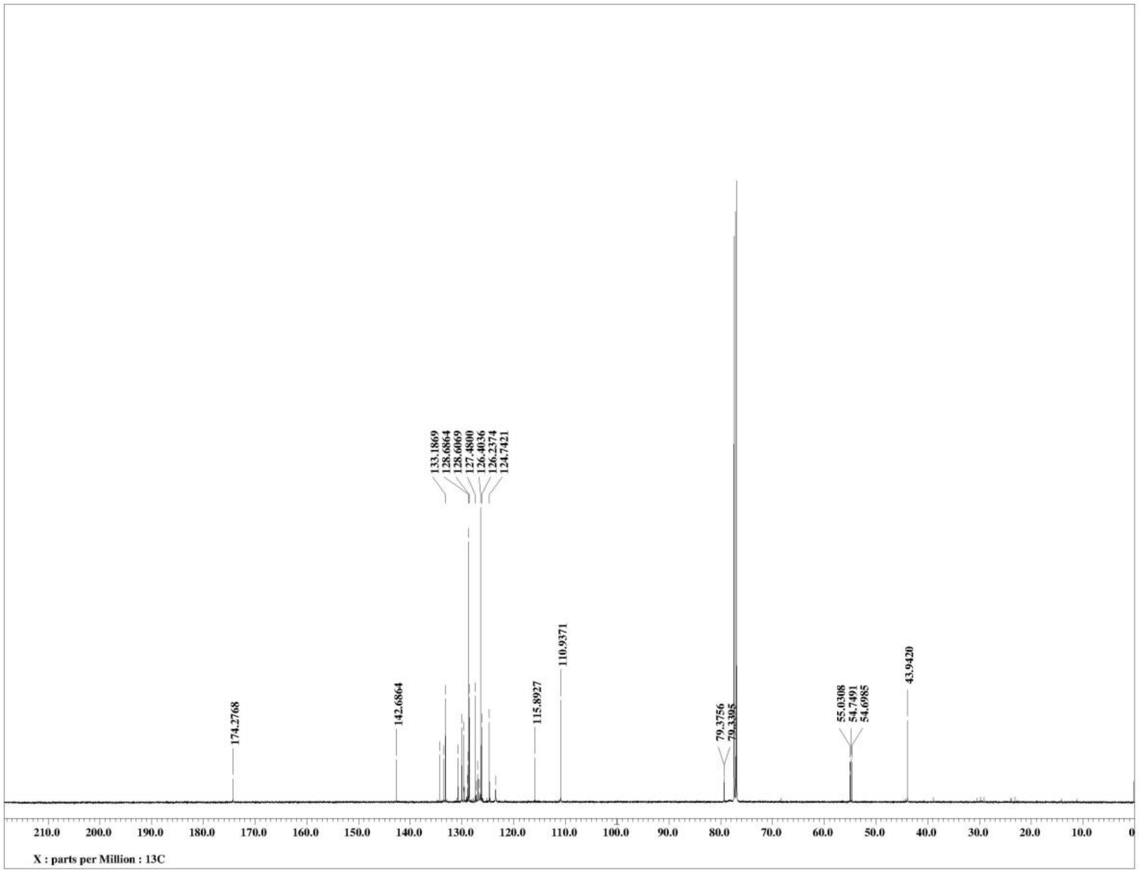

133.1869
128.6864
128.6069
127.4800
126.4036
126.2374
124.7421
174.2768
142.6864
115.8927
110.9371
79.3756
79.3395
55.0308
54.7491
54.6985
43.9420
210.0
200.0
190.0
180.0
170.0
160.0
150.0
140.0
130.0
120.0
110.0
100.0
90.0
80.0
70.0
60.0
50.0
40.0
30.0
20.0
10.0
0
X : parts per Million : 13C
